# 2L-PCA: a two-level principal component analyzer for quantitative drug design and its applications

**DOI:** 10.18632/oncotarget.19757

**Published:** 2017-08-01

**Authors:** Qi-Shi Du, Shu-Qing Wang, Neng-Zhong Xie, Qing-Yan Wang, Ri-Bo Huang, Kuo-Chen Chou

**Affiliations:** ^1^ State Key Laboratory of China for Biomass Energy Enzyme Technology, National Engineering Research Center of China for Non-Food Biorefinery, Guangxi Academy of Sciences, Nanning 530007, China; ^2^ School of Pharmacy, Tianjin Medical University, Tianjin 300070, China; ^3^ Center for Informational Biology, University of Electronic Science and Technology of China, Chengdu 610054, China; ^4^ Gordon Life Science Institute, Boston, MA 02478, USA

**Keywords:** drug design, PCA, molecular fragments, physicochemical properties, peptides

## Abstract

A two-level principal component predictor (2L-PCA) was proposed based on the principal component analysis (PCA) approach. It can be used to quantitatively analyze various compounds and peptides about their functions or potentials to become useful drugs. One level is for dealing with the physicochemical properties of drug molecules, while the other level is for dealing with their structural fragments. The predictor has the self-learning and feedback features to automatically improve its accuracy. It is anticipated that 2L-PCA will become a very useful tool for timely providing various useful clues during the process of drug development.

## INTRODUCTION

With the fast developments of computer-aided drug design (CADD) [1–5], currently a number of drug design approaches are developed, and several computer software packs [6, 7] are available that can speed up the discovery of new chemical and biological drugs in more efficient and economical procedure. However, so far we still have no perfect theories, ideal technologies, and faultless software tools that can guarantee complete success of the designed drugs due to the complicity of the interactions between medicinal drugs and their biological targets [8, 9]. The factors and parameters that may affect the bioactivities of drugs are not only from the structure of drug itself, but also from its biological target, coenzymes, and interaction environment [10, 11].

Principal component analysis (PCA) [12–14] is a useful tool that has been widely used in chemistry, biology, environment, and many fields of social science. The PCA approach has also been used in drug design for many years. Traditionally PCA is a single level and one direction prediction and analysis technique, described as the following equation

$$\sum_{k=1}^{K}\left(a_{k}x_{i,k}\right)=w_{i}$$ (1)

where $\left\{x_{i,k}\right\}$ are the physicochemical parameters of the *i*-th molecule, $\left\{a_{k}\right\}$ are the coefficients of molecular parameters, and *w*_*i*_ is bioactivity of the *i*-th molecule [15, 16]. The bioactivity *w*_*i*_ could be logarithm of IC_50,*i*_ (pIC_50,*i*_=-logIC_50,*i*_), or binding free energy Δ*G*°_*i*_ between drug and receptor.

After the coefficients $\left\{a_{k}\right\}$ of parameters are solved from the linear equations Eq.1 in a training set of drug candidates, the parameter coefficients $\left\{a_{k}\right\}$ can be used to predict the bioactivities of the designed or newly synthesized drug compounds,

$$w_{i}^{\text{pred}}=\sum_{k=1}^{K}\left(a_{k}x_{i,k}\right)$$ (2)

where *K* is the total number of molecular parameters. Currently hundreds even thousands of molecular parameters are available for drug design [17, 18]. However for certain drug-receptor interaction system, these parameters are not equally important; actually too many parameters may cause the over correlation problem [19, 20]. In PCA technique only the principle components are selected to describe the bioactivities of drug molecules, and to predict the bioactivities of drug candidates.

In the present study, an improved principal component analysis method, the so-called two-level principal component analysis (2L-PCA), is proposed to deal with the extreme complexity and huge amount of parameters in drug design and discovery. In the 2L-PCA predictor, the 1^st^ level is to deal with the physicochemical properties of drug molecules, and the 2^nd^ level is to deal with the fragments of molecular structures. The proposed two-level model can not only significantly enhance the prediction power, but also yield more useful information for in-depth analysis.

According to Chou’s 5-step rule [21] that has been widely used by many investigators (see, e.g., [22–37]), to develop a really useful statistical predictor, one should consider the following five procedures: (1) benchmark dataset; (2) sample representation; (3) operation algorithm; (4) cross validation; (5) web-server. Below, let us describe how to deal with them one-by-one. However, to comply with the Journal’s rubric style, they are not exactly following the aforementioned order.

## RESULTS AND DISCUSSION

As an example to show the advantage of 2L-PCA, we applied it for predicting the binding affinity of epitope-peptides with class I MHC molecules HLA-A*0201 [38, 39]. HLA-A*0201 is one of the most frequent class I alleles found in many different species and populations, which plays a critical role for antigen presentation in both viral antigens [40] and tumor antigens from a variety of cancers [41–44], and is expressed in approximately 50% of Caucasians population [45].

The epitope-peptides consist of nine amino acids [38, 39]. In the 2L-PCA study for the epitope-peptides, the nine side chains of the nine amino acids are the nine fragments. Eight physicochemical properties are used as the descriptors of the 20 natural amino acids. Four of them are the HMLP parameters [15, 16], describing the lipophilic character, hydrophilic character, surface area with lipophilic potential, and surface area with hydrophilic potential, respectively. The fifth property is the volume of amino acid side chains. The remaining three properties are the secondary structural potency indices of amino acids: the α-potency, β-potency, and coil-potency [46]. Listed in Table 1 are the eight physicochemical parameters of 20 amino acids used in this study.

**Table 1:** Eight physicochemical parameters^a^ of 20 natural amino acid side chains

A.A.	Lip	Hyd	S_L_ (Å^2^)	S_H_ (Å^2^)	P_α_	P_β_	P_c_	V(Å^3^)
Leu (L)	1.2906	0.0000	84.5476	0.0000	1.21	1.30	0.68	166.7
Ile (I)	1.1046	0.0000	88.6055	0.0000	1.08	1.60	0.66	166.7
Val (V)	0.5324	0.0000	77.8108	0.0000	1.06	1.70	0.62	140.0
Phe (F)	0.4412	-0.1195	105.7054	11.2472	1.13	1.38	0.71	189.9
Met (M)	1.0768	-0.3068	70.3631	23.2299	1.45	1.05	0.58	162.9
Trp (W)	0.8364	-0.4310	133.6980	14.8820	1.08	1.37	0.75	227.8
Ala (A)	0.1744	0.0000	34.7760	0.0000	1.42	0.83	0.70	88.6
Cys (C)	0.2479	-0.2402	23.5563	30.4540	0.70	1.19	1.18	108.5
Gly (G)	0.0208	0.0000	3.7616	0.0000	0.57	0.75	1.50	60.1
Tyr (Y)	0.4534	-0.5896	80.9646	42.7160	0.69	1.47	1.06	193.6
Thr (T)	1.4265	-0.4369	46.7285	16.0490	0.83	1.19	1.07	116.1
Ser (S)	0.2346	-0.6040	26.0681	15.9613	0.77	0.75	1.32	89.0
His (H)	0.8124	-0.7766	82.1701	13.8631	1.00	0.87	1.06	153.2
Gln (Q)	1.0036	-0.7211	70.0876	17.8662	1.11	1.10	0.86	143.9
Lys (K)	1.4600	-0.6229	97.7144	8.0786	1.16	0.74	0.98	168.7
Asn (N)	0.6396	-0.7211	50.5075	17.7804	0.67	0.89	1.35	117.7
Glu (E)	1.0315	-0.9298	57.1582	25.5726	1.51	0.37	0.84	138.4
Asp (D)	0.6058	-0.9298	37.4173	25.2736	1.01	0.54	1.20	111.1
Arg (R)	1.2424	-1.4797	90.8008	35.3095	0.98	0.93	1.04	173.4
Pro (P)	0.3226	0.0000	69.2297	0.0000	0.57	0.55	1.59	122.7

Lip: lipophilic index; Hyd: hydrophilic index; S_L_: lipophilic surface area; S_H_: hydrophilic surface area; P_α_: potency of α-helix; P_β_: potency of β-band; P_**c**_: potency of loop; V: volume of side chains.

In this study the HMLP parameters were used to describe the lipophilicity and hydrophilicity of molecular fragments. In peptides the HMLP parameters of the 20 natural amino acid side chains are available from literatures. However, the HMLP parameters of common chemical molecular fragments have to be derived using complicated calculations. In such cases other hydrophobic parameters can be used, e.g., the atom-based hydrophobic parameters in [47].

To reduce computational time, the cross validation in this study was performed via the independent dataset test [48], as described as follows. The sequences and experimental binding affinities of the 90 peptides were used as the training dataset to train the model, while those of the 40 peptides taken from [49] as the independent dataset to test the model. Actually, such 40 peptides had also been compiled in a series of publications [41, 42, 50–55]. The logarithms (pIC_50_) of IC_50_ were used as the bioactivity, because they are related to the changes in the free binding energy [55, 56]. Listed in Table 2 are the sequences and the experimental pIC_50_ of the peptides used in the training set. The binding strength of the 90 training peptides and 40 testing peptides covers the low, intermediate, and high affinity. The following two criteria were applied in the choice of the testing peptides: **(1)** the range of binding affinities in the testing dataset should not exceed the range of affinities in the training set; **(2)** the amino acid at each position in the testing dataset should also be present at that position in the training set of peptides. These two conditions make the 130 peptides to be the ideal benchmark dataset for 2L-PCA method.

**Table 2:** Amino acid sequences and experimental and predicted bioactivities of 90 MHC-I peptides in the training set

No.	Peptide sequence	Expt pIC_50_	Pred pIC_50_	pIC_50_ Diff	No.	Peptide sequence	Expt pIC_50_	Pred pIC_50_	pIC_50_ Diff
1	VALVGLFVL	5.148	5.7543	-0.6063	46	VVMGTLVAL	7.174	7.3163	-0.1423
2	GTLVALVGL	5.342	5.9368	-0.5948	47	YLEPGPVTI	7.187	7.1654	0.0216
3	LQTTIHDII	5.501	5.8143	-0.3133	48	GLSRYVARL	7.248	7.4620	-0.2131
4	SLHVGTQCA	5.842	6.1580	-0.3160	49	LLAQFTSAI	7.301	7.4302	-0.1292
5	ALPYWNFAT	5.869	6.6416	-0.7726	50	VLLDYQGML	7.328	7.5911	-0.2631
6	SLNFMGYVI	5.881	5.9560	-0.0750	51	YLEPGPVTV	7.342	7.4078	-0.0658
7	NLQSLTNLL	6.000	6.6992	-0.6992	52	ILSPFMPLL	7.3470	7.1400	0.2070
8	FVTWHRYHL	6.025	5.7230	0.3020	53	YLSPGPVTA	7.383	7.5610	-0.1780
9	DPKVKQWPL	6.176	5.7407	0.4354	54	IIDQVPFSV	7.398	7.6528	-0.2548
10	ITSQVPFSV	6.196	6.5888	-0.3928	55	SVYDFFVWL	7.444	7.3654	0.0786
11	ALAKAAAAI	6.211	6.2433	-0.0323	56	ITWQVPFSV	7.463	7.4417	0.0213
12	GLGQVPLIV	6.301	6.5651	-0.2641	57	ITYQVPFSV	7.480	7.6613	-0.1813
13	MLDLQPETT	6.335	6.8570	-0.5220	58	GLYSSTVPV	7.481	7.6303	-0.1493
14	LLSSNLSWL	6.342	6.3502	-0.0082	59	VMGTLVALV	7.553	7.2369	0.3161
15	GLACHQLCA	6.380	6.0594	0.3206	60	LLLCLIFLL	7.585	7.1406	0.4444
16	LIGNESFAL	6.415	7.0559	-0.6409	61	SLDDYNHLV	7.585	7.1764	0.4086
17	ALAKAAAAV	6.419	6.4857	-0.0667	62	VLIQRNPQL	7.644	6.9473	0.6967
18	LLAVGATKV	6.477	6.5115	-0.0344	63	SLYADSPSV	7.658	7.7106	-0.0526
19	ALAKAAAAL	6.511	6.2262	0.2848	64	ILSQVPFSV	7.699	7.6472	0.0518
20	WILRGTSFV	6.556	6.9084	-0.3524	65	IMDQVPFSV	7.719	8.0305	-0.3115
21	IISCTCPTV	6.580	6.6649	-0.0849	66	QLFEDNYAL	7.764	7.4713	0.2927
22	FLGGTPVCL	6.623	6.8756	-0.2526	67	ALMDKSLHV	7.770	7.5250	0.2450
23	ALIHHNTHL	6.623	6.7908	-0.1677	68	YAIDLPVSV	7.796	7.6075	0.1885
24	NLSWLSLDV	6.639	6.0466	0.5924	69	FVWLHYYSV	7.824	8.1149	-0.2909
25	YMIMVKCWM	6.663	6.6427	0.02035	70	MLGTHTMEV	7.845	7.3180	0.5270
26	VLQAGFFLL	6.682	7.0412	-0.3592	71	LLFGYPVYV	7.886	8.0253	-0.1393
27	GTLGIVCPI	6.714	6.5233	0.1907	72	ILKEPVHGV	7.921	7.5915	0.3295
28	VILGVLLLI	6.785	7.4728	-0.6878	73	YLMPGPVTV	7.932	7.9139	0.0181
29	VTWHRYHLL	6.793	6.5597	0.2333	74	WLDQVPFSV	7.939	7.9514	-0.0124
30	PLLPIFFCL	6.796	7.5217	-0.7257	75	KTWGQYWQV	7.955	7.6934	0.2616
31	TLGIVCPIC	6.815	5.9499	0.8651	76	ALMPLYACI	8.000	7.4383	0.5617
32	CLTSTVQLV	6.832	7.1061	-0.2741	77	YLAPGPVTA	8.032	7.6408	0.3912
33	ILLLCLIFL	6.845	6.7815	0.0635	78	YLYPGPVTV	8.051	8.3112	-0.2602
34	FAFRDLCIV	6.886	6.6689	0.2171	79	LLMGTLGIV	8.097	7.6769	0.4201
35	FLEPGPVTA	6.898	7.4940	-0.5960	80	YLWPGPVTV	8.125	8.0916	0.0334
36	ALAKAAAAA	6.947	6.8081	0.1389	81	FLLTRILTI	8.149	7.8796	0.2694
37	LMAVVLASL	6.954	7.4908	-0.5368	82	GLLGWSPQA	8.237	8.2184	0.0185
38	YVITTQHWL	6.983	6.3410	0.6420	83	ILYQVPFSV	8.310	8.7197	-0.4097
39	LLCLIFLLV	6.996	7.5015	-0.5055	84	GILTVILGV	8.347	7.8414	0.5056
40	ITAQVPFSV	7.020	6.6685	0.3515	85	NMVPFFPPV	8.398	8.0854	0.3126
41	YLEPGPVTL	7.058	7.1483	-0.0903	86	ILDQVPFSV	8.481	7.6904	0.7906
42	YTDQVPFSV	7.066	7.0742	-0.0082	87	YLFPGPVTA	8.495	8.3473	0.1477
43	NLYVSLLLL	7.114	6.9769	0.1371	88	YLDQVPFSV	8.638	8.1326	0.5054
44	ILHNGAYSL	7.127	7.3493	-0.2223	89	ILFQVPFSV	8.699	8.4335	0.2655
45	SIISAVVGI	7.159	7.3048	-0.1458	90	ILWQVPFSV	8.770	8.5002	0.2698

Statistical indices:

R=0.887132 R^2^= 0.787003 RES=0.366873 SEE=0.038672.

**Figure 1:**
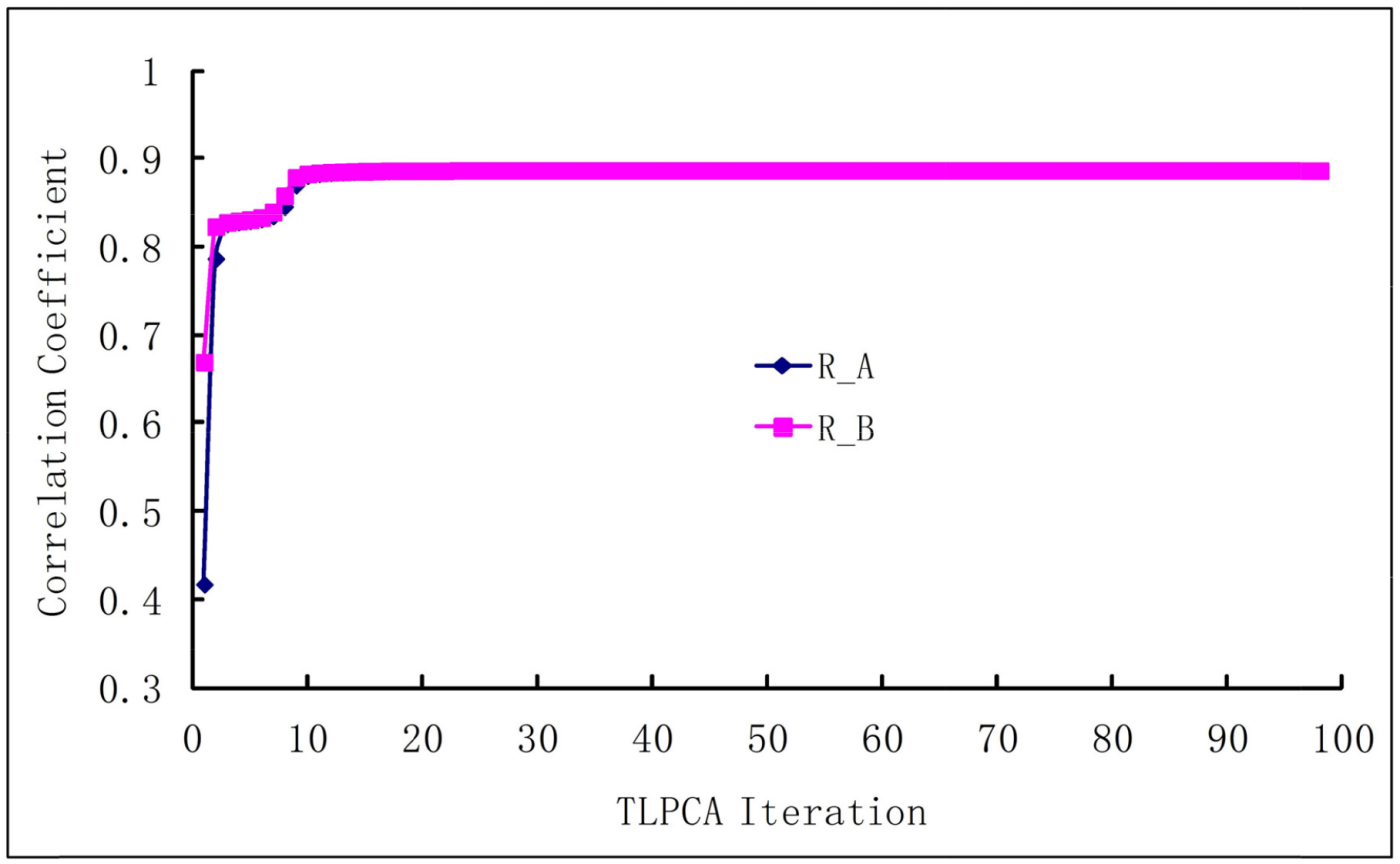
The correlation coefficients between experimental and predicted bioactivities increase with the iterations *R*_*a*_ is the correlation coefficient in the iterative procedure for $\left\{a_{k}^{\left(n\right)}\right\}$ of the physicochemical properties, and *R*_*b*_ is the correlation coefficient in the iterative procedure for $\left\{b_{l}^{\left(n\right)}\right\}$ of the molecular fragments.

**Figure 2:**
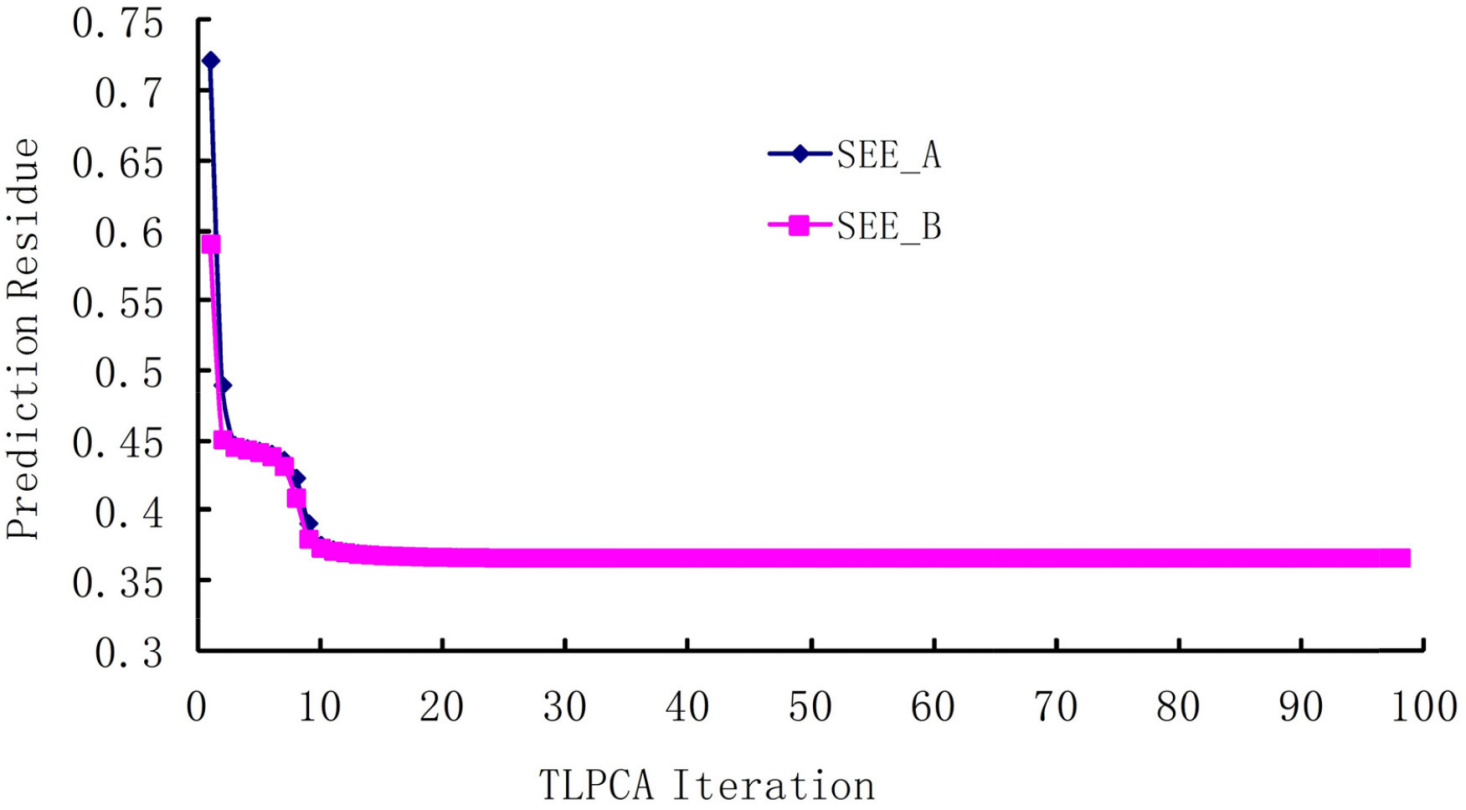
The residue between predicted bioactivities and experimental bioactivities in the iterative procedure The *Q* is the average square root of the summation of squared differences between predicted bioactivities and experimental bioactivities. *Q*_*a*_ is for $\left\{a_{k}^{\left(n\right)}\right\}$ iteration and *Q*_*b*_ is for $\left\{b_{l}^{\left(n\right)}\right\}$ iteration.

The iterative 2L-PCA technique described in Method section is used for the binding affinity study of peptides based on the sequences and experimental data listed in Table 2. The initial coefficient values $\left\{b_{l}^{\left(0\right)}\right\}$ of fragment parameters were assigned to 1, implying that all fragment parameters are equally important. Shown in Figure 1 are the curves of correlation coefficients *R vs* iterations , where the curve *R*_*a*_ is for the iteration of coefficients $\left\{a_{k}\right\}$, and the curve *R*_*b*_ is for the iterations of coefficients $\left\{b_{l}\right\}$. The average fitting error *Q* between the calculated bioactivities and the experimental bioactivities of peptides are shown in Figure 2, where *Q*_*a*_ is for $\left\{a_{k}\right\}$ iteration and *Q*_*b*_ for $\left\{b_{l}\right\}$ iteration. It has been observed that, after 10 to 12 iterations, the iterative result converged smoothly. The converged prediction coefficient sets $\left\{a_{k}^{\left(n\right)}\right\}$ and $\left\{b_{l}^{\left(n\right)}\right\}$ are given in Table 3. In the iterative solution precedure the correlation coefficien increases from the first value R_A_^(1)^=0.4167 to the converged value R_A_^(98)^=0.8871, and the prediction residue decreases from the first value Q_A_^(1)^=0.7223 to the converged value Q_A_^(98)^=0.0387.

**Table 3:** Prediction coefficients of eight physicochemical properties and nine residue positions obtained from the training set of MHC-I peptides

No.	Property	Coefficient	No.	Position	Coefficient
		$\left\{a_{k}\right\}$		(Residue)	$\left\{b_{kl}\right\}$
1	Lip	-0.02445	1	R1	2.53268
2	Hyd	0.19258	2	R2	8.36712
3	S^L^	-0.00212	3	R3	3.06856
4	S^H^	0.00348	4	R4	-4.89559
5	P_α_	0.15367	5	R5	3.12686
6	P_β_	0.07823	6	R6	2.45367
7	P_c_	0.19764	7	R7	1.24669
8	Vol	0.00366	8	R8	-3.50416
--	--	--	9	R9	-3.79249

The predicted pIC_50_ of the 40 queried peptides in the testing set are given in Table 4, which were predicted using the coefficients $\left\{a_{k}^{\left(n\right)}\right\}$ of properties and $\left\{b_{l}^{\left(n\right)}\right\}$ of fragments based on the eight physicochemical parameters and the nine fragments (amino acid side chains). The diversity of the peptides in the training set is very important for the prediction power of TLPC, especially for the residue positions at which we want to make prediction. It is expected that, with more experimental data available, the predictive power of 2L-PCA will be further improved. Actually, 30 prediction servers for human MHC-I peptide molecules were evaluated in a review article [57]. Among the 30 existing servers, 16 were ranked as the first class that provided the most accurate prediction results for MHC-I peptide molecules with the correlation coefficients ranging from r = 0.55 to r = 0.87. It has been shown in this study that the prediction correlation coefficient yielded by our 2L-PCA method is r = 0.868, being ranked around the very top of the first class.

**Table 4:** Amino acid sequences and experimental and predicted bioactivities of 40 MHC-I peptides in the testing set

No	Sequence	Expt pIC_50_	Pred pIC_50_	pIC_50_ Diff	No	Sequence	Expt pIC_50_	Pred pIC_50_	pIC_50_ Diff
1	LLGCAANWI	5.301	5.1708	0.1302	21	ITFQVPFSV	7.179	7.3750	-0.1960
2	SAANDPIFV	5.342	4.8592	0.4828	22	FTDQVPFSV	7.212	6.8379	0.3741
3	TTAEEAAGI	5.380	5.4678	-0.0878	23	RLMKQDFSV	7.342	7.5681	-0.2261
4	LTVILGVLL	5.580	5.3216	0.2584	24	KLHLYSHPI	7.352	6.6450	0.7070
5	HLLVGSSGL	5.792	6.4811	-0.6891	25	ITMQVPFSV	7.398	7.2641	0.1340
6	GIGILTVIL	6.000	5.7321	0.2679	26	KIFGSLAFL	7.478	6.7818	0.6962
7	TVILGVLLL	6.072	5.4662	0.6058	27	ALVGLFVLL	7.585	7.3852	0.1998
8	WTDQVPFSV	6.145	6.8930	-0.7480	28	YLSPGPVTV	7.642	7.2387	0.4033
9	AIAKAAAAV	6.176	6.4480	-0.2720	29	GLYSSTVPV	7.699	7.6303	0.0687
10	ILTVILGVL	6.419	7.0160	-0.5970	30	YLYPGPVTA	7.772	8.6335	-0.8615
11	AVAKAAAAV	6.495	5.9131	0.5819	31	YLAPGPVTV	7.818	7.3184	0.4996
12	ILDEAYVMA	6.623	7.4445	-0.8215	32	VVLGVVFGI	7.845	7.4509	0.3941
13	LLWFHISCL	6.682	6.3594	0.3226	33	MMWYWGPSL	7.921	7.4007	0.5203
14	TLDSQVMSL	6.793	7.2566	-0.4636	34	ILAQVPFSV	7.939	7.7270	0.2120
15	HLYQGCQVV	6.832	7.6799	-0.8479	35	FLLSLGIHL	8.053	8.1578	-0.1048
16	QLFHLCLII	6.886	7.6475	-0.7615	36	ILMQVPFSV	8.125	8.3225	-0.1975
17	ITDQVPFSV	6.947	6.6320	0.3150	37	YLFPGPVTV	8.237	8.0249	0.2121
18	ALCRWGLLL	7.000	7.2766	-0.2766	38	YLMPGPVTA	8.367	8.2363	0.1307
19	NLGNLNVSI	7.119	7.0974	0.02160	39	YLWPGPVTA	8.495	8.4140	0.0810
20	HLYSHPIIL	7.131	7.5663	-0.4353	40	FLDQVPFSV	8.658	7.8964	0.7616

Statistical indices:

R=0.867872 R^2^= 0.753202 RES=0.469728 SEE=0.074271.

2L-PCA neither needs knowing the exact comformations of the peptides nor needs aligning the peptides according to a template. The two steps are necessary but quite difficult for CoMFA [58, 59] and CoMSIA [60, 61] owing to that there are numerous possible conformations for peptides and that the experimental crystal structure for serving as a template is often not available. 2L-PCA method provides an alternate way for design of the chemical drugs and peptide drugs.

**Figure 3:**
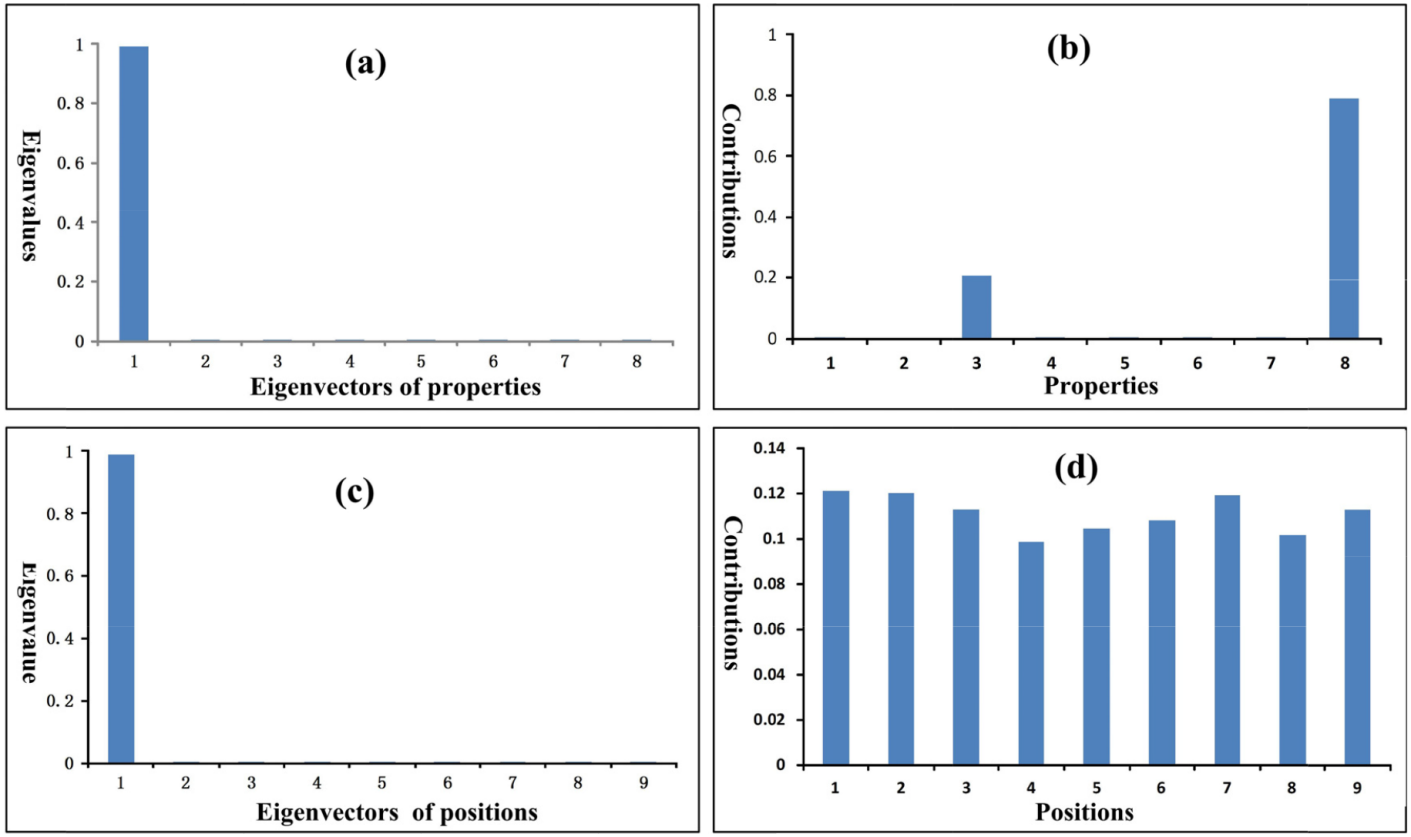
Eigenvalues and contributions of properties and peptide positions **(a)** The eigenvalues of property eigenvectors. **(b)** The contributions of properties to the eigenvalues. The volumes (Vol) and hydrophobic surface areas (S_L_) of amino acid side chains make the largest contributions. **(c)** The eigenvalues of peptide position eigenvectors. **(d)** The contributions of peptide positions to the eigenvalues. The contributions of all nine amino acid positions are almost equally important.

The eigenvalues and contributions of physicochemical properties and amino acid positions in peptides are summarized in Table 5 and shown in Figure 3. In Table 5 the eigenvalues are normalized. The eigenvalue portion of the first three property eigenvectors is almost 100%, and the eigenvalue portion of the first eigenvector alone is larger than 99%. Most contributions are made by the three properties: side chain volume (Vol), lipophilic surface area (S^L^), and hydrophilic surface area (S^H^), as shown in Figure 3b and Table 5. The contributions of other 5 properties seem very small. The eigenvalue of the first peptide position eigenvector is larger than 98%. In Table 5 the contributions of the nine amino acid positions are different. However the differences are not big, implying that all positions are almost equally important. The detailed computation results are given in Supplementary Information 1.

**Table 5:** Eigenvalues and contributions of physicochemical properties and amino acid positions in training set of peptides

Physicochemical properties				Positions (fragments)			
No.	Eigenvalue^a^	Property	Contribution	No	Eigenvalue ^a^	Position ^b^	Contribution
1	0.99118	Lip	0.00004	1	0.98873	Residue-1	0.12126
2	0.00641	Hyd	0.00000	2	0.00300	Residue-2	0.12043
3	0.00240	S^L^	0.20595	3	0.00249	Residue-3	0.1130
4	0.00005	S^H^	0.00534	4	0.00213	Residue-4	0.09871
5	0.00004	P_α_	0.00004	5	0.00106	Residue-5	0.1046
6	0.00003	P_β_	0.00005	6	0.00094	Residue-6	0.10804
7	0.00002	P_c_	0.00002	7	0.0007	Residue-7	0.11931
8	0.00001	Vol	0.78857	8	0.00058	Residue-8	0.10186
--	--	--	--	9	0.00037	Residue-9	0.11278

^a^ Eigenvalues are normalized.

^b^ The positions of peptides are equal to the fragments of molecules.

We are often facing two kinds of challenges in theoretical prediction for drug design: one is over-correlation problem, and the other is lack of information and explanation for the predicted results. The over-correlation problem is caused by large amount of parameters used in the prediction model, which may yield quite good correlation results in self-consistency test [62, 63], but very poor predicted results in independent dataset test owing to the high dimensional disaster [19] or “curse of dimensionality” problem. To solve this problem, the pseudo amino acid composition (PseAAC) was introduced [64]. Ever since then, the concept of PseAAC or the general PseAAC [21] has been widely used in drug development and biomedicine [65, 66] and nearly all the areas of computational proteomics (see, e.g., [67] as well as a long list of references cited in [68, 69]). Actually, the physicochemical properties used here can be regarded as some optimal pseudo components [70]. It is through such a PseAAC approach to remove the trivial parameters (or reduce the feature vector’s dimension) and grasp the key ones. Besides, the traditional prediction methods fail to provide a good explanation for the predicted results; i.e., how do the physicochemical properties and the structural changes affect the bioactivities? In contrast to that, the proposed “2L-PCA” method can provide more information about the impact of the physicochemical properties and molecular fragments to the bioactivities of drug candidates.

## MATERIALS AND METHODS

**Figure 4:**
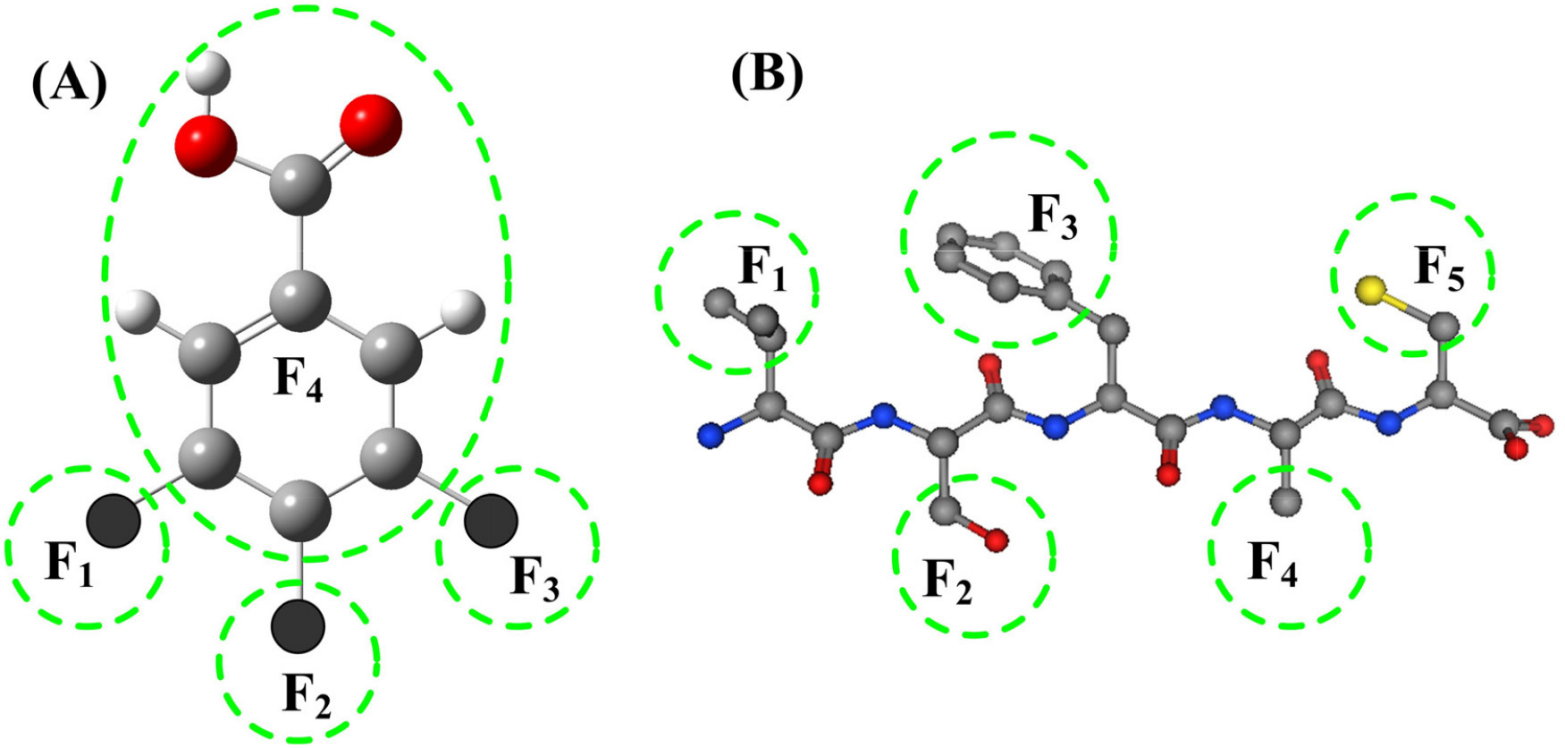
Illustration of molecular fragments **(A)** The structural fragments in neuraminidase (NA) of influenza virus A inhibitors. The molecular structure is divided into 4 fragments according to the substitutes being investigated. The fragments F_1_, F_2_ and F_3_ are three substituent groups, and the fragment F_4_ is the remaining part of the molecular parent. **(B)** In short peptides each side chain of amino acid residue is a fragment.

In practical drug design and development, usually the basic structure of drug candidates keep constant, only small modifications are made on several fragments. The structure parameters of the entire molecules cannot clearly describe the detailed characters of the small changes at individual fragments or substitutes. In the 2L-PCA model the molecular structures are separated into several fragments, and are described by a set of fragment parameters. An example of molecular structure and its fragments is shown in Figure 4A. The idea of molecular fragments also can be applied to the peptide drugs, in which each side chain of an amino acid is a fragment, as shown in Figure 4B.

### General 3D equation of 2L-PCA

In the 2L-PCA prediction model the bioactivity *w*_*i*_ of molecule *i* is the summation of contributions Δ*g*_*i,l*_ from all molecular fragments; i.e.,

$$\sum_{l=1}^{L}b_{l}{\Delta g}_{i,l}=w_{i}$$ (3)

where Δ*g*_*i,l*_ is the contribution of fragment *l* to the bioactivity *w*_*i*_ of molecule *i*, *b*_*l*_ is the prediction coefficient of fragment *l*, and *L* is the total number of molecular fragments. The contribution Δ*g*_*i,l*_ of fragment *l* is the summation of the contributions from all physicochemical properties of fragment *l*, namely

$${\Delta g}_{i,l}=\sum_{k=1}^{K}a_{k}x_{i,l,k}$$ (4)

where *x*_*i,l,k*_ is the physicochemical property *k* of fragment *l* in molecule *i*, *a*_*k*_ is the prediction coefficient of physicochemical property *k*, and *K* is the total number of physicochemical properties.

Inserting the Eq.4 into Eq.3 we get the general equation of 2L-PCA prediction model as given by

$$\begin{array}{c} \sum_{l=1}^{L}b_{l}\left(\sum_{k=1}^{K}a_{k}X_{i,l,k}\right)=w_{i}\\ \left(i=1,2\ldots \ldots ,N\right) \end{array}$$ (5)

where *N* is the total number of molecular samples. Eq.5 can be expressed in vector and matrix form as given below

$$X_{N,L,K}B_{L}A_{K}=W_{N}$$ (6)

where **X**_N,L,K_ is the three dimensional (3D) data matrix of molecular parameters, **W**_N_ is the bioactivity column vector of molecular samples, **B**_L_ is the coefficient vector of fragments, and **A**_K_ is the coefficient vector of physicochemical properties.

### 2D equations of properties and fragments

The general three-dimensional 2L-PCA equation of Eq.6 can be reduced to two 2D equations with the following algebra operations,

$$X_{N,L,K}A_{K}=H_{N,L}$$ (7)

where **H**_N,L_ is the 2D data matrix of molecular fragments. Substituting **H**_N,L_ into Eq.6, we obtain the following fragment 2D equation

$$H_{N,L}B_{L}=W_{N}$$ (8)

Likewise, the property 2D equation can also be expressed as

$$X_{N,L,K}B_{L}=F_{N,K}$$ (9)

and

$$F_{N,K}A_{K}=W_{N}$$ (10)

where **F**_N,K_ is the 2D data matrix of physicochemical properties.

### Algebra solutions of property and fragment 2D equations

The fragment 2D equation Eq.8 and the property 2D equation Eq.10 can be solved using the standard algebra method. Both sides of the fragment 2D equation of Eq.8 are multiplied with the transposed matrix **H**^t^_N,L_ from left, it follows that

$$H_{N,L}^{t}H_{N,L}=U_{L,L}$$ (11)

and

$$H_{N,L}^{t}W_{N}=S_{L}$$ (12)

Thus, we get the following symmetrically square matrix equation of fragments

$$U_{L,L}B_{L}=S_{L}$$ (13)

Since the fragment square matrix equation of Eq.13 is multiplied by its inverse matrix **U**^-1^_L,L_, the prediction coefficients **B**_L_ for the fragments are obtained, as given below

$$B_{L}=U_{L,L}^{-1}S_{L}$$ (14)

where the inverse matrix **U**^-1^_L,L_ can be obtained by solving the eigen equation [71] [48] of **U**_L,L_, namely the equation

$$U_{L,L}\Psi_{L,L}=\alpha_{L,L}\Psi_{L,L}$$ (15)

meaning

$$U_{L,L}^{-1}=\frac{1}{\alpha_{L}}\Psi_{L,L}$$ (16)

where **Ψ**_L,L_ is the eigenvectors and α_L_ is the eigenvalues of fragment square matrix **U**_L,L_[72, 73].

Similarly, left-multiplying both sides of property 2D equation of Eq.10 with **F**^t^_N,K_, we have

$$F_{\mathrm{N,K}}^{t}F_{\mathrm{N,K}}=V_{\mathrm{K,K}}$$ (17)

and

$$F_{N,K}^{t}W_{N}=T_{K}$$ (18)

From Eqs.17-18, we get the following square matrix equation of properties

$$V_{K,K}A_{K}=T_{K}$$ (19)

Multiplying Equation Eq.19 with the inverse matrix **V**^-1^_K,K_, will give the solution of property prediction coefficients **A**_K_; i.e.

$$A_{K}=V_{K,K}^{-1}T_{K}$$ (20)

Thus, the inverse matrix **V**^-1^_K,K_ is obtained by solving the eigen equation of property square matrix **V**_K,K_:

$$V_{K,K}\Phi_{K,K}=\beta_{K}\Phi_{K,K}$$ (21)

and

$$V_{K,K}^{-1}=\frac{1}{\beta_{K}}\Phi_{K,K}$$ (22)

where **Φ**_K,K_ is the eigen-vectors and **β**_k_ is the eigen-values of the property square matrix **V**_K,K_.

### Iterative solution of 2L-PCA equations

In the training dataset for drug candidates the two prediction coefficients set **A**_K_ and **B**_L_ in the 2L-PCA general equation Eq.6 are solved in an iterative procedure [74, 75]. Firstly the initial fragment coefficients **B**^(0)^_L_ are assigned to 1 {b_*i*_=1, *i*=1,2…,L}, implying all fragments are equally important. The initial **B**^(0)^_L_ are used in the property 2D equations Eq (9) and (10), thus the first solution of property coefficients **A**^(1)^_K_ is obtained by solving the eigen-equations Eq.17-20. Then the property coefficients **A**^(1)^_K_ are used in the fragment equations Eqs.7-8, and the first solution of fragment coefficients **B**^(1)^_L_ are obtained by solving eigen-equations Eq.11-14. In the next iterative cycle the **B**^(1)^_L_ is used to find the **A**^(2)^_K_. Above iterative procedure is repeated for *n* times, until to reaching a threshold value ε; i.e.,

$$\left|Q^{\left(n+1\right)}-Q^{\left(n\right)}\right|=\left|\sqrt{\frac{1}{N}\sum_{i=1}^{N}{\left(w_{i}^{\mathrm{expt}}-w_{i}^{\left(n+1\right)}\right)}^{2}}-\sqrt{\frac{1}{N}\sum_{i=1}^{N}{\left(w_{i}^{\mathrm{expt}}-w_{i}^{\left(n\right)}\right)}^{2}}\right|\leq \varepsilon$$ (23)

The bioactivities of designed drugs and newly synthesized drug candidates are predicted using the converged coefficients $\left\{a_{k}^{\left(n\right)}\right\}$ and $\left\{b_{l}^{\left(n\right)}\right\}$ as given below

$$w_{i}^{\text{pred}}=\sum_{l=1}^{L}b_{l}^{\left(n\right)}\left(\sum_{k=1}^{K}a_{k}^{\left(n\right)}x_{i,l,k}\right)$$ (24)

**Figure 5:**
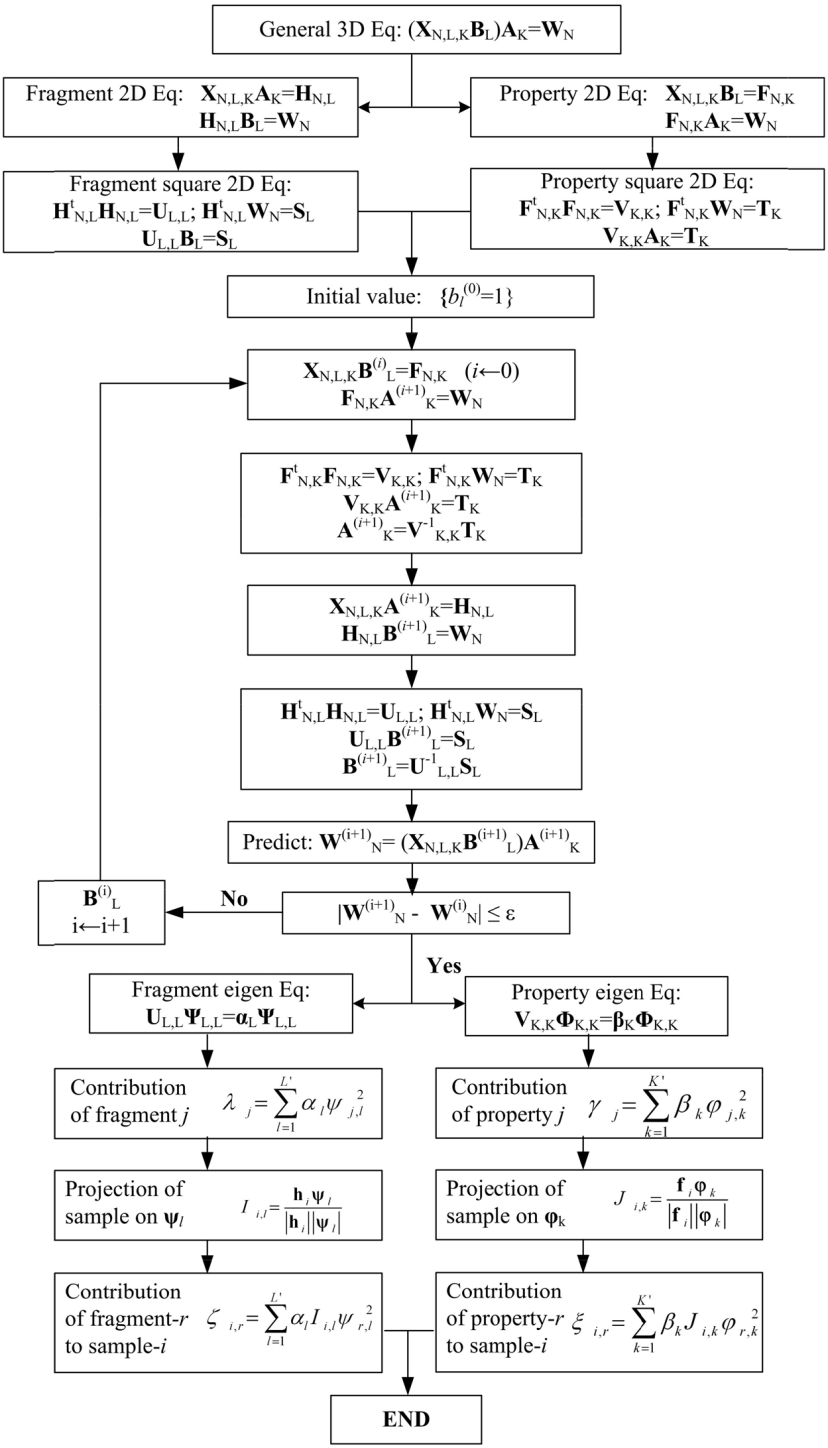
The iterative algebra solution procedure of the solution of 2L-PCA prediction model for the two sets of coefficients {*a*_*k*_^(*n*)^} and {*b*_*l*_^(*n*)^}, where *N* is the number of molecular samples, *L* is the number of fragments in molecules, *L*′ is the principal number of fragments, *K* is the number of physicochemical properties, and *K*′ is the principal number of properties

Illustrated in Figure 5 is the iterative solution procedure for the 2L-PCA predictor.

### Principal component analysis of properties and fragments

The property eigenvectors $\left\{\varphi_{k}\right\}$ are orthogonal and normalized; i.e.,

$$\varphi_{k}\;\varphi_{j}=0\left(k\neq j\right)$$ (25)

and

$$\varphi_{k}\;\varphi_{k}=\sum_{j=1}^{K}\varphi_{j,k}^{2}=1$$ (26)

where the term φ^2^_*j,k*_ is the component of the *j*-th property in the *k*-th eigen-vector **φ**_***k***_. The first K′ property eigen-vectors are the principal components whose eigen-values are larger than a threshold (e.g., ε=90% or 95%); i.e.,

$$\frac{\sum_{K=1}^{K^{\prime }}\beta_{k}^{2}}{\sum_{k=1}^{K}\beta_{k}^{2}}\geq \varepsilon$$ (27)

The total contribution γ_*j*_ of the *j*-th property to the bioactivity of molecular samples in training set is defined as the following summation,

$$\gamma_{j}=\sum_{K=1}^{K^{\prime }}\beta_{k}\varphi_{j,k}^{2}$$ (28)

The property eigen-vectors $\left\{\varphi_{k}\right\}$ span an orthogonal multiple space, in which a drug molecule P_*i*_ is a vector, and its projection *J*_*i,k*_ on the *k*-th property-eigenvector **φ**_*k*_ is calculated by

$$J_{i,k}=\frac{f_{i}.\phi_{k}}{\left|f_{i}\right|\left|\phi_{k}\right|}=\frac{\sum_{j=1}^{K}f_{i,j}\varphi_{j,k}}{\sqrt{\sum_{j=1}^{K}f_{i,j}^{2}}\sqrt{\sum_{j=1}^{K}\phi_{i,j}^{2}}}$$ (29)

where **f**_*i*_ is the *i*-th row vector of the property matrix **F**_NK_ of Eq.9. In the projection *J*_*i,k*_ of molecular sample P_*i*_ on the *k*-th property-eigenvector **j**_*k*_ the component of the *r*-th property is α_*k*_φ^2^_*r,k*_, therefore the total contribution of *r*-th property to the sample P_*i*_ is the summation of components from all principal property eigenvectors, namely

$$\xi_{i,r}=\sum_{k=1}^{K^{\prime }}\beta_{k}J_{i,k}\varphi_{r,k}^{2}$$ (30)

Similarly, the fragment eigenvectors ψ_*l*_ span an L-dimensional orthogonal space. The first L′ fragment eigenvectors are the principal components. The total contribution factor λ_*j*_ of the *j*-th fragment to the bioactivity of peptide set is given by

$$\lambda_{j}=\sum_{l=1}^{L^{\prime }}\alpha_{l}\psi_{j,l}^{2}$$ (31)

In the same way the projection *I*_*i,l*_ of sample P_*i*_ on the *l*-th fragment-eigenvector ψ_*l*_ can be calculated by

$$I_{i,l}=\frac{h_{i}.\phi_{l}}{\left|h_{i}\right|\left|\phi_{l}\right|}=\frac{\sum_{j=1}^{L}h_{i,j}\psi_{j,l}}{\sqrt{\sum_{j=1}^{L}h_{i,j}^{2}}\sqrt{\sum_{j=1}^{L}\psi_{i,j}^{2}}}$$ (32)

where **h**_*i*_ is the *i*-th row vector of the fragment matrix **H**_NL_ of Eq.7. In the projection *I*_*i,l*_ of molecule P_*i*_ on the *l*-th fragment-eigenvector **φ**_*l*_ the component of the *r*-th fragment is α_*l*_ψ^2^_*r,l*_, therefore the total contribution of *r*-th fragment to the sample P_*i*_ is the summation of components from all principal fragment eigenvectors; i.e.,

$$\varsigma_{i,r}=\sum_{l=1}^{L^{\prime }}\alpha_{l}I_{i,l}\psi_{r,l}^{2}$$ (33)

### Web-server

As pointed out in [76], user-friendly and publicly accessible web-servers represent the future direction for developing practically more useful predictors or any computational tools. Actually, user-friendly web-servers as given in a series of recent publications [23-25, 30, 32, 34-36, 69, 70, 77-91] will significantly enhance the impacts by attracting the broad experimental scientists [66, 92]. We will do our best to establish a web-server for 2L-PCA as soon as possible. Once it has been done, an announcement will be made thorough a publication or our webpage.

## CONCLUSION

The 2L-PCA predictor proposed in this paper is a very useful tool for drug design. Its advantages can be summarized as follows. (1) With 2L-PCA, the molecular structures of drug candidates can be separated into several fragments described by physicochemical parameters of the molecular fragments, thus the small modifications on individual fragments can be clearly shown. (2) Its two prediction coefficient sets $\left\{a_{k}\right\}$ of properties and $\left\{b_{l}\right\}$ of fragments can be solved in an iterative procedure, which possesses self-learning ability and information feed-back function in certain degree, and hence greatly promoting the prediction power of 2L-PCA. (3) It possesses the information from both of the structures of molecular fragments and the physicochemical properties, able to significantly improve the drug candidates in both the structure and property. (4) Its elegant algebra solution procedure will be very useful for further enhancing the ability of principal component analysis (PCA).

## SUPPLEMENTARY MATERIALS




