# Supplementary material for: 2L-PCA: a two-level principal component analyzer for quantitative drug design and its applications
Source: Oncotarget. 2017 Aug 1;8(41):70564–78. doi: 10.18632/oncotarget.19757 (PMC5642577; doi:10.18632/oncotarget.19757)
Supplement: Supplementary file 1 [file oncotarget-08-70564-s001.pdf]

# 2L-PCA: a two-level principal component analyzer for quantitative drug design and its applications

## SUPPLEMENTARY MATERIALS

**Supplementary Information 1: It consists of two types of calculation results: (1) the predicted results by 2L-PCA, and (2) the analyzed results by 2L-PCA.**

See Supplementary File 1
