# Supplementary material for: 2L-PCA: a two-level principal component analyzer for quantitative drug design and its applications
Source: Oncotarget. 2017 Aug 1;8(41):70564–78. doi: 10.18632/oncotarget.19757 (PMC5642577; doi:10.18632/oncotarget.19757)
Supplement: Supplementary file 2 [file oncotarget-08-70564-s002.docx]

**Supplementary Information 1: It consists of two types of calculation results**. (**1**) Predict_90_40_9_8.RSL contains the predicted results by 2L-PCA, and (**2**) TLPCA_90_40_9_8.RSL contains the analyzed results by 2L-PCA.

1. Predict_90_40_9_8.RSL

*** Input Peptide DATA ***

NP1, NP2, NAAP, NPARA: 90 40 9 8

Sample_Peptide pIC50s

1 VALVGLFVL 5.14800

2 GTLVALVGL 5.34200

3 LQTTIHDII 5.50100

4 SLHVGTQCA 5.84200

5 ALPYWNFAT 5.86900

6 SLNFMGYVI 5.88100

7 NLQSLTNLL 6.00000

8 FVTWHRYHL 6.02500

9 DPKVKQWPL 6.17600

10 ITSQVPFSV 6.19600

11 ALAKAAAAI 6.21100

12 GLGQVPLIV 6.30100

13 MLDLQPETT 6.33500

14 LLSSNLSWL 6.34200

15 GLACHQLCA 6.38000

16 LIGNESFAL 6.41500

17 ALAKAAAAV 6.41900

18 LLAVGATKV 6.47700

19 ALAKAAAAL 6.51100

20 WILRGTSFV 6.55600

21 IISCTCPTV 6.58000

22 FLGGTPVCL 6.62300

23 ALIHHNTHL 6.62300

24 NLSWLSLDV 6.63900

25 YMIMVKCWM 6.66300

26 VLQAGFFLL 6.68200

27 GTLGIVCPI 6.71400

28 VILGVLLLI 6.78500

29 VTWHRYHLL 6.79300

30 PLLPIFFCL 6.79600

31 TLGIVCPIC 6.81500

32 CLTSTVQLV 6.83200

33 ILLLCLIFL 6.84500

34 FAFRDLCIV 6.88600

35 FLEPGPVTA 6.89800

36 ALAKAAAAA 6.94700

37 LMAVVLASL 6.95400

38 YVITTQHWL 6.98300

39 LLCLIFLLV 6.99600

40 ITAQVPFSV 7.02000

41 YLEPGPVTL 7.05800

42 YTDQVPFSV 7.06600

43 NLYVSLLLL 7.11400

44 ILHNGAYSL 7.12700

45 SIISAVVGI 7.15900

46 VVMGTLVAL 7.17400

47 YLEPGPVTI 7.18700

48 GLSRYVARL 7.24800

49 LLAQFTSAI 7.30100

50 VLLDYQGML 7.32800

51 YLEPGPVTV 7.34200

52 ILSPFMPLL 7.34700

53 YLSPGPVTA 7.38300

54 IIDQVPFSV 7.39800

55 SVYDFFVWL 7.44400

56 ITWQVPFSV 7.46300

57 ITYQVPFSV 7.48000

58 GLYSSTVPV 7.48100

59 VMGTLVALV 7.55300

60 LLLCLIFLL 7.58500

61 SLDDYNHLV 7.58500

62 VLIQRNPQL 7.64400

63 SLYADSPSV 7.65800

64 ILSQVPFSV 7.69900

65 IMDQVPFSV 7.71900

66 QLFEDNYAL 7.76400

67 ALMDKSLHV 7.77000

68 YAIDLPVSV 7.79600

69 FVWLHYYSV 7.82400

70 MLGTHTMEV 7.84500

71 LLFGYPVYV 7.88600

72 ILKEPVHGV 7.92100

73 YLMPGPVTV 7.93200

74 WLDQVPFSV 7.93900

75 KTWGQYWQV 7.95500

76 ALMPLYACI 8.00000

77 YLAPGPVTA 8.03200

78 YLYPGPVTV 8.05100

79 LLMGTLGIV 8.09700

80 YLWPGPVTV 8.12500

81 FLLTRILTI 8.14900

82 GLLGWSPQA 8.23700

83 ILYQVPFSV 8.31000

84 GILTVILGV 8.34700

85 NMVPFFPPV 8.39800

86 ILDQVPFSV 8.48100

87 YLFPGPVTA 8.49500

88 YLDQVPFSV 8.63800

89 ILFQVPFSV 8.69900

90 ILWQVPFSV 8.77000

Test_Peptides

1 LLGCAANWI 5.30100

2 SAANDPIFV 5.34200

3 TTAEEAAGI 5.38000

4 LTVILGVLL 5.58000

5 HLLVGSSGL 5.79200

6 GIGILTVIL 6.00000

7 TVILGVLLL 6.07200

8 WTDQVPFSV 6.14500

9 AIAKAAAAV 6.17600

10 ILTVILGVL 6.41900

11 AVAKAAAAV 6.49500

12 ILDEAYVMA 6.62300

13 LLWFHISCL 6.68200

14 TLDSQVMSL 6.79300

15 HLYQGCQVV 6.83200

16 QLFHLCLII 6.88600

17 ITDQVPFSV 6.94700

18 ALCRWGLLL 7.00000

19 NLGNLNVSI 7.11900

20 HLYSHPIIL 7.13100

21 ITFQVPFSV 7.17900

22 FTDQVPFSV 7.21200

23 RLMKQDFSV 7.34200

24 KLHLYSHPI 7.35200

25 ITMQVPFSV 7.39800

26 KIFGSLAFL 7.47800

27 ALVGLFVLL 7.58500

28 YLSPGPVTV 7.64200

29 GLYSSTVPV 7.69900

30 YLYPGPVTA 7.77200

31 YLAPGPVTV 7.81800

32 VVLGVVFGI 7.84500

33 MMWYWGPSL 7.92100

34 ILAQVPFSV 7.93900

35 FLLSLGIHL 8.05300

36 ILMQVPFSV 8.12500

37 YLFPGPVTV 8.23700

38 YLMPGPVTA 8.36700

39 YLWPGPVTA 8.49500

40 FLDQVPFSV 8.65800

Parameters of 20 Amino Acids: 8

Index Ala Arg Asn Asp Cys Gln Glu Gly His Ile Leu Lys Met Phe Pro Ser Thr Trp Tyr Val

Lip 0.1744 1.2424 0.6396 0.6058 0.2479 1.0036 1.0315 0.0208 0.8124 1.1046 1.2906 1.4600 1.0768 0.4412 0.3226 0.2346 1.4265 0.8364 0.4534 0.5324

Hyd 0.0000 -1.4797 -0.7211 -0.9298 -0.2402 -0.7211 -0.9298 0.0000 -0.7766 0.0000 0.0000 -0.6229 -0.3068 -0.1195 0.0000 -0.6040 -0.4369 -0.4310 -0.5896 0.0000

S_H 34.7760 90.8010 50.5080 37.4170 23.5560 70.0880 57.1582 3.7620 82.1700 88.6060 84.5480 97.7140 70.3631 105.7050 69.2300 26.0680 46.7290 133.6980 80.9650 77.8110

S_L 0.0000 35.3100 17.7800 25.2733 30.4540 17.8660 25.5730 0.0000 13.8630 0.0000 0.0000 8.0790 23.2300 11.2470 0.0000 15.9610 16.0490 14.8820 42.7610 0.0000

Pa 1.4200 0.9800 0.6700 1.0100 0.7000 1.1100 1.5100 0.5700 1.0000 1.0800 1.2100 1.1600 1.4500 1.1300 0.5700 0.7700 0.8300 1.0800 0.6900 1.0600

Pb 0.8300 0.9300 0.8900 0.5400 1.1900 1.1000 0.3700 0.7500 0.8700 1.6000 1.3000 0.7400 1.0500 1.3800 0.5500 0.7500 1.1900 1.3700 1.4700 1.7000

Pc 0.7000 1.0400 1.3500 1.2000 1.1200 0.8600 0.8400 1.5000 1.0600 0.6600 0.6800 0.9800 0.5800 0.7100 1.5900 1.3200 1.0700 0.7500 1.0600 0.6200

Vol 88.6000 173.4000 117.7000 111.1000 108.5000 143.9000 138.4000 60.1000 153.2000 166.7000 166.7000 168.7000 162.9000 189.9000 122.7000 89.0000 116.1000 227.8000 193.6000 140.0000

END of INPUT

3D DATA Matrix of Sample Peptides:

1 VALVGLFVL 5.1480

V 0.5324 0.0000 77.8110 0.0000 1.0600 1.7000 0.6200 140.0000

A 0.1744 0.0000 34.7760 0.0000 1.4200 0.8300 0.7000 88.6000

L 1.2906 0.0000 84.5480 0.0000 1.2100 1.3000 0.6800 166.7000

V 0.5324 0.0000 77.8110 0.0000 1.0600 1.7000 0.6200 140.0000

G 0.0208 0.0000 3.7620 0.0000 0.5700 0.7500 1.5000 60.1000

L 1.2906 0.0000 84.5480 0.0000 1.2100 1.3000 0.6800 166.7000

F 0.4412 -0.1195 105.7050 11.2470 1.1300 1.3800 0.7100 189.9000

V 0.5324 0.0000 77.8110 0.0000 1.0600 1.7000 0.6200 140.0000

L 1.2906 0.0000 84.5480 0.0000 1.2100 1.3000 0.6800 166.7000

2 GTLVALVGL 5.3420

G 0.0208 0.0000 3.7620 0.0000 0.5700 0.7500 1.5000 60.1000

T 1.4265 -0.4369 46.7290 16.0490 0.8300 1.1900 1.0700 116.1000

L 1.2906 0.0000 84.5480 0.0000 1.2100 1.3000 0.6800 166.7000

V 0.5324 0.0000 77.8110 0.0000 1.0600 1.7000 0.6200 140.0000

A 0.1744 0.0000 34.7760 0.0000 1.4200 0.8300 0.7000 88.6000

L 1.2906 0.0000 84.5480 0.0000 1.2100 1.3000 0.6800 166.7000

V 0.5324 0.0000 77.8110 0.0000 1.0600 1.7000 0.6200 140.0000

G 0.0208 0.0000 3.7620 0.0000 0.5700 0.7500 1.5000 60.1000

L 1.2906 0.0000 84.5480 0.0000 1.2100 1.3000 0.6800 166.7000

3 LQTTIHDII 5.5010

L 1.2906 0.0000 84.5480 0.0000 1.2100 1.3000 0.6800 166.7000

Q 1.0036 -0.7211 70.0880 17.8660 1.1100 1.1000 0.8600 143.9000

T 1.4265 -0.4369 46.7290 16.0490 0.8300 1.1900 1.0700 116.1000

T 1.4265 -0.4369 46.7290 16.0490 0.8300 1.1900 1.0700 116.1000

I 1.1046 0.0000 88.6060 0.0000 1.0800 1.6000 0.6600 166.7000

H 0.8124 -0.7766 82.1700 13.8630 1.0000 0.8700 1.0600 153.2000

D 0.6058 -0.9298 37.4170 25.2733 1.0100 0.5400 1.2000 111.1000

I 1.1046 0.0000 88.6060 0.0000 1.0800 1.6000 0.6600 166.7000

I 1.1046 0.0000 88.6060 0.0000 1.0800 1.6000 0.6600 166.7000

4 SLHVGTQCA 5.8420

S 0.2346 -0.6040 26.0680 15.9610 0.7700 0.7500 1.3200 89.0000

L 1.2906 0.0000 84.5480 0.0000 1.2100 1.3000 0.6800 166.7000

H 0.8124 -0.7766 82.1700 13.8630 1.0000 0.8700 1.0600 153.2000

V 0.5324 0.0000 77.8110 0.0000 1.0600 1.7000 0.6200 140.0000

G 0.0208 0.0000 3.7620 0.0000 0.5700 0.7500 1.5000 60.1000

T 1.4265 -0.4369 46.7290 16.0490 0.8300 1.1900 1.0700 116.1000

Q 1.0036 -0.7211 70.0880 17.8660 1.1100 1.1000 0.8600 143.9000

C 0.2479 -0.2402 23.5560 30.4540 0.7000 1.1900 1.1200 108.5000

A 0.1744 0.0000 34.7760 0.0000 1.4200 0.8300 0.7000 88.6000

5 ALPYWNFAT 5.8690

A 0.1744 0.0000 34.7760 0.0000 1.4200 0.8300 0.7000 88.6000

L 1.2906 0.0000 84.5480 0.0000 1.2100 1.3000 0.6800 166.7000

P 0.3226 0.0000 69.2300 0.0000 0.5700 0.5500 1.5900 122.7000

Y 0.4534 -0.5896 80.9650 42.7610 0.6900 1.4700 1.0600 193.6000

W 0.8364 -0.4310 133.6980 14.8820 1.0800 1.3700 0.7500 227.8000

N 0.6396 -0.7211 50.5080 17.7800 0.6700 0.8900 1.3500 117.7000

F 0.4412 -0.1195 105.7050 11.2470 1.1300 1.3800 0.7100 189.9000

A 0.1744 0.0000 34.7760 0.0000 1.4200 0.8300 0.7000 88.6000

T 1.4265 -0.4369 46.7290 16.0490 0.8300 1.1900 1.0700 116.1000

6 SLNFMGYVI 5.8810

S 0.2346 -0.6040 26.0680 15.9610 0.7700 0.7500 1.3200 89.0000

L 1.2906 0.0000 84.5480 0.0000 1.2100 1.3000 0.6800 166.7000

N 0.6396 -0.7211 50.5080 17.7800 0.6700 0.8900 1.3500 117.7000

F 0.4412 -0.1195 105.7050 11.2470 1.1300 1.3800 0.7100 189.9000

M 1.0768 -0.3068 70.3631 23.2300 1.4500 1.0500 0.5800 162.9000

G 0.0208 0.0000 3.7620 0.0000 0.5700 0.7500 1.5000 60.1000

Y 0.4534 -0.5896 80.9650 42.7610 0.6900 1.4700 1.0600 193.6000

V 0.5324 0.0000 77.8110 0.0000 1.0600 1.7000 0.6200 140.0000

I 1.1046 0.0000 88.6060 0.0000 1.0800 1.6000 0.6600 166.7000

7 NLQSLTNLL 6.0000

N 0.6396 -0.7211 50.5080 17.7800 0.6700 0.8900 1.3500 117.7000

L 1.2906 0.0000 84.5480 0.0000 1.2100 1.3000 0.6800 166.7000

Q 1.0036 -0.7211 70.0880 17.8660 1.1100 1.1000 0.8600 143.9000

S 0.2346 -0.6040 26.0680 15.9610 0.7700 0.7500 1.3200 89.0000

L 1.2906 0.0000 84.5480 0.0000 1.2100 1.3000 0.6800 166.7000

T 1.4265 -0.4369 46.7290 16.0490 0.8300 1.1900 1.0700 116.1000

N 0.6396 -0.7211 50.5080 17.7800 0.6700 0.8900 1.3500 117.7000

L 1.2906 0.0000 84.5480 0.0000 1.2100 1.3000 0.6800 166.7000

L 1.2906 0.0000 84.5480 0.0000 1.2100 1.3000 0.6800 166.7000

8 FVTWHRYHL 6.0250

F 0.4412 -0.1195 105.7050 11.2470 1.1300 1.3800 0.7100 189.9000

V 0.5324 0.0000 77.8110 0.0000 1.0600 1.7000 0.6200 140.0000

T 1.4265 -0.4369 46.7290 16.0490 0.8300 1.1900 1.0700 116.1000

W 0.8364 -0.4310 133.6980 14.8820 1.0800 1.3700 0.7500 227.8000

H 0.8124 -0.7766 82.1700 13.8630 1.0000 0.8700 1.0600 153.2000

R 1.2424 -1.4797 90.8010 35.3100 0.9800 0.9300 1.0400 173.4000

Y 0.4534 -0.5896 80.9650 42.7610 0.6900 1.4700 1.0600 193.6000

H 0.8124 -0.7766 82.1700 13.8630 1.0000 0.8700 1.0600 153.2000

L 1.2906 0.0000 84.5480 0.0000 1.2100 1.3000 0.6800 166.7000

9 DPKVKQWPL 6.1760

D 0.6058 -0.9298 37.4170 25.2733 1.0100 0.5400 1.2000 111.1000

P 0.3226 0.0000 69.2300 0.0000 0.5700 0.5500 1.5900 122.7000

K 1.4600 -0.6229 97.7140 8.0790 1.1600 0.7400 0.9800 168.7000

V 0.5324 0.0000 77.8110 0.0000 1.0600 1.7000 0.6200 140.0000

K 1.4600 -0.6229 97.7140 8.0790 1.1600 0.7400 0.9800 168.7000

Q 1.0036 -0.7211 70.0880 17.8660 1.1100 1.1000 0.8600 143.9000

W 0.8364 -0.4310 133.6980 14.8820 1.0800 1.3700 0.7500 227.8000

P 0.3226 0.0000 69.2300 0.0000 0.5700 0.5500 1.5900 122.7000

L 1.2906 0.0000 84.5480 0.0000 1.2100 1.3000 0.6800 166.7000

10 ITSQVPFSV 6.1960

I 1.1046 0.0000 88.6060 0.0000 1.0800 1.6000 0.6600 166.7000

T 1.4265 -0.4369 46.7290 16.0490 0.8300 1.1900 1.0700 116.1000

S 0.2346 -0.6040 26.0680 15.9610 0.7700 0.7500 1.3200 89.0000

Q 1.0036 -0.7211 70.0880 17.8660 1.1100 1.1000 0.8600 143.9000

V 0.5324 0.0000 77.8110 0.0000 1.0600 1.7000 0.6200 140.0000

P 0.3226 0.0000 69.2300 0.0000 0.5700 0.5500 1.5900 122.7000

F 0.4412 -0.1195 105.7050 11.2470 1.1300 1.3800 0.7100 189.9000

S 0.2346 -0.6040 26.0680 15.9610 0.7700 0.7500 1.3200 89.0000

V 0.5324 0.0000 77.8110 0.0000 1.0600 1.7000 0.6200 140.0000

11 ALAKAAAAI 6.2110

A 0.1744 0.0000 34.7760 0.0000 1.4200 0.8300 0.7000 88.6000

L 1.2906 0.0000 84.5480 0.0000 1.2100 1.3000 0.6800 166.7000

A 0.1744 0.0000 34.7760 0.0000 1.4200 0.8300 0.7000 88.6000

K 1.4600 -0.6229 97.7140 8.0790 1.1600 0.7400 0.9800 168.7000

A 0.1744 0.0000 34.7760 0.0000 1.4200 0.8300 0.7000 88.6000

A 0.1744 0.0000 34.7760 0.0000 1.4200 0.8300 0.7000 88.6000

A 0.1744 0.0000 34.7760 0.0000 1.4200 0.8300 0.7000 88.6000

A 0.1744 0.0000 34.7760 0.0000 1.4200 0.8300 0.7000 88.6000

I 1.1046 0.0000 88.6060 0.0000 1.0800 1.6000 0.6600 166.7000

12 GLGQVPLIV 6.3010

G 0.0208 0.0000 3.7620 0.0000 0.5700 0.7500 1.5000 60.1000

L 1.2906 0.0000 84.5480 0.0000 1.2100 1.3000 0.6800 166.7000

G 0.0208 0.0000 3.7620 0.0000 0.5700 0.7500 1.5000 60.1000

Q 1.0036 -0.7211 70.0880 17.8660 1.1100 1.1000 0.8600 143.9000

V 0.5324 0.0000 77.8110 0.0000 1.0600 1.7000 0.6200 140.0000

P 0.3226 0.0000 69.2300 0.0000 0.5700 0.5500 1.5900 122.7000

L 1.2906 0.0000 84.5480 0.0000 1.2100 1.3000 0.6800 166.7000

I 1.1046 0.0000 88.6060 0.0000 1.0800 1.6000 0.6600 166.7000

V 0.5324 0.0000 77.8110 0.0000 1.0600 1.7000 0.6200 140.0000

13 MLDLQPETT 6.3350

M 1.0768 -0.3068 70.3631 23.2300 1.4500 1.0500 0.5800 162.9000

L 1.2906 0.0000 84.5480 0.0000 1.2100 1.3000 0.6800 166.7000

D 0.6058 -0.9298 37.4170 25.2733 1.0100 0.5400 1.2000 111.1000

L 1.2906 0.0000 84.5480 0.0000 1.2100 1.3000 0.6800 166.7000

Q 1.0036 -0.7211 70.0880 17.8660 1.1100 1.1000 0.8600 143.9000

P 0.3226 0.0000 69.2300 0.0000 0.5700 0.5500 1.5900 122.7000

E 1.0315 -0.9298 57.1582 25.5730 1.5100 0.3700 0.8400 138.4000

T 1.4265 -0.4369 46.7290 16.0490 0.8300 1.1900 1.0700 116.1000

T 1.4265 -0.4369 46.7290 16.0490 0.8300 1.1900 1.0700 116.1000

14 LLSSNLSWL 6.3420

L 1.2906 0.0000 84.5480 0.0000 1.2100 1.3000 0.6800 166.7000

L 1.2906 0.0000 84.5480 0.0000 1.2100 1.3000 0.6800 166.7000

S 0.2346 -0.6040 26.0680 15.9610 0.7700 0.7500 1.3200 89.0000

S 0.2346 -0.6040 26.0680 15.9610 0.7700 0.7500 1.3200 89.0000

N 0.6396 -0.7211 50.5080 17.7800 0.6700 0.8900 1.3500 117.7000

L 1.2906 0.0000 84.5480 0.0000 1.2100 1.3000 0.6800 166.7000

S 0.2346 -0.6040 26.0680 15.9610 0.7700 0.7500 1.3200 89.0000

W 0.8364 -0.4310 133.6980 14.8820 1.0800 1.3700 0.7500 227.8000

L 1.2906 0.0000 84.5480 0.0000 1.2100 1.3000 0.6800 166.7000

15 GLACHQLCA 6.3800

G 0.0208 0.0000 3.7620 0.0000 0.5700 0.7500 1.5000 60.1000

L 1.2906 0.0000 84.5480 0.0000 1.2100 1.3000 0.6800 166.7000

A 0.1744 0.0000 34.7760 0.0000 1.4200 0.8300 0.7000 88.6000

C 0.2479 -0.2402 23.5560 30.4540 0.7000 1.1900 1.1200 108.5000

H 0.8124 -0.7766 82.1700 13.8630 1.0000 0.8700 1.0600 153.2000

Q 1.0036 -0.7211 70.0880 17.8660 1.1100 1.1000 0.8600 143.9000

L 1.2906 0.0000 84.5480 0.0000 1.2100 1.3000 0.6800 166.7000

C 0.2479 -0.2402 23.5560 30.4540 0.7000 1.1900 1.1200 108.5000

A 0.1744 0.0000 34.7760 0.0000 1.4200 0.8300 0.7000 88.6000

16 LIGNESFAL 6.4150

L 1.2906 0.0000 84.5480 0.0000 1.2100 1.3000 0.6800 166.7000

I 1.1046 0.0000 88.6060 0.0000 1.0800 1.6000 0.6600 166.7000

G 0.0208 0.0000 3.7620 0.0000 0.5700 0.7500 1.5000 60.1000

N 0.6396 -0.7211 50.5080 17.7800 0.6700 0.8900 1.3500 117.7000

E 1.0315 -0.9298 57.1582 25.5730 1.5100 0.3700 0.8400 138.4000

S 0.2346 -0.6040 26.0680 15.9610 0.7700 0.7500 1.3200 89.0000

F 0.4412 -0.1195 105.7050 11.2470 1.1300 1.3800 0.7100 189.9000

A 0.1744 0.0000 34.7760 0.0000 1.4200 0.8300 0.7000 88.6000

L 1.2906 0.0000 84.5480 0.0000 1.2100 1.3000 0.6800 166.7000

17 ALAKAAAAV 6.4190

A 0.1744 0.0000 34.7760 0.0000 1.4200 0.8300 0.7000 88.6000

L 1.2906 0.0000 84.5480 0.0000 1.2100 1.3000 0.6800 166.7000

A 0.1744 0.0000 34.7760 0.0000 1.4200 0.8300 0.7000 88.6000

K 1.4600 -0.6229 97.7140 8.0790 1.1600 0.7400 0.9800 168.7000

A 0.1744 0.0000 34.7760 0.0000 1.4200 0.8300 0.7000 88.6000

A 0.1744 0.0000 34.7760 0.0000 1.4200 0.8300 0.7000 88.6000

A 0.1744 0.0000 34.7760 0.0000 1.4200 0.8300 0.7000 88.6000

A 0.1744 0.0000 34.7760 0.0000 1.4200 0.8300 0.7000 88.6000

V 0.5324 0.0000 77.8110 0.0000 1.0600 1.7000 0.6200 140.0000

18 LLAVGATKV 6.4770

L 1.2906 0.0000 84.5480 0.0000 1.2100 1.3000 0.6800 166.7000

L 1.2906 0.0000 84.5480 0.0000 1.2100 1.3000 0.6800 166.7000

A 0.1744 0.0000 34.7760 0.0000 1.4200 0.8300 0.7000 88.6000

V 0.5324 0.0000 77.8110 0.0000 1.0600 1.7000 0.6200 140.0000

G 0.0208 0.0000 3.7620 0.0000 0.5700 0.7500 1.5000 60.1000

A 0.1744 0.0000 34.7760 0.0000 1.4200 0.8300 0.7000 88.6000

T 1.4265 -0.4369 46.7290 16.0490 0.8300 1.1900 1.0700 116.1000

K 1.4600 -0.6229 97.7140 8.0790 1.1600 0.7400 0.9800 168.7000

V 0.5324 0.0000 77.8110 0.0000 1.0600 1.7000 0.6200 140.0000

19 ALAKAAAAL 6.5110

A 0.1744 0.0000 34.7760 0.0000 1.4200 0.8300 0.7000 88.6000

L 1.2906 0.0000 84.5480 0.0000 1.2100 1.3000 0.6800 166.7000

A 0.1744 0.0000 34.7760 0.0000 1.4200 0.8300 0.7000 88.6000

K 1.4600 -0.6229 97.7140 8.0790 1.1600 0.7400 0.9800 168.7000

A 0.1744 0.0000 34.7760 0.0000 1.4200 0.8300 0.7000 88.6000

A 0.1744 0.0000 34.7760 0.0000 1.4200 0.8300 0.7000 88.6000

A 0.1744 0.0000 34.7760 0.0000 1.4200 0.8300 0.7000 88.6000

A 0.1744 0.0000 34.7760 0.0000 1.4200 0.8300 0.7000 88.6000

L 1.2906 0.0000 84.5480 0.0000 1.2100 1.3000 0.6800 166.7000

20 WILRGTSFV 6.5560

W 0.8364 -0.4310 133.6980 14.8820 1.0800 1.3700 0.7500 227.8000

I 1.1046 0.0000 88.6060 0.0000 1.0800 1.6000 0.6600 166.7000

L 1.2906 0.0000 84.5480 0.0000 1.2100 1.3000 0.6800 166.7000

R 1.2424 -1.4797 90.8010 35.3100 0.9800 0.9300 1.0400 173.4000

G 0.0208 0.0000 3.7620 0.0000 0.5700 0.7500 1.5000 60.1000

T 1.4265 -0.4369 46.7290 16.0490 0.8300 1.1900 1.0700 116.1000

S 0.2346 -0.6040 26.0680 15.9610 0.7700 0.7500 1.3200 89.0000

F 0.4412 -0.1195 105.7050 11.2470 1.1300 1.3800 0.7100 189.9000

V 0.5324 0.0000 77.8110 0.0000 1.0600 1.7000 0.6200 140.0000

21 IISCTCPTV 6.5800

I 1.1046 0.0000 88.6060 0.0000 1.0800 1.6000 0.6600 166.7000

I 1.1046 0.0000 88.6060 0.0000 1.0800 1.6000 0.6600 166.7000

S 0.2346 -0.6040 26.0680 15.9610 0.7700 0.7500 1.3200 89.0000

C 0.2479 -0.2402 23.5560 30.4540 0.7000 1.1900 1.1200 108.5000

T 1.4265 -0.4369 46.7290 16.0490 0.8300 1.1900 1.0700 116.1000

C 0.2479 -0.2402 23.5560 30.4540 0.7000 1.1900 1.1200 108.5000

P 0.3226 0.0000 69.2300 0.0000 0.5700 0.5500 1.5900 122.7000

T 1.4265 -0.4369 46.7290 16.0490 0.8300 1.1900 1.0700 116.1000

V 0.5324 0.0000 77.8110 0.0000 1.0600 1.7000 0.6200 140.0000

22 FLGGTPVCL 6.6230

F 0.4412 -0.1195 105.7050 11.2470 1.1300 1.3800 0.7100 189.9000

L 1.2906 0.0000 84.5480 0.0000 1.2100 1.3000 0.6800 166.7000

G 0.0208 0.0000 3.7620 0.0000 0.5700 0.7500 1.5000 60.1000

G 0.0208 0.0000 3.7620 0.0000 0.5700 0.7500 1.5000 60.1000

T 1.4265 -0.4369 46.7290 16.0490 0.8300 1.1900 1.0700 116.1000

P 0.3226 0.0000 69.2300 0.0000 0.5700 0.5500 1.5900 122.7000

V 0.5324 0.0000 77.8110 0.0000 1.0600 1.7000 0.6200 140.0000

C 0.2479 -0.2402 23.5560 30.4540 0.7000 1.1900 1.1200 108.5000

L 1.2906 0.0000 84.5480 0.0000 1.2100 1.3000 0.6800 166.7000

23 ALIHHNTHL 6.6230

A 0.1744 0.0000 34.7760 0.0000 1.4200 0.8300 0.7000 88.6000

L 1.2906 0.0000 84.5480 0.0000 1.2100 1.3000 0.6800 166.7000

I 1.1046 0.0000 88.6060 0.0000 1.0800 1.6000 0.6600 166.7000

H 0.8124 -0.7766 82.1700 13.8630 1.0000 0.8700 1.0600 153.2000

H 0.8124 -0.7766 82.1700 13.8630 1.0000 0.8700 1.0600 153.2000

N 0.6396 -0.7211 50.5080 17.7800 0.6700 0.8900 1.3500 117.7000

T 1.4265 -0.4369 46.7290 16.0490 0.8300 1.1900 1.0700 116.1000

H 0.8124 -0.7766 82.1700 13.8630 1.0000 0.8700 1.0600 153.2000

L 1.2906 0.0000 84.5480 0.0000 1.2100 1.3000 0.6800 166.7000

24 NLSWLSLDV 6.6390

N 0.6396 -0.7211 50.5080 17.7800 0.6700 0.8900 1.3500 117.7000

L 1.2906 0.0000 84.5480 0.0000 1.2100 1.3000 0.6800 166.7000

S 0.2346 -0.6040 26.0680 15.9610 0.7700 0.7500 1.3200 89.0000

W 0.8364 -0.4310 133.6980 14.8820 1.0800 1.3700 0.7500 227.8000

L 1.2906 0.0000 84.5480 0.0000 1.2100 1.3000 0.6800 166.7000

S 0.2346 -0.6040 26.0680 15.9610 0.7700 0.7500 1.3200 89.0000

L 1.2906 0.0000 84.5480 0.0000 1.2100 1.3000 0.6800 166.7000

D 0.6058 -0.9298 37.4170 25.2733 1.0100 0.5400 1.2000 111.1000

V 0.5324 0.0000 77.8110 0.0000 1.0600 1.7000 0.6200 140.0000

25 YMIMVKCWM 6.6630

Y 0.4534 -0.5896 80.9650 42.7610 0.6900 1.4700 1.0600 193.6000

M 1.0768 -0.3068 70.3631 23.2300 1.4500 1.0500 0.5800 162.9000

I 1.1046 0.0000 88.6060 0.0000 1.0800 1.6000 0.6600 166.7000

M 1.0768 -0.3068 70.3631 23.2300 1.4500 1.0500 0.5800 162.9000

V 0.5324 0.0000 77.8110 0.0000 1.0600 1.7000 0.6200 140.0000

K 1.4600 -0.6229 97.7140 8.0790 1.1600 0.7400 0.9800 168.7000

C 0.2479 -0.2402 23.5560 30.4540 0.7000 1.1900 1.1200 108.5000

W 0.8364 -0.4310 133.6980 14.8820 1.0800 1.3700 0.7500 227.8000

M 1.0768 -0.3068 70.3631 23.2300 1.4500 1.0500 0.5800 162.9000

26 VLQAGFFLL 6.6820

V 0.5324 0.0000 77.8110 0.0000 1.0600 1.7000 0.6200 140.0000

L 1.2906 0.0000 84.5480 0.0000 1.2100 1.3000 0.6800 166.7000

Q 1.0036 -0.7211 70.0880 17.8660 1.1100 1.1000 0.8600 143.9000

A 0.1744 0.0000 34.7760 0.0000 1.4200 0.8300 0.7000 88.6000

G 0.0208 0.0000 3.7620 0.0000 0.5700 0.7500 1.5000 60.1000

F 0.4412 -0.1195 105.7050 11.2470 1.1300 1.3800 0.7100 189.9000

F 0.4412 -0.1195 105.7050 11.2470 1.1300 1.3800 0.7100 189.9000

L 1.2906 0.0000 84.5480 0.0000 1.2100 1.3000 0.6800 166.7000

L 1.2906 0.0000 84.5480 0.0000 1.2100 1.3000 0.6800 166.7000

27 GTLGIVCPI 6.7140

G 0.0208 0.0000 3.7620 0.0000 0.5700 0.7500 1.5000 60.1000

T 1.4265 -0.4369 46.7290 16.0490 0.8300 1.1900 1.0700 116.1000

L 1.2906 0.0000 84.5480 0.0000 1.2100 1.3000 0.6800 166.7000

G 0.0208 0.0000 3.7620 0.0000 0.5700 0.7500 1.5000 60.1000

I 1.1046 0.0000 88.6060 0.0000 1.0800 1.6000 0.6600 166.7000

V 0.5324 0.0000 77.8110 0.0000 1.0600 1.7000 0.6200 140.0000

C 0.2479 -0.2402 23.5560 30.4540 0.7000 1.1900 1.1200 108.5000

P 0.3226 0.0000 69.2300 0.0000 0.5700 0.5500 1.5900 122.7000

I 1.1046 0.0000 88.6060 0.0000 1.0800 1.6000 0.6600 166.7000

28 VILGVLLLI 6.7850

V 0.5324 0.0000 77.8110 0.0000 1.0600 1.7000 0.6200 140.0000

I 1.1046 0.0000 88.6060 0.0000 1.0800 1.6000 0.6600 166.7000

L 1.2906 0.0000 84.5480 0.0000 1.2100 1.3000 0.6800 166.7000

G 0.0208 0.0000 3.7620 0.0000 0.5700 0.7500 1.5000 60.1000

V 0.5324 0.0000 77.8110 0.0000 1.0600 1.7000 0.6200 140.0000

L 1.2906 0.0000 84.5480 0.0000 1.2100 1.3000 0.6800 166.7000

L 1.2906 0.0000 84.5480 0.0000 1.2100 1.3000 0.6800 166.7000

L 1.2906 0.0000 84.5480 0.0000 1.2100 1.3000 0.6800 166.7000

I 1.1046 0.0000 88.6060 0.0000 1.0800 1.6000 0.6600 166.7000

29 VTWHRYHLL 6.7930

V 0.5324 0.0000 77.8110 0.0000 1.0600 1.7000 0.6200 140.0000

T 1.4265 -0.4369 46.7290 16.0490 0.8300 1.1900 1.0700 116.1000

W 0.8364 -0.4310 133.6980 14.8820 1.0800 1.3700 0.7500 227.8000

H 0.8124 -0.7766 82.1700 13.8630 1.0000 0.8700 1.0600 153.2000

R 1.2424 -1.4797 90.8010 35.3100 0.9800 0.9300 1.0400 173.4000

Y 0.4534 -0.5896 80.9650 42.7610 0.6900 1.4700 1.0600 193.6000

H 0.8124 -0.7766 82.1700 13.8630 1.0000 0.8700 1.0600 153.2000

L 1.2906 0.0000 84.5480 0.0000 1.2100 1.3000 0.6800 166.7000

L 1.2906 0.0000 84.5480 0.0000 1.2100 1.3000 0.6800 166.7000

30 PLLPIFFCL 6.7960

P 0.3226 0.0000 69.2300 0.0000 0.5700 0.5500 1.5900 122.7000

L 1.2906 0.0000 84.5480 0.0000 1.2100 1.3000 0.6800 166.7000

L 1.2906 0.0000 84.5480 0.0000 1.2100 1.3000 0.6800 166.7000

P 0.3226 0.0000 69.2300 0.0000 0.5700 0.5500 1.5900 122.7000

I 1.1046 0.0000 88.6060 0.0000 1.0800 1.6000 0.6600 166.7000

F 0.4412 -0.1195 105.7050 11.2470 1.1300 1.3800 0.7100 189.9000

F 0.4412 -0.1195 105.7050 11.2470 1.1300 1.3800 0.7100 189.9000

C 0.2479 -0.2402 23.5560 30.4540 0.7000 1.1900 1.1200 108.5000

L 1.2906 0.0000 84.5480 0.0000 1.2100 1.3000 0.6800 166.7000

31 TLGIVCPIC 6.8150

T 1.4265 -0.4369 46.7290 16.0490 0.8300 1.1900 1.0700 116.1000

L 1.2906 0.0000 84.5480 0.0000 1.2100 1.3000 0.6800 166.7000

G 0.0208 0.0000 3.7620 0.0000 0.5700 0.7500 1.5000 60.1000

I 1.1046 0.0000 88.6060 0.0000 1.0800 1.6000 0.6600 166.7000

V 0.5324 0.0000 77.8110 0.0000 1.0600 1.7000 0.6200 140.0000

C 0.2479 -0.2402 23.5560 30.4540 0.7000 1.1900 1.1200 108.5000

P 0.3226 0.0000 69.2300 0.0000 0.5700 0.5500 1.5900 122.7000

I 1.1046 0.0000 88.6060 0.0000 1.0800 1.6000 0.6600 166.7000

C 0.2479 -0.2402 23.5560 30.4540 0.7000 1.1900 1.1200 108.5000

32 CLTSTVQLV 6.8320

C 0.2479 -0.2402 23.5560 30.4540 0.7000 1.1900 1.1200 108.5000

L 1.2906 0.0000 84.5480 0.0000 1.2100 1.3000 0.6800 166.7000

T 1.4265 -0.4369 46.7290 16.0490 0.8300 1.1900 1.0700 116.1000

S 0.2346 -0.6040 26.0680 15.9610 0.7700 0.7500 1.3200 89.0000

T 1.4265 -0.4369 46.7290 16.0490 0.8300 1.1900 1.0700 116.1000

V 0.5324 0.0000 77.8110 0.0000 1.0600 1.7000 0.6200 140.0000

Q 1.0036 -0.7211 70.0880 17.8660 1.1100 1.1000 0.8600 143.9000

L 1.2906 0.0000 84.5480 0.0000 1.2100 1.3000 0.6800 166.7000

V 0.5324 0.0000 77.8110 0.0000 1.0600 1.7000 0.6200 140.0000

33 ILLLCLIFL 6.8450

I 1.1046 0.0000 88.6060 0.0000 1.0800 1.6000 0.6600 166.7000

L 1.2906 0.0000 84.5480 0.0000 1.2100 1.3000 0.6800 166.7000

L 1.2906 0.0000 84.5480 0.0000 1.2100 1.3000 0.6800 166.7000

L 1.2906 0.0000 84.5480 0.0000 1.2100 1.3000 0.6800 166.7000

C 0.2479 -0.2402 23.5560 30.4540 0.7000 1.1900 1.1200 108.5000

L 1.2906 0.0000 84.5480 0.0000 1.2100 1.3000 0.6800 166.7000

I 1.1046 0.0000 88.6060 0.0000 1.0800 1.6000 0.6600 166.7000

F 0.4412 -0.1195 105.7050 11.2470 1.1300 1.3800 0.7100 189.9000

L 1.2906 0.0000 84.5480 0.0000 1.2100 1.3000 0.6800 166.7000

34 FAFRDLCIV 6.8860

F 0.4412 -0.1195 105.7050 11.2470 1.1300 1.3800 0.7100 189.9000

A 0.1744 0.0000 34.7760 0.0000 1.4200 0.8300 0.7000 88.6000

F 0.4412 -0.1195 105.7050 11.2470 1.1300 1.3800 0.7100 189.9000

R 1.2424 -1.4797 90.8010 35.3100 0.9800 0.9300 1.0400 173.4000

D 0.6058 -0.9298 37.4170 25.2733 1.0100 0.5400 1.2000 111.1000

L 1.2906 0.0000 84.5480 0.0000 1.2100 1.3000 0.6800 166.7000

C 0.2479 -0.2402 23.5560 30.4540 0.7000 1.1900 1.1200 108.5000

I 1.1046 0.0000 88.6060 0.0000 1.0800 1.6000 0.6600 166.7000

V 0.5324 0.0000 77.8110 0.0000 1.0600 1.7000 0.6200 140.0000

35 FLEPGPVTA 6.8980

F 0.4412 -0.1195 105.7050 11.2470 1.1300 1.3800 0.7100 189.9000

L 1.2906 0.0000 84.5480 0.0000 1.2100 1.3000 0.6800 166.7000

E 1.0315 -0.9298 57.1582 25.5730 1.5100 0.3700 0.8400 138.4000

P 0.3226 0.0000 69.2300 0.0000 0.5700 0.5500 1.5900 122.7000

G 0.0208 0.0000 3.7620 0.0000 0.5700 0.7500 1.5000 60.1000

P 0.3226 0.0000 69.2300 0.0000 0.5700 0.5500 1.5900 122.7000

V 0.5324 0.0000 77.8110 0.0000 1.0600 1.7000 0.6200 140.0000

T 1.4265 -0.4369 46.7290 16.0490 0.8300 1.1900 1.0700 116.1000

A 0.1744 0.0000 34.7760 0.0000 1.4200 0.8300 0.7000 88.6000

36 ALAKAAAAA 6.9470

A 0.1744 0.0000 34.7760 0.0000 1.4200 0.8300 0.7000 88.6000

L 1.2906 0.0000 84.5480 0.0000 1.2100 1.3000 0.6800 166.7000

A 0.1744 0.0000 34.7760 0.0000 1.4200 0.8300 0.7000 88.6000

K 1.4600 -0.6229 97.7140 8.0790 1.1600 0.7400 0.9800 168.7000

A 0.1744 0.0000 34.7760 0.0000 1.4200 0.8300 0.7000 88.6000

A 0.1744 0.0000 34.7760 0.0000 1.4200 0.8300 0.7000 88.6000

A 0.1744 0.0000 34.7760 0.0000 1.4200 0.8300 0.7000 88.6000

A 0.1744 0.0000 34.7760 0.0000 1.4200 0.8300 0.7000 88.6000

A 0.1744 0.0000 34.7760 0.0000 1.4200 0.8300 0.7000 88.6000

37 LMAVVLASL 6.9540

L 1.2906 0.0000 84.5480 0.0000 1.2100 1.3000 0.6800 166.7000

M 1.0768 -0.3068 70.3631 23.2300 1.4500 1.0500 0.5800 162.9000

A 0.1744 0.0000 34.7760 0.0000 1.4200 0.8300 0.7000 88.6000

V 0.5324 0.0000 77.8110 0.0000 1.0600 1.7000 0.6200 140.0000

V 0.5324 0.0000 77.8110 0.0000 1.0600 1.7000 0.6200 140.0000

L 1.2906 0.0000 84.5480 0.0000 1.2100 1.3000 0.6800 166.7000

A 0.1744 0.0000 34.7760 0.0000 1.4200 0.8300 0.7000 88.6000

S 0.2346 -0.6040 26.0680 15.9610 0.7700 0.7500 1.3200 89.0000

L 1.2906 0.0000 84.5480 0.0000 1.2100 1.3000 0.6800 166.7000

38 YVITTQHWL 6.9830

Y 0.4534 -0.5896 80.9650 42.7610 0.6900 1.4700 1.0600 193.6000

V 0.5324 0.0000 77.8110 0.0000 1.0600 1.7000 0.6200 140.0000

I 1.1046 0.0000 88.6060 0.0000 1.0800 1.6000 0.6600 166.7000

T 1.4265 -0.4369 46.7290 16.0490 0.8300 1.1900 1.0700 116.1000

T 1.4265 -0.4369 46.7290 16.0490 0.8300 1.1900 1.0700 116.1000

Q 1.0036 -0.7211 70.0880 17.8660 1.1100 1.1000 0.8600 143.9000

H 0.8124 -0.7766 82.1700 13.8630 1.0000 0.8700 1.0600 153.2000

W 0.8364 -0.4310 133.6980 14.8820 1.0800 1.3700 0.7500 227.8000

L 1.2906 0.0000 84.5480 0.0000 1.2100 1.3000 0.6800 166.7000

39 LLCLIFLLV 6.9960

L 1.2906 0.0000 84.5480 0.0000 1.2100 1.3000 0.6800 166.7000

L 1.2906 0.0000 84.5480 0.0000 1.2100 1.3000 0.6800 166.7000

C 0.2479 -0.2402 23.5560 30.4540 0.7000 1.1900 1.1200 108.5000

L 1.2906 0.0000 84.5480 0.0000 1.2100 1.3000 0.6800 166.7000

I 1.1046 0.0000 88.6060 0.0000 1.0800 1.6000 0.6600 166.7000

F 0.4412 -0.1195 105.7050 11.2470 1.1300 1.3800 0.7100 189.9000

L 1.2906 0.0000 84.5480 0.0000 1.2100 1.3000 0.6800 166.7000

L 1.2906 0.0000 84.5480 0.0000 1.2100 1.3000 0.6800 166.7000

V 0.5324 0.0000 77.8110 0.0000 1.0600 1.7000 0.6200 140.0000

40 ITAQVPFSV 7.0200

I 1.1046 0.0000 88.6060 0.0000 1.0800 1.6000 0.6600 166.7000

T 1.4265 -0.4369 46.7290 16.0490 0.8300 1.1900 1.0700 116.1000

A 0.1744 0.0000 34.7760 0.0000 1.4200 0.8300 0.7000 88.6000

Q 1.0036 -0.7211 70.0880 17.8660 1.1100 1.1000 0.8600 143.9000

V 0.5324 0.0000 77.8110 0.0000 1.0600 1.7000 0.6200 140.0000

P 0.3226 0.0000 69.2300 0.0000 0.5700 0.5500 1.5900 122.7000

F 0.4412 -0.1195 105.7050 11.2470 1.1300 1.3800 0.7100 189.9000

S 0.2346 -0.6040 26.0680 15.9610 0.7700 0.7500 1.3200 89.0000

V 0.5324 0.0000 77.8110 0.0000 1.0600 1.7000 0.6200 140.0000

41 YLEPGPVTL 7.0580

Y 0.4534 -0.5896 80.9650 42.7610 0.6900 1.4700 1.0600 193.6000

L 1.2906 0.0000 84.5480 0.0000 1.2100 1.3000 0.6800 166.7000

E 1.0315 -0.9298 57.1582 25.5730 1.5100 0.3700 0.8400 138.4000

P 0.3226 0.0000 69.2300 0.0000 0.5700 0.5500 1.5900 122.7000

G 0.0208 0.0000 3.7620 0.0000 0.5700 0.7500 1.5000 60.1000

P 0.3226 0.0000 69.2300 0.0000 0.5700 0.5500 1.5900 122.7000

V 0.5324 0.0000 77.8110 0.0000 1.0600 1.7000 0.6200 140.0000

T 1.4265 -0.4369 46.7290 16.0490 0.8300 1.1900 1.0700 116.1000

L 1.2906 0.0000 84.5480 0.0000 1.2100 1.3000 0.6800 166.7000

42 YTDQVPFSV 7.0660

Y 0.4534 -0.5896 80.9650 42.7610 0.6900 1.4700 1.0600 193.6000

T 1.4265 -0.4369 46.7290 16.0490 0.8300 1.1900 1.0700 116.1000

D 0.6058 -0.9298 37.4170 25.2733 1.0100 0.5400 1.2000 111.1000

Q 1.0036 -0.7211 70.0880 17.8660 1.1100 1.1000 0.8600 143.9000

V 0.5324 0.0000 77.8110 0.0000 1.0600 1.7000 0.6200 140.0000

P 0.3226 0.0000 69.2300 0.0000 0.5700 0.5500 1.5900 122.7000

F 0.4412 -0.1195 105.7050 11.2470 1.1300 1.3800 0.7100 189.9000

S 0.2346 -0.6040 26.0680 15.9610 0.7700 0.7500 1.3200 89.0000

V 0.5324 0.0000 77.8110 0.0000 1.0600 1.7000 0.6200 140.0000

43 NLYVSLLLL 7.1140

N 0.6396 -0.7211 50.5080 17.7800 0.6700 0.8900 1.3500 117.7000

L 1.2906 0.0000 84.5480 0.0000 1.2100 1.3000 0.6800 166.7000

Y 0.4534 -0.5896 80.9650 42.7610 0.6900 1.4700 1.0600 193.6000

V 0.5324 0.0000 77.8110 0.0000 1.0600 1.7000 0.6200 140.0000

S 0.2346 -0.6040 26.0680 15.9610 0.7700 0.7500 1.3200 89.0000

L 1.2906 0.0000 84.5480 0.0000 1.2100 1.3000 0.6800 166.7000

L 1.2906 0.0000 84.5480 0.0000 1.2100 1.3000 0.6800 166.7000

L 1.2906 0.0000 84.5480 0.0000 1.2100 1.3000 0.6800 166.7000

L 1.2906 0.0000 84.5480 0.0000 1.2100 1.3000 0.6800 166.7000

44 ILHNGAYSL 7.1270

I 1.1046 0.0000 88.6060 0.0000 1.0800 1.6000 0.6600 166.7000

L 1.2906 0.0000 84.5480 0.0000 1.2100 1.3000 0.6800 166.7000

H 0.8124 -0.7766 82.1700 13.8630 1.0000 0.8700 1.0600 153.2000

N 0.6396 -0.7211 50.5080 17.7800 0.6700 0.8900 1.3500 117.7000

G 0.0208 0.0000 3.7620 0.0000 0.5700 0.7500 1.5000 60.1000

A 0.1744 0.0000 34.7760 0.0000 1.4200 0.8300 0.7000 88.6000

Y 0.4534 -0.5896 80.9650 42.7610 0.6900 1.4700 1.0600 193.6000

S 0.2346 -0.6040 26.0680 15.9610 0.7700 0.7500 1.3200 89.0000

L 1.2906 0.0000 84.5480 0.0000 1.2100 1.3000 0.6800 166.7000

45 SIISAVVGI 7.1590

S 0.2346 -0.6040 26.0680 15.9610 0.7700 0.7500 1.3200 89.0000

I 1.1046 0.0000 88.6060 0.0000 1.0800 1.6000 0.6600 166.7000

I 1.1046 0.0000 88.6060 0.0000 1.0800 1.6000 0.6600 166.7000

S 0.2346 -0.6040 26.0680 15.9610 0.7700 0.7500 1.3200 89.0000

A 0.1744 0.0000 34.7760 0.0000 1.4200 0.8300 0.7000 88.6000

V 0.5324 0.0000 77.8110 0.0000 1.0600 1.7000 0.6200 140.0000

V 0.5324 0.0000 77.8110 0.0000 1.0600 1.7000 0.6200 140.0000

G 0.0208 0.0000 3.7620 0.0000 0.5700 0.7500 1.5000 60.1000

I 1.1046 0.0000 88.6060 0.0000 1.0800 1.6000 0.6600 166.7000

46 VVMGTLVAL 7.1740

V 0.5324 0.0000 77.8110 0.0000 1.0600 1.7000 0.6200 140.0000

V 0.5324 0.0000 77.8110 0.0000 1.0600 1.7000 0.6200 140.0000

M 1.0768 -0.3068 70.3631 23.2300 1.4500 1.0500 0.5800 162.9000

G 0.0208 0.0000 3.7620 0.0000 0.5700 0.7500 1.5000 60.1000

T 1.4265 -0.4369 46.7290 16.0490 0.8300 1.1900 1.0700 116.1000

L 1.2906 0.0000 84.5480 0.0000 1.2100 1.3000 0.6800 166.7000

V 0.5324 0.0000 77.8110 0.0000 1.0600 1.7000 0.6200 140.0000

A 0.1744 0.0000 34.7760 0.0000 1.4200 0.8300 0.7000 88.6000

L 1.2906 0.0000 84.5480 0.0000 1.2100 1.3000 0.6800 166.7000

47 YLEPGPVTI 7.1870

Y 0.4534 -0.5896 80.9650 42.7610 0.6900 1.4700 1.0600 193.6000

L 1.2906 0.0000 84.5480 0.0000 1.2100 1.3000 0.6800 166.7000

E 1.0315 -0.9298 57.1582 25.5730 1.5100 0.3700 0.8400 138.4000

P 0.3226 0.0000 69.2300 0.0000 0.5700 0.5500 1.5900 122.7000

G 0.0208 0.0000 3.7620 0.0000 0.5700 0.7500 1.5000 60.1000

P 0.3226 0.0000 69.2300 0.0000 0.5700 0.5500 1.5900 122.7000

V 0.5324 0.0000 77.8110 0.0000 1.0600 1.7000 0.6200 140.0000

T 1.4265 -0.4369 46.7290 16.0490 0.8300 1.1900 1.0700 116.1000

I 1.1046 0.0000 88.6060 0.0000 1.0800 1.6000 0.6600 166.7000

48 GLSRYVARL 7.2480

G 0.0208 0.0000 3.7620 0.0000 0.5700 0.7500 1.5000 60.1000

L 1.2906 0.0000 84.5480 0.0000 1.2100 1.3000 0.6800 166.7000

S 0.2346 -0.6040 26.0680 15.9610 0.7700 0.7500 1.3200 89.0000

R 1.2424 -1.4797 90.8010 35.3100 0.9800 0.9300 1.0400 173.4000

Y 0.4534 -0.5896 80.9650 42.7610 0.6900 1.4700 1.0600 193.6000

V 0.5324 0.0000 77.8110 0.0000 1.0600 1.7000 0.6200 140.0000

A 0.1744 0.0000 34.7760 0.0000 1.4200 0.8300 0.7000 88.6000

R 1.2424 -1.4797 90.8010 35.3100 0.9800 0.9300 1.0400 173.4000

L 1.2906 0.0000 84.5480 0.0000 1.2100 1.3000 0.6800 166.7000

49 LLAQFTSAI 7.3010

L 1.2906 0.0000 84.5480 0.0000 1.2100 1.3000 0.6800 166.7000

L 1.2906 0.0000 84.5480 0.0000 1.2100 1.3000 0.6800 166.7000

A 0.1744 0.0000 34.7760 0.0000 1.4200 0.8300 0.7000 88.6000

Q 1.0036 -0.7211 70.0880 17.8660 1.1100 1.1000 0.8600 143.9000

F 0.4412 -0.1195 105.7050 11.2470 1.1300 1.3800 0.7100 189.9000

T 1.4265 -0.4369 46.7290 16.0490 0.8300 1.1900 1.0700 116.1000

S 0.2346 -0.6040 26.0680 15.9610 0.7700 0.7500 1.3200 89.0000

A 0.1744 0.0000 34.7760 0.0000 1.4200 0.8300 0.7000 88.6000

I 1.1046 0.0000 88.6060 0.0000 1.0800 1.6000 0.6600 166.7000

50 VLLDYQGML 7.3280

V 0.5324 0.0000 77.8110 0.0000 1.0600 1.7000 0.6200 140.0000

L 1.2906 0.0000 84.5480 0.0000 1.2100 1.3000 0.6800 166.7000

L 1.2906 0.0000 84.5480 0.0000 1.2100 1.3000 0.6800 166.7000

D 0.6058 -0.9298 37.4170 25.2733 1.0100 0.5400 1.2000 111.1000

Y 0.4534 -0.5896 80.9650 42.7610 0.6900 1.4700 1.0600 193.6000

Q 1.0036 -0.7211 70.0880 17.8660 1.1100 1.1000 0.8600 143.9000

G 0.0208 0.0000 3.7620 0.0000 0.5700 0.7500 1.5000 60.1000

M 1.0768 -0.3068 70.3631 23.2300 1.4500 1.0500 0.5800 162.9000

L 1.2906 0.0000 84.5480 0.0000 1.2100 1.3000 0.6800 166.7000

51 YLEPGPVTV 7.3420

Y 0.4534 -0.5896 80.9650 42.7610 0.6900 1.4700 1.0600 193.6000

L 1.2906 0.0000 84.5480 0.0000 1.2100 1.3000 0.6800 166.7000

E 1.0315 -0.9298 57.1582 25.5730 1.5100 0.3700 0.8400 138.4000

P 0.3226 0.0000 69.2300 0.0000 0.5700 0.5500 1.5900 122.7000

G 0.0208 0.0000 3.7620 0.0000 0.5700 0.7500 1.5000 60.1000

P 0.3226 0.0000 69.2300 0.0000 0.5700 0.5500 1.5900 122.7000

V 0.5324 0.0000 77.8110 0.0000 1.0600 1.7000 0.6200 140.0000

T 1.4265 -0.4369 46.7290 16.0490 0.8300 1.1900 1.0700 116.1000

V 0.5324 0.0000 77.8110 0.0000 1.0600 1.7000 0.6200 140.0000

52 ILSPFMPLL 7.3470

I 1.1046 0.0000 88.6060 0.0000 1.0800 1.6000 0.6600 166.7000

L 1.2906 0.0000 84.5480 0.0000 1.2100 1.3000 0.6800 166.7000

S 0.2346 -0.6040 26.0680 15.9610 0.7700 0.7500 1.3200 89.0000

P 0.3226 0.0000 69.2300 0.0000 0.5700 0.5500 1.5900 122.7000

F 0.4412 -0.1195 105.7050 11.2470 1.1300 1.3800 0.7100 189.9000

M 1.0768 -0.3068 70.3631 23.2300 1.4500 1.0500 0.5800 162.9000

P 0.3226 0.0000 69.2300 0.0000 0.5700 0.5500 1.5900 122.7000

L 1.2906 0.0000 84.5480 0.0000 1.2100 1.3000 0.6800 166.7000

L 1.2906 0.0000 84.5480 0.0000 1.2100 1.3000 0.6800 166.7000

53 YLSPGPVTA 7.3830

Y 0.4534 -0.5896 80.9650 42.7610 0.6900 1.4700 1.0600 193.6000

L 1.2906 0.0000 84.5480 0.0000 1.2100 1.3000 0.6800 166.7000

S 0.2346 -0.6040 26.0680 15.9610 0.7700 0.7500 1.3200 89.0000

P 0.3226 0.0000 69.2300 0.0000 0.5700 0.5500 1.5900 122.7000

G 0.0208 0.0000 3.7620 0.0000 0.5700 0.7500 1.5000 60.1000

P 0.3226 0.0000 69.2300 0.0000 0.5700 0.5500 1.5900 122.7000

V 0.5324 0.0000 77.8110 0.0000 1.0600 1.7000 0.6200 140.0000

T 1.4265 -0.4369 46.7290 16.0490 0.8300 1.1900 1.0700 116.1000

A 0.1744 0.0000 34.7760 0.0000 1.4200 0.8300 0.7000 88.6000

54 IIDQVPFSV 7.3980

I 1.1046 0.0000 88.6060 0.0000 1.0800 1.6000 0.6600 166.7000

I 1.1046 0.0000 88.6060 0.0000 1.0800 1.6000 0.6600 166.7000

D 0.6058 -0.9298 37.4170 25.2733 1.0100 0.5400 1.2000 111.1000

Q 1.0036 -0.7211 70.0880 17.8660 1.1100 1.1000 0.8600 143.9000

V 0.5324 0.0000 77.8110 0.0000 1.0600 1.7000 0.6200 140.0000

P 0.3226 0.0000 69.2300 0.0000 0.5700 0.5500 1.5900 122.7000

F 0.4412 -0.1195 105.7050 11.2470 1.1300 1.3800 0.7100 189.9000

S 0.2346 -0.6040 26.0680 15.9610 0.7700 0.7500 1.3200 89.0000

V 0.5324 0.0000 77.8110 0.0000 1.0600 1.7000 0.6200 140.0000

55 SVYDFFVWL 7.4440

S 0.2346 -0.6040 26.0680 15.9610 0.7700 0.7500 1.3200 89.0000

V 0.5324 0.0000 77.8110 0.0000 1.0600 1.7000 0.6200 140.0000

Y 0.4534 -0.5896 80.9650 42.7610 0.6900 1.4700 1.0600 193.6000

D 0.6058 -0.9298 37.4170 25.2733 1.0100 0.5400 1.2000 111.1000

F 0.4412 -0.1195 105.7050 11.2470 1.1300 1.3800 0.7100 189.9000

F 0.4412 -0.1195 105.7050 11.2470 1.1300 1.3800 0.7100 189.9000

V 0.5324 0.0000 77.8110 0.0000 1.0600 1.7000 0.6200 140.0000

W 0.8364 -0.4310 133.6980 14.8820 1.0800 1.3700 0.7500 227.8000

L 1.2906 0.0000 84.5480 0.0000 1.2100 1.3000 0.6800 166.7000

56 ITWQVPFSV 7.4630

I 1.1046 0.0000 88.6060 0.0000 1.0800 1.6000 0.6600 166.7000

T 1.4265 -0.4369 46.7290 16.0490 0.8300 1.1900 1.0700 116.1000

W 0.8364 -0.4310 133.6980 14.8820 1.0800 1.3700 0.7500 227.8000

Q 1.0036 -0.7211 70.0880 17.8660 1.1100 1.1000 0.8600 143.9000

V 0.5324 0.0000 77.8110 0.0000 1.0600 1.7000 0.6200 140.0000

P 0.3226 0.0000 69.2300 0.0000 0.5700 0.5500 1.5900 122.7000

F 0.4412 -0.1195 105.7050 11.2470 1.1300 1.3800 0.7100 189.9000

S 0.2346 -0.6040 26.0680 15.9610 0.7700 0.7500 1.3200 89.0000

V 0.5324 0.0000 77.8110 0.0000 1.0600 1.7000 0.6200 140.0000

57 ITYQVPFSV 7.4800

I 1.1046 0.0000 88.6060 0.0000 1.0800 1.6000 0.6600 166.7000

T 1.4265 -0.4369 46.7290 16.0490 0.8300 1.1900 1.0700 116.1000

Y 0.4534 -0.5896 80.9650 42.7610 0.6900 1.4700 1.0600 193.6000

Q 1.0036 -0.7211 70.0880 17.8660 1.1100 1.1000 0.8600 143.9000

V 0.5324 0.0000 77.8110 0.0000 1.0600 1.7000 0.6200 140.0000

P 0.3226 0.0000 69.2300 0.0000 0.5700 0.5500 1.5900 122.7000

F 0.4412 -0.1195 105.7050 11.2470 1.1300 1.3800 0.7100 189.9000

S 0.2346 -0.6040 26.0680 15.9610 0.7700 0.7500 1.3200 89.0000

V 0.5324 0.0000 77.8110 0.0000 1.0600 1.7000 0.6200 140.0000

58 GLYSSTVPV 7.4810

G 0.0208 0.0000 3.7620 0.0000 0.5700 0.7500 1.5000 60.1000

L 1.2906 0.0000 84.5480 0.0000 1.2100 1.3000 0.6800 166.7000

Y 0.4534 -0.5896 80.9650 42.7610 0.6900 1.4700 1.0600 193.6000

S 0.2346 -0.6040 26.0680 15.9610 0.7700 0.7500 1.3200 89.0000

S 0.2346 -0.6040 26.0680 15.9610 0.7700 0.7500 1.3200 89.0000

T 1.4265 -0.4369 46.7290 16.0490 0.8300 1.1900 1.0700 116.1000

V 0.5324 0.0000 77.8110 0.0000 1.0600 1.7000 0.6200 140.0000

P 0.3226 0.0000 69.2300 0.0000 0.5700 0.5500 1.5900 122.7000

V 0.5324 0.0000 77.8110 0.0000 1.0600 1.7000 0.6200 140.0000

59 VMGTLVALV 7.5530

V 0.5324 0.0000 77.8110 0.0000 1.0600 1.7000 0.6200 140.0000

M 1.0768 -0.3068 70.3631 23.2300 1.4500 1.0500 0.5800 162.9000

G 0.0208 0.0000 3.7620 0.0000 0.5700 0.7500 1.5000 60.1000

T 1.4265 -0.4369 46.7290 16.0490 0.8300 1.1900 1.0700 116.1000

L 1.2906 0.0000 84.5480 0.0000 1.2100 1.3000 0.6800 166.7000

V 0.5324 0.0000 77.8110 0.0000 1.0600 1.7000 0.6200 140.0000

A 0.1744 0.0000 34.7760 0.0000 1.4200 0.8300 0.7000 88.6000

L 1.2906 0.0000 84.5480 0.0000 1.2100 1.3000 0.6800 166.7000

V 0.5324 0.0000 77.8110 0.0000 1.0600 1.7000 0.6200 140.0000

60 LLLCLIFLL 7.5850

L 1.2906 0.0000 84.5480 0.0000 1.2100 1.3000 0.6800 166.7000

L 1.2906 0.0000 84.5480 0.0000 1.2100 1.3000 0.6800 166.7000

L 1.2906 0.0000 84.5480 0.0000 1.2100 1.3000 0.6800 166.7000

C 0.2479 -0.2402 23.5560 30.4540 0.7000 1.1900 1.1200 108.5000

L 1.2906 0.0000 84.5480 0.0000 1.2100 1.3000 0.6800 166.7000

I 1.1046 0.0000 88.6060 0.0000 1.0800 1.6000 0.6600 166.7000

F 0.4412 -0.1195 105.7050 11.2470 1.1300 1.3800 0.7100 189.9000

L 1.2906 0.0000 84.5480 0.0000 1.2100 1.3000 0.6800 166.7000

L 1.2906 0.0000 84.5480 0.0000 1.2100 1.3000 0.6800 166.7000

61 SLDDYNHLV 7.5850

S 0.2346 -0.6040 26.0680 15.9610 0.7700 0.7500 1.3200 89.0000

L 1.2906 0.0000 84.5480 0.0000 1.2100 1.3000 0.6800 166.7000

D 0.6058 -0.9298 37.4170 25.2733 1.0100 0.5400 1.2000 111.1000

D 0.6058 -0.9298 37.4170 25.2733 1.0100 0.5400 1.2000 111.1000

Y 0.4534 -0.5896 80.9650 42.7610 0.6900 1.4700 1.0600 193.6000

N 0.6396 -0.7211 50.5080 17.7800 0.6700 0.8900 1.3500 117.7000

H 0.8124 -0.7766 82.1700 13.8630 1.0000 0.8700 1.0600 153.2000

L 1.2906 0.0000 84.5480 0.0000 1.2100 1.3000 0.6800 166.7000

V 0.5324 0.0000 77.8110 0.0000 1.0600 1.7000 0.6200 140.0000

62 VLIQRNPQL 7.6440

V 0.5324 0.0000 77.8110 0.0000 1.0600 1.7000 0.6200 140.0000

L 1.2906 0.0000 84.5480 0.0000 1.2100 1.3000 0.6800 166.7000

I 1.1046 0.0000 88.6060 0.0000 1.0800 1.6000 0.6600 166.7000

Q 1.0036 -0.7211 70.0880 17.8660 1.1100 1.1000 0.8600 143.9000

R 1.2424 -1.4797 90.8010 35.3100 0.9800 0.9300 1.0400 173.4000

N 0.6396 -0.7211 50.5080 17.7800 0.6700 0.8900 1.3500 117.7000

P 0.3226 0.0000 69.2300 0.0000 0.5700 0.5500 1.5900 122.7000

Q 1.0036 -0.7211 70.0880 17.8660 1.1100 1.1000 0.8600 143.9000

L 1.2906 0.0000 84.5480 0.0000 1.2100 1.3000 0.6800 166.7000

63 SLYADSPSV 7.6580

S 0.2346 -0.6040 26.0680 15.9610 0.7700 0.7500 1.3200 89.0000

L 1.2906 0.0000 84.5480 0.0000 1.2100 1.3000 0.6800 166.7000

Y 0.4534 -0.5896 80.9650 42.7610 0.6900 1.4700 1.0600 193.6000

A 0.1744 0.0000 34.7760 0.0000 1.4200 0.8300 0.7000 88.6000

D 0.6058 -0.9298 37.4170 25.2733 1.0100 0.5400 1.2000 111.1000

S 0.2346 -0.6040 26.0680 15.9610 0.7700 0.7500 1.3200 89.0000

P 0.3226 0.0000 69.2300 0.0000 0.5700 0.5500 1.5900 122.7000

S 0.2346 -0.6040 26.0680 15.9610 0.7700 0.7500 1.3200 89.0000

V 0.5324 0.0000 77.8110 0.0000 1.0600 1.7000 0.6200 140.0000

64 ILSQVPFSV 7.6990

I 1.1046 0.0000 88.6060 0.0000 1.0800 1.6000 0.6600 166.7000

L 1.2906 0.0000 84.5480 0.0000 1.2100 1.3000 0.6800 166.7000

S 0.2346 -0.6040 26.0680 15.9610 0.7700 0.7500 1.3200 89.0000

Q 1.0036 -0.7211 70.0880 17.8660 1.1100 1.1000 0.8600 143.9000

V 0.5324 0.0000 77.8110 0.0000 1.0600 1.7000 0.6200 140.0000

P 0.3226 0.0000 69.2300 0.0000 0.5700 0.5500 1.5900 122.7000

F 0.4412 -0.1195 105.7050 11.2470 1.1300 1.3800 0.7100 189.9000

S 0.2346 -0.6040 26.0680 15.9610 0.7700 0.7500 1.3200 89.0000

V 0.5324 0.0000 77.8110 0.0000 1.0600 1.7000 0.6200 140.0000

65 IMDQVPFSV 7.7190

I 1.1046 0.0000 88.6060 0.0000 1.0800 1.6000 0.6600 166.7000

M 1.0768 -0.3068 70.3631 23.2300 1.4500 1.0500 0.5800 162.9000

D 0.6058 -0.9298 37.4170 25.2733 1.0100 0.5400 1.2000 111.1000

Q 1.0036 -0.7211 70.0880 17.8660 1.1100 1.1000 0.8600 143.9000

V 0.5324 0.0000 77.8110 0.0000 1.0600 1.7000 0.6200 140.0000

P 0.3226 0.0000 69.2300 0.0000 0.5700 0.5500 1.5900 122.7000

F 0.4412 -0.1195 105.7050 11.2470 1.1300 1.3800 0.7100 189.9000

S 0.2346 -0.6040 26.0680 15.9610 0.7700 0.7500 1.3200 89.0000

V 0.5324 0.0000 77.8110 0.0000 1.0600 1.7000 0.6200 140.0000

66 QLFEDNYAL 7.7640

Q 1.0036 -0.7211 70.0880 17.8660 1.1100 1.1000 0.8600 143.9000

L 1.2906 0.0000 84.5480 0.0000 1.2100 1.3000 0.6800 166.7000

F 0.4412 -0.1195 105.7050 11.2470 1.1300 1.3800 0.7100 189.9000

E 1.0315 -0.9298 57.1582 25.5730 1.5100 0.3700 0.8400 138.4000

D 0.6058 -0.9298 37.4170 25.2733 1.0100 0.5400 1.2000 111.1000

N 0.6396 -0.7211 50.5080 17.7800 0.6700 0.8900 1.3500 117.7000

Y 0.4534 -0.5896 80.9650 42.7610 0.6900 1.4700 1.0600 193.6000

A 0.1744 0.0000 34.7760 0.0000 1.4200 0.8300 0.7000 88.6000

L 1.2906 0.0000 84.5480 0.0000 1.2100 1.3000 0.6800 166.7000

67 ALMDKSLHV 7.7700

A 0.1744 0.0000 34.7760 0.0000 1.4200 0.8300 0.7000 88.6000

L 1.2906 0.0000 84.5480 0.0000 1.2100 1.3000 0.6800 166.7000

M 1.0768 -0.3068 70.3631 23.2300 1.4500 1.0500 0.5800 162.9000

D 0.6058 -0.9298 37.4170 25.2733 1.0100 0.5400 1.2000 111.1000

K 1.4600 -0.6229 97.7140 8.0790 1.1600 0.7400 0.9800 168.7000

S 0.2346 -0.6040 26.0680 15.9610 0.7700 0.7500 1.3200 89.0000

L 1.2906 0.0000 84.5480 0.0000 1.2100 1.3000 0.6800 166.7000

H 0.8124 -0.7766 82.1700 13.8630 1.0000 0.8700 1.0600 153.2000

V 0.5324 0.0000 77.8110 0.0000 1.0600 1.7000 0.6200 140.0000

68 YAIDLPVSV 7.7960

Y 0.4534 -0.5896 80.9650 42.7610 0.6900 1.4700 1.0600 193.6000

A 0.1744 0.0000 34.7760 0.0000 1.4200 0.8300 0.7000 88.6000

I 1.1046 0.0000 88.6060 0.0000 1.0800 1.6000 0.6600 166.7000

D 0.6058 -0.9298 37.4170 25.2733 1.0100 0.5400 1.2000 111.1000

L 1.2906 0.0000 84.5480 0.0000 1.2100 1.3000 0.6800 166.7000

P 0.3226 0.0000 69.2300 0.0000 0.5700 0.5500 1.5900 122.7000

V 0.5324 0.0000 77.8110 0.0000 1.0600 1.7000 0.6200 140.0000

S 0.2346 -0.6040 26.0680 15.9610 0.7700 0.7500 1.3200 89.0000

V 0.5324 0.0000 77.8110 0.0000 1.0600 1.7000 0.6200 140.0000

69 FVWLHYYSV 7.8240

F 0.4412 -0.1195 105.7050 11.2470 1.1300 1.3800 0.7100 189.9000

V 0.5324 0.0000 77.8110 0.0000 1.0600 1.7000 0.6200 140.0000

W 0.8364 -0.4310 133.6980 14.8820 1.0800 1.3700 0.7500 227.8000

L 1.2906 0.0000 84.5480 0.0000 1.2100 1.3000 0.6800 166.7000

H 0.8124 -0.7766 82.1700 13.8630 1.0000 0.8700 1.0600 153.2000

Y 0.4534 -0.5896 80.9650 42.7610 0.6900 1.4700 1.0600 193.6000

Y 0.4534 -0.5896 80.9650 42.7610 0.6900 1.4700 1.0600 193.6000

S 0.2346 -0.6040 26.0680 15.9610 0.7700 0.7500 1.3200 89.0000

V 0.5324 0.0000 77.8110 0.0000 1.0600 1.7000 0.6200 140.0000

70 MLGTHTMEV 7.8450

M 1.0768 -0.3068 70.3631 23.2300 1.4500 1.0500 0.5800 162.9000

L 1.2906 0.0000 84.5480 0.0000 1.2100 1.3000 0.6800 166.7000

G 0.0208 0.0000 3.7620 0.0000 0.5700 0.7500 1.5000 60.1000

T 1.4265 -0.4369 46.7290 16.0490 0.8300 1.1900 1.0700 116.1000

H 0.8124 -0.7766 82.1700 13.8630 1.0000 0.8700 1.0600 153.2000

T 1.4265 -0.4369 46.7290 16.0490 0.8300 1.1900 1.0700 116.1000

M 1.0768 -0.3068 70.3631 23.2300 1.4500 1.0500 0.5800 162.9000

E 1.0315 -0.9298 57.1582 25.5730 1.5100 0.3700 0.8400 138.4000

V 0.5324 0.0000 77.8110 0.0000 1.0600 1.7000 0.6200 140.0000

71 LLFGYPVYV 7.8860

L 1.2906 0.0000 84.5480 0.0000 1.2100 1.3000 0.6800 166.7000

L 1.2906 0.0000 84.5480 0.0000 1.2100 1.3000 0.6800 166.7000

F 0.4412 -0.1195 105.7050 11.2470 1.1300 1.3800 0.7100 189.9000

G 0.0208 0.0000 3.7620 0.0000 0.5700 0.7500 1.5000 60.1000

Y 0.4534 -0.5896 80.9650 42.7610 0.6900 1.4700 1.0600 193.6000

P 0.3226 0.0000 69.2300 0.0000 0.5700 0.5500 1.5900 122.7000

V 0.5324 0.0000 77.8110 0.0000 1.0600 1.7000 0.6200 140.0000

Y 0.4534 -0.5896 80.9650 42.7610 0.6900 1.4700 1.0600 193.6000

V 0.5324 0.0000 77.8110 0.0000 1.0600 1.7000 0.6200 140.0000

72 ILKEPVHGV 7.9210

I 1.1046 0.0000 88.6060 0.0000 1.0800 1.6000 0.6600 166.7000

L 1.2906 0.0000 84.5480 0.0000 1.2100 1.3000 0.6800 166.7000

K 1.4600 -0.6229 97.7140 8.0790 1.1600 0.7400 0.9800 168.7000

E 1.0315 -0.9298 57.1582 25.5730 1.5100 0.3700 0.8400 138.4000

P 0.3226 0.0000 69.2300 0.0000 0.5700 0.5500 1.5900 122.7000

V 0.5324 0.0000 77.8110 0.0000 1.0600 1.7000 0.6200 140.0000

H 0.8124 -0.7766 82.1700 13.8630 1.0000 0.8700 1.0600 153.2000

G 0.0208 0.0000 3.7620 0.0000 0.5700 0.7500 1.5000 60.1000

V 0.5324 0.0000 77.8110 0.0000 1.0600 1.7000 0.6200 140.0000

73 YLMPGPVTV 7.9320

Y 0.4534 -0.5896 80.9650 42.7610 0.6900 1.4700 1.0600 193.6000

L 1.2906 0.0000 84.5480 0.0000 1.2100 1.3000 0.6800 166.7000

M 1.0768 -0.3068 70.3631 23.2300 1.4500 1.0500 0.5800 162.9000

P 0.3226 0.0000 69.2300 0.0000 0.5700 0.5500 1.5900 122.7000

G 0.0208 0.0000 3.7620 0.0000 0.5700 0.7500 1.5000 60.1000

P 0.3226 0.0000 69.2300 0.0000 0.5700 0.5500 1.5900 122.7000

V 0.5324 0.0000 77.8110 0.0000 1.0600 1.7000 0.6200 140.0000

T 1.4265 -0.4369 46.7290 16.0490 0.8300 1.1900 1.0700 116.1000

V 0.5324 0.0000 77.8110 0.0000 1.0600 1.7000 0.6200 140.0000

74 WLDQVPFSV 7.9390

W 0.8364 -0.4310 133.6980 14.8820 1.0800 1.3700 0.7500 227.8000

L 1.2906 0.0000 84.5480 0.0000 1.2100 1.3000 0.6800 166.7000

D 0.6058 -0.9298 37.4170 25.2733 1.0100 0.5400 1.2000 111.1000

Q 1.0036 -0.7211 70.0880 17.8660 1.1100 1.1000 0.8600 143.9000

V 0.5324 0.0000 77.8110 0.0000 1.0600 1.7000 0.6200 140.0000

P 0.3226 0.0000 69.2300 0.0000 0.5700 0.5500 1.5900 122.7000

F 0.4412 -0.1195 105.7050 11.2470 1.1300 1.3800 0.7100 189.9000

S 0.2346 -0.6040 26.0680 15.9610 0.7700 0.7500 1.3200 89.0000

V 0.5324 0.0000 77.8110 0.0000 1.0600 1.7000 0.6200 140.0000

75 KTWGQYWQV 7.9550

K 1.4600 -0.6229 97.7140 8.0790 1.1600 0.7400 0.9800 168.7000

T 1.4265 -0.4369 46.7290 16.0490 0.8300 1.1900 1.0700 116.1000

W 0.8364 -0.4310 133.6980 14.8820 1.0800 1.3700 0.7500 227.8000

G 0.0208 0.0000 3.7620 0.0000 0.5700 0.7500 1.5000 60.1000

Q 1.0036 -0.7211 70.0880 17.8660 1.1100 1.1000 0.8600 143.9000

Y 0.4534 -0.5896 80.9650 42.7610 0.6900 1.4700 1.0600 193.6000

W 0.8364 -0.4310 133.6980 14.8820 1.0800 1.3700 0.7500 227.8000

Q 1.0036 -0.7211 70.0880 17.8660 1.1100 1.1000 0.8600 143.9000

V 0.5324 0.0000 77.8110 0.0000 1.0600 1.7000 0.6200 140.0000

76 ALMPLYACI 8.0000

A 0.1744 0.0000 34.7760 0.0000 1.4200 0.8300 0.7000 88.6000

L 1.2906 0.0000 84.5480 0.0000 1.2100 1.3000 0.6800 166.7000

M 1.0768 -0.3068 70.3631 23.2300 1.4500 1.0500 0.5800 162.9000

P 0.3226 0.0000 69.2300 0.0000 0.5700 0.5500 1.5900 122.7000

L 1.2906 0.0000 84.5480 0.0000 1.2100 1.3000 0.6800 166.7000

Y 0.4534 -0.5896 80.9650 42.7610 0.6900 1.4700 1.0600 193.6000

A 0.1744 0.0000 34.7760 0.0000 1.4200 0.8300 0.7000 88.6000

C 0.2479 -0.2402 23.5560 30.4540 0.7000 1.1900 1.1200 108.5000

I 1.1046 0.0000 88.6060 0.0000 1.0800 1.6000 0.6600 166.7000

77 YLAPGPVTA 8.0320

Y 0.4534 -0.5896 80.9650 42.7610 0.6900 1.4700 1.0600 193.6000

L 1.2906 0.0000 84.5480 0.0000 1.2100 1.3000 0.6800 166.7000

A 0.1744 0.0000 34.7760 0.0000 1.4200 0.8300 0.7000 88.6000

P 0.3226 0.0000 69.2300 0.0000 0.5700 0.5500 1.5900 122.7000

G 0.0208 0.0000 3.7620 0.0000 0.5700 0.7500 1.5000 60.1000

P 0.3226 0.0000 69.2300 0.0000 0.5700 0.5500 1.5900 122.7000

V 0.5324 0.0000 77.8110 0.0000 1.0600 1.7000 0.6200 140.0000

T 1.4265 -0.4369 46.7290 16.0490 0.8300 1.1900 1.0700 116.1000

A 0.1744 0.0000 34.7760 0.0000 1.4200 0.8300 0.7000 88.6000

78 YLYPGPVTV 8.0510

Y 0.4534 -0.5896 80.9650 42.7610 0.6900 1.4700 1.0600 193.6000

L 1.2906 0.0000 84.5480 0.0000 1.2100 1.3000 0.6800 166.7000

Y 0.4534 -0.5896 80.9650 42.7610 0.6900 1.4700 1.0600 193.6000

P 0.3226 0.0000 69.2300 0.0000 0.5700 0.5500 1.5900 122.7000

G 0.0208 0.0000 3.7620 0.0000 0.5700 0.7500 1.5000 60.1000

P 0.3226 0.0000 69.2300 0.0000 0.5700 0.5500 1.5900 122.7000

V 0.5324 0.0000 77.8110 0.0000 1.0600 1.7000 0.6200 140.0000

T 1.4265 -0.4369 46.7290 16.0490 0.8300 1.1900 1.0700 116.1000

V 0.5324 0.0000 77.8110 0.0000 1.0600 1.7000 0.6200 140.0000

79 LLMGTLGIV 8.0970

L 1.2906 0.0000 84.5480 0.0000 1.2100 1.3000 0.6800 166.7000

L 1.2906 0.0000 84.5480 0.0000 1.2100 1.3000 0.6800 166.7000

M 1.0768 -0.3068 70.3631 23.2300 1.4500 1.0500 0.5800 162.9000

G 0.0208 0.0000 3.7620 0.0000 0.5700 0.7500 1.5000 60.1000

T 1.4265 -0.4369 46.7290 16.0490 0.8300 1.1900 1.0700 116.1000

L 1.2906 0.0000 84.5480 0.0000 1.2100 1.3000 0.6800 166.7000

G 0.0208 0.0000 3.7620 0.0000 0.5700 0.7500 1.5000 60.1000

I 1.1046 0.0000 88.6060 0.0000 1.0800 1.6000 0.6600 166.7000

V 0.5324 0.0000 77.8110 0.0000 1.0600 1.7000 0.6200 140.0000

80 YLWPGPVTV 8.1250

Y 0.4534 -0.5896 80.9650 42.7610 0.6900 1.4700 1.0600 193.6000

L 1.2906 0.0000 84.5480 0.0000 1.2100 1.3000 0.6800 166.7000

W 0.8364 -0.4310 133.6980 14.8820 1.0800 1.3700 0.7500 227.8000

P 0.3226 0.0000 69.2300 0.0000 0.5700 0.5500 1.5900 122.7000

G 0.0208 0.0000 3.7620 0.0000 0.5700 0.7500 1.5000 60.1000

P 0.3226 0.0000 69.2300 0.0000 0.5700 0.5500 1.5900 122.7000

V 0.5324 0.0000 77.8110 0.0000 1.0600 1.7000 0.6200 140.0000

T 1.4265 -0.4369 46.7290 16.0490 0.8300 1.1900 1.0700 116.1000

V 0.5324 0.0000 77.8110 0.0000 1.0600 1.7000 0.6200 140.0000

81 FLLTRILTI 8.1490

F 0.4412 -0.1195 105.7050 11.2470 1.1300 1.3800 0.7100 189.9000

L 1.2906 0.0000 84.5480 0.0000 1.2100 1.3000 0.6800 166.7000

L 1.2906 0.0000 84.5480 0.0000 1.2100 1.3000 0.6800 166.7000

T 1.4265 -0.4369 46.7290 16.0490 0.8300 1.1900 1.0700 116.1000

R 1.2424 -1.4797 90.8010 35.3100 0.9800 0.9300 1.0400 173.4000

I 1.1046 0.0000 88.6060 0.0000 1.0800 1.6000 0.6600 166.7000

L 1.2906 0.0000 84.5480 0.0000 1.2100 1.3000 0.6800 166.7000

T 1.4265 -0.4369 46.7290 16.0490 0.8300 1.1900 1.0700 116.1000

I 1.1046 0.0000 88.6060 0.0000 1.0800 1.6000 0.6600 166.7000

82 GLLGWSPQA 8.2370

G 0.0208 0.0000 3.7620 0.0000 0.5700 0.7500 1.5000 60.1000

L 1.2906 0.0000 84.5480 0.0000 1.2100 1.3000 0.6800 166.7000

L 1.2906 0.0000 84.5480 0.0000 1.2100 1.3000 0.6800 166.7000

G 0.0208 0.0000 3.7620 0.0000 0.5700 0.7500 1.5000 60.1000

W 0.8364 -0.4310 133.6980 14.8820 1.0800 1.3700 0.7500 227.8000

S 0.2346 -0.6040 26.0680 15.9610 0.7700 0.7500 1.3200 89.0000

P 0.3226 0.0000 69.2300 0.0000 0.5700 0.5500 1.5900 122.7000

Q 1.0036 -0.7211 70.0880 17.8660 1.1100 1.1000 0.8600 143.9000

A 0.1744 0.0000 34.7760 0.0000 1.4200 0.8300 0.7000 88.6000

83 ILYQVPFSV 8.3100

I 1.1046 0.0000 88.6060 0.0000 1.0800 1.6000 0.6600 166.7000

L 1.2906 0.0000 84.5480 0.0000 1.2100 1.3000 0.6800 166.7000

Y 0.4534 -0.5896 80.9650 42.7610 0.6900 1.4700 1.0600 193.6000

Q 1.0036 -0.7211 70.0880 17.8660 1.1100 1.1000 0.8600 143.9000

V 0.5324 0.0000 77.8110 0.0000 1.0600 1.7000 0.6200 140.0000

P 0.3226 0.0000 69.2300 0.0000 0.5700 0.5500 1.5900 122.7000

F 0.4412 -0.1195 105.7050 11.2470 1.1300 1.3800 0.7100 189.9000

S 0.2346 -0.6040 26.0680 15.9610 0.7700 0.7500 1.3200 89.0000

V 0.5324 0.0000 77.8110 0.0000 1.0600 1.7000 0.6200 140.0000

84 GILTVILGV 8.3470

G 0.0208 0.0000 3.7620 0.0000 0.5700 0.7500 1.5000 60.1000

I 1.1046 0.0000 88.6060 0.0000 1.0800 1.6000 0.6600 166.7000

L 1.2906 0.0000 84.5480 0.0000 1.2100 1.3000 0.6800 166.7000

T 1.4265 -0.4369 46.7290 16.0490 0.8300 1.1900 1.0700 116.1000

V 0.5324 0.0000 77.8110 0.0000 1.0600 1.7000 0.6200 140.0000

I 1.1046 0.0000 88.6060 0.0000 1.0800 1.6000 0.6600 166.7000

L 1.2906 0.0000 84.5480 0.0000 1.2100 1.3000 0.6800 166.7000

G 0.0208 0.0000 3.7620 0.0000 0.5700 0.7500 1.5000 60.1000

V 0.5324 0.0000 77.8110 0.0000 1.0600 1.7000 0.6200 140.0000

85 NMVPFFPPV 8.3980

N 0.6396 -0.7211 50.5080 17.7800 0.6700 0.8900 1.3500 117.7000

M 1.0768 -0.3068 70.3631 23.2300 1.4500 1.0500 0.5800 162.9000

V 0.5324 0.0000 77.8110 0.0000 1.0600 1.7000 0.6200 140.0000

P 0.3226 0.0000 69.2300 0.0000 0.5700 0.5500 1.5900 122.7000

F 0.4412 -0.1195 105.7050 11.2470 1.1300 1.3800 0.7100 189.9000

F 0.4412 -0.1195 105.7050 11.2470 1.1300 1.3800 0.7100 189.9000

P 0.3226 0.0000 69.2300 0.0000 0.5700 0.5500 1.5900 122.7000

P 0.3226 0.0000 69.2300 0.0000 0.5700 0.5500 1.5900 122.7000

V 0.5324 0.0000 77.8110 0.0000 1.0600 1.7000 0.6200 140.0000

86 ILDQVPFSV 8.4810

I 1.1046 0.0000 88.6060 0.0000 1.0800 1.6000 0.6600 166.7000

L 1.2906 0.0000 84.5480 0.0000 1.2100 1.3000 0.6800 166.7000

D 0.6058 -0.9298 37.4170 25.2733 1.0100 0.5400 1.2000 111.1000

Q 1.0036 -0.7211 70.0880 17.8660 1.1100 1.1000 0.8600 143.9000

V 0.5324 0.0000 77.8110 0.0000 1.0600 1.7000 0.6200 140.0000

P 0.3226 0.0000 69.2300 0.0000 0.5700 0.5500 1.5900 122.7000

F 0.4412 -0.1195 105.7050 11.2470 1.1300 1.3800 0.7100 189.9000

S 0.2346 -0.6040 26.0680 15.9610 0.7700 0.7500 1.3200 89.0000

V 0.5324 0.0000 77.8110 0.0000 1.0600 1.7000 0.6200 140.0000

87 YLFPGPVTA 8.4950

Y 0.4534 -0.5896 80.9650 42.7610 0.6900 1.4700 1.0600 193.6000

L 1.2906 0.0000 84.5480 0.0000 1.2100 1.3000 0.6800 166.7000

F 0.4412 -0.1195 105.7050 11.2470 1.1300 1.3800 0.7100 189.9000

P 0.3226 0.0000 69.2300 0.0000 0.5700 0.5500 1.5900 122.7000

G 0.0208 0.0000 3.7620 0.0000 0.5700 0.7500 1.5000 60.1000

P 0.3226 0.0000 69.2300 0.0000 0.5700 0.5500 1.5900 122.7000

V 0.5324 0.0000 77.8110 0.0000 1.0600 1.7000 0.6200 140.0000

T 1.4265 -0.4369 46.7290 16.0490 0.8300 1.1900 1.0700 116.1000

A 0.1744 0.0000 34.7760 0.0000 1.4200 0.8300 0.7000 88.6000

88 YLDQVPFSV 8.6380

Y 0.4534 -0.5896 80.9650 42.7610 0.6900 1.4700 1.0600 193.6000

L 1.2906 0.0000 84.5480 0.0000 1.2100 1.3000 0.6800 166.7000

D 0.6058 -0.9298 37.4170 25.2733 1.0100 0.5400 1.2000 111.1000

Q 1.0036 -0.7211 70.0880 17.8660 1.1100 1.1000 0.8600 143.9000

V 0.5324 0.0000 77.8110 0.0000 1.0600 1.7000 0.6200 140.0000

P 0.3226 0.0000 69.2300 0.0000 0.5700 0.5500 1.5900 122.7000

F 0.4412 -0.1195 105.7050 11.2470 1.1300 1.3800 0.7100 189.9000

S 0.2346 -0.6040 26.0680 15.9610 0.7700 0.7500 1.3200 89.0000

V 0.5324 0.0000 77.8110 0.0000 1.0600 1.7000 0.6200 140.0000

89 ILFQVPFSV 8.6990

I 1.1046 0.0000 88.6060 0.0000 1.0800 1.6000 0.6600 166.7000

L 1.2906 0.0000 84.5480 0.0000 1.2100 1.3000 0.6800 166.7000

F 0.4412 -0.1195 105.7050 11.2470 1.1300 1.3800 0.7100 189.9000

Q 1.0036 -0.7211 70.0880 17.8660 1.1100 1.1000 0.8600 143.9000

V 0.5324 0.0000 77.8110 0.0000 1.0600 1.7000 0.6200 140.0000

P 0.3226 0.0000 69.2300 0.0000 0.5700 0.5500 1.5900 122.7000

F 0.4412 -0.1195 105.7050 11.2470 1.1300 1.3800 0.7100 189.9000

S 0.2346 -0.6040 26.0680 15.9610 0.7700 0.7500 1.3200 89.0000

V 0.5324 0.0000 77.8110 0.0000 1.0600 1.7000 0.6200 140.0000

90 ILWQVPFSV 8.7700

I 1.1046 0.0000 88.6060 0.0000 1.0800 1.6000 0.6600 166.7000

L 1.2906 0.0000 84.5480 0.0000 1.2100 1.3000 0.6800 166.7000

W 0.8364 -0.4310 133.6980 14.8820 1.0800 1.3700 0.7500 227.8000

Q 1.0036 -0.7211 70.0880 17.8660 1.1100 1.1000 0.8600 143.9000

V 0.5324 0.0000 77.8110 0.0000 1.0600 1.7000 0.6200 140.0000

P 0.3226 0.0000 69.2300 0.0000 0.5700 0.5500 1.5900 122.7000

F 0.4412 -0.1195 105.7050 11.2470 1.1300 1.3800 0.7100 189.9000

S 0.2346 -0.6040 26.0680 15.9610 0.7700 0.7500 1.3200 89.0000

V 0.5324 0.0000 77.8110 0.0000 1.0600 1.7000 0.6200 140.0000

3D DATA Matrix of Unknown Peptides:

1 LLGCAANWI 5.3010

V 1.2906 0.0000 84.5480 0.0000 1.2100 1.3000 0.6800 166.7000

A 1.2906 0.0000 84.5480 0.0000 1.2100 1.3000 0.6800 166.7000

L 0.0208 0.0000 3.7620 0.0000 0.5700 0.7500 1.5000 60.1000

V 0.2479 -0.2402 23.5560 30.4540 0.7000 1.1900 1.1200 108.5000

G 0.1744 0.0000 34.7760 0.0000 1.4200 0.8300 0.7000 88.6000

L 0.1744 0.0000 34.7760 0.0000 1.4200 0.8300 0.7000 88.6000

F 0.6396 -0.7211 50.5080 17.7800 0.6700 0.8900 1.3500 117.7000

V 0.8364 -0.4310 133.6980 14.8820 1.0800 1.3700 0.7500 227.8000

L 1.1046 0.0000 88.6060 0.0000 1.0800 1.6000 0.6600 166.7000

2 SAANDPIFV 5.3420

G 0.2346 -0.6040 26.0680 15.9610 0.7700 0.7500 1.3200 89.0000

T 0.1744 0.0000 34.7760 0.0000 1.4200 0.8300 0.7000 88.6000

L 0.1744 0.0000 34.7760 0.0000 1.4200 0.8300 0.7000 88.6000

V 0.6396 -0.7211 50.5080 17.7800 0.6700 0.8900 1.3500 117.7000

A 0.6058 -0.9298 37.4170 25.2733 1.0100 0.5400 1.2000 111.1000

L 0.3226 0.0000 69.2300 0.0000 0.5700 0.5500 1.5900 122.7000

V 1.1046 0.0000 88.6060 0.0000 1.0800 1.6000 0.6600 166.7000

G 0.4412 -0.1195 105.7050 11.2470 1.1300 1.3800 0.7100 189.9000

L 0.5324 0.0000 77.8110 0.0000 1.0600 1.7000 0.6200 140.0000

3 TTAEEAAGI 5.3800

L 1.4265 -0.4369 46.7290 16.0490 0.8300 1.1900 1.0700 116.1000

Q 1.4265 -0.4369 46.7290 16.0490 0.8300 1.1900 1.0700 116.1000

T 0.1744 0.0000 34.7760 0.0000 1.4200 0.8300 0.7000 88.6000

T 1.0315 -0.9298 57.1582 25.5730 1.5100 0.3700 0.8400 138.4000

I 1.0315 -0.9298 57.1582 25.5730 1.5100 0.3700 0.8400 138.4000

H 0.1744 0.0000 34.7760 0.0000 1.4200 0.8300 0.7000 88.6000

D 0.1744 0.0000 34.7760 0.0000 1.4200 0.8300 0.7000 88.6000

I 0.0208 0.0000 3.7620 0.0000 0.5700 0.7500 1.5000 60.1000

I 1.1046 0.0000 88.6060 0.0000 1.0800 1.6000 0.6600 166.7000

4 LTVILGVLL 5.5800

S 1.2906 0.0000 84.5480 0.0000 1.2100 1.3000 0.6800 166.7000

L 1.4265 -0.4369 46.7290 16.0490 0.8300 1.1900 1.0700 116.1000

H 0.5324 0.0000 77.8110 0.0000 1.0600 1.7000 0.6200 140.0000

V 1.1046 0.0000 88.6060 0.0000 1.0800 1.6000 0.6600 166.7000

G 1.2906 0.0000 84.5480 0.0000 1.2100 1.3000 0.6800 166.7000

T 0.0208 0.0000 3.7620 0.0000 0.5700 0.7500 1.5000 60.1000

Q 0.5324 0.0000 77.8110 0.0000 1.0600 1.7000 0.6200 140.0000

C 1.2906 0.0000 84.5480 0.0000 1.2100 1.3000 0.6800 166.7000

A 1.2906 0.0000 84.5480 0.0000 1.2100 1.3000 0.6800 166.7000

5 HLLVGSSGL 5.7920

A 0.8124 -0.7766 82.1700 13.8630 1.0000 0.8700 1.0600 153.2000

L 1.2906 0.0000 84.5480 0.0000 1.2100 1.3000 0.6800 166.7000

P 1.2906 0.0000 84.5480 0.0000 1.2100 1.3000 0.6800 166.7000

Y 0.5324 0.0000 77.8110 0.0000 1.0600 1.7000 0.6200 140.0000

W 0.0208 0.0000 3.7620 0.0000 0.5700 0.7500 1.5000 60.1000

N 0.2346 -0.6040 26.0680 15.9610 0.7700 0.7500 1.3200 89.0000

F 0.2346 -0.6040 26.0680 15.9610 0.7700 0.7500 1.3200 89.0000

A 0.0208 0.0000 3.7620 0.0000 0.5700 0.7500 1.5000 60.1000

T 1.2906 0.0000 84.5480 0.0000 1.2100 1.3000 0.6800 166.7000

6 GIGILTVIL 6.0000

S 0.0208 0.0000 3.7620 0.0000 0.5700 0.7500 1.5000 60.1000

L 1.1046 0.0000 88.6060 0.0000 1.0800 1.6000 0.6600 166.7000

N 0.0208 0.0000 3.7620 0.0000 0.5700 0.7500 1.5000 60.1000

F 1.1046 0.0000 88.6060 0.0000 1.0800 1.6000 0.6600 166.7000

M 1.2906 0.0000 84.5480 0.0000 1.2100 1.3000 0.6800 166.7000

G 1.4265 -0.4369 46.7290 16.0490 0.8300 1.1900 1.0700 116.1000

Y 0.5324 0.0000 77.8110 0.0000 1.0600 1.7000 0.6200 140.0000

V 1.1046 0.0000 88.6060 0.0000 1.0800 1.6000 0.6600 166.7000

I 1.2906 0.0000 84.5480 0.0000 1.2100 1.3000 0.6800 166.7000

7 TVILGVLLL 6.0720

N 1.4265 -0.4369 46.7290 16.0490 0.8300 1.1900 1.0700 116.1000

L 0.5324 0.0000 77.8110 0.0000 1.0600 1.7000 0.6200 140.0000

Q 1.1046 0.0000 88.6060 0.0000 1.0800 1.6000 0.6600 166.7000

S 1.2906 0.0000 84.5480 0.0000 1.2100 1.3000 0.6800 166.7000

L 0.0208 0.0000 3.7620 0.0000 0.5700 0.7500 1.5000 60.1000

T 0.5324 0.0000 77.8110 0.0000 1.0600 1.7000 0.6200 140.0000

N 1.2906 0.0000 84.5480 0.0000 1.2100 1.3000 0.6800 166.7000

L 1.2906 0.0000 84.5480 0.0000 1.2100 1.3000 0.6800 166.7000

L 1.2906 0.0000 84.5480 0.0000 1.2100 1.3000 0.6800 166.7000

8 WTDQVPFSV 6.1450

F 0.8364 -0.4310 133.6980 14.8820 1.0800 1.3700 0.7500 227.8000

V 1.4265 -0.4369 46.7290 16.0490 0.8300 1.1900 1.0700 116.1000

T 0.6058 -0.9298 37.4170 25.2733 1.0100 0.5400 1.2000 111.1000

W 1.0036 -0.7211 70.0880 17.8660 1.1100 1.1000 0.8600 143.9000

H 0.5324 0.0000 77.8110 0.0000 1.0600 1.7000 0.6200 140.0000

R 0.3226 0.0000 69.2300 0.0000 0.5700 0.5500 1.5900 122.7000

Y 0.4412 -0.1195 105.7050 11.2470 1.1300 1.3800 0.7100 189.9000

H 0.2346 -0.6040 26.0680 15.9610 0.7700 0.7500 1.3200 89.0000

L 0.5324 0.0000 77.8110 0.0000 1.0600 1.7000 0.6200 140.0000

9 AIAKAAAAV 6.1760

D 0.1744 0.0000 34.7760 0.0000 1.4200 0.8300 0.7000 88.6000

P 1.1046 0.0000 88.6060 0.0000 1.0800 1.6000 0.6600 166.7000

K 0.1744 0.0000 34.7760 0.0000 1.4200 0.8300 0.7000 88.6000

V 1.4600 -0.6229 97.7140 8.0790 1.1600 0.7400 0.9800 168.7000

K 0.1744 0.0000 34.7760 0.0000 1.4200 0.8300 0.7000 88.6000

Q 0.1744 0.0000 34.7760 0.0000 1.4200 0.8300 0.7000 88.6000

W 0.1744 0.0000 34.7760 0.0000 1.4200 0.8300 0.7000 88.6000

P 0.1744 0.0000 34.7760 0.0000 1.4200 0.8300 0.7000 88.6000

L 0.5324 0.0000 77.8110 0.0000 1.0600 1.7000 0.6200 140.0000

10 ILTVILGVL 6.4190

I 1.1046 0.0000 88.6060 0.0000 1.0800 1.6000 0.6600 166.7000

T 1.2906 0.0000 84.5480 0.0000 1.2100 1.3000 0.6800 166.7000

S 1.4265 -0.4369 46.7290 16.0490 0.8300 1.1900 1.0700 116.1000

Q 0.5324 0.0000 77.8110 0.0000 1.0600 1.7000 0.6200 140.0000

V 1.1046 0.0000 88.6060 0.0000 1.0800 1.6000 0.6600 166.7000

P 1.2906 0.0000 84.5480 0.0000 1.2100 1.3000 0.6800 166.7000

F 0.0208 0.0000 3.7620 0.0000 0.5700 0.7500 1.5000 60.1000

S 0.5324 0.0000 77.8110 0.0000 1.0600 1.7000 0.6200 140.0000

V 1.2906 0.0000 84.5480 0.0000 1.2100 1.3000 0.6800 166.7000

11 AVAKAAAAV 6.4950

A 0.1744 0.0000 34.7760 0.0000 1.4200 0.8300 0.7000 88.6000

L 0.5324 0.0000 77.8110 0.0000 1.0600 1.7000 0.6200 140.0000

A 0.1744 0.0000 34.7760 0.0000 1.4200 0.8300 0.7000 88.6000

K 1.4600 -0.6229 97.7140 8.0790 1.1600 0.7400 0.9800 168.7000

A 0.1744 0.0000 34.7760 0.0000 1.4200 0.8300 0.7000 88.6000

A 0.1744 0.0000 34.7760 0.0000 1.4200 0.8300 0.7000 88.6000

A 0.1744 0.0000 34.7760 0.0000 1.4200 0.8300 0.7000 88.6000

A 0.1744 0.0000 34.7760 0.0000 1.4200 0.8300 0.7000 88.6000

I 0.5324 0.0000 77.8110 0.0000 1.0600 1.7000 0.6200 140.0000

12 ILDEAYVMA 6.6230

G 1.1046 0.0000 88.6060 0.0000 1.0800 1.6000 0.6600 166.7000

L 1.2906 0.0000 84.5480 0.0000 1.2100 1.3000 0.6800 166.7000

G 0.6058 -0.9298 37.4170 25.2733 1.0100 0.5400 1.2000 111.1000

Q 1.0315 -0.9298 57.1582 25.5730 1.5100 0.3700 0.8400 138.4000

V 0.1744 0.0000 34.7760 0.0000 1.4200 0.8300 0.7000 88.6000

P 0.4534 -0.5896 80.9650 42.7610 0.6900 1.4700 1.0600 193.6000

L 0.5324 0.0000 77.8110 0.0000 1.0600 1.7000 0.6200 140.0000

I 1.0768 -0.3068 70.3631 23.2300 1.4500 1.0500 0.5800 162.9000

V 0.1744 0.0000 34.7760 0.0000 1.4200 0.8300 0.7000 88.6000

13 LLWFHISCL 6.6820

M 1.2906 0.0000 84.5480 0.0000 1.2100 1.3000 0.6800 166.7000

L 1.2906 0.0000 84.5480 0.0000 1.2100 1.3000 0.6800 166.7000

D 0.8364 -0.4310 133.6980 14.8820 1.0800 1.3700 0.7500 227.8000

L 0.4412 -0.1195 105.7050 11.2470 1.1300 1.3800 0.7100 189.9000

Q 0.8124 -0.7766 82.1700 13.8630 1.0000 0.8700 1.0600 153.2000

P 1.1046 0.0000 88.6060 0.0000 1.0800 1.6000 0.6600 166.7000

E 0.2346 -0.6040 26.0680 15.9610 0.7700 0.7500 1.3200 89.0000

T 0.2479 -0.2402 23.5560 30.4540 0.7000 1.1900 1.1200 108.5000

T 1.2906 0.0000 84.5480 0.0000 1.2100 1.3000 0.6800 166.7000

14 TLDSQVMSL 6.7930

L 1.4265 -0.4369 46.7290 16.0490 0.8300 1.1900 1.0700 116.1000

L 1.2906 0.0000 84.5480 0.0000 1.2100 1.3000 0.6800 166.7000

S 0.6058 -0.9298 37.4170 25.2733 1.0100 0.5400 1.2000 111.1000

S 0.2346 -0.6040 26.0680 15.9610 0.7700 0.7500 1.3200 89.0000

N 1.0036 -0.7211 70.0880 17.8660 1.1100 1.1000 0.8600 143.9000

L 0.5324 0.0000 77.8110 0.0000 1.0600 1.7000 0.6200 140.0000

S 1.0768 -0.3068 70.3631 23.2300 1.4500 1.0500 0.5800 162.9000

W 0.2346 -0.6040 26.0680 15.9610 0.7700 0.7500 1.3200 89.0000

L 1.2906 0.0000 84.5480 0.0000 1.2100 1.3000 0.6800 166.7000

15 HLYQGCQVV 6.8320

G 0.8124 -0.7766 82.1700 13.8630 1.0000 0.8700 1.0600 153.2000

L 1.2906 0.0000 84.5480 0.0000 1.2100 1.3000 0.6800 166.7000

A 0.4534 -0.5896 80.9650 42.7610 0.6900 1.4700 1.0600 193.6000

C 1.0036 -0.7211 70.0880 17.8660 1.1100 1.1000 0.8600 143.9000

H 0.0208 0.0000 3.7620 0.0000 0.5700 0.7500 1.5000 60.1000

Q 0.2479 -0.2402 23.5560 30.4540 0.7000 1.1900 1.1200 108.5000

L 1.0036 -0.7211 70.0880 17.8660 1.1100 1.1000 0.8600 143.9000

C 0.5324 0.0000 77.8110 0.0000 1.0600 1.7000 0.6200 140.0000

A 0.5324 0.0000 77.8110 0.0000 1.0600 1.7000 0.6200 140.0000

16 QLFHLCLII 6.8860

L 1.0036 -0.7211 70.0880 17.8660 1.1100 1.1000 0.8600 143.9000

I 1.2906 0.0000 84.5480 0.0000 1.2100 1.3000 0.6800 166.7000

G 0.4412 -0.1195 105.7050 11.2470 1.1300 1.3800 0.7100 189.9000

N 0.8124 -0.7766 82.1700 13.8630 1.0000 0.8700 1.0600 153.2000

E 1.2906 0.0000 84.5480 0.0000 1.2100 1.3000 0.6800 166.7000

S 0.2479 -0.2402 23.5560 30.4540 0.7000 1.1900 1.1200 108.5000

F 1.2906 0.0000 84.5480 0.0000 1.2100 1.3000 0.6800 166.7000

A 1.1046 0.0000 88.6060 0.0000 1.0800 1.6000 0.6600 166.7000

L 1.1046 0.0000 88.6060 0.0000 1.0800 1.6000 0.6600 166.7000

17 ITDQVPFSV 6.9470

A 1.1046 0.0000 88.6060 0.0000 1.0800 1.6000 0.6600 166.7000

L 1.4265 -0.4369 46.7290 16.0490 0.8300 1.1900 1.0700 116.1000

A 0.6058 -0.9298 37.4170 25.2733 1.0100 0.5400 1.2000 111.1000

K 1.0036 -0.7211 70.0880 17.8660 1.1100 1.1000 0.8600 143.9000

A 0.5324 0.0000 77.8110 0.0000 1.0600 1.7000 0.6200 140.0000

A 0.3226 0.0000 69.2300 0.0000 0.5700 0.5500 1.5900 122.7000

A 0.4412 -0.1195 105.7050 11.2470 1.1300 1.3800 0.7100 189.9000

A 0.2346 -0.6040 26.0680 15.9610 0.7700 0.7500 1.3200 89.0000

V 0.5324 0.0000 77.8110 0.0000 1.0600 1.7000 0.6200 140.0000

18 ALCRWGLLL 7.0000

L 0.1744 0.0000 34.7760 0.0000 1.4200 0.8300 0.7000 88.6000

L 1.2906 0.0000 84.5480 0.0000 1.2100 1.3000 0.6800 166.7000

A 0.2479 -0.2402 23.5560 30.4540 0.7000 1.1900 1.1200 108.5000

V 1.2424 -1.4797 90.8010 35.3100 0.9800 0.9300 1.0400 173.4000

G 0.8364 -0.4310 133.6980 14.8820 1.0800 1.3700 0.7500 227.8000

A 0.0208 0.0000 3.7620 0.0000 0.5700 0.7500 1.5000 60.1000

T 1.2906 0.0000 84.5480 0.0000 1.2100 1.3000 0.6800 166.7000

K 1.2906 0.0000 84.5480 0.0000 1.2100 1.3000 0.6800 166.7000

V 1.2906 0.0000 84.5480 0.0000 1.2100 1.3000 0.6800 166.7000

19 NLGNLNVSI 7.1190

A 0.6396 -0.7211 50.5080 17.7800 0.6700 0.8900 1.3500 117.7000

L 1.2906 0.0000 84.5480 0.0000 1.2100 1.3000 0.6800 166.7000

A 0.0208 0.0000 3.7620 0.0000 0.5700 0.7500 1.5000 60.1000

K 0.6396 -0.7211 50.5080 17.7800 0.6700 0.8900 1.3500 117.7000

A 1.2906 0.0000 84.5480 0.0000 1.2100 1.3000 0.6800 166.7000

A 0.6396 -0.7211 50.5080 17.7800 0.6700 0.8900 1.3500 117.7000

A 0.5324 0.0000 77.8110 0.0000 1.0600 1.7000 0.6200 140.0000

A 0.2346 -0.6040 26.0680 15.9610 0.7700 0.7500 1.3200 89.0000

L 1.1046 0.0000 88.6060 0.0000 1.0800 1.6000 0.6600 166.7000

20 HLYSHPIIL 7.1310

W 0.8124 -0.7766 82.1700 13.8630 1.0000 0.8700 1.0600 153.2000

I 1.2906 0.0000 84.5480 0.0000 1.2100 1.3000 0.6800 166.7000

L 0.4534 -0.5896 80.9650 42.7610 0.6900 1.4700 1.0600 193.6000

R 0.2346 -0.6040 26.0680 15.9610 0.7700 0.7500 1.3200 89.0000

G 0.8124 -0.7766 82.1700 13.8630 1.0000 0.8700 1.0600 153.2000

T 0.3226 0.0000 69.2300 0.0000 0.5700 0.5500 1.5900 122.7000

S 1.1046 0.0000 88.6060 0.0000 1.0800 1.6000 0.6600 166.7000

F 1.1046 0.0000 88.6060 0.0000 1.0800 1.6000 0.6600 166.7000

V 1.2906 0.0000 84.5480 0.0000 1.2100 1.3000 0.6800 166.7000

21 ITFQVPFSV 7.1790

I 1.1046 0.0000 88.6060 0.0000 1.0800 1.6000 0.6600 166.7000

I 1.4265 -0.4369 46.7290 16.0490 0.8300 1.1900 1.0700 116.1000

S 0.4412 -0.1195 105.7050 11.2470 1.1300 1.3800 0.7100 189.9000

C 1.0036 -0.7211 70.0880 17.8660 1.1100 1.1000 0.8600 143.9000

T 0.5324 0.0000 77.8110 0.0000 1.0600 1.7000 0.6200 140.0000

C 0.3226 0.0000 69.2300 0.0000 0.5700 0.5500 1.5900 122.7000

P 0.4412 -0.1195 105.7050 11.2470 1.1300 1.3800 0.7100 189.9000

T 0.2346 -0.6040 26.0680 15.9610 0.7700 0.7500 1.3200 89.0000

V 0.5324 0.0000 77.8110 0.0000 1.0600 1.7000 0.6200 140.0000

22 FTDQVPFSV 7.2120

F 0.4412 -0.1195 105.7050 11.2470 1.1300 1.3800 0.7100 189.9000

L 1.4265 -0.4369 46.7290 16.0490 0.8300 1.1900 1.0700 116.1000

G 0.6058 -0.9298 37.4170 25.2733 1.0100 0.5400 1.2000 111.1000

G 1.0036 -0.7211 70.0880 17.8660 1.1100 1.1000 0.8600 143.9000

T 0.5324 0.0000 77.8110 0.0000 1.0600 1.7000 0.6200 140.0000

P 0.3226 0.0000 69.2300 0.0000 0.5700 0.5500 1.5900 122.7000

V 0.4412 -0.1195 105.7050 11.2470 1.1300 1.3800 0.7100 189.9000

C 0.2346 -0.6040 26.0680 15.9610 0.7700 0.7500 1.3200 89.0000

L 0.5324 0.0000 77.8110 0.0000 1.0600 1.7000 0.6200 140.0000

23 RLMKQDFSV 7.3420

A 1.2424 -1.4797 90.8010 35.3100 0.9800 0.9300 1.0400 173.4000

L 1.2906 0.0000 84.5480 0.0000 1.2100 1.3000 0.6800 166.7000

I 1.0768 -0.3068 70.3631 23.2300 1.4500 1.0500 0.5800 162.9000

H 1.4600 -0.6229 97.7140 8.0790 1.1600 0.7400 0.9800 168.7000

H 1.0036 -0.7211 70.0880 17.8660 1.1100 1.1000 0.8600 143.9000

N 0.6058 -0.9298 37.4170 25.2733 1.0100 0.5400 1.2000 111.1000

T 0.4412 -0.1195 105.7050 11.2470 1.1300 1.3800 0.7100 189.9000

H 0.2346 -0.6040 26.0680 15.9610 0.7700 0.7500 1.3200 89.0000

L 0.5324 0.0000 77.8110 0.0000 1.0600 1.7000 0.6200 140.0000

24 KLHLYSHPI 7.3520

N 1.4600 -0.6229 97.7140 8.0790 1.1600 0.7400 0.9800 168.7000

L 1.2906 0.0000 84.5480 0.0000 1.2100 1.3000 0.6800 166.7000

S 0.8124 -0.7766 82.1700 13.8630 1.0000 0.8700 1.0600 153.2000

W 1.2906 0.0000 84.5480 0.0000 1.2100 1.3000 0.6800 166.7000

L 0.4534 -0.5896 80.9650 42.7610 0.6900 1.4700 1.0600 193.6000

S 0.2346 -0.6040 26.0680 15.9610 0.7700 0.7500 1.3200 89.0000

L 0.8124 -0.7766 82.1700 13.8630 1.0000 0.8700 1.0600 153.2000

D 0.3226 0.0000 69.2300 0.0000 0.5700 0.5500 1.5900 122.7000

V 1.1046 0.0000 88.6060 0.0000 1.0800 1.6000 0.6600 166.7000

25 ITMQVPFSV 7.3980

Y 1.1046 0.0000 88.6060 0.0000 1.0800 1.6000 0.6600 166.7000

M 1.4265 -0.4369 46.7290 16.0490 0.8300 1.1900 1.0700 116.1000

I 1.0768 -0.3068 70.3631 23.2300 1.4500 1.0500 0.5800 162.9000

M 1.0036 -0.7211 70.0880 17.8660 1.1100 1.1000 0.8600 143.9000

V 0.5324 0.0000 77.8110 0.0000 1.0600 1.7000 0.6200 140.0000

K 0.3226 0.0000 69.2300 0.0000 0.5700 0.5500 1.5900 122.7000

C 0.4412 -0.1195 105.7050 11.2470 1.1300 1.3800 0.7100 189.9000

W 0.2346 -0.6040 26.0680 15.9610 0.7700 0.7500 1.3200 89.0000

M 0.5324 0.0000 77.8110 0.0000 1.0600 1.7000 0.6200 140.0000

26 KIFGSLAFL 7.4780

V 1.4600 -0.6229 97.7140 8.0790 1.1600 0.7400 0.9800 168.7000

L 1.1046 0.0000 88.6060 0.0000 1.0800 1.6000 0.6600 166.7000

Q 0.4412 -0.1195 105.7050 11.2470 1.1300 1.3800 0.7100 189.9000

A 0.0208 0.0000 3.7620 0.0000 0.5700 0.7500 1.5000 60.1000

G 0.2346 -0.6040 26.0680 15.9610 0.7700 0.7500 1.3200 89.0000

F 1.2906 0.0000 84.5480 0.0000 1.2100 1.3000 0.6800 166.7000

F 0.1744 0.0000 34.7760 0.0000 1.4200 0.8300 0.7000 88.6000

L 0.4412 -0.1195 105.7050 11.2470 1.1300 1.3800 0.7100 189.9000

L 1.2906 0.0000 84.5480 0.0000 1.2100 1.3000 0.6800 166.7000

27 ALVGLFVLL 7.5850

G 0.1744 0.0000 34.7760 0.0000 1.4200 0.8300 0.7000 88.6000

T 1.2906 0.0000 84.5480 0.0000 1.2100 1.3000 0.6800 166.7000

L 0.5324 0.0000 77.8110 0.0000 1.0600 1.7000 0.6200 140.0000

G 0.0208 0.0000 3.7620 0.0000 0.5700 0.7500 1.5000 60.1000

I 1.2906 0.0000 84.5480 0.0000 1.2100 1.3000 0.6800 166.7000

V 0.4412 -0.1195 105.7050 11.2470 1.1300 1.3800 0.7100 189.9000

C 0.5324 0.0000 77.8110 0.0000 1.0600 1.7000 0.6200 140.0000

P 1.2906 0.0000 84.5480 0.0000 1.2100 1.3000 0.6800 166.7000

I 1.2906 0.0000 84.5480 0.0000 1.2100 1.3000 0.6800 166.7000

28 YLSPGPVTV 7.6420

V 0.4534 -0.5896 80.9650 42.7610 0.6900 1.4700 1.0600 193.6000

I 1.2906 0.0000 84.5480 0.0000 1.2100 1.3000 0.6800 166.7000

L 0.2346 -0.6040 26.0680 15.9610 0.7700 0.7500 1.3200 89.0000

G 0.3226 0.0000 69.2300 0.0000 0.5700 0.5500 1.5900 122.7000

V 0.0208 0.0000 3.7620 0.0000 0.5700 0.7500 1.5000 60.1000

L 0.3226 0.0000 69.2300 0.0000 0.5700 0.5500 1.5900 122.7000

L 0.5324 0.0000 77.8110 0.0000 1.0600 1.7000 0.6200 140.0000

L 1.4265 -0.4369 46.7290 16.0490 0.8300 1.1900 1.0700 116.1000

I 0.5324 0.0000 77.8110 0.0000 1.0600 1.7000 0.6200 140.0000

29 GLYSSTVPV 7.6990

V 0.0208 0.0000 3.7620 0.0000 0.5700 0.7500 1.5000 60.1000

T 1.2906 0.0000 84.5480 0.0000 1.2100 1.3000 0.6800 166.7000

W 0.4534 -0.5896 80.9650 42.7610 0.6900 1.4700 1.0600 193.6000

H 0.2346 -0.6040 26.0680 15.9610 0.7700 0.7500 1.3200 89.0000

R 0.2346 -0.6040 26.0680 15.9610 0.7700 0.7500 1.3200 89.0000

Y 1.4265 -0.4369 46.7290 16.0490 0.8300 1.1900 1.0700 116.1000

H 0.5324 0.0000 77.8110 0.0000 1.0600 1.7000 0.6200 140.0000

L 0.3226 0.0000 69.2300 0.0000 0.5700 0.5500 1.5900 122.7000

L 0.5324 0.0000 77.8110 0.0000 1.0600 1.7000 0.6200 140.0000

30 YLYPGPVTA 7.7720

P 0.4534 -0.5896 80.9650 42.7610 0.6900 1.4700 1.0600 193.6000

L 1.2906 0.0000 84.5480 0.0000 1.2100 1.3000 0.6800 166.7000

L 0.4534 -0.5896 80.9650 42.7610 0.6900 1.4700 1.0600 193.6000

P 0.3226 0.0000 69.2300 0.0000 0.5700 0.5500 1.5900 122.7000

I 0.0208 0.0000 3.7620 0.0000 0.5700 0.7500 1.5000 60.1000

F 0.3226 0.0000 69.2300 0.0000 0.5700 0.5500 1.5900 122.7000

F 0.5324 0.0000 77.8110 0.0000 1.0600 1.7000 0.6200 140.0000

C 1.4265 -0.4369 46.7290 16.0490 0.8300 1.1900 1.0700 116.1000

L 0.1744 0.0000 34.7760 0.0000 1.4200 0.8300 0.7000 88.6000

31 YLAPGPVTV 7.8180

T 0.4534 -0.5896 80.9650 42.7610 0.6900 1.4700 1.0600 193.6000

L 1.2906 0.0000 84.5480 0.0000 1.2100 1.3000 0.6800 166.7000

G 0.1744 0.0000 34.7760 0.0000 1.4200 0.8300 0.7000 88.6000

I 0.3226 0.0000 69.2300 0.0000 0.5700 0.5500 1.5900 122.7000

V 0.0208 0.0000 3.7620 0.0000 0.5700 0.7500 1.5000 60.1000

C 0.3226 0.0000 69.2300 0.0000 0.5700 0.5500 1.5900 122.7000

P 0.5324 0.0000 77.8110 0.0000 1.0600 1.7000 0.6200 140.0000

I 1.4265 -0.4369 46.7290 16.0490 0.8300 1.1900 1.0700 116.1000

C 0.5324 0.0000 77.8110 0.0000 1.0600 1.7000 0.6200 140.0000

32 VVLGVVFGI 7.8450

C 0.5324 0.0000 77.8110 0.0000 1.0600 1.7000 0.6200 140.0000

L 0.5324 0.0000 77.8110 0.0000 1.0600 1.7000 0.6200 140.0000

T 1.2906 0.0000 84.5480 0.0000 1.2100 1.3000 0.6800 166.7000

S 0.0208 0.0000 3.7620 0.0000 0.5700 0.7500 1.5000 60.1000

T 0.5324 0.0000 77.8110 0.0000 1.0600 1.7000 0.6200 140.0000

V 0.5324 0.0000 77.8110 0.0000 1.0600 1.7000 0.6200 140.0000

Q 0.4412 -0.1195 105.7050 11.2470 1.1300 1.3800 0.7100 189.9000

L 0.0208 0.0000 3.7620 0.0000 0.5700 0.7500 1.5000 60.1000

V 1.1046 0.0000 88.6060 0.0000 1.0800 1.6000 0.6600 166.7000

33 MMWYWGPSL 7.9210

I 1.0768 -0.3068 70.3631 23.2300 1.4500 1.0500 0.5800 162.9000

L 1.0768 -0.3068 70.3631 23.2300 1.4500 1.0500 0.5800 162.9000

L 0.8364 -0.4310 133.6980 14.8820 1.0800 1.3700 0.7500 227.8000

L 0.4534 -0.5896 80.9650 42.7610 0.6900 1.4700 1.0600 193.6000

C 0.8364 -0.4310 133.6980 14.8820 1.0800 1.3700 0.7500 227.8000

L 0.0208 0.0000 3.7620 0.0000 0.5700 0.7500 1.5000 60.1000

I 0.3226 0.0000 69.2300 0.0000 0.5700 0.5500 1.5900 122.7000

F 0.2346 -0.6040 26.0680 15.9610 0.7700 0.7500 1.3200 89.0000

L 1.2906 0.0000 84.5480 0.0000 1.2100 1.3000 0.6800 166.7000

34 ILAQVPFSV 7.9390

F 1.1046 0.0000 88.6060 0.0000 1.0800 1.6000 0.6600 166.7000

A 1.2906 0.0000 84.5480 0.0000 1.2100 1.3000 0.6800 166.7000

F 0.1744 0.0000 34.7760 0.0000 1.4200 0.8300 0.7000 88.6000

R 1.0036 -0.7211 70.0880 17.8660 1.1100 1.1000 0.8600 143.9000

D 0.5324 0.0000 77.8110 0.0000 1.0600 1.7000 0.6200 140.0000

L 0.3226 0.0000 69.2300 0.0000 0.5700 0.5500 1.5900 122.7000

C 0.4412 -0.1195 105.7050 11.2470 1.1300 1.3800 0.7100 189.9000

I 0.2346 -0.6040 26.0680 15.9610 0.7700 0.7500 1.3200 89.0000

V 0.5324 0.0000 77.8110 0.0000 1.0600 1.7000 0.6200 140.0000

35 FLLSLGIHL 8.0530

F 0.4412 -0.1195 105.7050 11.2470 1.1300 1.3800 0.7100 189.9000

L 1.2906 0.0000 84.5480 0.0000 1.2100 1.3000 0.6800 166.7000

E 1.2906 0.0000 84.5480 0.0000 1.2100 1.3000 0.6800 166.7000

P 0.2346 -0.6040 26.0680 15.9610 0.7700 0.7500 1.3200 89.0000

G 1.2906 0.0000 84.5480 0.0000 1.2100 1.3000 0.6800 166.7000

P 0.0208 0.0000 3.7620 0.0000 0.5700 0.7500 1.5000 60.1000

V 1.1046 0.0000 88.6060 0.0000 1.0800 1.6000 0.6600 166.7000

T 0.8124 -0.7766 82.1700 13.8630 1.0000 0.8700 1.0600 153.2000

A 1.2906 0.0000 84.5480 0.0000 1.2100 1.3000 0.6800 166.7000

36 ILMQVPFSV 8.1250

A 1.1046 0.0000 88.6060 0.0000 1.0800 1.6000 0.6600 166.7000

L 1.2906 0.0000 84.5480 0.0000 1.2100 1.3000 0.6800 166.7000

A 1.0768 -0.3068 70.3631 23.2300 1.4500 1.0500 0.5800 162.9000

K 1.0036 -0.7211 70.0880 17.8660 1.1100 1.1000 0.8600 143.9000

A 0.5324 0.0000 77.8110 0.0000 1.0600 1.7000 0.6200 140.0000

A 0.3226 0.0000 69.2300 0.0000 0.5700 0.5500 1.5900 122.7000

A 0.4412 -0.1195 105.7050 11.2470 1.1300 1.3800 0.7100 189.9000

A 0.2346 -0.6040 26.0680 15.9610 0.7700 0.7500 1.3200 89.0000

A 0.5324 0.0000 77.8110 0.0000 1.0600 1.7000 0.6200 140.0000

37 YLFPGPVTV 8.2370

L 0.4534 -0.5896 80.9650 42.7610 0.6900 1.4700 1.0600 193.6000

M 1.2906 0.0000 84.5480 0.0000 1.2100 1.3000 0.6800 166.7000

A 0.4412 -0.1195 105.7050 11.2470 1.1300 1.3800 0.7100 189.9000

V 0.3226 0.0000 69.2300 0.0000 0.5700 0.5500 1.5900 122.7000

V 0.0208 0.0000 3.7620 0.0000 0.5700 0.7500 1.5000 60.1000

L 0.3226 0.0000 69.2300 0.0000 0.5700 0.5500 1.5900 122.7000

A 0.5324 0.0000 77.8110 0.0000 1.0600 1.7000 0.6200 140.0000

S 1.4265 -0.4369 46.7290 16.0490 0.8300 1.1900 1.0700 116.1000

L 0.5324 0.0000 77.8110 0.0000 1.0600 1.7000 0.6200 140.0000

38 YLMPGPVTA 8.3670

Y 0.4534 -0.5896 80.9650 42.7610 0.6900 1.4700 1.0600 193.6000

V 1.2906 0.0000 84.5480 0.0000 1.2100 1.3000 0.6800 166.7000

I 1.0768 -0.3068 70.3631 23.2300 1.4500 1.0500 0.5800 162.9000

T 0.3226 0.0000 69.2300 0.0000 0.5700 0.5500 1.5900 122.7000

T 0.0208 0.0000 3.7620 0.0000 0.5700 0.7500 1.5000 60.1000

Q 0.3226 0.0000 69.2300 0.0000 0.5700 0.5500 1.5900 122.7000

H 0.5324 0.0000 77.8110 0.0000 1.0600 1.7000 0.6200 140.0000

W 1.4265 -0.4369 46.7290 16.0490 0.8300 1.1900 1.0700 116.1000

L 0.1744 0.0000 34.7760 0.0000 1.4200 0.8300 0.7000 88.6000

39 YLWPGPVTA 8.4950

L 0.4534 -0.5896 80.9650 42.7610 0.6900 1.4700 1.0600 193.6000

L 1.2906 0.0000 84.5480 0.0000 1.2100 1.3000 0.6800 166.7000

C 0.8364 -0.4310 133.6980 14.8820 1.0800 1.3700 0.7500 227.8000

L 0.3226 0.0000 69.2300 0.0000 0.5700 0.5500 1.5900 122.7000

I 0.0208 0.0000 3.7620 0.0000 0.5700 0.7500 1.5000 60.1000

F 0.3226 0.0000 69.2300 0.0000 0.5700 0.5500 1.5900 122.7000

L 0.5324 0.0000 77.8110 0.0000 1.0600 1.7000 0.6200 140.0000

L 1.4265 -0.4369 46.7290 16.0490 0.8300 1.1900 1.0700 116.1000

V 0.1744 0.0000 34.7760 0.0000 1.4200 0.8300 0.7000 88.6000

40 FLDQVPFSV 8.6580

I 0.4412 -0.1195 105.7050 11.2470 1.1300 1.3800 0.7100 189.9000

T 1.2906 0.0000 84.5480 0.0000 1.2100 1.3000 0.6800 166.7000

A 0.6058 -0.9298 37.4170 25.2733 1.0100 0.5400 1.2000 111.1000

Q 1.0036 -0.7211 70.0880 17.8660 1.1100 1.1000 0.8600 143.9000

V 0.5324 0.0000 77.8110 0.0000 1.0600 1.7000 0.6200 140.0000

P 0.3226 0.0000 69.2300 0.0000 0.5700 0.5500 1.5900 122.7000

F 0.4412 -0.1195 105.7050 11.2470 1.1300 1.3800 0.7100 189.9000

S 0.2346 -0.6040 26.0680 15.9610 0.7700 0.7500 1.3200 89.0000

V 0.5324 0.0000 77.8110 0.0000 1.0600 1.7000 0.6200 140.0000

Normalized 3D Data Matrix AV3D(N,L,K):

Statistical indices of Training calculation:

ITERATION (A): 1

No. Sequence Expt. Act Predicted Act Difference

1 VALVGLFVL 5.14800 7.18511 -2.03711

2 GTLVALVGL 5.34200 6.79495 -1.45295

3 LQTTIHDII 5.50100 6.63029 -1.12929

4 SLHVGTQCA 5.84200 6.80358 -0.96158

5 ALPYWNFAT 5.86900 7.54633 -1.67733

6 SLNFMGYVI 5.88100 7.31741 -1.43641

7 NLQSLTNLL 6.00000 6.63664 -0.63664

8 FVTWHRYHL 6.02500 7.46164 -1.43664

9 DPKVKQWPL 6.17600 7.44208 -1.26608

10 ITSQVPFSV 6.19600 7.30458 -1.10858

11 ALAKAAAAI 6.21100 6.70121 -0.49021

12 GLGQVPLIV 6.30100 7.14770 -0.84670

13 MLDLQPETT 6.33500 6.60464 -0.26964

14 LLSSNLSWL 6.34200 6.93500 -0.59300

15 GLACHQLCA 6.38000 6.83141 -0.45141

16 LIGNESFAL 6.41500 6.88925 -0.47425

17 ALAKAAAAV 6.41900 6.75683 -0.33783

18 LLAVGATKV 6.47700 6.79889 -0.32189

19 ALAKAAAAL 6.51100 6.65269 -0.14169

20 WILRGTSFV 6.55600 7.19925 -0.64325

21 IISCTCPTV 6.58000 7.09277 -0.51277

22 FLGGTPVCL 6.62300 7.24555 -0.62255

23 ALIHHNTHL 6.62300 6.81304 -0.19004

24 NLSWLSLDV 6.63900 6.98495 -0.34595

25 YMIMVKCWM 6.66300 7.36390 -0.70090

26 VLQAGFFLL 6.68200 7.19847 -0.51647

27 GTLGIVCPI 6.71400 7.10878 -0.39478

28 VILGVLLLI 6.78500 6.94174 -0.15674

29 VTWHRYHLL 6.79300 7.22784 -0.43484

30 PLLPIFFCL 6.79600 7.70988 -0.91388

31 TLGIVCPIC 6.81500 7.16553 -0.35053

32 CLTSTVQLV 6.83200 6.76432 0.06768

33 ILLLCLIFL 6.84500 7.02401 -0.17901

34 FAFRDLCIV 6.88600 7.27085 -0.38485

35 FLEPGPVTA 6.89800 7.32212 -0.42412

36 ALAKAAAAA 6.94700 6.65516 0.29184

37 LMAVVLASL 6.95400 6.85779 0.09621

38 YVITTQHWL 6.98300 7.14266 -0.15966

39 LLCLIFLLV 6.99600 7.07964 -0.08364

40 ITAQVPFSV 7.02000 7.29486 -0.27486

41 YLEPGPVTL 7.05800 7.31330 -0.25530

42 YTDQVPFSV 7.06600 7.42932 -0.36332

43 NLYVSLLLL 7.11400 7.00641 0.10759

44 ILHNGAYSL 7.12700 7.02561 0.10139

45 SIISAVVGI 7.15900 7.01709 0.14191

46 VVMGTLVAL 7.17400 6.89506 0.27894

47 YLEPGPVTI 7.18700 7.36181 -0.17481

48 GLSRYVARL 7.24800 6.97656 0.27144

49 LLAQFTSAI 7.30100 6.83876 0.46224

50 VLLDYQGML 7.32800 6.92617 0.40183

51 YLEPGPVTV 7.34200 7.41744 -0.07544

52 ILSPFMPLL 7.34700 7.42748 -0.08048

53 YLSPGPVTA 7.38300 7.41466 -0.03166

54 IIDQVPFSV 7.39800 7.36749 0.03051

55 SVYDFFVWL 7.44400 7.72939 -0.28539

56 ITWQVPFSV 7.46300 7.52820 -0.06520

57 ITYQVPFSV 7.48000 7.51985 -0.03985

58 GLYSSTVPV 7.48100 7.27824 0.20276

59 VMGTLVALV 7.55300 6.89506 0.65794

60 LLLCLIFLL 7.58500 6.97550 0.60950

61 SLDDYNHLV 7.58500 6.97840 0.60660

62 VLIQRNPQL 7.64400 7.03819 0.60581

63 SLYADSPSV 7.65800 7.19896 0.45904

64 ILSQVPFSV 7.69900 7.37316 0.32584

65 IMDQVPFSV 7.71900 7.33130 0.38770

66 QLFEDNYAL 7.76400 6.98547 0.77853

67 ALMDKSLHV 7.77000 6.78301 0.98699

68 YAIDLPVSV 7.79600 7.32725 0.46875

69 FVWLHYYSV 7.82400 7.82105 0.00295

70 MLGTHTMEV 7.84500 6.59735 1.24765

71 LLFGYPVYV 7.88600 7.78370 0.10230

72 ILKEPVHGV 7.92100 7.13063 0.79037

73 YLMPGPVTV 7.93200 7.51648 0.41552

74 WLDQVPFSV 7.93900 7.50626 0.43274

75 KTWGQYWQV 7.95500 7.36455 0.59045

76 ALMPLYACI 8.00000 7.23669 0.76331

77 YLAPGPVTA 8.03200 7.40494 0.62706

78 YLYPGPVTV 8.05100 7.73160 0.31940

79 LLMGTLGIV 8.09700 6.74919 1.34781

80 YLWPGPVTV 8.12500 7.73996 0.38504

81 FLLTRILTI 8.14900 6.81069 1.33831

82 GLLGWSPQA 8.23700 7.14137 1.09563

83 ILYQVPFSV 8.31000 7.58843 0.72157

84 GILTVILGV 8.34700 6.88952 1.45748

85 NMVPFFPPV 8.39800 8.10171 0.29629

86 ILDQVPFSV 8.48100 7.31897 1.16203

87 YLFPGPVTA 8.49500 7.63628 0.85872

88 YLDQVPFSV 8.63800 7.49790 1.14010

89 ILFQVPFSV 8.69900 7.59478 1.10422

90 ILWQVPFSV 8.77000 7.59678 1.17322

Correlation Coeffecient and Stand Error

CORL: 0.417601 CORL~2: 0.174390

RES: 0.722306 SEE: 0.076138

Statistical indices of Training calculation:

ITERATION (B): 1

No. Sequence Expt. Act Predicted Act Difference

1 VALVGLFVL 5.14800 6.44770 -1.29970

2 GTLVALVGL 5.34200 6.36428 -1.02228

3 LQTTIHDII 5.50100 6.79458 -1.29358

4 SLHVGTQCA 5.84200 6.40682 -0.56482

5 ALPYWNFAT 5.86900 7.14106 -1.27206

6 SLNFMGYVI 5.88100 6.46935 -0.58835

7 NLQSLTNLL 6.00000 6.55039 -0.55039

8 FVTWHRYHL 6.02500 6.68779 -0.66279

9 DPKVKQWPL 6.17600 6.46839 -0.29239

10 ITSQVPFSV 6.19600 7.54597 -1.34997

11 ALAKAAAAI 6.21100 6.80998 -0.59898

12 GLGQVPLIV 6.30100 7.54469 -1.24369

13 MLDLQPETT 6.33500 6.77413 -0.43913

14 LLSSNLSWL 6.34200 6.17707 0.16493

15 GLACHQLCA 6.38000 6.49805 -0.11805

16 LIGNESFAL 6.41500 6.58565 -0.17065

17 ALAKAAAAV 6.41900 7.02573 -0.60673

18 LLAVGATKV 6.47700 6.90049 -0.42349

19 ALAKAAAAL 6.51100 6.62180 -0.11080

20 WILRGTSFV 6.55600 6.80055 -0.24455

21 IISCTCPTV 6.58000 7.28796 -0.70796

22 FLGGTPVCL 6.62300 6.97598 -0.35298

23 ALIHHNTHL 6.62300 6.69577 -0.07277

24 NLSWLSLDV 6.63900 6.93881 -0.29981

25 YMIMVKCWM 6.66300 6.73528 -0.07228

26 VLQAGFFLL 6.68200 7.11377 -0.43177

27 GTLGIVCPI 6.71400 6.34605 0.36795

28 VILGVLLLI 6.78500 7.22056 -0.43556

29 VTWHRYHLL 6.79300 7.34503 -0.55203

30 PLLPIFFCL 6.79600 6.89628 -0.10028

31 TLGIVCPIC 6.81500 7.03414 -0.21914

32 CLTSTVQLV 6.83200 6.97876 -0.14676

33 ILLLCLIFL 6.84500 6.29066 0.55434

34 FAFRDLCIV 6.88600 7.60287 -0.71687

35 FLEPGPVTA 6.89800 6.98612 -0.08812

36 ALAKAAAAA 6.94700 6.63136 0.31564

37 LMAVVLASL 6.95400 6.70976 0.24424

38 YVITTQHWL 6.98300 6.59492 0.38808

39 LLCLIFLLV 6.99600 7.60894 -0.61294

40 ITAQVPFSV 7.02000 7.52620 -0.50620

41 YLEPGPVTL 7.05800 6.96930 0.08870

42 YTDQVPFSV 7.06600 7.64020 -0.57420

43 NLYVSLLLL 7.11400 7.04120 0.07280

44 ILHNGAYSL 7.12700 6.78481 0.34219

45 SIISAVVGI 7.15900 7.14403 0.01497

46 VVMGTLVAL 7.17400 6.84399 0.33001

47 YLEPGPVTI 7.18700 7.15749 0.02951

48 GLSRYVARL 7.24800 7.27402 -0.02602

49 LLAQFTSAI 7.30100 7.19189 0.10911

50 VLLDYQGML 7.32800 7.19871 0.12929

51 YLEPGPVTV 7.34200 7.37323 -0.03123

52 ILSPFMPLL 7.34700 6.90885 0.43815

53 YLSPGPVTA 7.38300 7.18004 0.20296

54 IIDQVPFSV 7.39800 7.67485 -0.27685

55 SVYDFFVWL 7.44400 7.61794 -0.17394

56 ITWQVPFSV 7.46300 8.00085 -0.53785

57 ITYQVPFSV 7.48000 7.98385 -0.50385

58 GLYSSTVPV 7.48100 6.85957 0.62143

59 VMGTLVALV 7.55300 7.44464 0.10836

60 LLLCLIFLL 7.58500 6.64083 0.94417

61 SLDDYNHLV 7.58500 7.52284 0.06216

62 VLIQRNPQL 7.64400 6.97816 0.66584

63 SLYADSPSV 7.65800 7.44833 0.20967

64 ILSQVPFSV 7.69900 7.68601 0.01299

65 IMDQVPFSV 7.71900 7.60095 0.11805

66 QLFEDNYAL 7.76400 7.20054 0.56346

67 ALMDKSLHV 7.77000 7.03683 0.73317

68 YAIDLPVSV 7.79600 7.78856 0.00744

69 FVWLHYYSV 7.82400 8.42445 -0.60045

70 MLGTHTMEV 7.84500 7.38258 0.46242

71 LLFGYPVYV 7.88600 7.76775 0.11825

72 ILKEPVHGV 7.92100 7.83323 0.08777

73 YLMPGPVTV 7.93200 7.57470 0.35730

74 WLDQVPFSV 7.93900 7.78979 0.14921

75 KTWGQYWQV 7.95500 7.73107 0.22393

76 ALMPLYACI 8.00000 6.69551 1.30449

77 YLAPGPVTA 8.03200 7.16027 0.87173

78 YLYPGPVTV 8.05100 8.01229 0.03871

79 LLMGTLGIV 8.09700 6.79829 1.29871

80 YLWPGPVTV 8.12500 8.02929 0.09571

81 FLLTRILTI 8.14900 7.39380 0.75520

82 GLLGWSPQA 8.23700 7.17306 1.06394

83 ILYQVPFSV 8.31000 8.12389 0.18611

84 GILTVILGV 8.34700 7.46946 0.87754

85 NMVPFFPPV 8.39800 7.24721 1.15079

86 ILDQVPFSV 8.48100 7.57578 0.90522

87 YLFPGPVTA 8.49500 7.63085 0.86415

88 YLDQVPFSV 8.63800 7.78024 0.85776

89 ILFQVPFSV 8.69900 8.13682 0.56218

90 ILWQVPFSV 8.77000 8.14089 0.62911

Correlation Coeffecient and Stand Error

CORL: 0.669624 CORL~2: 0.448396

RES: 0.591189 SEE: 0.062317

Statistical indices of Training calculation:

ITERATION (A): 2

No. Sequence Expt. Act Predicted Act Difference

1 VALVGLFVL 5.14800 6.39308 -1.24508

2 GTLVALVGL 5.34200 6.43685 -1.09485

3 LQTTIHDII 5.50100 6.57457 -1.07357

4 SLHVGTQCA 5.84200 5.71916 0.12284

5 ALPYWNFAT 5.86900 6.06610 -0.19710

6 SLNFMGYVI 5.88100 6.08484 -0.20384

7 NLQSLTNLL 6.00000 6.37476 -0.37476

8 FVTWHRYHL 6.02500 6.39874 -0.37374

9 DPKVKQWPL 6.17600 6.14072 0.03528

10 ITSQVPFSV 6.19600 6.99482 -0.79882

11 ALAKAAAAI 6.21100 7.00644 -0.79544

12 GLGQVPLIV 6.30100 6.79525 -0.49425

13 MLDLQPETT 6.33500 6.13044 0.20456

14 LLSSNLSWL 6.34200 5.86504 0.47696

15 GLACHQLCA 6.38000 6.20062 0.17938

16 LIGNESFAL 6.41500 6.52928 -0.11428

17 ALAKAAAAV 6.41900 7.11030 -0.69130

18 LLAVGATKV 6.47700 6.85258 -0.37558

19 ALAKAAAAL 6.51100 6.86059 -0.34959

20 WILRGTSFV 6.55600 6.37208 0.18392

21 IISCTCPTV 6.58000 7.09622 -0.51622

22 FLGGTPVCL 6.62300 6.55906 0.06394

23 ALIHHNTHL 6.62300 6.78256 -0.15956

24 NLSWLSLDV 6.63900 6.53648 0.10252

25 YMIMVKCWM 6.66300 6.90648 -0.24348

26 VLQAGFFLL 6.68200 6.95447 -0.27247

27 GTLGIVCPI 6.71400 6.70005 0.01395

28 VILGVLLLI 6.78500 7.49901 -0.71401

29 VTWHRYHLL 6.79300 7.27470 -0.48170

30 PLLPIFFCL 6.79600 7.31561 -0.51961

31 TLGIVCPIC 6.81500 6.43220 0.38280

32 CLTSTVQLV 6.83200 6.44594 0.38606

33 ILLLCLIFL 6.84500 6.35519 0.48981

34 FAFRDLCIV 6.88600 7.69582 -0.80982

35 FLEPGPVTA 6.89800 7.11259 -0.21459

36 ALAKAAAAA 6.94700 6.71839 0.22861

37 LMAVVLASL 6.95400 7.29144 -0.33744

38 YVITTQHWL 6.98300 6.46215 0.52085

39 LLCLIFLLV 6.99600 7.65145 -0.65545

40 ITAQVPFSV 7.02000 7.18586 -0.16586

41 YLEPGPVTL 7.05800 7.26154 -0.20354

42 YTDQVPFSV 7.06600 7.24387 -0.17787

43 NLYVSLLLL 7.11400 6.97971 0.13429

44 ILHNGAYSL 7.12700 6.98872 0.13828

45 SIISAVVGI 7.15900 7.58781 -0.42881

46 VVMGTLVAL 7.17400 7.03664 0.13736

47 YLEPGPVTI 7.18700 7.40739 -0.22039

48 GLSRYVARL 7.24800 7.44307 -0.19507

49 LLAQFTSAI 7.30100 7.31628 -0.01528

50 VLLDYQGML 7.32800 7.38997 -0.06197

51 YLEPGPVTV 7.34200 7.51125 -0.16925

52 ILSPFMPLL 7.34700 7.27533 0.07167

53 YLSPGPVTA 7.38300 7.07504 0.30796

54 IIDQVPFSV 7.39800 7.54170 -0.14370

55 SVYDFFVWL 7.44400 7.87463 -0.43063

56 ITWQVPFSV 7.46300 7.83292 -0.36992

57 ITYQVPFSV 7.48000 7.82645 -0.34645

58 GLYSSTVPV 7.48100 7.08205 0.39895

59 VMGTLVALV 7.55300 7.40874 0.14426

60 LLLCLIFLL 7.58500 6.94280 0.64220

61 SLDDYNHLV 7.58500 7.22632 0.35868

62 VLIQRNPQL 7.64400 6.95506 0.68894

63 SLYADSPSV 7.65800 7.44477 0.21323

64 ILSQVPFSV 7.69900 7.49086 0.20814

65 IMDQVPFSV 7.71900 7.59510 0.12390

66 QLFEDNYAL 7.76400 7.14297 0.62103

67 ALMDKSLHV 7.77000 7.10911 0.66089

68 YAIDLPVSV 7.79600 7.95580 -0.15980

69 FVWLHYYSV 7.82400 8.80447 -0.98047

70 MLGTHTMEV 7.84500 7.02976 0.81524

71 LLFGYPVYV 7.88600 7.78997 0.09603

72 ILKEPVHGV 7.92100 7.56073 0.36027

73 YLMPGPVTV 7.93200 7.86224 0.06976

74 WLDQVPFSV 7.93900 7.74354 0.19546

75 KTWGQYWQV 7.95500 7.74297 0.21203

76 ALMPLYACI 8.00000 7.43879 0.56121

77 YLAPGPVTA 8.03200 7.26608 0.76592

78 YLYPGPVTV 8.05100 8.29858 -0.24758

79 LLMGTLGIV 8.09700 6.88159 1.21541

80 YLWPGPVTV 8.12500 8.30505 -0.18005

81 FLLTRILTI 8.14900 8.05804 0.09096

82 GLLGWSPQA 8.23700 7.31468 0.92232

83 ILYQVPFSV 8.31000 8.32249 -0.01249

84 GILTVILGV 8.34700 7.87321 0.47379

85 NMVPFFPPV 8.39800 8.14009 0.25791

86 ILDQVPFSV 8.48100 7.46492 1.01608

87 YLFPGPVTA 8.49500 7.89464 0.60036

88 YLDQVPFSV 8.63800 7.73990 0.89810

89 ILFQVPFSV 8.69900 8.31045 0.38855

90 ILWQVPFSV 8.77000 8.32895 0.44105

Correlation Coeffecient and Stand Error

CORL: 0.787187 CORL~2: 0.619663

RES: 0.490244 SEE: 0.051676

Statistical indices of Training calculation:

ITERATION (B): 2

No. Sequence Expt. Act Predicted Act Difference

1 VALVGLFVL 5.14800 6.29328 -1.14528

2 GTLVALVGL 5.34200 5.94486 -0.60286

3 LQTTIHDII 5.50100 6.52429 -1.02329

4 SLHVGTQCA 5.84200 5.76366 0.07834

5 ALPYWNFAT 5.86900 5.97674 -0.10774

6 SLNFMGYVI 5.88100 5.92624 -0.04524

7 NLQSLTNLL 6.00000 6.58571 -0.58571

8 FVTWHRYHL 6.02500 6.24471 -0.21971

9 DPKVKQWPL 6.17600 6.21236 -0.03636

10 ITSQVPFSV 6.19600 6.64373 -0.44773

11 ALAKAAAAI 6.21100 7.07080 -0.85980

12 GLGQVPLIV 6.30100 6.79103 -0.49003

13 MLDLQPETT 6.33500 6.10284 0.23216

14 LLSSNLSWL 6.34200 5.82922 0.51278

15 GLACHQLCA 6.38000 6.20549 0.17451

16 LIGNESFAL 6.41500 6.86147 -0.44647

17 ALAKAAAAV 6.41900 7.16633 -0.74733

18 LLAVGATKV 6.47700 6.65349 -0.17649

19 ALAKAAAAL 6.51100 6.93665 -0.42565

20 WILRGTSFV 6.55600 6.50132 0.05468

21 IISCTCPTV 6.58000 7.06556 -0.48556

22 FLGGTPVCL 6.62300 6.61350 0.00950

23 ALIHHNTHL 6.62300 6.78580 -0.16280

24 NLSWLSLDV 6.63900 6.26863 0.37037

25 YMIMVKCWM 6.66300 6.86093 -0.19793

26 VLQAGFFLL 6.68200 6.83453 -0.15253

27 GTLGIVCPI 6.71400 6.34102 0.37298

28 VILGVLLLI 6.78500 7.64883 -0.86383

29 VTWHRYHLL 6.79300 6.65058 0.14242

30 PLLPIFFCL 6.79600 7.15876 -0.36276

31 TLGIVCPIC 6.81500 6.31550 0.49950

32 CLTSTVQLV 6.83200 6.39102 0.44098

33 ILLLCLIFL 6.84500 6.25580 0.58920

34 FAFRDLCIV 6.88600 7.67753 -0.79153

35 FLEPGPVTA 6.89800 7.05172 -0.15372

36 ALAKAAAAA 6.94700 6.80585 0.14115

37 LMAVVLASL 6.95400 7.27688 -0.32288

38 YVITTQHWL 6.98300 6.74747 0.23553

39 LLCLIFLLV 6.99600 7.38467 -0.38867

40 ITAQVPFSV 7.02000 6.85415 0.16585

41 YLEPGPVTL 7.05800 7.18806 -0.13006

42 YTDQVPFSV 7.06600 6.84061 0.22539

43 NLYVSLLLL 7.11400 6.94422 0.16978

44 ILHNGAYSL 7.12700 7.22254 -0.09554

45 SIISAVVGI 7.15900 7.77304 -0.61404

46 VVMGTLVAL 7.17400 7.31466 -0.14066

47 YLEPGPVTI 7.18700 7.32221 -0.13521

48 GLSRYVARL 7.24800 7.44159 -0.19359

49 LLAQFTSAI 7.30100 7.43416 -0.13316

50 VLLDYQGML 7.32800 7.43916 -0.11116

51 YLEPGPVTV 7.34200 7.41774 -0.07574

52 ILSPFMPLL 7.34700 7.11324 0.23376

53 YLSPGPVTA 7.38300 7.00847 0.37453

54 IIDQVPFSV 7.39800 7.70841 -0.31041

55 SVYDFFVWL 7.44400 7.94277 -0.49877

56 ITWQVPFSV 7.46300 7.56684 -0.10384

57 ITYQVPFSV 7.48000 7.55972 -0.07972

58 GLYSSTVPV 7.48100 7.43853 0.04247

59 VMGTLVALV 7.55300 7.60609 -0.05309

60 LLLCLIFLL 7.58500 6.93823 0.64677

61 SLDDYNHLV 7.58500 7.35327 0.23173

62 VLIQRNPQL 7.64400 7.09553 0.54847

63 SLYADSPSV 7.65800 7.61711 0.04089

64 ILSQVPFSV 7.69900 7.59044 0.10856

65 IMDQVPFSV 7.71900 7.81032 -0.09132

66 QLFEDNYAL 7.76400 7.49336 0.27064

67 ALMDKSLHV 7.77000 7.31255 0.45745

68 YAIDLPVSV 7.79600 7.95774 -0.16174

69 FVWLHYYSV 7.82400 8.78931 -0.96531

70 MLGTHTMEV 7.84500 7.38661 0.45839

71 LLFGYPVYV 7.88600 7.88953 -0.00353

72 ILKEPVHGV 7.92100 7.53419 0.38681

73 YLMPGPVTV 7.93200 7.80434 0.12766

74 WLDQVPFSV 7.93900 7.79029 0.14871

75 KTWGQYWQV 7.95500 7.39990 0.55510

76 ALMPLYACI 8.00000 7.16639 0.83361

77 YLAPGPVTA 8.03200 7.21888 0.81312

78 YLYPGPVTV 8.05100 8.28493 -0.23393

79 LLMGTLGIV 8.09700 6.93221 1.16479

80 YLWPGPVTV 8.12500 8.29206 -0.16706

81 FLLTRILTI 8.14900 8.21693 -0.06793

82 GLLGWSPQA 8.23700 7.55682 0.68018

83 ILYQVPFSV 8.31000 8.50643 -0.19643

84 GILTVILGV 8.34700 8.15481 0.19219

85 NMVPFFPPV 8.39800 8.00269 0.39531

86 ILDQVPFSV 8.48100 7.56187 0.91913

87 YLFPGPVTA 8.49500 7.91120 0.58380

88 YLDQVPFSV 8.63800 7.78732 0.85068

89 ILFQVPFSV 8.69900 8.49318 0.20582

90 ILWQVPFSV 8.77000 8.51355 0.25645

Correlation Coeffecient and Stand Error

CORL: 0.823347 CORL~2: 0.677901

RES: 0.451452 SEE: 0.047587

Statistical indices of Training calculation:

ITERATION (A): 3

No. Sequence Expt. Act Predicted Act Difference

1 VALVGLFVL 5.14800 6.30640 -1.15840

2 GTLVALVGL 5.34200 6.07283 -0.73083

3 LQTTIHDII 5.50100 6.52946 -1.02846

4 SLHVGTQCA 5.84200 5.72151 0.12049

5 ALPYWNFAT 5.86900 5.98612 -0.11712

6 SLNFMGYVI 5.88100 5.98252 -0.10152

7 NLQSLTNLL 6.00000 6.67071 -0.67071

8 FVTWHRYHL 6.02500 6.24660 -0.22160

9 DPKVKQWPL 6.17600 6.26664 -0.09064

10 ITSQVPFSV 6.19600 6.58808 -0.39208

11 ALAKAAAAI 6.21100 7.04891 -0.83791

12 GLGQVPLIV 6.30100 6.65724 -0.35624

13 MLDLQPETT 6.33500 6.12883 0.20617

14 LLSSNLSWL 6.34200 5.87530 0.46670

15 GLACHQLCA 6.38000 6.15651 0.22349

16 LIGNESFAL 6.41500 6.90814 -0.49314

17 ALAKAAAAV 6.41900 7.09600 -0.67700

18 LLAVGATKV 6.47700 6.58578 -0.10878

19 ALAKAAAAL 6.51100 6.94233 -0.43133

20 WILRGTSFV 6.55600 6.42944 0.12656

21 IISCTCPTV 6.58000 6.96954 -0.38954

22 FLGGTPVCL 6.62300 6.66929 -0.04629

23 ALIHHNTHL 6.62300 6.85497 -0.23197

24 NLSWLSLDV 6.63900 6.22216 0.41684

25 YMIMVKCWM 6.66300 6.94660 -0.28360

26 VLQAGFFLL 6.68200 6.83414 -0.15214

27 GTLGIVCPI 6.71400 6.46975 0.24425

28 VILGVLLLI 6.78500 7.68042 -0.89542

29 VTWHRYHLL 6.79300 6.69819 0.09481

30 PLLPIFFCL 6.79600 7.26579 -0.46979

31 TLGIVCPIC 6.81500 6.25002 0.56498

32 CLTSTVQLV 6.83200 6.41729 0.41471

33 ILLLCLIFL 6.84500 6.33350 0.51150

34 FAFRDLCIV 6.88600 7.55710 -0.67110

35 FLEPGPVTA 6.89800 7.03068 -0.13268

36 ALAKAAAAA 6.94700 6.74537 0.20163

37 LMAVVLASL 6.95400 7.41489 -0.46089

38 YVITTQHWL 6.98300 6.72484 0.25816

39 LLCLIFLLV 6.99600 7.33267 -0.33667

40 ITAQVPFSV 7.02000 6.81380 0.20620

41 YLEPGPVTL 7.05800 7.24140 -0.18340

42 YTDQVPFSV 7.06600 6.81747 0.24853

43 NLYVSLLLL 7.11400 6.98640 0.12760

44 ILHNGAYSL 7.12700 7.28570 -0.15870

45 SIISAVVGI 7.15900 7.82744 -0.66844

46 VVMGTLVAL 7.17400 7.42115 -0.24715

47 YLEPGPVTI 7.18700 7.34798 -0.16098

48 GLSRYVARL 7.24800 7.41038 -0.16238

49 LLAQFTSAI 7.30100 7.46004 -0.15904

50 VLLDYQGML 7.32800 7.45739 -0.12939

51 YLEPGPVTV 7.34200 7.39507 -0.05307

52 ILSPFMPLL 7.34700 7.15477 0.19223

53 YLSPGPVTA 7.38300 6.92618 0.45682

54 IIDQVPFSV 7.39800 7.65312 -0.25512

55 SVYDFFVWL 7.44400 7.89783 -0.45383

56 ITWQVPFSV 7.46300 7.54684 -0.08384

57 ITYQVPFSV 7.48000 7.54808 -0.06808

58 GLYSSTVPV 7.48100 7.46445 0.01655

59 VMGTLVALV 7.55300 7.52738 0.02562

60 LLLCLIFLL 7.58500 7.02236 0.56264

61 SLDDYNHLV 7.58500 7.29335 0.29165

62 VLIQRNPQL 7.64400 7.12734 0.51666

63 SLYADSPSV 7.65800 7.62801 0.02999

64 ILSQVPFSV 7.69900 7.53463 0.16437

65 IMDQVPFSV 7.71900 7.82910 -0.11010

66 QLFEDNYAL 7.76400 7.52093 0.24307

67 ALMDKSLHV 7.77000 7.36255 0.40745

68 YAIDLPVSV 7.79600 7.91777 -0.12177

69 FVWLHYYSV 7.82400 8.69543 -0.87143

70 MLGTHTMEV 7.84500 7.28210 0.56290

71 LLFGYPVYV 7.88600 7.90166 -0.01566

72 ILKEPVHGV 7.92100 7.51282 0.40818

73 YLMPGPVTV 7.93200 7.79432 0.13768

74 WLDQVPFSV 7.93900 7.76349 0.17551

75 KTWGQYWQV 7.95500 7.44606 0.50894

76 ALMPLYACI 8.00000 7.29974 0.70026

77 YLAPGPVTA 8.03200 7.15190 0.88010

78 YLYPGPVTV 8.05100 8.23681 -0.18581

79 LLMGTLGIV 8.09700 7.03239 1.06461

80 YLWPGPVTV 8.12500 8.23557 -0.11057

81 FLLTRILTI 8.14900 8.23512 -0.08612

82 GLLGWSPQA 8.23700 7.57522 0.66178

83 ILYQVPFSV 8.31000 8.49463 -0.18463

84 GILTVILGV 8.34700 8.12799 0.21901

85 NMVPFFPPV 8.39800 8.06438 0.33362

86 ILDQVPFSV 8.48100 7.53670 0.94430

87 YLFPGPVTA 8.49500 7.85327 0.64173

88 YLDQVPFSV 8.63800 7.76401 0.87399

89 ILFQVPFSV 8.69900 8.46172 0.23728

90 ILWQVPFSV 8.77000 8.49338 0.27662

Correlation Coeffecient and Stand Error

CORL: 0.826572 CORL~2: 0.683221

RES: 0.447412 SEE: 0.047161

Statistical indices of Training calculation:

ITERATION (B): 3

No. Sequence Expt. Act Predicted Act Difference

1 VALVGLFVL 5.14800 6.26637 -1.11837

2 GTLVALVGL 5.34200 5.97594 -0.63394

3 LQTTIHDII 5.50100 6.51685 -1.01585

4 SLHVGTQCA 5.84200 5.69168 0.15032

5 ALPYWNFAT 5.86900 6.04016 -0.17116

6 SLNFMGYVI 5.88100 5.92498 -0.04398

7 NLQSLTNLL 6.00000 6.67290 -0.67290

8 FVTWHRYHL 6.02500 6.24999 -0.22499

9 DPKVKQWPL 6.17600 6.22238 -0.04638

10 ITSQVPFSV 6.19600 6.54060 -0.34460

11 ALAKAAAAI 6.21100 7.03592 -0.82492

12 GLGQVPLIV 6.30100 6.61775 -0.31675

13 MLDLQPETT 6.33500 6.19430 0.14070

14 LLSSNLSWL 6.34200 5.84542 0.49658

15 GLACHQLCA 6.38000 6.12864 0.25136

16 LIGNESFAL 6.41500 6.93148 -0.51648

17 ALAKAAAAV 6.41900 7.07946 -0.66046

18 LLAVGATKV 6.47700 6.54128 -0.06428

19 ALAKAAAAL 6.51100 6.93737 -0.42637

20 WILRGTSFV 6.55600 6.44937 0.10663

21 IISCTCPTV 6.58000 6.94046 -0.36046

22 FLGGTPVCL 6.62300 6.69437 -0.07137

23 ALIHHNTHL 6.62300 6.84812 -0.22512

24 NLSWLSLDV 6.63900 6.15587 0.48313

25 YMIMVKCWM 6.66300 6.95857 -0.29557

26 VLQAGFFLL 6.68200 6.80084 -0.11884

27 GTLGIVCPI 6.71400 6.39165 0.32235

28 VILGVLLLI 6.78500 7.70533 -0.92033

29 VTWHRYHLL 6.79300 6.61584 0.17716

30 PLLPIFFCL 6.79600 7.24130 -0.44530

31 TLGIVCPIC 6.81500 6.18877 0.62623

32 CLTSTVQLV 6.83200 6.38179 0.45021

33 ILLLCLIFL 6.84500 6.31188 0.53312

34 FAFRDLCIV 6.88600 7.56453 -0.67853

35 FLEPGPVTA 6.89800 7.07110 -0.17310

36 ALAKAAAAA 6.94700 6.75525 0.19175

37 LMAVVLASL 6.95400 7.42618 -0.47218

38 YVITTQHWL 6.98300 6.76370 0.21930

39 LLCLIFLLV 6.99600 7.29179 -0.29579

40 ITAQVPFSV 7.02000 6.76936 0.25064

41 YLEPGPVTL 7.05800 7.26912 -0.21112

42 YTDQVPFSV 7.06600 6.80537 0.26063

43 NLYVSLLLL 7.11400 6.93971 0.17429

44 ILHNGAYSL 7.12700 7.30038 -0.17338

45 SIISAVVGI 7.15900 7.81956 -0.66056

46 VVMGTLVAL 7.17400 7.43615 -0.26215

47 YLEPGPVTI 7.18700 7.36767 -0.18067

48 GLSRYVARL 7.24800 7.41078 -0.16278

49 LLAQFTSAI 7.30100 7.49601 -0.19501

50 VLLDYQGML 7.32800 7.49860 -0.17060

51 YLEPGPVTV 7.34200 7.41121 -0.06921

52 ILSPFMPLL 7.34700 7.16432 0.18268

53 YLSPGPVTA 7.38300 6.96716 0.41584

54 IIDQVPFSV 7.39800 7.67197 -0.27397

55 SVYDFFVWL 7.44400 7.89230 -0.44830

56 ITWQVPFSV 7.46300 7.51225 -0.04925

57 ITYQVPFSV 7.48000 7.51351 -0.03351

58 GLYSSTVPV 7.48100 7.45094 0.03006

59 VMGTLVALV 7.55300 7.55278 0.00022

60 LLLCLIFLL 7.58500 7.01208 0.57292

61 SLDDYNHLV 7.58500 7.30197 0.28303

62 VLIQRNPQL 7.64400 7.14217 0.50183

63 SLYADSPSV 7.65800 7.60478 0.05322

64 ILSQVPFSV 7.69900 7.54618 0.15282

65 IMDQVPFSV 7.71900 7.85892 -0.13992

66 QLFEDNYAL 7.76400 7.52544 0.23856

67 ALMDKSLHV 7.77000 7.36129 0.40871

68 YAIDLPVSV 7.79600 7.95655 -0.16055

69 FVWLHYYSV 7.82400 8.71728 -0.89328

70 MLGTHTMEV 7.84500 7.32123 0.52377

71 LLFGYPVYV 7.88600 7.92517 -0.03917

72 ILKEPVHGV 7.92100 7.51027 0.41073

73 YLMPGPVTV 7.93200 7.81584 0.11616

74 WLDQVPFSV 7.93900 7.81035 0.12865

75 KTWGQYWQV 7.95500 7.36481 0.59019

76 ALMPLYACI 8.00000 7.25274 0.74726

77 YLAPGPVTA 8.03200 7.19591 0.83609

78 YLYPGPVTV 8.05100 8.26427 -0.21327

79 LLMGTLGIV 8.09700 7.00789 1.08911

80 YLWPGPVTV 8.12500 8.26301 -0.13801

81 FLLTRILTI 8.14900 8.29006 -0.14106

82 GLLGWSPQA 8.23700 7.62226 0.61474

83 ILYQVPFSV 8.31000 8.51909 -0.20909

84 GILTVILGV 8.34700 8.13303 0.21397

85 NMVPFFPPV 8.39800 8.04732 0.35068

86 ILDQVPFSV 8.48100 7.54828 0.93272

87 YLFPGPVTA 8.49500 7.90671 0.58829

88 YLDQVPFSV 8.63800 7.81096 0.82704

89 ILFQVPFSV 8.69900 8.48573 0.21327

90 ILWQVPFSV 8.77000 8.51783 0.25217

Correlation Coeffecient and Stand Error

CORL: 0.828068 CORL~2: 0.685697

RES: 0.445995 SEE: 0.047012

Statistical indices of Training calculation:

ITERATION (A): 4

No. Sequence Expt. Act Predicted Act Difference

1 VALVGLFVL 5.14800 6.26410 -1.11610

2 GTLVALVGL 5.34200 6.03597 -0.69397

3 LQTTIHDII 5.50100 6.53038 -1.02938

4 SLHVGTQCA 5.84200 5.68260 0.15940

5 ALPYWNFAT 5.86900 6.06620 -0.19720

6 SLNFMGYVI 5.88100 5.98036 -0.09936

7 NLQSLTNLL 6.00000 6.71189 -0.71189

8 FVTWHRYHL 6.02500 6.28134 -0.25634

9 DPKVKQWPL 6.17600 6.23300 -0.05700

10 ITSQVPFSV 6.19600 6.55529 -0.35929

11 ALAKAAAAI 6.21100 7.02387 -0.81287

12 GLGQVPLIV 6.30100 6.59599 -0.29499

13 MLDLQPETT 6.33500 6.20979 0.12521

14 LLSSNLSWL 6.34200 5.89487 0.44713

15 GLACHQLCA 6.38000 6.08537 0.29463

16 LIGNESFAL 6.41500 6.96291 -0.54791

17 ALAKAAAAV 6.41900 7.04836 -0.62936

18 LLAVGATKV 6.47700 6.53498 -0.05798

19 ALAKAAAAL 6.51100 6.92944 -0.41844

20 WILRGTSFV 6.55600 6.46479 0.09121

21 IISCTCPTV 6.58000 6.92008 -0.34008

22 FLGGTPVCL 6.62300 6.71798 -0.09498

23 ALIHHNTHL 6.62300 6.88373 -0.26073

24 NLSWLSLDV 6.63900 6.17814 0.46086

25 YMIMVKCWM 6.66300 6.98400 -0.32100

26 VLQAGFFLL 6.68200 6.82340 -0.14140

27 GTLGIVCPI 6.71400 6.47610 0.23790

28 VILGVLLLI 6.78500 7.71379 -0.92879

29 VTWHRYHLL 6.79300 6.65580 0.13720

30 PLLPIFFCL 6.79600 7.27117 -0.47517

31 TLGIVCPIC 6.81500 6.20041 0.61459

32 CLTSTVQLV 6.83200 6.42638 0.40562

33 ILLLCLIFL 6.84500 6.36819 0.47681

34 FAFRDLCIV 6.88600 7.48354 -0.59754

35 FLEPGPVTA 6.89800 7.03275 -0.13475

36 ALAKAAAAA 6.94700 6.71189 0.23511

37 LMAVVLASL 6.95400 7.43385 -0.47985

38 YVITTQHWL 6.98300 6.77555 0.20745

39 LLCLIFLLV 6.99600 7.28601 -0.29001

40 ITAQVPFSV 7.02000 6.77016 0.24984

41 YLEPGPVTL 7.05800 7.27567 -0.21767

42 YTDQVPFSV 7.06600 6.82209 0.24391

43 NLYVSLLLL 7.11400 6.96743 0.14657

44 ILHNGAYSL 7.12700 7.31524 -0.18824

45 SIISAVVGI 7.15900 7.82403 -0.66503

46 VVMGTLVAL 7.17400 7.47416 -0.30016

47 YLEPGPVTI 7.18700 7.37009 -0.18309

48 GLSRYVARL 7.24800 7.40910 -0.16110

49 LLAQFTSAI 7.30100 7.50653 -0.20553

50 VLLDYQGML 7.32800 7.52152 -0.19352

51 YLEPGPVTV 7.34200 7.39458 -0.05258

52 ILSPFMPLL 7.34700 7.17822 0.16878

53 YLSPGPVTA 7.38300 6.93509 0.44791

54 IIDQVPFSV 7.39800 7.65310 -0.25510

55 SVYDFFVWL 7.44400 7.87786 -0.43386

56 ITWQVPFSV 7.46300 7.51447 -0.05147

57 ITYQVPFSV 7.48000 7.53132 -0.05132

58 GLYSSTVPV 7.48100 7.47705 0.00395

59 VMGTLVALV 7.55300 7.50598 0.04702

60 LLLCLIFLL 7.58500 7.03591 0.54909

61 SLDDYNHLV 7.58500 7.29262 0.29238

62 VLIQRNPQL 7.64400 7.16028 0.48372

63 SLYADSPSV 7.65800 7.62203 0.03597

64 ILSQVPFSV 7.69900 7.52829 0.17071

65 IMDQVPFSV 7.71900 7.83874 -0.11974

66 QLFEDNYAL 7.76400 7.55283 0.21117

67 ALMDKSLHV 7.77000 7.36697 0.40303

68 YAIDLPVSV 7.79600 7.91958 -0.12358

69 FVWLHYYSV 7.82400 8.65487 -0.83087

70 MLGTHTMEV 7.84500 7.29801 0.54699

71 LLFGYPVYV 7.88600 7.90502 -0.01902

72 ILKEPVHGV 7.92100 7.49912 0.42188

73 YLMPGPVTV 7.93200 7.80298 0.12902

74 WLDQVPFSV 7.93900 7.78706 0.15194

75 KTWGQYWQV 7.95500 7.38140 0.57360

76 ALMPLYACI 8.00000 7.28398 0.71602

77 YLAPGPVTA 8.03200 7.14996 0.88204

78 YLYPGPVTV 8.05100 8.24759 -0.19659

79 LLMGTLGIV 8.09700 7.03670 1.06030

80 YLWPGPVTV 8.12500 8.23075 -0.10575

81 FLLTRILTI 8.14900 8.26173 -0.11273

82 GLLGWSPQA 8.23700 7.59628 0.64072

83 ILYQVPFSV 8.31000 8.50432 -0.19432

84 GILTVILGV 8.34700 8.10616 0.24084

85 NMVPFFPPV 8.39800 8.04732 0.35068

86 ILDQVPFSV 8.48100 7.53458 0.94642

87 YLFPGPVTA 8.49500 7.85792 0.63708

88 YLDQVPFSV 8.63800 7.79509 0.84291

89 ILFQVPFSV 8.69900 8.45112 0.24788

90 ILWQVPFSV 8.77000 8.48748 0.28252

Correlation Coeffecient and Stand Error

CORL: 0.828614 CORL~2: 0.686601

RES: 0.445019 SEE: 0.046909

Statistical indices of Training calculation:

ITERATION (B): 4

No. Sequence Expt. Act Predicted Act Difference

1 VALVGLFVL 5.14800 6.22875 -1.08075

2 GTLVALVGL 5.34200 5.96436 -0.62236

3 LQTTIHDII 5.50100 6.50618 -1.00518

4 SLHVGTQCA 5.84200 5.65128 0.19072

5 ALPYWNFAT 5.86900 6.11698 -0.24798

6 SLNFMGYVI 5.88100 5.91978 -0.03878

7 NLQSLTNLL 6.00000 6.69729 -0.69729

8 FVTWHRYHL 6.02500 6.26759 -0.24259

9 DPKVKQWPL 6.17600 6.19194 -0.01594

10 ITSQVPFSV 6.19600 6.50917 -0.31317

11 ALAKAAAAI 6.21100 7.00852 -0.79752

12 GLGQVPLIV 6.30100 6.56210 -0.26110

13 MLDLQPETT 6.33500 6.27929 0.05571

14 LLSSNLSWL 6.34200 5.86554 0.47646

15 GLACHQLCA 6.38000 6.06750 0.31250

16 LIGNESFAL 6.41500 6.96803 -0.55303

17 ALAKAAAAV 6.41900 7.03081 -0.61181

18 LLAVGATKV 6.47700 6.49783 -0.02083

19 ALAKAAAAL 6.51100 6.92258 -0.41158

20 WILRGTSFV 6.55600 6.44980 0.10620

21 IISCTCPTV 6.58000 6.90097 -0.32097

22 FLGGTPVCL 6.62300 6.72746 -0.10446

23 ALIHHNTHL 6.62300 6.87117 -0.24817

24 NLSWLSLDV 6.63900 6.11821 0.52079

25 YMIMVKCWM 6.66300 6.97117 -0.30817

26 VLQAGFFLL 6.68200 6.81737 -0.13537

27 GTLGIVCPI 6.71400 6.41758 0.29642

28 VILGVLLLI 6.78500 7.73178 -0.94678

29 VTWHRYHLL 6.79300 6.61429 0.17871

30 PLLPIFFCL 6.79600 7.27473 -0.47873

31 TLGIVCPIC 6.81500 6.15556 0.65944

32 CLTSTVQLV 6.83200 6.39872 0.43328

33 ILLLCLIFL 6.84500 6.34765 0.49735

34 FAFRDLCIV 6.88600 7.48282 -0.59682

35 FLEPGPVTA 6.89800 7.06888 -0.17088

36 ALAKAAAAA 6.94700 6.72459 0.22241

37 LMAVVLASL 6.95400 7.44885 -0.49485

38 YVITTQHWL 6.98300 6.78903 0.19397

39 LLCLIFLLV 6.99600 7.27568 -0.27968

40 ITAQVPFSV 7.02000 6.72818 0.29182

41 YLEPGPVTL 7.05800 7.29440 -0.23640

42 YTDQVPFSV 7.06600 6.79830 0.26770

43 NLYVSLLLL 7.11400 6.94187 0.17213

44 ILHNGAYSL 7.12700 7.32485 -0.19785

45 SIISAVVGI 7.15900 7.82895 -0.66995

46 VVMGTLVAL 7.17400 7.49033 -0.31633

47 YLEPGPVTI 7.18700 7.38033 -0.19333

48 GLSRYVARL 7.24800 7.41684 -0.16884

49 LLAQFTSAI 7.30100 7.51390 -0.21290

50 VLLDYQGML 7.32800 7.54496 -0.21696

51 YLEPGPVTV 7.34200 7.40262 -0.06062

52 ILSPFMPLL 7.34700 7.18965 0.15735

53 YLSPGPVTA 7.38300 6.97101 0.41199

54 IIDQVPFSV 7.39800 7.66531 -0.26731

55 SVYDFFVWL 7.44400 7.89840 -0.45440

56 ITWQVPFSV 7.46300 7.48680 -0.02380

57 ITYQVPFSV 7.48000 7.50397 -0.02397

58 GLYSSTVPV 7.48100 7.45711 0.02389

59 VMGTLVALV 7.55300 7.52480 0.02820

60 LLLCLIFLL 7.58500 7.03177 0.55323

61 SLDDYNHLV 7.58500 7.28675 0.29825

62 VLIQRNPQL 7.64400 7.16255 0.48145

63 SLYADSPSV 7.65800 7.60406 0.05394

64 ILSQVPFSV 7.69900 7.53406 0.16494

65 IMDQVPFSV 7.71900 7.86085 -0.14185

66 QLFEDNYAL 7.76400 7.55418 0.20982

67 ALMDKSLHV 7.77000 7.35669 0.41331

68 YAIDLPVSV 7.79600 7.94210 -0.14610

69 FVWLHYYSV 7.82400 8.69570 -0.87170

70 MLGTHTMEV 7.84500 7.30401 0.54099

71 LLFGYPVYV 7.88600 7.92031 -0.03431

72 ILKEPVHGV 7.92100 7.50163 0.41937

73 YLMPGPVTV 7.93200 7.81886 0.11314

74 WLDQVPFSV 7.93900 7.81447 0.12453

75 KTWGQYWQV 7.95500 7.34383 0.61117

76 ALMPLYACI 8.00000 7.27197 0.72803

77 YLAPGPVTA 8.03200 7.19002 0.84198

78 YLYPGPVTV 8.05100 8.27202 -0.22102

79 LLMGTLGIV 8.09700 7.02008 1.07692

80 YLWPGPVTV 8.12500 8.25485 -0.12985

81 FLLTRILTI 8.14900 8.30382 -0.15482

82 GLLGWSPQA 8.23700 7.63481 0.60219

83 ILYQVPFSV 8.31000 8.52886 -0.21886

84 GILTVILGV 8.34700 8.11989 0.22711

85 NMVPFFPPV 8.39800 8.06215 0.33585

86 ILDQVPFSV 8.48100 7.54048 0.94052

87 YLFPGPVTA 8.49500 7.91158 0.58342

88 YLDQVPFSV 8.63800 7.82319 0.81481

89 ILFQVPFSV 8.69900 8.47464 0.22436

90 ILWQVPFSV 8.77000 8.51169 0.25831

Correlation Coeffecient and Stand Error

CORL: 0.829648 CORL~2: 0.688316

RES: 0.444146 SEE: 0.046817

Statistical indices of Training calculation:

ITERATION (A): 5

No. Sequence Expt. Act Predicted Act Difference

1 VALVGLFVL 5.14800 6.23689 -1.08889

2 GTLVALVGL 5.34200 6.01887 -0.67687

3 LQTTIHDII 5.50100 6.50802 -1.00702

4 SLHVGTQCA 5.84200 5.65178 0.19022

5 ALPYWNFAT 5.86900 6.14963 -0.28063

6 SLNFMGYVI 5.88100 5.97651 -0.09551

7 NLQSLTNLL 6.00000 6.73180 -0.73180

8 FVTWHRYHL 6.02500 6.29033 -0.26533

9 DPKVKQWPL 6.17600 6.20748 -0.03148

10 ITSQVPFSV 6.19600 6.52641 -0.33041

11 ALAKAAAAI 6.21100 6.99764 -0.78664

12 GLGQVPLIV 6.30100 6.56026 -0.25926

13 MLDLQPETT 6.33500 6.28358 0.05142

14 LLSSNLSWL 6.34200 5.91843 0.42357

15 GLACHQLCA 6.38000 6.02879 0.35121

16 LIGNESFAL 6.41500 7.00327 -0.58827

17 ALAKAAAAV 6.41900 7.00085 -0.58185

18 LLAVGATKV 6.47700 6.50264 -0.02564

19 ALAKAAAAL 6.51100 6.91687 -0.40587

20 WILRGTSFV 6.55600 6.47995 0.07605

21 IISCTCPTV 6.58000 6.88254 -0.30254

22 FLGGTPVCL 6.62300 6.75229 -0.12929

23 ALIHHNTHL 6.62300 6.91023 -0.28723

24 NLSWLSLDV 6.63900 6.14717 0.49183

25 YMIMVKCWM 6.66300 6.98629 -0.32329

26 VLQAGFFLL 6.68200 6.84189 -0.15989

27 GTLGIVCPI 6.71400 6.48083 0.23317

28 VILGVLLLI 6.78500 7.72758 -0.94258

29 VTWHRYHLL 6.79300 6.64130 0.15170

30 PLLPIFFCL 6.79600 7.30038 -0.50438

31 TLGIVCPIC 6.81500 6.17758 0.63742

32 CLTSTVQLV 6.83200 6.43701 0.39499

33 ILLLCLIFL 6.84500 6.40554 0.43946

34 FAFRDLCIV 6.88600 7.40889 -0.52289

35 FLEPGPVTA 6.89800 7.02998 -0.13198

36 ALAKAAAAA 6.94700 6.68625 0.26075

37 LMAVVLASL 6.95400 7.45103 -0.49703

38 YVITTQHWL 6.98300 6.79455 0.18845

39 LLCLIFLLV 6.99600 7.26984 -0.27384

40 ITAQVPFSV 7.02000 6.73125 0.28875

41 YLEPGPVTL 7.05800 7.29700 -0.23900

42 YTDQVPFSV 7.06600 6.81341 0.25259

43 NLYVSLLLL 7.11400 6.97198 0.14202

44 ILHNGAYSL 7.12700 7.34583 -0.21883

45 SIISAVVGI 7.15900 7.82138 -0.66238

46 VVMGTLVAL 7.17400 7.50888 -0.33488

47 YLEPGPVTI 7.18700 7.37777 -0.19077

48 GLSRYVARL 7.24800 7.44083 -0.19283

49 LLAQFTSAI 7.30100 7.53160 -0.23060

50 VLLDYQGML 7.32800 7.57583 -0.24783

51 YLEPGPVTV 7.34200 7.38098 -0.03898

52 ILSPFMPLL 7.34700 7.19520 0.15180

53 YLSPGPVTA 7.38300 6.94358 0.43942

54 IIDQVPFSV 7.39800 7.64858 -0.25058

55 SVYDFFVWL 7.44400 7.88223 -0.43823

56 ITWQVPFSV 7.46300 7.49078 -0.02778

57 ITYQVPFSV 7.48000 7.52238 -0.04238

58 GLYSSTVPV 7.48100 7.47283 0.00817

59 VMGTLVALV 7.55300 7.47822 0.07478

60 LLLCLIFLL 7.58500 7.05837 0.52663

61 SLDDYNHLV 7.58500 7.27566 0.30934

62 VLIQRNPQL 7.64400 7.18680 0.45720

63 SLYADSPSV 7.65800 7.62083 0.03717

64 ILSQVPFSV 7.69900 7.52400 0.17500

65 IMDQVPFSV 7.71900 7.83660 -0.11760

66 QLFEDNYAL 7.76400 7.58476 0.17924

67 ALMDKSLHV 7.77000 7.36445 0.40555

68 YAIDLPVSV 7.79600 7.90825 -0.11225

69 FVWLHYYSV 7.82400 8.62316 -0.79916

70 MLGTHTMEV 7.84500 7.29262 0.55238

71 LLFGYPVYV 7.88600 7.89693 -0.01093

72 ILKEPVHGV 7.92100 7.49598 0.42502

73 YLMPGPVTV 7.93200 7.80282 0.12918

74 WLDQVPFSV 7.93900 7.79496 0.14404

75 KTWGQYWQV 7.95500 7.34753 0.60747

76 ALMPLYACI 8.00000 7.28828 0.71172

77 YLAPGPVTA 8.03200 7.14841 0.88359

78 YLYPGPVTV 8.05100 8.25415 -0.20315

79 LLMGTLGIV 8.09700 7.03899 1.05801

80 YLWPGPVTV 8.12500 8.22255 -0.09755

81 FLLTRILTI 8.14900 8.27353 -0.12453

82 GLLGWSPQA 8.23700 7.61782 0.61918

83 ILYQVPFSV 8.31000 8.51997 -0.20997

84 GILTVILGV 8.34700 8.08792 0.25908

85 NMVPFFPPV 8.39800 8.03164 0.36636

86 ILDQVPFSV 8.48100 7.53125 0.94975

87 YLFPGPVTA 8.49500 7.86785 0.62715

88 YLDQVPFSV 8.63800 7.81100 0.82700

89 ILFQVPFSV 8.69900 8.44827 0.25073

90 ILWQVPFSV 8.77000 8.48837 0.28163

Correlation Coeffecient and Stand Error

CORL: 0.830100 CORL~2: 0.689066

RES: 0.443265 SEE: 0.046724

Statistical indices of Training calculation:

ITERATION (B): 5

No. Sequence Expt. Act Predicted Act Difference

1 VALVGLFVL 5.14800 6.20235 -1.05435

2 GTLVALVGL 5.34200 5.94469 -0.60269

3 LQTTIHDII 5.50100 6.47566 -0.97466

4 SLHVGTQCA 5.84200 5.62103 0.22097

5 ALPYWNFAT 5.86900 6.20626 -0.33726

6 SLNFMGYVI 5.88100 5.90859 -0.02759

7 NLQSLTNLL 6.00000 6.70717 -0.70717

8 FVTWHRYHL 6.02500 6.27217 -0.24717

9 DPKVKQWPL 6.17600 6.16338 0.01262

10 ITSQVPFSV 6.19600 6.47588 -0.27988

11 ALAKAAAAI 6.21100 6.97665 -0.76565

12 GLGQVPLIV 6.30100 6.52585 -0.22485

13 MLDLQPETT 6.33500 6.36350 -0.02850

14 LLSSNLSWL 6.34200 5.89308 0.44892

15 GLACHQLCA 6.38000 6.01612 0.36388

16 LIGNESFAL 6.41500 7.00388 -0.58888

17 ALAKAAAAV 6.41900 6.97947 -0.56047

18 LLAVGATKV 6.47700 6.46336 0.01364

19 ALAKAAAAL 6.51100 6.90576 -0.39476

20 WILRGTSFV 6.55600 6.45844 0.09756

21 IISCTCPTV 6.58000 6.86541 -0.28541

22 FLGGTPVCL 6.62300 6.76212 -0.13912

23 ALIHHNTHL 6.62300 6.89047 -0.26747

24 NLSWLSLDV 6.63900 6.08067 0.55833

25 YMIMVKCWM 6.66300 6.96744 -0.30444

26 VLQAGFFLL 6.68200 6.85081 -0.16881

27 GTLGIVCPI 6.71400 6.42025 0.29375

28 VILGVLLLI 6.78500 7.74430 -0.95930

29 VTWHRYHLL 6.79300 6.61014 0.18286

30 PLLPIFFCL 6.79600 7.31723 -0.52123

31 TLGIVCPIC 6.81500 6.13375 0.68125

32 CLTSTVQLV 6.83200 6.41440 0.41760

33 ILLLCLIFL 6.84500 6.38848 0.45652

34 FAFRDLCIV 6.88600 7.40635 -0.52035

35 FLEPGPVTA 6.89800 7.07247 -0.17447

36 ALAKAAAAA 6.94700 6.70335 0.24365

37 LMAVVLASL 6.95400 7.46600 -0.51200

38 YVITTQHWL 6.98300 6.80656 0.17644

39 LLCLIFLLV 6.99600 7.27167 -0.27567

40 ITAQVPFSV 7.02000 6.68473 0.33527

41 YLEPGPVTL 7.05800 7.31409 -0.25609

42 YTDQVPFSV 7.06600 6.78467 0.28133

43 NLYVSLLLL 7.11400 6.94985 0.16415

44 ILHNGAYSL 7.12700 7.35486 -0.22786

45 SIISAVVGI 7.15900 7.82653 -0.66753

46 VVMGTLVAL 7.17400 7.52725 -0.35325

47 YLEPGPVTI 7.18700 7.38499 -0.19799

48 GLSRYVARL 7.24800 7.44768 -0.19968

49 LLAQFTSAI 7.30100 7.52444 -0.22344

50 VLLDYQGML 7.32800 7.59283 -0.26483

51 YLEPGPVTV 7.34200 7.38780 -0.04580

52 ILSPFMPLL 7.34700 7.20803 0.13897

53 YLSPGPVTA 7.38300 6.98648 0.39652

54 IIDQVPFSV 7.39800 7.66004 -0.26204

55 SVYDFFVWL 7.44400 7.91404 -0.47004

56 ITWQVPFSV 7.46300 7.45916 0.00384

57 ITYQVPFSV 7.48000 7.49138 -0.01138

58 GLYSSTVPV 7.48100 7.44625 0.03475

59 VMGTLVALV 7.55300 7.49831 0.05469

60 LLLCLIFLL 7.58500 7.05800 0.52700

61 SLDDYNHLV 7.58500 7.26112 0.32388

62 VLIQRNPQL 7.64400 7.18270 0.46130

63 SLYADSPSV 7.65800 7.59785 0.06015

64 ILSQVPFSV 7.69900 7.52881 0.17019

65 IMDQVPFSV 7.71900 7.85849 -0.13949

66 QLFEDNYAL 7.76400 7.58327 0.18073

67 ALMDKSLHV 7.77000 7.34840 0.42160

68 YAIDLPVSV 7.79600 7.92550 -0.12950

69 FVWLHYYSV 7.82400 8.67621 -0.85221

70 MLGTHTMEV 7.84500 7.28839 0.55661

71 LLFGYPVYV 7.88600 7.91172 -0.02572

72 ILKEPVHGV 7.92100 7.49963 0.42137

73 YLMPGPVTV 7.93200 7.81792 0.11408

74 WLDQVPFSV 7.93900 7.82032 0.11868

75 KTWGQYWQV 7.95500 7.32218 0.63282

76 ALMPLYACI 8.00000 7.28797 0.71203

77 YLAPGPVTA 8.03200 7.19533 0.83667

78 YLYPGPVTV 8.05100 8.27810 -0.22710

79 LLMGTLGIV 8.09700 7.02586 1.07114

80 YLWPGPVTV 8.12500 8.24588 -0.12088

81 FLLTRILTI 8.14900 8.31341 -0.16441

82 GLLGWSPQA 8.23700 7.65521 0.58179

83 ILYQVPFSV 8.31000 8.54431 -0.23431

84 GILTVILGV 8.34700 8.10232 0.24468

85 NMVPFFPPV 8.39800 8.05715 0.34085

86 ILDQVPFSV 8.48100 7.53620 0.94480

87 YLFPGPVTA 8.49500 7.92887 0.56613

88 YLDQVPFSV 8.63800 7.83760 0.80040

89 ILFQVPFSV 8.69900 8.47121 0.22779

90 ILWQVPFSV 8.77000 8.51209 0.25791

Correlation Coeffecient and Stand Error

CORL: 0.831264 CORL~2: 0.690999

RES: 0.442259 SEE: 0.046618

Statistical indices of Training calculation:

ITERATION (A): 6

No. Sequence Expt. Act Predicted Act Difference

1 VALVGLFVL 5.14800 6.21378 -1.06578

2 GTLVALVGL 5.34200 6.00747 -0.66547

3 LQTTIHDII 5.50100 6.46688 -0.96588

4 SLHVGTQCA 5.84200 5.62250 0.21950

5 ALPYWNFAT 5.86900 6.24186 -0.37286

6 SLNFMGYVI 5.88100 5.97671 -0.09571

7 NLQSLTNLL 6.00000 6.74646 -0.74646

8 FVTWHRYHL 6.02500 6.28545 -0.26045

9 DPKVKQWPL 6.17600 6.18465 -0.00865

10 ITSQVPFSV 6.19600 6.49318 -0.29718

11 ALAKAAAAI 6.21100 6.95962 -0.74862

12 GLGQVPLIV 6.30100 6.52766 -0.22666

13 MLDLQPETT 6.33500 6.35980 -0.02480

14 LLSSNLSWL 6.34200 5.95345 0.38855

15 GLACHQLCA 6.38000 5.96512 0.41488

16 LIGNESFAL 6.41500 7.04764 -0.63264

17 ALAKAAAAV 6.41900 6.93985 -0.52085

18 LLAVGATKV 6.47700 6.47168 0.00532

19 ALAKAAAAL 6.51100 6.89598 -0.38498

20 WILRGTSFV 6.55600 6.49577 0.06023

21 IISCTCPTV 6.58000 6.83771 -0.25771

22 FLGGTPVCL 6.62300 6.78959 -0.16659

23 ALIHHNTHL 6.62300 6.93810 -0.31510

24 NLSWLSLDV 6.63900 6.11254 0.52646

25 YMIMVKCWM 6.66300 6.98342 -0.32042

26 VLQAGFFLL 6.68200 6.87740 -0.19540

27 GTLGIVCPI 6.71400 6.48362 0.23038

28 VILGVLLLI 6.78500 7.73058 -0.94558

29 VTWHRYHLL 6.79300 6.63438 0.15862

30 PLLPIFFCL 6.79600 7.34560 -0.54960

31 TLGIVCPIC 6.81500 6.16147 0.65353

32 CLTSTVQLV 6.83200 6.45586 0.37614

33 ILLLCLIFL 6.84500 6.45728 0.38772

34 FAFRDLCIV 6.88600 7.31949 -0.43349

35 FLEPGPVTA 6.89800 7.02735 -0.12935

36 ALAKAAAAA 6.94700 6.65689 0.29011

37 LMAVVLASL 6.95400 7.47357 -0.51957

38 YVITTQHWL 6.98300 6.80104 0.18196

39 LLCLIFLLV 6.99600 7.26446 -0.26846

40 ITAQVPFSV 7.02000 6.68603 0.33397

41 YLEPGPVTL 7.05800 7.31748 -0.25948

42 YTDQVPFSV 7.06600 6.80164 0.26436

43 NLYVSLLLL 7.11400 6.99039 0.12361

44 ILHNGAYSL 7.12700 7.38441 -0.25741

45 SIISAVVGI 7.15900 7.81045 -0.65145

46 VVMGTLVAL 7.17400 7.53919 -0.36519

47 YLEPGPVTI 7.18700 7.38112 -0.19412

48 GLSRYVARL 7.24800 7.48891 -0.24091

49 LLAQFTSAI 7.30100 7.55195 -0.25095

50 VLLDYQGML 7.32800 7.63257 -0.30457

51 YLEPGPVTV 7.34200 7.36135 -0.01935

52 ILSPFMPLL 7.34700 7.21005 0.13695

53 YLSPGPVTA 7.38300 6.95484 0.42816

54 IIDQVPFSV 7.39800 7.63967 -0.24167

55 SVYDFFVWL 7.44400 7.88903 -0.44503

56 ITWQVPFSV 7.46300 7.46579 -0.00279

57 ITYQVPFSV 7.48000 7.51630 -0.03630

58 GLYSSTVPV 7.48100 7.46332 0.01768

59 VMGTLVALV 7.55300 7.44205 0.11095

60 LLLCLIFLL 7.58500 7.09104 0.49396

61 SLDDYNHLV 7.58500 7.24742 0.33758

62 VLIQRNPQL 7.64400 7.21385 0.43015

63 SLYADSPSV 7.65800 7.62360 0.03440

64 ILSQVPFSV 7.69900 7.51995 0.17905

65 IMDQVPFSV 7.71900 7.83360 -0.11460

66 QLFEDNYAL 7.76400 7.62275 0.14125

67 ALMDKSLHV 7.77000 7.36275 0.40725

68 YAIDLPVSV 7.79600 7.88629 -0.09029

69 FVWLHYYSV 7.82400 8.58413 -0.76013

70 MLGTHTMEV 7.84500 7.27803 0.56697

71 LLFGYPVYV 7.88600 7.88623 -0.00023

72 ILKEPVHGV 7.92100 7.49579 0.42521

73 YLMPGPVTV 7.93200 7.80117 0.13083

74 WLDQVPFSV 7.93900 7.80133 0.13767

75 KTWGQYWQV 7.95500 7.32474 0.63026

76 ALMPLYACI 8.00000 7.30247 0.69753

77 YLAPGPVTA 8.03200 7.14769 0.88431

78 YLYPGPVTV 8.05100 8.26092 -0.20992

79 LLMGTLGIV 8.09700 7.05003 1.04697

80 YLWPGPVTV 8.12500 8.21042 -0.08542

81 FLLTRILTI 8.14900 8.27973 -0.13073

82 GLLGWSPQA 8.23700 7.64575 0.59125

83 ILYQVPFSV 8.31000 8.54307 -0.23307

84 GILTVILGV 8.34700 8.06133 0.28567

85 NMVPFFPPV 8.39800 8.00994 0.38806

86 ILDQVPFSV 8.48100 7.52849 0.95251

87 YLFPGPVTA 8.49500 7.88281 0.61219

88 YLDQVPFSV 8.63800 7.82842 0.80958

89 ILFQVPFSV 8.69900 8.44793 0.25107

90 ILWQVPFSV 8.77000 8.49257 0.27743

Correlation Coeffecient and Stand Error

CORL: 0.831990 CORL~2: 0.692207

RES: 0.441020 SEE: 0.046488

Statistical indices of Training calculation:

ITERATION (B): 6

No. Sequence Expt. Act Predicted Act Difference

1 VALVGLFVL 5.14800 6.17387 -1.02587

2 GTLVALVGL 5.34200 5.90898 -0.56698

3 LQTTIHDII 5.50100 6.41871 -0.91771

4 SLHVGTQCA 5.84200 5.59588 0.24612

5 ALPYWNFAT 5.86900 6.32468 -0.45568

6 SLNFMGYVI 5.88100 5.89515 -0.01415

7 NLQSLTNLL 6.00000 6.70826 -0.70826

8 FVTWHRYHL 6.02500 6.26399 -0.23899

9 DPKVKQWPL 6.17600 6.12987 0.04613

10 ITSQVPFSV 6.19600 6.42877 -0.23277

11 ALAKAAAAI 6.21100 6.92466 -0.71366

12 GLGQVPLIV 6.30100 6.49050 -0.18950

13 MLDLQPETT 6.33500 6.46807 -0.13307

14 LLSSNLSWL 6.34200 5.93374 0.40826

15 GLACHQLCA 6.38000 5.96283 0.41717

16 LIGNESFAL 6.41500 7.04621 -0.63121

17 ALAKAAAAV 6.41900 6.90899 -0.48999

18 LLAVGATKV 6.47700 6.42160 0.05540

19 ALAKAAAAL 6.51100 6.87422 -0.36322

20 WILRGTSFV 6.55600 6.47513 0.08087

21 IISCTCPTV 6.58000 6.81731 -0.23731

22 FLGGTPVCL 6.62300 6.80476 -0.18176

23 ALIHHNTHL 6.62300 6.90366 -0.28066

24 NLSWLSLDV 6.63900 6.02827 0.61073

25 YMIMVKCWM 6.66300 6.96337 -0.30037

26 VLQAGFFLL 6.68200 6.90257 -0.22057

27 GTLGIVCPI 6.71400 6.40337 0.31063

28 VILGVLLLI 6.78500 7.74859 -0.96359

29 VTWHRYHLL 6.79300 6.59942 0.19358

30 PLLPIFFCL 6.79600 7.37790 -0.58190

31 TLGIVCPIC 6.81500 6.11116 0.70384

32 CLTSTVQLV 6.83200 6.43679 0.39521

33 ILLLCLIFL 6.84500 6.44742 0.39758

34 FAFRDLCIV 6.88600 7.31463 -0.42863

35 FLEPGPVTA 6.89800 7.08829 -0.19029

36 ALAKAAAAA 6.94700 6.68471 0.26229

37 LMAVVLASL 6.95400 7.48813 -0.53413

38 YVITTQHWL 6.98300 6.81909 0.16391

39 LLCLIFLLV 6.99600 7.27861 -0.28261

40 ITAQVPFSV 7.02000 6.62533 0.39467

41 YLEPGPVTL 7.05800 7.33343 -0.27543

42 YTDQVPFSV 7.06600 6.76451 0.30149

43 NLYVSLLLL 7.11400 6.96439 0.14961

44 ILHNGAYSL 7.12700 7.39264 -0.26564

45 SIISAVVGI 7.15900 7.80821 -0.64921

46 VVMGTLVAL 7.17400 7.55809 -0.38409

47 YLEPGPVTI 7.18700 7.38388 -0.19688

48 GLSRYVARL 7.24800 7.49205 -0.24405

49 LLAQFTSAI 7.30100 7.52935 -0.22835

50 VLLDYQGML 7.32800 7.64714 -0.31914

51 YLEPGPVTV 7.34200 7.36821 -0.02621

52 ILSPFMPLL 7.34700 7.22783 0.11917

53 YLSPGPVTA 7.38300 7.01801 0.36499

54 IIDQVPFSV 7.39800 7.65454 -0.25654

55 SVYDFFVWL 7.44400 7.93592 -0.49192

56 ITWQVPFSV 7.46300 7.42010 0.04290

57 ITYQVPFSV 7.48000 7.47157 0.00843

58 GLYSSTVPV 7.48100 7.42496 0.05604

59 VMGTLVALV 7.55300 7.46927 0.08373

60 LLLCLIFLL 7.58500 7.09650 0.48850

61 SLDDYNHLV 7.58500 7.22511 0.35989

62 VLIQRNPQL 7.64400 7.20026 0.44374

63 SLYADSPSV 7.65800 7.58618 0.07182

64 ILSQVPFSV 7.69900 7.52693 0.17207

65 IMDQVPFSV 7.71900 7.86195 -0.14295

66 QLFEDNYAL 7.76400 7.61595 0.14805

67 ALMDKSLHV 7.77000 7.33785 0.43215

68 YAIDLPVSV 7.79600 7.89885 -0.10285

69 FVWLHYYSV 7.82400 8.65499 -0.83099

70 MLGTHTMEV 7.84500 7.26763 0.57737

71 LLFGYPVYV 7.88600 7.91069 -0.02469

72 ILKEPVHGV 7.92100 7.49836 0.42264

73 YLMPGPVTV 7.93200 7.81648 0.11552

74 WLDQVPFSV 7.93900 7.83313 0.10587

75 KTWGQYWQV 7.95500 7.30019 0.65481

76 ALMPLYACI 8.00000 7.30896 0.69104

77 YLAPGPVTA 8.03200 7.21457 0.81743

78 YLYPGPVTV 8.05100 8.28508 -0.23408

79 LLMGTLGIV 8.09700 7.03849 1.05851

80 YLWPGPVTV 8.12500 8.23361 -0.10861

81 FLLTRILTI 8.14900 8.31934 -0.17034

82 GLLGWSPQA 8.23700 7.69313 0.54387

83 ILYQVPFSV 8.31000 8.56973 -0.25973

84 GILTVILGV 8.34700 8.07415 0.27285

85 NMVPFFPPV 8.39800 8.04789 0.35011

86 ILDQVPFSV 8.48100 7.53563 0.94537

87 YLFPGPVTA 8.49500 7.96383 0.53117

88 YLDQVPFSV 8.63800 7.86266 0.77534

89 ILFQVPFSV 8.69900 8.47275 0.22625

90 ILWQVPFSV 8.77000 8.51825 0.25175

Correlation Coeffecient and Stand Error

CORL: 0.833819 CORL~2: 0.695254

RES: 0.439306 SEE: 0.046307

Statistical indices of Training calculation:

ITERATION (A): 7

No. Sequence Expt. Act Predicted Act Difference

1 VALVGLFVL 5.14800 6.18373 -1.03573

2 GTLVALVGL 5.34200 5.99811 -0.65611

3 LQTTIHDII 5.50100 6.38959 -0.88859

4 SLHVGTQCA 5.84200 5.59823 0.24377

5 ALPYWNFAT 5.86900 6.36303 -0.49403

6 SLNFMGYVI 5.88100 5.99435 -0.11335

7 NLQSLTNLL 6.00000 6.76343 -0.76343

8 FVTWHRYHL 6.02500 6.25645 -0.23145

9 DPKVKQWPL 6.17600 6.15988 0.01612

10 ITSQVPFSV 6.19600 6.44767 -0.25167

11 ALAKAAAAI 6.21100 6.88880 -0.67780

12 GLGQVPLIV 6.30100 6.49104 -0.19004

13 MLDLQPETT 6.33500 6.45097 -0.11597

14 LLSSNLSWL 6.34200 6.01400 0.32800

15 GLACHQLCA 6.38000 5.88032 0.49968

16 LIGNESFAL 6.41500 7.10840 -0.69340

17 ALAKAAAAV 6.41900 6.84357 -0.42457

18 LLAVGATKV 6.47700 6.43508 0.04192

19 ALAKAAAAL 6.51100 6.84905 -0.33805

20 WILRGTSFV 6.55600 6.52582 0.03018

21 IISCTCPTV 6.58000 6.76721 -0.18721

22 FLGGTPVCL 6.62300 6.83861 -0.21561

23 ALIHHNTHL 6.62300 6.97081 -0.34781

24 NLSWLSLDV 6.63900 6.06832 0.57068

25 YMIMVKCWM 6.66300 6.98496 -0.32196

26 VLQAGFFLL 6.68200 6.93223 -0.25023

27 GTLGIVCPI 6.71400 6.48481 0.22919

28 VILGVLLLI 6.78500 7.71790 -0.93290

29 VTWHRYHLL 6.79300 6.62405 0.16895

30 PLLPIFFCL 6.79600 7.41662 -0.62062

31 TLGIVCPIC 6.81500 6.14515 0.66985

32 CLTSTVQLV 6.83200 6.49517 0.33683

33 ILLLCLIFL 6.84500 6.54495 0.30005

34 FAFRDLCIV 6.88600 7.18569 -0.29969

35 FLEPGPVTA 6.89800 7.02852 -0.13052

36 ALAKAAAAA 6.94700 6.61338 0.33362

37 LMAVVLASL 6.95400 7.51061 -0.55661

38 YVITTQHWL 6.98300 6.78660 0.19640

39 LLCLIFLLV 6.99600 7.26927 -0.27327

40 ITAQVPFSV 7.02000 6.62286 0.39714

41 YLEPGPVTL 7.05800 7.34021 -0.28221

42 YTDQVPFSV 7.06600 6.78970 0.27630

43 NLYVSLLLL 7.11400 7.02892 0.08508

44 ILHNGAYSL 7.12700 7.43858 -0.31158

45 SIISAVVGI 7.15900 7.77585 -0.61685

46 VVMGTLVAL 7.17400 7.56220 -0.38820

47 YLEPGPVTI 7.18700 7.37996 -0.19296

48 GLSRYVARL 7.24800 7.55728 -0.30928

49 LLAQFTSAI 7.30100 7.57678 -0.27578

50 VLLDYQGML 7.32800 7.70309 -0.37509

51 YLEPGPVTV 7.34200 7.33473 0.00727

52 ILSPFMPLL 7.34700 7.22553 0.12147

53 YLSPGPVTA 7.38300 6.97453 0.40847

54 IIDQVPFSV 7.39800 7.62372 -0.22572

55 SVYDFFVWL 7.44400 7.88231 -0.43831

56 ITWQVPFSV 7.46300 7.43333 0.02967

57 ITYQVPFSV 7.48000 7.51372 -0.03372

58 GLYSSTVPV 7.48100 7.45520 0.02580

59 VMGTLVALV 7.55300 7.38618 0.16682

60 LLLCLIFLL 7.58500 7.14489 0.44011

61 SLDDYNHLV 7.58500 7.20628 0.37872

62 VLIQRNPQL 7.64400 7.24339 0.40061

63 SLYADSPSV 7.65800 7.63605 0.02195

64 ILSQVPFSV 7.69900 7.51720 0.18180

65 IMDQVPFSV 7.71900 7.83733 -0.11833

66 QLFEDNYAL 7.76400 7.67386 0.09014

67 ALMDKSLHV 7.77000 7.36905 0.40095

68 YAIDLPVSV 7.79600 7.84190 -0.04590

69 FVWLHYYSV 7.82400 8.51414 -0.69014

70 MLGTHTMEV 7.84500 7.25432 0.59068

71 LLFGYPVYV 7.88600 7.88262 0.00338

72 ILKEPVHGV 7.92100 7.49602 0.42498

73 YLMPGPVTV 7.93200 7.80142 0.13058

74 WLDQVPFSV 7.93900 7.81310 0.12590

75 KTWGQYWQV 7.95500 7.31003 0.64497

76 ALMPLYACI 8.00000 7.33043 0.66957

77 YLAPGPVTA 8.03200 7.14971 0.88229

78 YLYPGPVTV 8.05100 8.27078 -0.21978

79 LLMGTLGIV 8.09700 7.08645 1.01055

80 YLWPGPVTV 8.12500 8.19038 -0.06538

81 FLLTRILTI 8.14900 8.27564 -0.12664

82 GLLGWSPQA 8.23700 7.69660 0.54040

83 ILYQVPFSV 8.31000 8.58326 -0.27326

84 GILTVILGV 8.34700 8.01319 0.33381

85 NMVPFFPPV 8.39800 7.97858 0.41942

86 ILDQVPFSV 8.48100 7.53003 0.95097

87 YLFPGPVTA 8.49500 7.90810 0.58690

88 YLDQVPFSV 8.63800 7.85923 0.77877

89 ILFQVPFSV 8.69900 8.45078 0.24822

90 ILWQVPFSV 8.77000 8.50286 0.26714

Correlation Coeffecient and Stand Error

CORL: 0.835608 CORL~2: 0.698241

RES: 0.436675 SEE: 0.046030

Statistical indices of Training calculation:

ITERATION (B): 7

No. Sequence Expt. Act Predicted Act Difference

1 VALVGLFVL 5.14800 6.12257 -0.97457

2 GTLVALVGL 5.34200 5.83403 -0.49203

3 LQTTIHDII 5.50100 6.30005 -0.79905

4 SLHVGTQCA 5.84200 5.59149 0.25051

5 ALPYWNFAT 5.86900 6.50344 -0.63444

6 SLNFMGYVI 5.88100 5.88265 -0.00165

7 NLQSLTNLL 6.00000 6.69450 -0.69450

8 FVTWHRYHL 6.02500 6.21921 -0.19421

9 DPKVKQWPL 6.17600 6.07625 0.09975

10 ITSQVPFSV 6.19600 6.35368 -0.15768

11 ALAKAAAAI 6.21100 6.81479 -0.60379

12 GLGQVPLIV 6.30100 6.45493 -0.15393

13 MLDLQPETT 6.33500 6.62115 -0.28615

14 LLSSNLSWL 6.34200 6.00555 0.33645

15 GLACHQLCA 6.38000 5.91093 0.46907

16 LIGNESFAL 6.41500 7.09909 -0.68409

17 ALAKAAAAV 6.41900 6.79071 -0.37171

18 LLAVGATKV 6.47700 6.36146 0.11554

19 ALAKAAAAL 6.51100 6.79363 -0.28263

20 WILRGTSFV 6.55600 6.51504 0.04096

21 IISCTCPTV 6.58000 6.74092 -0.16092

22 FLGGTPVCL 6.62300 6.86335 -0.24035

23 ALIHHNTHL 6.62300 6.89602 -0.27302

24 NLSWLSLDV 6.63900 5.94509 0.69391

25 YMIMVKCWM 6.66300 6.96190 -0.29890

26 VLQAGFFLL 6.68200 6.98840 -0.30640

27 GTLGIVCPI 6.71400 6.35311 0.36089

28 VILGVLLLI 6.78500 7.73878 -0.95378

29 VTWHRYHLL 6.79300 6.56860 0.22440

30 PLLPIFFCL 6.79600 7.47818 -0.68218

31 TLGIVCPIC 6.81500 6.07682 0.73818

32 CLTSTVQLV 6.83200 6.48735 0.34465

33 ILLLCLIFL 6.84500 6.54971 0.29529

34 FAFRDLCIV 6.88600 7.17761 -0.29161

35 FLEPGPVTA 6.89800 7.13719 -0.23919

36 ALAKAAAAA 6.94700 6.66816 0.27884

37 LMAVVLASL 6.95400 7.51782 -0.56382

38 YVITTQHWL 6.98300 6.81332 0.16968

39 LLCLIFLLV 6.99600 7.31317 -0.31717

40 ITAQVPFSV 7.02000 6.53191 0.48809

41 YLEPGPVTL 7.05800 7.34795 -0.28995

42 YTDQVPFSV 7.06600 6.73609 0.32991

43 NLYVSLLLL 7.11400 6.99017 0.12383

44 ILHNGAYSL 7.12700 7.44098 -0.31398

45 SIISAVVGI 7.15900 7.74949 -0.59049

46 VVMGTLVAL 7.17400 7.57150 -0.39750

47 YLEPGPVTI 7.18700 7.36911 -0.18211

48 GLSRYVARL 7.24800 7.54360 -0.29560

49 LLAQFTSAI 7.30100 7.51842 -0.21742

50 VLLDYQGML 7.32800 7.70890 -0.38090

51 YLEPGPVTV 7.34200 7.34503 -0.00303

52 ILSPFMPLL 7.34700 7.25180 0.09520

53 YLSPGPVTA 7.38300 7.09022 0.29278

54 IIDQVPFSV 7.39800 7.65081 -0.25281

55 SVYDFFVWL 7.44400 7.95424 -0.51024

56 ITWQVPFSV 7.46300 7.35642 0.10658

57 ITYQVPFSV 7.48000 7.43821 0.04179

58 GLYSSTVPV 7.48100 7.39695 0.08405

59 VMGTLVALV 7.55300 7.43377 0.11923

60 LLLCLIFLL 7.58500 7.16129 0.42371

61 SLDDYNHLV 7.58500 7.17412 0.41088

62 VLIQRNPQL 7.64400 7.20254 0.44146

63 SLYADSPSV 7.65800 7.56683 0.09117

64 ILSQVPFSV 7.69900 7.53434 0.16466

65 IMDQVPFSV 7.71900 7.88661 -0.16761

66 QLFEDNYAL 7.76400 7.65157 0.11243

67 ALMDKSLHV 7.77000 7.32839 0.44161

68 YAIDLPVSV 7.79600 7.84183 -0.04583

69 FVWLHYYSV 7.82400 8.62308 -0.79908

70 MLGTHTMEV 7.84500 7.23973 0.60527

71 LLFGYPVYV 7.88600 7.93993 -0.05393

72 ILKEPVHGV 7.92100 7.49602 0.42498

73 YLMPGPVTV 7.93200 7.81981 0.11219

74 WLDQVPFSV 7.93900 7.86499 0.07401

75 KTWGQYWQV 7.95500 7.28675 0.66825

76 ALMPLYACI 8.00000 7.34563 0.65437

77 YLAPGPVTA 8.03200 7.26844 0.76356

78 YLYPGPVTV 8.05100 8.29729 -0.24629

79 LLMGTLGIV 8.09700 7.08335 1.01365

80 YLWPGPVTV 8.12500 8.21551 -0.09051

81 FLLTRILTI 8.14900 8.31229 -0.16329

82 GLLGWSPQA 8.23700 7.77507 0.46193

83 ILYQVPFSV 8.31000 8.61886 -0.30886

84 GILTVILGV 8.34700 8.02533 0.32167

85 NMVPFFPPV 8.39800 8.04560 0.35240

86 ILDQVPFSV 8.48100 7.54739 0.93361

87 YLFPGPVTA 8.49500 8.03997 0.45503

88 YLDQVPFSV 8.63800 7.91674 0.72126

89 ILFQVPFSV 8.69900 8.48409 0.21491

90 ILWQVPFSV 8.77000 8.53707 0.23293

Correlation Coeffecient and Stand Error

CORL: 0.839961 CORL~2: 0.705535

RES: 0.432169 SEE: 0.045555

Statistical indices of Training calculation:

ITERATION (A): 8

No. Sequence Expt. Act Predicted Act Difference

1 VALVGLFVL 5.14800 6.11164 -0.96364

2 GTLVALVGL 5.34200 5.98874 -0.64674

3 LQTTIHDII 5.50100 6.21935 -0.71835

4 SLHVGTQCA 5.84200 5.61749 0.22451

5 ALPYWNFAT 5.86900 6.53074 -0.66174

6 SLNFMGYVI 5.88100 6.05736 -0.17636

7 NLQSLTNLL 6.00000 6.78452 -0.78452

8 FVTWHRYHL 6.02500 6.14809 -0.12309

9 DPKVKQWPL 6.17600 6.11455 0.06145

10 ITSQVPFSV 6.19600 6.39065 -0.19465

11 ALAKAAAAI 6.21100 6.72521 -0.51421

12 GLGQVPLIV 6.30100 6.46019 -0.15919

13 MLDLQPETT 6.33500 6.56942 -0.23442

14 LLSSNLSWL 6.34200 6.13158 0.21042

15 GLACHQLCA 6.38000 5.77390 0.60610

16 LIGNESFAL 6.41500 7.18655 -0.77155

17 ALAKAAAAV 6.41900 6.67125 -0.25225

18 LLAVGATKV 6.47700 6.40227 0.07473

19 ALAKAAAAL 6.51100 6.71605 -0.20505

20 WILRGTSFV 6.55600 6.60504 -0.04904

21 IISCTCPTV 6.58000 6.64963 -0.06963

22 FLGGTPVCL 6.62300 6.90284 -0.27984

23 ALIHHNTHL 6.62300 6.99815 -0.37515

24 NLSWLSLDV 6.63900 6.02037 0.61863

25 YMIMVKCWM 6.66300 6.99100 -0.32800

26 VLQAGFFLL 6.68200 7.01061 -0.32861

27 GTLGIVCPI 6.71400 6.48554 0.22846

28 VILGVLLLI 6.78500 7.66353 -0.87853

29 VTWHRYHLL 6.79300 6.59116 0.20184

30 PLLPIFFCL 6.79600 7.53015 -0.73415

31 TLGIVCPIC 6.81500 6.11965 0.69535

32 CLTSTVQLV 6.83200 6.60500 0.22700

33 ILLLCLIFL 6.84500 6.70854 0.13646

34 FAFRDLCIV 6.88600 6.95323 -0.06723

35 FLEPGPVTA 6.89800 7.05578 -0.15778

36 ALAKAAAAA 6.94700 6.54597 0.40103

37 LMAVVLASL 6.95400 7.57201 -0.61801

38 YVITTQHWL 6.98300 6.69803 0.28497

39 LLCLIFLLV 6.99600 7.30927 -0.31327

40 ITAQVPFSV 7.02000 6.53580 0.48420

41 YLEPGPVTL 7.05800 7.35306 -0.29506

42 YTDQVPFSV 7.06600 6.79505 0.27095

43 NLYVSLLLL 7.11400 7.10247 0.01153

44 ILHNGAYSL 7.12700 7.50925 -0.38225

45 SIISAVVGI 7.15900 7.66820 -0.50920

46 VVMGTLVAL 7.17400 7.53897 -0.36497

47 YLEPGPVTI 7.18700 7.36221 -0.17521

48 GLSRYVARL 7.24800 7.63818 -0.39018

49 LLAQFTSAI 7.30100 7.60468 -0.30368

50 VLLDYQGML 7.32800 7.78211 -0.45411

51 YLEPGPVTV 7.34200 7.30826 0.03374

52 ILSPFMPLL 7.34700 7.23405 0.11295

53 YLSPGPVTA 7.38300 7.02993 0.35307

54 IIDQVPFSV 7.39800 7.59754 -0.19954

55 SVYDFFVWL 7.44400 7.80161 -0.35761

56 ITWQVPFSV 7.46300 7.39304 0.06996

57 ITYQVPFSV 7.48000 7.52837 -0.04837

58 GLYSSTVPV 7.48100 7.47219 0.00881

59 VMGTLVALV 7.55300 7.29262 0.26038

60 LLLCLIFLL 7.58500 7.23647 0.34853

61 SLDDYNHLV 7.58500 7.15143 0.43357

62 VLIQRNPQL 7.64400 7.25567 0.38833

63 SLYADSPSV 7.65800 7.67713 -0.01913

64 ILSQVPFSV 7.69900 7.52696 0.17204

65 IMDQVPFSV 7.71900 7.87238 -0.15338

66 QLFEDNYAL 7.76400 7.73113 0.03287

67 ALMDKSLHV 7.77000 7.40925 0.36075

68 YAIDLPVSV 7.79600 7.74089 0.05511

69 FVWLHYYSV 7.82400 8.35985 -0.53585

70 MLGTHTMEV 7.84500 7.22737 0.61763

71 LLFGYPVYV 7.88600 7.91594 -0.02994

72 ILKEPVHGV 7.92100 7.49973 0.42127

73 YLMPGPVTV 7.93200 7.81849 0.11351

74 WLDQVPFSV 7.93900 7.84572 0.09328

75 KTWGQYWQV 7.95500 7.33115 0.62385

76 ALMPLYACI 8.00000 7.38776 0.61224

77 YLAPGPVTA 8.03200 7.17507 0.85693

78 YLYPGPVTV 8.05100 8.29293 -0.24193

79 LLMGTLGIV 8.09700 7.21062 0.88638

80 YLWPGPVTV 8.12500 8.15760 -0.03260

81 FLLTRILTI 8.14900 8.23301 -0.08401

82 GLLGWSPQA 8.23700 7.81182 0.42518

83 ILYQVPFSV 8.31000 8.66468 -0.35468

84 GILTVILGV 8.34700 7.92060 0.42640

85 NMVPFFPPV 8.39800 7.94245 0.45555

86 ILDQVPFSV 8.48100 7.55281 0.92819

87 YLFPGPVTA 8.49500 7.96665 0.52835

88 YLDQVPFSV 8.63800 7.93136 0.70664

89 ILFQVPFSV 8.69900 8.46368 0.23532

90 ILWQVPFSV 8.77000 8.52935 0.24065

Correlation Coeffecient and Stand Error

CORL: 0.846041 CORL~2: 0.715786

RES: 0.423790 SEE: 0.044671

Statistical indices of Training calculation:

ITERATION (B): 8

No. Sequence Expt. Act Predicted Act Difference

1 VALVGLFVL 5.14800 5.98345 -0.83545

2 GTLVALVGL 5.34200 5.70543 -0.36343

3 LQTTIHDII 5.50100 6.03867 -0.53767

4 SLHVGTQCA 5.84200 5.69101 0.15099

5 ALPYWNFAT 5.86900 6.71906 -0.85006

6 SLNFMGYVI 5.88100 5.89863 -0.01763

7 NLQSLTNLL 6.00000 6.65476 -0.65476

8 FVTWHRYHL 6.02500 6.04062 -0.01562

9 DPKVKQWPL 6.17600 5.96502 0.21098

10 ITSQVPFSV 6.19600 6.27503 -0.07903

11 ALAKAAAAI 6.21100 6.55847 -0.34747

12 GLGQVPLIV 6.30100 6.46742 -0.16642

13 MLDLQPETT 6.33500 6.81151 -0.47651

14 LLSSNLSWL 6.34200 6.14474 0.19726

15 GLACHQLCA 6.38000 5.90049 0.47951

16 LIGNESFAL 6.41500 7.13211 -0.71711

17 ALAKAAAAV 6.41900 6.58682 -0.16782

18 LLAVGATKV 6.47700 6.31585 0.16115

19 ALAKAAAAL 6.51100 6.56328 -0.05228

20 WILRGTSFV 6.55600 6.63294 -0.07694

21 IISCTCPTV 6.58000 6.64202 -0.06202

22 FLGGTPVCL 6.62300 6.92597 -0.30297

23 ALIHHNTHL 6.62300 6.82564 -0.20264

24 NLSWLSLDV 6.63900 5.85634 0.78266

25 YMIMVKCWM 6.66300 6.94519 -0.28219

26 VLQAGFFLL 6.68200 7.11275 -0.43075

27 GTLGIVCPI 6.71400 6.26466 0.44934

28 VILGVLLLI 6.78500 7.68178 -0.89678

29 VTWHRYHLL 6.79300 6.49296 0.30004

30 PLLPIFFCL 6.79600 7.62508 -0.82908

31 TLGIVCPIC 6.81500 6.02425 0.79075

32 CLTSTVQLV 6.83200 6.65025 0.18175

33 ILLLCLIFL 6.84500 6.72801 0.11699

34 FAFRDLCIV 6.88600 6.94923 -0.06323

35 FLEPGPVTA 6.89800 7.26316 -0.36516

36 ALAKAAAAA 6.94700 6.65263 0.29437

37 LMAVVLASL 6.95400 7.54054 -0.58654

38 YVITTQHWL 6.98300 6.69511 0.28789

39 LLCLIFLLV 6.99600 7.43329 -0.43729

40 ITAQVPFSV 7.02000 6.42024 0.59976

41 YLEPGPVTL 7.05800 7.31986 -0.26186

42 YTDQVPFSV 7.06600 6.73555 0.33045

43 NLYVSLLLL 7.11400 7.03690 0.07710

44 ILHNGAYSL 7.12700 7.47595 -0.34895

45 SIISAVVGI 7.15900 7.57421 -0.41521

46 VVMGTLVAL 7.17400 7.48164 -0.30764

47 YLEPGPVTI 7.18700 7.31505 -0.12805

48 GLSRYVARL 7.24800 7.55842 -0.31042

49 LLAQFTSAI 7.30100 7.46580 -0.16480

50 VLLDYQGML 7.32800 7.73882 -0.41082

51 YLEPGPVTV 7.34200 7.34339 -0.00139

52 ILSPFMPLL 7.34700 7.26201 0.08499

53 YLSPGPVTA 7.38300 7.25609 0.12691

54 IIDQVPFSV 7.39800 7.65827 -0.26027

55 SVYDFFVWL 7.44400 7.86801 -0.42401

56 ITWQVPFSV 7.46300 7.27786 0.18514

57 ITYQVPFSV 7.48000 7.41326 0.06674

58 GLYSSTVPV 7.48100 7.40451 0.07649

59 VMGTLVALV 7.55300 7.38781 0.16519

60 LLLCLIFLL 7.58500 7.25760 0.32740

61 SLDDYNHLV 7.58500 7.12489 0.46011

62 VLIQRNPQL 7.64400 7.13555 0.50845

63 SLYADSPSV 7.65800 7.56216 0.09584

64 ILSQVPFSV 7.69900 7.58100 0.11800

65 IMDQVPFSV 7.71900 7.97414 -0.25514

66 QLFEDNYAL 7.76400 7.65398 0.11002

67 ALMDKSLHV 7.77000 7.35637 0.41363

68 YAIDLPVSV 7.79600 7.70252 0.09348

69 FVWLHYYSV 7.82400 8.52811 -0.70411

70 MLGTHTMEV 7.84500 7.23468 0.61032

71 LLFGYPVYV 7.88600 8.05146 -0.16546

72 ILKEPVHGV 7.92100 7.50499 0.41601

73 YLMPGPVTV 7.93200 7.85385 0.07815

74 WLDQVPFSV 7.93900 7.94318 -0.00418

75 KTWGQYWQV 7.95500 7.34709 0.60791

76 ALMPLYACI 8.00000 7.41547 0.58453

77 YLAPGPVTA 8.03200 7.40130 0.63070

78 YLYPGPVTV 8.05100 8.32851 -0.27751

79 LLMGTLGIV 8.09700 7.25641 0.84059

80 YLWPGPVTV 8.12500 8.19311 -0.06811

81 FLLTRILTI 8.14900 8.24753 -0.09853

82 GLLGWSPQA 8.23700 7.94992 0.28708

83 ILYQVPFSV 8.31000 8.71923 -0.40923

84 GILTVILGV 8.34700 7.94618 0.40082

85 NMVPFFPPV 8.39800 8.07571 0.32229

86 ILDQVPFSV 8.48100 7.60687 0.87413

87 YLFPGPVTA 8.49500 8.19324 0.30176

88 YLDQVPFSV 8.63800 8.04152 0.59648

89 ILFQVPFSV 8.69900 8.51815 0.18085

90 ILWQVPFSV 8.77000 8.58384 0.18616

Correlation Coeffecient and Stand Error

CORL: 0.858326 CORL~2: 0.736724

RES: 0.409522 SEE: 0.043167

Statistical indices of Training calculation:

ITERATION (A): 9

No. Sequence Expt. Act Predicted Act Difference

1 VALVGLFVL 5.14800 5.91648 -0.76848

2 GTLVALVGL 5.34200 5.93972 -0.59772

3 LQTTIHDII 5.50100 5.92069 -0.41969

4 SLHVGTQCA 5.84200 5.80856 0.03344

5 ALPYWNFAT 5.86900 6.66616 -0.79716

6 SLNFMGYVI 5.88100 6.13413 -0.25313

7 NLQSLTNLL 6.00000 6.76902 -0.76902

8 FVTWHRYHL 6.02500 5.91628 0.10872

9 DPKVKQWPL 6.17600 5.97384 0.20216

10 ITSQVPFSV 6.19600 6.40139 -0.20539

11 ALAKAAAAI 6.21100 6.41834 -0.20734

12 GLGQVPLIV 6.30100 6.51495 -0.21395

13 MLDLQPETT 6.33500 6.69732 -0.36232

14 LLSSNLSWL 6.34200 6.30100 0.04100

15 GLACHQLCA 6.38000 5.79166 0.58834

16 LIGNESFAL 6.41500 7.17420 -0.75920

17 ALAKAAAAV 6.41900 6.46496 -0.04596

18 LLAVGATKV 6.47700 6.43122 0.04578

19 ALAKAAAAL 6.51100 6.41518 0.09582

20 WILRGTSFV 6.55600 6.78429 -0.22829

21 IISCTCPTV 6.58000 6.57198 0.00802

22 FLGGTPVCL 6.62300 6.93723 -0.31423

23 ALIHHNTHL 6.62300 6.92787 -0.30487

24 NLSWLSLDV 6.63900 6.02437 0.61463

25 YMIMVKCWM 6.66300 6.97061 -0.30761

26 VLQAGFFLL 6.68200 7.08449 -0.40249

27 GTLGIVCPI 6.71400 6.47400 0.24000

28 VILGVLLLI 6.78500 7.56708 -0.78208

29 VTWHRYHLL 6.79300 6.53397 0.25903

30 PLLPIFFCL 6.79600 7.62619 -0.83019

31 TLGIVCPIC 6.81500 6.08189 0.73311

32 CLTSTVQLV 6.83200 6.87647 -0.04447

33 ILLLCLIFL 6.84500 6.90595 -0.06095

34 FAFRDLCIV 6.88600 6.68922 0.19678

35 FLEPGPVTA 6.89800 7.18968 -0.29168

36 ALAKAAAAA 6.94700 6.53051 0.41649

37 LMAVVLASL 6.95400 7.59506 -0.64106

38 YVITTQHWL 6.98300 6.48259 0.50041

39 LLCLIFLLV 6.99600 7.46228 -0.46628

40 ITAQVPFSV 7.02000 6.50840 0.51160

41 YLEPGPVTL 7.05800 7.27803 -0.22003

42 YTDQVPFSV 7.06600 6.88223 0.18377

43 NLYVSLLLL 7.11400 7.15261 -0.03861

44 ILHNGAYSL 7.12700 7.50031 -0.37331

45 SIISAVVGI 7.15900 7.43163 -0.27263

46 VVMGTLVAL 7.17400 7.38062 -0.20662

47 YLEPGPVTI 7.18700 7.28119 -0.09419

48 GLSRYVARL 7.24800 7.59914 -0.35114

49 LLAQFTSAI 7.30100 7.55433 -0.25333

50 VLLDYQGML 7.32800 7.76176 -0.43376

51 YLEPGPVTV 7.34200 7.32781 0.01419

52 ILSPFMPLL 7.34700 7.21361 0.13339

53 YLSPGPVTA 7.38300 7.20983 0.17317

54 IIDQVPFSV 7.39800 7.59678 -0.19878

55 SVYDFFVWL 7.44400 7.56915 -0.12515

56 ITWQVPFSV 7.46300 7.37540 0.08760

57 ITYQVPFSV 7.48000 7.57967 -0.09967

58 GLYSSTVPV 7.48100 7.54846 -0.06746

59 VMGTLVALV 7.55300 7.22377 0.32923

60 LLLCLIFLL 7.58500 7.30608 0.27892

61 SLDDYNHLV 7.58500 7.12925 0.45575

62 VLIQRNPQL 7.64400 7.14435 0.49965

63 SLYADSPSV 7.65800 7.71524 -0.05724

64 ILSQVPFSV 7.69900 7.58643 0.11257

65 IMDQVPFSV 7.71900 7.98533 -0.26633

66 QLFEDNYAL 7.76400 7.68191 0.08209

67 ALMDKSLHV 7.77000 7.49615 0.27385

68 YAIDLPVSV 7.79600 7.56849 0.22751

69 FVWLHYYSV 7.82400 8.15956 -0.33556

70 MLGTHTMEV 7.84500 7.25232 0.59268

71 LLFGYPVYV 7.88600 8.04386 -0.15786

72 ILKEPVHGV 7.92100 7.52312 0.39788

73 YLMPGPVTV 7.93200 7.87360 0.05840

74 WLDQVPFSV 7.93900 7.91891 0.02009

75 KTWGQYWQV 7.95500 7.47247 0.48253

76 ALMPLYACI 8.00000 7.45949 0.54051

77 YLAPGPVTA 8.03200 7.31684 0.71516

78 YLYPGPVTV 8.05100 8.32257 -0.27157

79 LLMGTLGIV 8.09700 7.49351 0.60349

80 YLWPGPVTV 8.12500 8.11830 0.00670

81 FLLTRILTI 8.14900 8.08081 0.06819

82 GLLGWSPQA 8.23700 8.00145 0.23555

83 ILYQVPFSV 8.31000 8.76471 -0.45471

84 GILTVILGV 8.34700 7.81430 0.53270

85 NMVPFFPPV 8.39800 7.98290 0.41510

86 ILDQVPFSV 8.48100 7.63058 0.85042

87 YLFPGPVTA 8.49500 8.10770 0.38730

88 YLDQVPFSV 8.63800 8.06727 0.57073

89 ILFQVPFSV 8.69900 8.48430 0.21470

90 ILWQVPFSV 8.77000 8.56045 0.20955

Correlation Coeffecient and Stand Error

CORL: 0.870214 CORL~2: 0.757273

RES: 0.391640 SEE: 0.041282

Statistical indices of Training calculation:

ITERATION (B): 9

No. Sequence Expt. Act Predicted Act Difference

1 VALVGLFVL 5.14800 5.77924 -0.63124

2 GTLVALVGL 5.34200 5.74200 -0.40000

3 LQTTIHDII 5.50100 5.75717 -0.25617

4 SLHVGTQCA 5.84200 5.90425 -0.06225

5 ALPYWNFAT 5.86900 6.73478 -0.86578

6 SLNFMGYVI 5.88100 6.00576 -0.12476

7 NLQSLTNLL 6.00000 6.65189 -0.65189

8 FVTWHRYHL 6.02500 5.78599 0.23901

9 DPKVKQWPL 6.17600 5.82204 0.35396

10 ITSQVPFSV 6.19600 6.35957 -0.16357

11 ALAKAAAAI 6.21100 6.26655 -0.05555

12 GLGQVPLIV 6.30100 6.55048 -0.24948

13 MLDLQPETT 6.33500 6.84107 -0.50607

14 LLSSNLSWL 6.34200 6.29905 0.04295

15 GLACHQLCA 6.38000 5.91288 0.46712

16 LIGNESFAL 6.41500 7.08116 -0.66616

17 ALAKAAAAV 6.41900 6.41101 0.00799

18 LLAVGATKV 6.47700 6.40763 0.06937

19 ALAKAAAAL 6.51100 6.25675 0.25425

20 WILRGTSFV 6.55600 6.81556 -0.25956

21 IISCTCPTV 6.58000 6.62124 -0.04124

22 FLGGTPVCL 6.62300 6.92188 -0.29888

23 ALIHHNTHL 6.62300 6.76660 -0.14360

24 NLSWLSLDV 6.63900 5.94298 0.69602

25 YMIMVKCWM 6.66300 6.90016 -0.23716

26 VLQAGFFLL 6.68200 7.13651 -0.45451

27 GTLGIVCPI 6.71400 6.30311 0.41089

28 VILGVLLLI 6.78500 7.56027 -0.77527

29 VTWHRYHLL 6.79300 6.47015 0.32285

30 PLLPIFFCL 6.79600 7.66449 -0.86849

31 TLGIVCPIC 6.81500 6.01453 0.80047

32 CLTSTVQLV 6.83200 6.93908 -0.10708

33 ILLLCLIFL 6.84500 6.89424 -0.04924

34 FAFRDLCIV 6.88600 6.69426 0.19174

35 FLEPGPVTA 6.89800 7.37262 -0.47462

36 ALAKAAAAA 6.94700 6.61411 0.33289

37 LMAVVLASL 6.95400 7.56777 -0.61377

38 YVITTQHWL 6.98300 6.40485 0.57815

39 LLCLIFLLV 6.99600 7.59025 -0.59425

40 ITAQVPFSV 7.02000 6.46465 0.55535

41 YLEPGPVTL 7.05800 7.23790 -0.17990

42 YTDQVPFSV 7.06600 6.88028 0.18572

43 NLYVSLLLL 7.11400 7.08660 0.02740

44 ILHNGAYSL 7.12700 7.43107 -0.30407

45 SIISAVVGI 7.15900 7.32886 -0.16986

46 VVMGTLVAL 7.17400 7.28406 -0.11006

47 YLEPGPVTI 7.18700 7.24770 -0.06070

48 GLSRYVARL 7.24800 7.50447 -0.25647

49 LLAQFTSAI 7.30100 7.44688 -0.14588

50 VLLDYQGML 7.32800 7.67830 -0.35030

51 YLEPGPVTV 7.34200 7.39216 -0.05016

52 ILSPFMPLL 7.34700 7.22646 0.12054

53 YLSPGPVTA 7.38300 7.41506 -0.03206

54 IIDQVPFSV 7.39800 7.65611 -0.25811

55 SVYDFFVWL 7.44400 7.53695 -0.09295

56 ITWQVPFSV 7.46300 7.31597 0.14703

57 ITYQVPFSV 7.48000 7.51654 -0.03654

58 GLYSSTVPV 7.48100 7.51097 -0.02997

59 VMGTLVALV 7.55300 7.29926 0.25374

60 LLLCLIFLL 7.58500 7.29497 0.29003

61 SLDDYNHLV 7.58500 7.11960 0.46540

62 VLIQRNPQL 7.64400 7.01291 0.63109

63 SLYADSPSV 7.65800 7.63291 0.02509

64 ILSQVPFSV 7.69900 7.64955 0.04945

65 IMDQVPFSV 7.71900 8.07906 -0.36006

66 QLFEDNYAL 7.76400 7.57072 0.19328

67 ALMDKSLHV 7.77000 7.45645 0.31355

68 YAIDLPVSV 7.79600 7.53710 0.25890

69 FVWLHYYSV 7.82400 8.29742 -0.47342

70 MLGTHTMEV 7.84500 7.28848 0.55652

71 LLFGYPVYV 7.88600 8.13798 -0.25198

72 ILKEPVHGV 7.92100 7.55017 0.37083

73 YLMPGPVTV 7.93200 7.92808 0.00392

74 WLDQVPFSV 7.93900 8.00808 -0.06908

75 KTWGQYWQV 7.95500 7.53677 0.41823

76 ALMPLYACI 8.00000 7.48714 0.51286

77 YLAPGPVTA 8.03200 7.52013 0.51187

78 YLYPGPVTV 8.05100 8.36893 -0.31793

79 LLMGTLGIV 8.09700 7.56555 0.53145

80 YLWPGPVTV 8.12500 8.16835 -0.04335

81 FLLTRILTI 8.14900 8.08016 0.06884

82 GLLGWSPQA 8.23700 8.09659 0.14041

83 ILYQVPFSV 8.31000 8.80652 -0.49652

84 GILTVILGV 8.34700 7.85042 0.49658

85 NMVPFFPPV 8.39800 8.10788 0.29012

86 ILDQVPFSV 8.48100 7.69290 0.78810

87 YLFPGPVTA 8.49500 8.29668 0.19832

88 YLDQVPFSV 8.63800 8.17026 0.46774

89 ILFQVPFSV 8.69900 8.53118 0.16782

90 ILWQVPFSV 8.77000 8.60595 0.16405

Correlation Coeffecient and Stand Error

CORL: 0.879201 CORL~2: 0.772994

RES: 0.380470 SEE: 0.040105

Statistical indices of Training calculation:

ITERATION (A): 10

No. Sequence Expt. Act Predicted Act Difference

1 VALVGLFVL 5.14800 5.78957 -0.64157

2 GTLVALVGL 5.34200 5.89643 -0.55443

3 LQTTIHDII 5.50100 5.76268 -0.26168

4 SLHVGTQCA 5.84200 5.98650 -0.14450

5 ALPYWNFAT 5.86900 6.69472 -0.82572

6 SLNFMGYVI 5.88100 6.11458 -0.23358

7 NLQSLTNLL 6.00000 6.71677 -0.71677

8 FVTWHRYHL 6.02500 5.82863 0.19637

9 DPKVKQWPL 6.17600 5.84588 0.33012

10 ITSQVPFSV 6.19600 6.47487 -0.27887

11 ALAKAAAAI 6.21100 6.25650 -0.04550

12 GLGQVPLIV 6.30100 6.58770 -0.28670

13 MLDLQPETT 6.33500 6.78210 -0.44710

14 LLSSNLSWL 6.34200 6.37985 -0.03785

15 GLACHQLCA 6.38000 5.91579 0.46421

16 LIGNESFAL 6.41500 7.08156 -0.66656

17 ALAKAAAAV 6.41900 6.40856 0.01044

18 LLAVGATKV 6.47700 6.47331 0.00369

19 ALAKAAAAL 6.51100 6.24233 0.26867

20 WILRGTSFV 6.55600 6.88998 -0.33398

21 IISCTCPTV 6.58000 6.62990 -0.04990

22 FLGGTPVCL 6.62300 6.93095 -0.30795

23 ALIHHNTHL 6.62300 6.82043 -0.19743

24 NLSWLSLDV 6.63900 6.05476 0.58424

25 YMIMVKCWM 6.66300 6.93351 -0.27051

26 VLQAGFFLL 6.68200 7.11929 -0.43729

27 GTLGIVCPI 6.71400 6.44414 0.26986

28 VILGVLLLI 6.78500 7.52678 -0.74178

29 VTWHRYHLL 6.79300 6.54670 0.24630

30 PLLPIFFCL 6.79600 7.62501 -0.82901

31 TLGIVCPIC 6.81500 6.08067 0.73433

32 CLTSTVQLV 6.83200 7.05454 -0.22254

33 ILLLCLIFL 6.84500 6.96120 -0.11620

34 FAFRDLCIV 6.88600 6.63469 0.25131

35 FLEPGPVTA 6.89800 7.32918 -0.43118

36 ALAKAAAAA 6.94700 6.60743 0.33957

37 LMAVVLASL 6.95400 7.57370 -0.61970

38 YVITTQHWL 6.98300 6.38113 0.60187

39 LLCLIFLLV 6.99600 7.59065 -0.59465

40 ITAQVPFSV 7.02000 6.56707 0.45293

41 YLEPGPVTL 7.05800 7.19951 -0.14151

42 YTDQVPFSV 7.06600 6.97775 0.08825

43 NLYVSLLLL 7.11400 7.11080 0.00320

44 ILHNGAYSL 7.12700 7.41339 -0.28639

45 SIISAVVGI 7.15900 7.29147 -0.13247

46 VVMGTLVAL 7.17400 7.27998 -0.10598

47 YLEPGPVTI 7.18700 7.21369 -0.02669

48 GLSRYVARL 7.24800 7.49987 -0.25187

49 LLAQFTSAI 7.30100 7.46315 -0.16215

50 VLLDYQGML 7.32800 7.66287 -0.33487

51 YLEPGPVTV 7.34200 7.36575 -0.02375

52 ILSPFMPLL 7.34700 7.20742 0.13958

53 YLSPGPVTA 7.38300 7.38684 -0.00384

54 IIDQVPFSV 7.39800 7.62707 -0.22907

55 SVYDFFVWL 7.44400 7.44736 -0.00336

56 ITWQVPFSV 7.46300 7.37840 0.08460

57 ITYQVPFSV 7.48000 7.60015 -0.12015

58 GLYSSTVPV 7.48100 7.55951 -0.07851

59 VMGTLVALV 7.55300 7.24587 0.30713

60 LLLCLIFLL 7.58500 7.28507 0.29993

61 SLDDYNHLV 7.58500 7.12539 0.45961

62 VLIQRNPQL 7.64400 7.01209 0.63191

63 SLYADSPSV 7.65800 7.65506 0.00294

64 ILSQVPFSV 7.69900 7.63596 0.06304

65 IMDQVPFSV 7.71900 8.06058 -0.34158

66 QLFEDNYAL 7.76400 7.55629 0.20771

67 ALMDKSLHV 7.77000 7.49627 0.27373

68 YAIDLPVSV 7.79600 7.49352 0.30248

69 FVWLHYYSV 7.82400 8.14901 -0.32501

70 MLGTHTMEV 7.84500 7.28932 0.55568

71 LLFGYPVYV 7.88600 8.10052 -0.21452

72 ILKEPVHGV 7.92100 7.54825 0.37275

73 YLMPGPVTV 7.93200 7.89942 0.03258

74 WLDQVPFSV 7.93900 7.95954 -0.02054

75 KTWGQYWQV 7.95500 7.59347 0.36153

76 ALMPLYACI 8.00000 7.48053 0.51947

77 YLAPGPVTA 8.03200 7.47904 0.55296

78 YLYPGPVTV 8.05100 8.31325 -0.26225

79 LLMGTLGIV 8.09700 7.63885 0.45815

80 YLWPGPVTV 8.12500 8.09150 0.03350

81 FLLTRILTI 8.14900 7.95389 0.19511

82 GLLGWSPQA 8.23700 8.06768 0.16932

83 ILYQVPFSV 8.31000 8.76124 -0.45124

84 GILTVILGV 8.34700 7.78246 0.56454

85 NMVPFFPPV 8.39800 8.06063 0.33737

86 ILDQVPFSV 8.48100 7.68032 0.80068

87 YLFPGPVTA 8.49500 8.22094 0.27406

88 YLDQVPFSV 8.63800 8.13884 0.49916

89 ILFQVPFSV 8.69900 8.47006 0.22894

90 ILWQVPFSV 8.77000 8.53949 0.23051

Correlation Coeffecient and Stand Error

CORL: 0.881051 CORL~2: 0.776251

RES: 0.376018 SEE: 0.039636

Statistical indices of Training calculation:

ITERATION (B): 10

No. Sequence Expt. Act Predicted Act Difference

1 VALVGLFVL 5.14800 5.70974 -0.56174

2 GTLVALVGL 5.34200 5.82889 -0.48689

3 LQTTIHDII 5.50100 5.68566 -0.18466

4 SLHVGTQCA 5.84200 5.99070 -0.14870

5 ALPYWNFAT 5.86900 6.70016 -0.83116

6 SLNFMGYVI 5.88100 6.03794 -0.15694

7 NLQSLTNLL 6.00000 6.66908 -0.66908

8 FVTWHRYHL 6.02500 5.75253 0.27247

9 DPKVKQWPL 6.17600 5.76122 0.41478

10 ITSQVPFSV 6.19600 6.45452 -0.25852

11 ALAKAAAAI 6.21100 6.20824 0.00276

12 GLGQVPLIV 6.30100 6.57078 -0.26978

13 MLDLQPETT 6.33500 6.82023 -0.48523

14 LLSSNLSWL 6.34200 6.34162 0.00038

15 GLACHQLCA 6.38000 5.91420 0.46580

16 LIGNESFAL 6.41500 7.04777 -0.63277

17 ALAKAAAAV 6.41900 6.39475 0.02425

18 LLAVGATKV 6.47700 6.46466 0.01234

19 ALAKAAAAL 6.51100 6.19085 0.32015

20 WILRGTSFV 6.55600 6.88735 -0.33135

21 IISCTCPTV 6.58000 6.63885 -0.05885

22 FLGGTPVCL 6.62300 6.90469 -0.28169

23 ALIHHNTHL 6.62300 6.77878 -0.15578

24 NLSWLSLDV 6.63900 6.01999 0.61901

25 YMIMVKCWM 6.66300 6.87830 -0.21530

26 VLQAGFFLL 6.68200 7.10075 -0.41875

27 GTLGIVCPI 6.71400 6.37748 0.33652

28 VILGVLLLI 6.78500 7.51244 -0.72744

29 VTWHRYHLL 6.79300 6.50889 0.28411

30 PLLPIFFCL 6.79600 7.61373 -0.81773

31 TLGIVCPIC 6.81500 6.02451 0.79049

32 CLTSTVQLV 6.83200 7.06348 -0.23148

33 ILLLCLIFL 6.84500 6.92516 -0.08016

34 FAFRDLCIV 6.88600 6.61580 0.27020

35 FLEPGPVTA 6.89800 7.39344 -0.49544

36 ALAKAAAAA 6.94700 6.63866 0.30834

37 LMAVVLASL 6.95400 7.58540 -0.63140

38 YVITTQHWL 6.98300 6.32401 0.65899

39 LLCLIFLLV 6.99600 7.61762 -0.62162

40 ITAQVPFSV 7.02000 6.54861 0.47139

41 YLEPGPVTL 7.05800 7.19166 -0.13366

42 YTDQVPFSV 7.06600 6.97892 0.08708

43 NLYVSLLLL 7.11400 7.07655 0.03745

44 ILHNGAYSL 7.12700 7.38993 -0.26293

45 SIISAVVGI 7.15900 7.27269 -0.11369

46 VVMGTLVAL 7.17400 7.26019 -0.08619

47 YLEPGPVTI 7.18700 7.20904 -0.02204

48 GLSRYVARL 7.24800 7.48542 -0.23742

49 LLAQFTSAI 7.30100 7.45436 -0.15336

50 VLLDYQGML 7.32800 7.63803 -0.31003

51 YLEPGPVTV 7.34200 7.39555 -0.05355

52 ILSPFMPLL 7.34700 7.19274 0.15426

53 YLSPGPVTA 7.38300 7.45805 -0.07505

54 IIDQVPFSV 7.39800 7.65222 -0.25422

55 SVYDFFVWL 7.44400 7.41393 0.03007

56 ITWQVPFSV 7.46300 7.37652 0.08648

57 ITYQVPFSV 7.48000 7.60280 -0.12280

58 GLYSSTVPV 7.48100 7.56498 -0.08398

59 VMGTLVALV 7.55300 7.26091 0.29209

60 LLLCLIFLL 7.58500 7.25901 0.32599

61 SLDDYNHLV 7.58500 7.11534 0.46966

62 VLIQRNPQL 7.64400 6.97735 0.66665

63 SLYADSPSV 7.65800 7.66327 -0.00527

64 ILSQVPFSV 7.69900 7.66235 0.03665

65 IMDQVPFSV 7.71900 8.10318 -0.38418

66 QLFEDNYAL 7.76400 7.52508 0.23892

67 ALMDKSLHV 7.77000 7.49746 0.27254

68 YAIDLPVSV 7.79600 7.50740 0.28860

69 FVWLHYYSV 7.82400 8.19971 -0.37571

70 MLGTHTMEV 7.84500 7.30181 0.54319

71 LLFGYPVYV 7.88600 8.11570 -0.22970

72 ILKEPVHGV 7.92100 7.57802 0.34298

73 YLMPGPVTV 7.93200 7.94013 -0.00813

74 WLDQVPFSV 7.93900 7.99939 -0.06039

75 KTWGQYWQV 7.95500 7.61386 0.34114

76 ALMPLYACI 8.00000 7.48131 0.51869

77 YLAPGPVTA 8.03200 7.55213 0.47987

78 YLYPGPVTV 8.05100 8.36242 -0.31142

79 LLMGTLGIV 8.09700 7.66844 0.42856

80 YLWPGPVTV 8.12500 8.13613 -0.01113

81 FLLTRILTI 8.14900 7.96659 0.18241

82 GLLGWSPQA 8.23700 8.12332 0.11368

83 ILYQVPFSV 8.31000 8.81063 -0.50063

84 GILTVILGV 8.34700 7.80974 0.53726

85 NMVPFFPPV 8.39800 8.10616 0.29184

86 ILDQVPFSV 8.48100 7.70761 0.77339

87 YLFPGPVTA 8.49500 8.30920 0.18580

88 YLDQVPFSV 8.63800 8.18675 0.45125

89 ILFQVPFSV 8.69900 8.51350 0.18550

90 ILWQVPFSV 8.77000 8.58435 0.18565

Correlation Coeffecient and Stand Error

CORL: 0.883078 CORL~2: 0.779826

RES: 0.374025 SEE: 0.039426

Statistical indices of Training calculation:

ITERATION (A): 11

No. Sequence Expt. Act Predicted Act Difference

1 VALVGLFVL 5.14800 5.75115 -0.60315

2 GTLVALVGL 5.34200 5.90102 -0.55902

3 LQTTIHDII 5.50100 5.73721 -0.23621

4 SLHVGTQCA 5.84200 6.03819 -0.19619

5 ALPYWNFAT 5.86900 6.70395 -0.83495

6 SLNFMGYVI 5.88100 6.08532 -0.20432

7 NLQSLTNLL 6.00000 6.70128 -0.70128

8 FVTWHRYHL 6.02500 5.81893 0.20607

9 DPKVKQWPL 6.17600 5.80414 0.37186

10 ITSQVPFSV 6.19600 6.50188 -0.30588

11 ALAKAAAAI 6.21100 6.23633 -0.02533

12 GLGQVPLIV 6.30100 6.58657 -0.28557

13 MLDLQPETT 6.33500 6.81732 -0.48232

14 LLSSNLSWL 6.34200 6.38110 -0.03910

15 GLACHQLCA 6.38000 5.95586 0.42414

16 LIGNESFAL 6.41500 7.05344 -0.63844

17 ALAKAAAAV 6.41900 6.41696 0.00204

18 LLAVGATKV 6.47700 6.48931 -0.01231

19 ALAKAAAAL 6.51100 6.22017 0.29083

20 WILRGTSFV 6.55600 6.90644 -0.35044

21 IISCTCPTV 6.58000 6.66007 -0.08007

22 FLGGTPVCL 6.62300 6.91999 -0.29699

23 ALIHHNTHL 6.62300 6.80302 -0.18002

24 NLSWLSLDV 6.63900 6.07148 0.56752

25 YMIMVKCWM 6.66300 6.89971 -0.23671

26 VLQAGFFLL 6.68200 7.10457 -0.42257

27 GTLGIVCPI 6.71400 6.43862 0.27538

28 VILGVLLLI 6.78500 7.50978 -0.72478

29 VTWHRYHLL 6.79300 6.55665 0.23635

30 PLLPIFFCL 6.79600 7.59864 -0.80264

31 TLGIVCPIC 6.81500 6.06983 0.74517

32 CLTSTVQLV 6.83200 7.09165 -0.25965

33 ILLLCLIFL 6.84500 6.94464 -0.09964

34 FAFRDLCIV 6.88600 6.61877 0.26723

35 FLEPGPVTA 6.89800 7.37804 -0.48004

36 ALAKAAAAA 6.94700 6.65528 0.29172

37 LMAVVLASL 6.95400 7.57630 -0.62230

38 YVITTQHWL 6.98300 6.35999 0.62301

39 LLCLIFLLV 6.99600 7.60394 -0.60794

40 ITAQVPFSV 7.02000 6.59136 0.42864

41 YLEPGPVTL 7.05800 7.18363 -0.12563

42 YTDQVPFSV 7.06600 7.00674 0.05926

43 NLYVSLLLL 7.11400 7.08151 0.03249

44 ILHNGAYSL 7.12700 7.38045 -0.25345

45 SIISAVVGI 7.15900 7.27602 -0.11702

46 VVMGTLVAL 7.17400 7.27773 -0.10373

47 YLEPGPVTI 7.18700 7.19980 -0.01280

48 GLSRYVARL 7.24800 7.47180 -0.22380

49 LLAQFTSAI 7.30100 7.44797 -0.14697

50 VLLDYQGML 7.32800 7.62622 -0.29822

51 YLEPGPVTV 7.34200 7.38042 -0.03842

52 ILSPFMPLL 7.34700 7.19463 0.15237

53 YLSPGPVTA 7.38300 7.44377 -0.06077

54 IIDQVPFSV 7.39800 7.63615 -0.23815

55 SVYDFFVWL 7.44400 7.40751 0.03649

56 ITWQVPFSV 7.46300 7.38767 0.07533

57 ITYQVPFSV 7.48000 7.61115 -0.13115

58 GLYSSTVPV 7.48100 7.56364 -0.08264

59 VMGTLVALV 7.55300 7.25025 0.30275

60 LLLCLIFLL 7.58500 7.25772 0.32728

61 SLDDYNHLV 7.58500 7.11866 0.46634

62 VLIQRNPQL 7.64400 6.98561 0.65839

63 SLYADSPSV 7.65800 7.64962 0.00838

64 ILSQVPFSV 7.69900 7.64394 0.05506

65 IMDQVPFSV 7.71900 8.07307 -0.35407

66 QLFEDNYAL 7.76400 7.51709 0.24691

67 ALMDKSLHV 7.77000 7.49301 0.27699

68 YAIDLPVSV 7.79600 7.49249 0.30351

69 FVWLHYYSV 7.82400 8.15600 -0.33200

70 MLGTHTMEV 7.84500 7.29468 0.55032

71 LLFGYPVYV 7.88600 8.08311 -0.19711

72 ILKEPVHGV 7.92100 7.56898 0.35202

73 YLMPGPVTV 7.93200 7.90930 0.02270

74 WLDQVPFSV 7.93900 7.96375 -0.02475

75 KTWGQYWQV 7.95500 7.62037 0.33463

76 ALMPLYACI 8.00000 7.47489 0.52511

77 YLAPGPVTA 8.03200 7.53325 0.49875

78 YLYPGPVTV 8.05100 8.31472 -0.26372

79 LLMGTLGIV 8.09700 7.66664 0.43036

80 YLWPGPVTV 8.12500 8.09125 0.03375

81 FLLTRILTI 8.14900 7.91936 0.22964

82 GLLGWSPQA 8.23700 8.08887 0.14813

83 ILYQVPFSV 8.31000 8.75320 -0.44320

84 GILTVILGV 8.34700 7.78327 0.56373

85 NMVPFFPPV 8.39800 8.08000 0.31800

86 ILDQVPFSV 8.48100 7.68765 0.79335

87 YLFPGPVTA 8.49500 8.26234 0.23266

88 YLDQVPFSV 8.63800 8.14879 0.48921

89 ILFQVPFSV 8.69900 8.46250 0.23650

90 ILWQVPFSV 8.77000 8.52973 0.24027

Correlation Coeffecient and Stand Error

CORL: 0.883194 CORL~2: 0.780032

RES: 0.372828 SEE: 0.039299

Statistical indices of Training calculation:

ITERATION (B): 11

No. Sequence Expt. Act Predicted Act Difference

1 VALVGLFVL 5.14800 5.69524 -0.54724

2 GTLVALVGL 5.34200 5.85760 -0.51560

3 LQTTIHDII 5.50100 5.68937 -0.18837

4 SLHVGTQCA 5.84200 6.02273 -0.18073

5 ALPYWNFAT 5.86900 6.69092 -0.82192

6 SLNFMGYVI 5.88100 6.02801 -0.14701

7 NLQSLTNLL 6.00000 6.67823 -0.67823

8 FVTWHRYHL 6.02500 5.75778 0.26722

9 DPKVKQWPL 6.17600 5.74815 0.42785

10 ITSQVPFSV 6.19600 6.48926 -0.29326

11 ALAKAAAAI 6.21100 6.20651 0.00449

12 GLGQVPLIV 6.30100 6.56788 -0.26688

13 MLDLQPETT 6.33500 6.82256 -0.48756

14 LLSSNLSWL 6.34200 6.34703 -0.00503

15 GLACHQLCA 6.38000 5.93099 0.44901

16 LIGNESFAL 6.41500 7.04071 -0.62571

17 ALAKAAAAV 6.41900 6.40527 0.01373

18 LLAVGATKV 6.47700 6.47588 0.00112

19 ALAKAAAAL 6.51100 6.18873 0.32227

20 WILRGTSFV 6.55600 6.90062 -0.34462

21 IISCTCPTV 6.58000 6.64700 -0.06700

22 FLGGTPVCL 6.62300 6.90144 -0.27844

23 ALIHHNTHL 6.62300 6.78555 -0.16255

24 NLSWLSLDV 6.63900 6.03653 0.60247

25 YMIMVKCWM 6.66300 6.85476 -0.19176

26 VLQAGFFLL 6.68200 7.08514 -0.40314

27 GTLGIVCPI 6.71400 6.40810 0.30590

28 VILGVLLLI 6.78500 7.50494 -0.71994

29 VTWHRYHLL 6.79300 6.52749 0.26551

30 PLLPIFFCL 6.79600 7.58763 -0.79163

31 TLGIVCPIC 6.81500 6.01826 0.79674

32 CLTSTVQLV 6.83200 7.09414 -0.26214

33 ILLLCLIFL 6.84500 6.91203 -0.06703

34 FAFRDLCIV 6.88600 6.60419 0.28181

35 FLEPGPVTA 6.89800 7.40602 -0.50802

36 ALAKAAAAA 6.94700 6.66750 0.27950

37 LMAVVLASL 6.95400 7.58183 -0.62783

38 YVITTQHWL 6.98300 6.32214 0.66086

39 LLCLIFLLV 6.99600 7.60739 -0.61139

40 ITAQVPFSV 7.02000 6.58107 0.43893

41 YLEPGPVTL 7.05800 7.17514 -0.11714

42 YTDQVPFSV 7.06600 7.00903 0.05697

43 NLYVSLLLL 7.11400 7.05841 0.05559

44 ILHNGAYSL 7.12700 7.37506 -0.24806

45 SIISAVVGI 7.15900 7.27530 -0.11630

46 VVMGTLVAL 7.17400 7.27553 -0.10153

47 YLEPGPVTI 7.18700 7.19292 -0.00592

48 GLSRYVARL 7.24800 7.47488 -0.22688

49 LLAQFTSAI 7.30100 7.45294 -0.15194

50 VLLDYQGML 7.32800 7.62566 -0.29766

51 YLEPGPVTV 7.34200 7.39168 -0.04968

52 ILSPFMPLL 7.34700 7.17861 0.16839

53 YLSPGPVTA 7.38300 7.47439 -0.09139

54 IIDQVPFSV 7.39800 7.65337 -0.25537

55 SVYDFFVWL 7.44400 7.39454 0.04946

56 ITWQVPFSV 7.46300 7.39809 0.06491

57 ITYQVPFSV 7.48000 7.62738 -0.14738

58 GLYSSTVPV 7.48100 7.57884 -0.09784

59 VMGTLVALV 7.55300 7.25441 0.29859

60 LLLCLIFLL 7.58500 7.23460 0.35040

61 SLDDYNHLV 7.58500 7.11939 0.46561

62 VLIQRNPQL 7.64400 6.97158 0.67242

63 SLYADSPSV 7.65800 7.67114 -0.01314

64 ILSQVPFSV 7.69900 7.66137 0.03763

65 IMDQVPFSV 7.71900 8.10179 -0.38279

66 QLFEDNYAL 7.76400 7.51123 0.25277

67 ALMDKSLHV 7.77000 7.50650 0.26350

68 YAIDLPVSV 7.79600 7.51409 0.28191

69 FVWLHYYSV 7.82400 8.17921 -0.35521

70 MLGTHTMEV 7.84500 7.30172 0.54328

71 LLFGYPVYV 7.88600 8.10021 -0.21421

72 ILKEPVHGV 7.92100 7.58996 0.33104

73 YLMPGPVTV 7.93200 7.93430 -0.00230

74 WLDQVPFSV 7.93900 7.99057 -0.05157

75 KTWGQYWQV 7.95500 7.63503 0.31997

76 ALMPLYACI 8.00000 7.46806 0.53194

77 YLAPGPVTA 8.03200 7.56619 0.46581

78 YLYPGPVTV 8.05100 8.35027 -0.29927

79 LLMGTLGIV 8.09700 7.68513 0.41187

80 YLWPGPVTV 8.12500 8.12098 0.00402

81 FLLTRILTI 8.14900 7.93116 0.21784

82 GLLGWSPQA 8.23700 8.13738 0.09962

83 ILYQVPFSV 8.31000 8.79949 -0.48949

84 GILTVILGV 8.34700 7.80471 0.54229

85 NMVPFFPPV 8.39800 8.10427 0.29373

86 ILDQVPFSV 8.48100 7.70623 0.77477

87 YLFPGPVTA 8.49500 8.31424 0.18076

88 YLDQVPFSV 8.63800 8.18114 0.45686

89 ILFQVPFSV 8.69900 8.50123 0.19777

90 ILWQVPFSV 8.77000 8.57020 0.19980

Correlation Coeffecient and Stand Error

CORL: 0.884238 CORL~2: 0.781877

RES: 0.371910 SEE: 0.039203

Statistical indices of Training calculation:

ITERATION (A): 12

No. Sequence Expt. Act Predicted Act Difference

1 VALVGLFVL 5.14800 5.73685 -0.58885

2 GTLVALVGL 5.34200 5.90455 -0.56255

3 LQTTIHDII 5.50100 5.73906 -0.23806

4 SLHVGTQCA 5.84200 6.06179 -0.21979

5 ALPYWNFAT 5.86900 6.70189 -0.83289

6 SLNFMGYVI 5.88100 6.06491 -0.18391

7 NLQSLTNLL 6.00000 6.69955 -0.69955

8 FVTWHRYHL 6.02500 5.80914 0.21586

9 DPKVKQWPL 6.17600 5.78809 0.38791

10 ITSQVPFSV 6.19600 6.51427 -0.31827

11 ALAKAAAAI 6.21100 6.23507 -0.02407

12 GLGQVPLIV 6.30100 6.58106 -0.28006

13 MLDLQPETT 6.33500 6.83127 -0.49627

14 LLSSNLSWL 6.34200 6.37605 -0.03405

15 GLACHQLCA 6.38000 5.97381 0.40619

16 LIGNESFAL 6.41500 7.04765 -0.63265

17 ALAKAAAAV 6.41900 6.42784 -0.00884

18 LLAVGATKV 6.47700 6.49582 -0.01882

19 ALAKAAAAL 6.51100 6.21843 0.29257

20 WILRGTSFV 6.55600 6.91028 -0.35428

21 IISCTCPTV 6.58000 6.66643 -0.08643

22 FLGGTPVCL 6.62300 6.91336 -0.29036

23 ALIHHNTHL 6.62300 6.80100 -0.17800

24 NLSWLSLDV 6.63900 6.07412 0.56488

25 YMIMVKCWM 6.66300 6.86923 -0.20623

26 VLQAGFFLL 6.68200 7.09087 -0.40887

27 GTLGIVCPI 6.71400 6.44363 0.27037

28 VILGVLLLI 6.78500 7.50211 -0.71711

29 VTWHRYHLL 6.79300 6.55705 0.23595

30 PLLPIFFCL 6.79600 7.58027 -0.78427

31 TLGIVCPIC 6.81500 6.05386 0.76114

32 CLTSTVQLV 6.83200 7.10293 -0.27093

33 ILLLCLIFL 6.84500 6.92311 -0.07811

34 FAFRDLCIV 6.88600 6.61541 0.27059

35 FLEPGPVTA 6.89800 7.40040 -0.50240

36 ALAKAAAAA 6.94700 6.68365 0.26335

37 LMAVVLASL 6.95400 7.57224 -0.61824

38 YVITTQHWL 6.98300 6.35436 0.62864

39 LLCLIFLLV 6.99600 7.59500 -0.59900

40 ITAQVPFSV 7.02000 6.60259 0.41741

41 YLEPGPVTL 7.05800 7.17665 -0.11865

42 YTDQVPFSV 7.06600 7.01844 0.04756

43 NLYVSLLLL 7.11400 7.06411 0.04989

44 ILHNGAYSL 7.12700 7.36958 -0.24258

45 SIISAVVGI 7.15900 7.27829 -0.11929

46 VVMGTLVAL 7.17400 7.28333 -0.10933

47 YLEPGPVTI 7.18700 7.19329 -0.00629

48 GLSRYVARL 7.24800 7.46292 -0.21492

49 LLAQFTSAI 7.30100 7.44532 -0.14432

50 VLLDYQGML 7.32800 7.61521 -0.28721

51 YLEPGPVTV 7.34200 7.38606 -0.04406

52 ILSPFMPLL 7.34700 7.18381 0.16319

53 YLSPGPVTA 7.38300 7.46733 -0.08433

54 IIDQVPFSV 7.39800 7.63957 -0.24157

55 SVYDFFVWL 7.44400 7.39192 0.05208

56 ITWQVPFSV 7.46300 7.39489 0.06811

57 ITYQVPFSV 7.48000 7.61860 -0.13860

58 GLYSSTVPV 7.48100 7.57276 -0.09176

59 VMGTLVALV 7.55300 7.25164 0.30136

60 LLLCLIFLL 7.58500 7.23722 0.34778

61 SLDDYNHLV 7.58500 7.12349 0.46151

62 VLIQRNPQL 7.64400 6.97906 0.66494

63 SLYADSPSV 7.65800 7.65856 -0.00056

64 ILSQVPFSV 7.69900 7.64521 0.05379

65 IMDQVPFSV 7.71900 8.07412 -0.35512

66 QLFEDNYAL 7.76400 7.50506 0.25894

67 ALMDKSLHV 7.77000 7.49785 0.27215

68 YAIDLPVSV 7.79600 7.50208 0.29392

69 FVWLHYYSV 7.82400 8.15153 -0.32753

70 MLGTHTMEV 7.84500 7.29744 0.54756

71 LLFGYPVYV 7.88600 8.07346 -0.18746

72 ILKEPVHGV 7.92100 7.57873 0.34227

73 YLMPGPVTV 7.93200 7.91259 0.01941

74 WLDQVPFSV 7.93900 7.96345 -0.02445

75 KTWGQYWQV 7.95500 7.62988 0.32512

76 ALMPLYACI 8.00000 7.46628 0.53372

77 YLAPGPVTA 8.03200 7.55565 0.47635

78 YLYPGPVTV 8.05100 8.31585 -0.26485

79 LLMGTLGIV 8.09700 7.67512 0.42188

80 YLWPGPVTV 8.12500 8.09215 0.03285

81 FLLTRILTI 8.14900 7.90685 0.24215

82 GLLGWSPQA 8.23700 8.10897 0.12803

83 ILYQVPFSV 8.31000 8.74954 -0.43954

84 GILTVILGV 8.34700 7.78810 0.55890

85 NMVPFFPPV 8.39800 8.08497 0.31303

86 ILDQVPFSV 8.48100 7.68903 0.79197

87 YLFPGPVTA 8.49500 8.28112 0.21388

88 YLDQVPFSV 8.63800 8.14938 0.48862

89 ILFQVPFSV 8.69900 8.45900 0.24000

90 ILWQVPFSV 8.77000 8.52584 0.24416

Correlation Coeffecient and Stand Error

CORL: 0.884272 CORL~2: 0.781938

RES: 0.371209 SEE: 0.039129

Statistical indices of Training calculation:

ITERATION (B): 12

No. Sequence Expt. Act Predicted Act Difference

1 VALVGLFVL 5.14800 5.69543 -0.54743

2 GTLVALVGL 5.34200 5.87198 -0.52998

3 LQTTIHDII 5.50100 5.70367 -0.20267

4 SLHVGTQCA 5.84200 6.04301 -0.20101

5 ALPYWNFAT 5.86900 6.68542 -0.81642

6 SLNFMGYVI 5.88100 6.01817 -0.13717

7 NLQSLTNLL 6.00000 6.68341 -0.68341

8 FVTWHRYHL 6.02500 5.75862 0.26638

9 DPKVKQWPL 6.17600 5.74560 0.43040

10 ITSQVPFSV 6.19600 6.50708 -0.31108

11 ALAKAAAAI 6.21100 6.20984 0.00116

12 GLGQVPLIV 6.30100 6.56492 -0.26392

13 MLDLQPETT 6.33500 6.82813 -0.49313

14 LLSSNLSWL 6.34200 6.34773 -0.00573

15 GLACHQLCA 6.38000 5.94704 0.43296

16 LIGNESFAL 6.41500 7.03995 -0.62495

17 ALAKAAAAV 6.41900 6.41544 0.00356

18 LLAVGATKV 6.47700 6.48077 -0.00377

19 ALAKAAAAL 6.51100 6.19209 0.31891

20 WILRGTSFV 6.55600 6.90305 -0.34705

21 IISCTCPTV 6.58000 6.65046 -0.07046

22 FLGGTPVCL 6.62300 6.89901 -0.27601

23 ALIHHNTHL 6.62300 6.78780 -0.16480

24 NLSWLSLDV 6.63900 6.04115 0.59785

25 YMIMVKCWM 6.66300 6.83064 -0.16764

26 VLQAGFFLL 6.68200 7.07641 -0.39441

27 GTLGIVCPI 6.71400 6.42627 0.28773

28 VILGVLLLI 6.78500 7.50113 -0.71613

29 VTWHRYHLL 6.79300 6.53650 0.25650

30 PLLPIFFCL 6.79600 7.57332 -0.77732

31 TLGIVCPIC 6.81500 6.00982 0.80518

32 CLTSTVQLV 6.83200 7.10381 -0.27181

33 ILLLCLIFL 6.84500 6.89612 -0.05112

34 FAFRDLCIV 6.88600 6.60654 0.27946

35 FLEPGPVTA 6.89800 7.41705 -0.51905

36 ALAKAAAAA 6.94700 6.68827 0.25873

37 LMAVVLASL 6.95400 7.57423 -0.62023

38 YVITTQHWL 6.98300 6.32517 0.65783

39 LLCLIFLLV 6.99600 7.59498 -0.59898

40 ITAQVPFSV 7.02000 6.59743 0.42257

41 YLEPGPVTL 7.05800 7.16803 -0.11003

42 YTDQVPFSV 7.06600 7.02309 0.04291

43 NLYVSLLLL 7.11400 7.04570 0.06830

44 ILHNGAYSL 7.12700 7.36822 -0.24122

45 SIISAVVGI 7.15900 7.28010 -0.12110

46 VVMGTLVAL 7.17400 7.28560 -0.11160

47 YLEPGPVTI 7.18700 7.18578 0.00122

48 GLSRYVARL 7.24800 7.46865 -0.22065

49 LLAQFTSAI 7.30100 7.45062 -0.14962

50 VLLDYQGML 7.32800 7.61910 -0.29110

51 YLEPGPVTV 7.34200 7.39137 -0.04937

52 ILSPFMPLL 7.34700 7.17136 0.17564

53 YLSPGPVTA 7.38300 7.48567 -0.10267

54 IIDQVPFSV 7.39800 7.65365 -0.25565

55 SVYDFFVWL 7.44400 7.38772 0.05628

56 ITWQVPFSV 7.46300 7.40789 0.05511

57 ITYQVPFSV 7.48000 7.63672 -0.15672

58 GLYSSTVPV 7.48100 7.58608 -0.10508

59 VMGTLVALV 7.55300 7.25236 0.30064

60 LLLCLIFLL 7.58500 7.21865 0.36635

61 SLDDYNHLV 7.58500 7.12622 0.45878

62 VLIQRNPQL 7.64400 6.96854 0.67546

63 SLYADSPSV 7.65800 7.67679 -0.01879

64 ILSQVPFSV 7.69900 7.65920 0.03980

65 IMDQVPFSV 7.71900 8.09632 -0.37732

66 QLFEDNYAL 7.76400 7.50404 0.25996

67 ALMDKSLHV 7.77000 7.51036 0.25964

68 YAIDLPVSV 7.79600 7.52435 0.27165

69 FVWLHYYSV 7.82400 8.16994 -0.34594

70 MLGTHTMEV 7.84500 7.30216 0.54284

71 LLFGYPVYV 7.88600 8.09037 -0.20437

72 ILKEPVHGV 7.92100 7.59419 0.32681

73 YLMPGPVTV 7.93200 7.92997 0.00203

74 WLDQVPFSV 7.93900 7.98491 -0.04591

75 KTWGQYWQV 7.95500 7.64649 0.30851

76 ALMPLYACI 8.00000 7.46056 0.53944

77 YLAPGPVTA 8.03200 7.57602 0.45598

78 YLYPGPVTV 8.05100 8.34247 -0.29147

79 LLMGTLGIV 8.09700 7.68893 0.40807

80 YLWPGPVTV 8.12500 8.11364 0.01136

81 FLLTRILTI 8.14900 7.91710 0.23190

82 GLLGWSPQA 8.23700 8.14893 0.08807

83 ILYQVPFSV 8.31000 8.78884 -0.47884

84 GILTVILGV 8.34700 7.80597 0.54103

85 NMVPFFPPV 8.39800 8.10312 0.29488

86 ILDQVPFSV 8.48100 7.70402 0.77698

87 YLFPGPVTA 8.49500 8.31811 0.17689

88 YLDQVPFSV 8.63800 8.17521 0.46279

89 ILFQVPFSV 8.69900 8.49164 0.20736

90 ILWQVPFSV 8.77000 8.56001 0.20999

Correlation Coeffecient and Stand Error

CORL: 0.884957 CORL~2: 0.783149

RES: 0.370617 SEE: 0.039067

Statistical indices of Training calculation:

ITERATION (A): 13

No. Sequence Expt. Act Predicted Act Difference

1 VALVGLFVL 5.14800 5.73248 -0.58448

2 GTLVALVGL 5.34200 5.90757 -0.56557

3 LQTTIHDII 5.50100 5.74594 -0.24494

4 SLHVGTQCA 5.84200 6.07658 -0.23458

5 ALPYWNFAT 5.86900 6.69668 -0.82768

6 SLNFMGYVI 5.88100 6.04991 -0.16891

7 NLQSLTNLL 6.00000 6.69950 -0.69950

8 FVTWHRYHL 6.02500 5.79902 0.22598

9 DPKVKQWPL 6.17600 5.77871 0.39729

10 ITSQVPFSV 6.19600 6.52348 -0.32748

11 ALAKAAAAI 6.21100 6.23533 -0.02433

12 GLGQVPLIV 6.30100 6.57734 -0.27634

13 MLDLQPETT 6.33500 6.83792 -0.50292

14 LLSSNLSWL 6.34200 6.37191 -0.02991

15 GLACHQLCA 6.38000 5.98553 0.39447

16 LIGNESFAL 6.41500 7.04673 -0.63173

17 ALAKAAAAV 6.41900 6.43590 -0.01690

18 LLAVGATKV 6.47700 6.49910 -0.02210

19 ALAKAAAAL 6.51100 6.21844 0.29256

20 WILRGTSFV 6.55600 6.91118 -0.35518

21 IISCTCPTV 6.58000 6.66750 -0.08750

22 FLGGTPVCL 6.62300 6.90809 -0.28509

23 ALIHHNTHL 6.62300 6.79985 -0.17685

24 NLSWLSLDV 6.63900 6.07284 0.56616

25 YMIMVKCWM 6.66300 6.84179 -0.17879

26 VLQAGFFLL 6.68200 7.08167 -0.39967

27 GTLGIVCPI 6.71400 6.45143 0.26257

28 VILGVLLLI 6.78500 7.49754 -0.71254

29 VTWHRYHLL 6.79300 6.55655 0.23645

30 PLLPIFFCL 6.79600 7.56901 -0.77301

31 TLGIVCPIC 6.81500 6.03959 0.77541

32 CLTSTVQLV 6.83200 7.10715 -0.27515

33 ILLLCLIFL 6.84500 6.90506 -0.06006

34 FAFRDLCIV 6.88600 6.61777 0.26823

35 FLEPGPVTA 6.89800 7.41483 -0.51683

36 ALAKAAAAA 6.94700 6.70257 0.24443

37 LMAVVLASL 6.95400 7.56511 -0.61111

38 YVITTQHWL 6.98300 6.35143 0.63157

39 LLCLIFLLV 6.99600 7.58466 -0.58866

40 ITAQVPFSV 7.02000 6.61083 0.40917

41 YLEPGPVTL 7.05800 7.17212 -0.11412

42 YTDQVPFSV 7.06600 7.02634 0.03966

43 NLYVSLLLL 7.11400 7.05150 0.06250

44 ILHNGAYSL 7.12700 7.36469 -0.23769

45 SIISAVVGI 7.15900 7.28146 -0.12246

46 VVMGTLVAL 7.17400 7.28788 -0.11388

47 YLEPGPVTI 7.18700 7.18901 -0.00201

48 GLSRYVARL 7.24800 7.45975 -0.21175

49 LLAQFTSAI 7.30100 7.44403 -0.14303

50 VLLDYQGML 7.32800 7.61040 -0.28240

51 YLEPGPVTV 7.34200 7.38958 -0.04758

52 ILSPFMPLL 7.34700 7.17668 0.17032

53 YLSPGPVTA 7.38300 7.48195 -0.09895

54 IIDQVPFSV 7.39800 7.64172 -0.24372

55 SVYDFFVWL 7.44400 7.38430 0.05970

56 ITWQVPFSV 7.46300 7.40082 0.06218

57 ITYQVPFSV 7.48000 7.62430 -0.14430

58 GLYSSTVPV 7.48100 7.58065 -0.09965

59 VMGTLVALV 7.55300 7.25125 0.30175

60 LLLCLIFLL 7.58500 7.22262 0.36238

61 SLDDYNHLV 7.58500 7.12997 0.45503

62 VLIQRNPQL 7.64400 6.97482 0.66918

63 SLYADSPSV 7.65800 7.66635 -0.00835

64 ILSQVPFSV 7.69900 7.64572 0.05328

65 IMDQVPFSV 7.71900 8.07160 -0.35260

66 QLFEDNYAL 7.76400 7.49881 0.26519

67 ALMDKSLHV 7.77000 7.50227 0.26773

68 YAIDLPVSV 7.79600 7.51347 0.28253

69 FVWLHYYSV 7.82400 8.14670 -0.32270

70 MLGTHTMEV 7.84500 7.29992 0.54508

71 LLFGYPVYV 7.88600 8.06754 -0.18154

72 ILKEPVHGV 7.92100 7.58321 0.33779

73 YLMPGPVTV 7.93200 7.91392 0.01808

74 WLDQVPFSV 7.93900 7.96273 -0.02373

75 KTWGQYWQV 7.95500 7.63748 0.31752

76 ALMPLYACI 8.00000 7.46076 0.53924

77 YLAPGPVTA 8.03200 7.56931 0.46269

78 YLYPGPVTV 8.05100 8.31610 -0.26510

79 LLMGTLGIV 8.09700 7.67857 0.41843

80 YLWPGPVTV 8.12500 8.09262 0.03238

81 FLLTRILTI 8.14900 7.90089 0.24811

82 GLLGWSPQA 8.23700 8.12515 0.11185

83 ILYQVPFSV 8.31000 8.74654 -0.43654

84 GILTVILGV 8.34700 7.79373 0.55327

85 NMVPFFPPV 8.39800 8.08689 0.31111

86 ILDQVPFSV 8.48100 7.68968 0.79132

87 YLFPGPVTA 8.49500 8.29247 0.20253

88 YLDQVPFSV 8.63800 8.14859 0.48941

89 ILFQVPFSV 8.69900 8.45624 0.24276

90 ILWQVPFSV 8.77000 8.52306 0.24694

Correlation Coeffecient and Stand Error

CORL: 0.884983 CORL~2: 0.783195

RES: 0.370137 SEE: 0.039016

Statistical indices of Training calculation:

ITERATION (B): 13

No. Sequence Expt. Act Predicted Act Difference

1 VALVGLFVL 5.14800 5.69998 -0.55198

2 GTLVALVGL 5.34200 5.88172 -0.53972

3 LQTTIHDII 5.50100 5.71757 -0.21657

4 SLHVGTQCA 5.84200 6.05835 -0.21635

5 ALPYWNFAT 5.86900 6.68058 -0.81158

6 SLNFMGYVI 5.88100 6.01059 -0.12959

7 NLQSLTNLL 6.00000 6.68651 -0.68651

8 FVTWHRYHL 6.02500 5.75658 0.26842

9 DPKVKQWPL 6.17600 5.74436 0.43164

10 ITSQVPFSV 6.19600 6.51897 -0.32297

11 ALAKAAAAI 6.21100 6.21340 -0.00240

12 GLGQVPLIV 6.30100 6.56340 -0.26240

13 MLDLQPETT 6.33500 6.83255 -0.49755

14 LLSSNLSWL 6.34200 6.34799 -0.00599

15 GLACHQLCA 6.38000 5.96071 0.41929

16 LIGNESFAL 6.41500 7.04097 -0.62597

17 ALAKAAAAV 6.41900 6.42404 -0.00504

18 LLAVGATKV 6.47700 6.48460 -0.00760

19 ALAKAAAAL 6.51100 6.19565 0.31535

20 WILRGTSFV 6.55600 6.90376 -0.34776

21 IISCTCPTV 6.58000 6.65247 -0.07247

22 FLGGTPVCL 6.62300 6.89624 -0.27324

23 ALIHHNTHL 6.62300 6.78864 -0.16564

24 NLSWLSLDV 6.63900 6.04324 0.59576

25 YMIMVKCWM 6.66300 6.80841 -0.14541

26 VLQAGFFLL 6.68200 7.07058 -0.38858

27 GTLGIVCPI 6.71400 6.43990 0.27410

28 VILGVLLLI 6.78500 7.49793 -0.71293

29 VTWHRYHLL 6.79300 6.54136 0.25164

30 PLLPIFFCL 6.79600 7.56448 -0.76848

31 TLGIVCPIC 6.81500 6.00212 0.81288

32 CLTSTVQLV 6.83200 7.10727 -0.27527

33 ILLLCLIFL 6.84500 6.88239 -0.03739

34 FAFRDLCIV 6.88600 6.61200 0.27400

35 FLEPGPVTA 6.89800 7.42658 -0.52858

36 ALAKAAAAA 6.94700 6.70410 0.24290

37 LMAVVLASL 6.95400 7.56575 -0.61175

38 YVITTQHWL 6.98300 6.32742 0.65558

39 LLCLIFLLV 6.99600 7.58401 -0.58801

40 ITAQVPFSV 7.02000 6.60805 0.41195

41 YLEPGPVTL 7.05800 7.16426 -0.10626

42 YTDQVPFSV 7.06600 7.03165 0.03435

43 NLYVSLLLL 7.11400 7.03628 0.07772

44 ILHNGAYSL 7.12700 7.36452 -0.23752

45 SIISAVVGI 7.15900 7.28368 -0.12468

46 VVMGTLVAL 7.17400 7.29150 -0.11750

47 YLEPGPVTI 7.18700 7.18201 0.00499

48 GLSRYVARL 7.24800 7.46545 -0.21745

49 LLAQFTSAI 7.30100 7.44847 -0.14747

50 VLLDYQGML 7.32800 7.61474 -0.28674

51 YLEPGPVTV 7.34200 7.39265 -0.05065

52 ILSPFMPLL 7.34700 7.16680 0.18020

53 YLSPGPVTA 7.38300 7.49498 -0.11198

54 IIDQVPFSV 7.39800 7.65357 -0.25557

55 SVYDFFVWL 7.44400 7.38345 0.06055

56 ITWQVPFSV 7.46300 7.41359 0.04941

57 ITYQVPFSV 7.48000 7.64147 -0.16147

58 GLYSSTVPV 7.48100 7.59176 -0.11076

59 VMGTLVALV 7.55300 7.25057 0.30243

60 LLLCLIFLL 7.58500 7.20743 0.37757

61 SLDDYNHLV 7.58500 7.13257 0.45243

62 VLIQRNPQL 7.64400 6.96586 0.67814

63 SLYADSPSV 7.65800 7.68127 -0.02327

64 ILSQVPFSV 7.69900 7.65740 0.04160

65 IMDQVPFSV 7.71900 8.08966 -0.37066

66 QLFEDNYAL 7.76400 7.49908 0.26492

67 ALMDKSLHV 7.77000 7.51278 0.25722

68 YAIDLPVSV 7.79600 7.53409 0.26191

69 FVWLHYYSV 7.82400 8.16304 -0.33904

70 MLGTHTMEV 7.84500 7.30338 0.54162

71 LLFGYPVYV 7.88600 8.08252 -0.19652

72 ILKEPVHGV 7.92100 7.59536 0.32564

73 YLMPGPVTV 7.93200 7.92731 0.00469

74 WLDQVPFSV 7.93900 7.98060 -0.04160

75 KTWGQYWQV 7.95500 7.65421 0.30079

76 ALMPLYACI 8.00000 7.45652 0.54348

77 YLAPGPVTA 8.03200 7.58406 0.44794

78 YLYPGPVTV 8.05100 8.33741 -0.28641

79 LLMGTLGIV 8.09700 7.68962 0.40738

80 YLWPGPVTV 8.12500 8.10953 0.01547

81 FLLTRILTI 8.14900 7.90989 0.23911

82 GLLGWSPQA 8.23700 8.15823 0.07877

83 ILYQVPFSV 8.31000 8.77990 -0.46990

84 GILTVILGV 8.34700 7.80903 0.53797

85 NMVPFFPPV 8.39800 8.10182 0.29618

86 ILDQVPFSV 8.48100 7.70223 0.77877

87 YLFPGPVTA 8.49500 8.32146 0.17354

88 YLDQVPFSV 8.63800 8.17008 0.46792

89 ILFQVPFSV 8.69900 8.48388 0.21512

90 ILWQVPFSV 8.77000 8.55202 0.21798

Correlation Coeffecient and Stand Error

CORL: 0.885464 CORL~2: 0.784046

RES: 0.369721 SEE: 0.038972

Statistical indices of Training calculation:

ITERATION (A): 14

No. Sequence Expt. Act Predicted Act Difference

1 VALVGLFVL 5.14800 5.73224 -0.58424

2 GTLVALVGL 5.34200 5.91053 -0.56853

3 LQTTIHDII 5.50100 5.75331 -0.25231

4 SLHVGTQCA 5.84200 6.08725 -0.24525

5 ALPYWNFAT 5.86900 6.69099 -0.82199

6 SLNFMGYVI 5.88100 6.03812 -0.15712

7 NLQSLTNLL 6.00000 6.69947 -0.69947

8 FVTWHRYHL 6.02500 5.78980 0.23520

9 DPKVKQWPL 6.17600 5.77210 0.40390

10 ITSQVPFSV 6.19600 6.53119 -0.33519

11 ALAKAAAAI 6.21100 6.23567 -0.02467

12 GLGQVPLIV 6.30100 6.57480 -0.27380

13 MLDLQPETT 6.33500 6.84156 -0.50656

14 LLSSNLSWL 6.34200 6.36866 -0.02666

15 GLACHQLCA 6.38000 5.99439 0.38561

16 LIGNESFAL 6.41500 7.04712 -0.63212

17 ALAKAAAAV 6.41900 6.44204 -0.02304

18 LLAVGATKV 6.47700 6.50108 -0.02408

19 ALAKAAAAL 6.51100 6.21859 0.29241

20 WILRGTSFV 6.55600 6.91113 -0.35513

21 IISCTCPTV 6.58000 6.66734 -0.08734

22 FLGGTPVCL 6.62300 6.90356 -0.28056

23 ALIHHNTHL 6.62300 6.79867 -0.17567

24 NLSWLSLDV 6.63900 6.07060 0.56840

25 YMIMVKCWM 6.66300 6.81773 -0.15473

26 VLQAGFFLL 6.68200 7.07522 -0.39322

27 GTLGIVCPI 6.71400 6.45944 0.25456

28 VILGVLLLI 6.78500 7.49426 -0.70926

29 VTWHRYHLL 6.79300 6.55630 0.23670

30 PLLPIFFCL 6.79600 7.56160 -0.76560

31 TLGIVCPIC 6.81500 6.02763 0.78737

32 CLTSTVQLV 6.83200 7.10866 -0.27666

33 ILLLCLIFL 6.84500 6.89015 -0.04515

34 FAFRDLCIV 6.88600 6.62206 0.26394

35 FLEPGPVTA 6.89800 7.42559 -0.52759

36 ALAKAAAAA 6.94700 6.71651 0.23049

37 LMAVVLASL 6.95400 7.55747 -0.60347

38 YVITTQHWL 6.98300 6.34929 0.63371

39 LLCLIFLLV 6.99600 7.57538 -0.57938

40 ITAQVPFSV 7.02000 6.61768 0.40232

41 YLEPGPVTL 7.05800 7.16881 -0.11081

42 YTDQVPFSV 7.06600 7.03257 0.03343

43 NLYVSLLLL 7.11400 7.04165 0.07235

44 ILHNGAYSL 7.12700 7.36198 -0.23498

45 SIISAVVGI 7.15900 7.28419 -0.12519

46 VVMGTLVAL 7.17400 7.29145 -0.11745

47 YLEPGPVTI 7.18700 7.18589 0.00111

48 GLSRYVARL 7.24800 7.45866 -0.21066

49 LLAQFTSAI 7.30100 7.44288 -0.14188

50 VLLDYQGML 7.32800 7.60749 -0.27949

51 YLEPGPVTV 7.34200 7.39227 -0.05027

52 ILSPFMPLL 7.34700 7.17164 0.17536

53 YLSPGPVTA 7.38300 7.49271 -0.10971

54 IIDQVPFSV 7.39800 7.64331 -0.24531

55 SVYDFFVWL 7.44400 7.37988 0.06412

56 ITWQVPFSV 7.46300 7.40586 0.05714

57 ITYQVPFSV 7.48000 7.62899 -0.14899

58 GLYSSTVPV 7.48100 7.58711 -0.10611

59 VMGTLVALV 7.55300 7.24996 0.30304

60 LLLCLIFLL 7.58500 7.21166 0.37334

61 SLDDYNHLV 7.58500 7.13575 0.44925

62 VLIQRNPQL 7.64400 6.97124 0.67276

63 SLYADSPSV 7.65800 7.67239 -0.01439

64 ILSQVPFSV 7.69900 7.64608 0.05292

65 IMDQVPFSV 7.71900 8.06795 -0.34895

66 QLFEDNYAL 7.76400 7.49459 0.26941

67 ALMDKSLHV 7.77000 7.50566 0.26434

68 YAIDLPVSV 7.79600 7.52435 0.27165

69 FVWLHYYSV 7.82400 8.14283 -0.31883

70 MLGTHTMEV 7.84500 7.30212 0.54288

71 LLFGYPVYV 7.88600 8.06287 -0.17687

72 ILKEPVHGV 7.92100 7.58542 0.33558

73 YLMPGPVTV 7.93200 7.91459 0.01741

74 WLDQVPFSV 7.93900 7.96194 -0.02294

75 KTWGQYWQV 7.95500 7.64437 0.31063

76 ALMPLYACI 8.00000 7.45734 0.54266

77 YLAPGPVTA 8.03200 7.57920 0.45280

78 YLYPGPVTV 8.05100 8.31605 -0.26505

79 LLMGTLGIV 8.09700 7.68005 0.41695

80 YLWPGPVTV 8.12500 8.09291 0.03209

81 FLLTRILTI 8.14900 7.89755 0.25145

82 GLLGWSPQA 8.23700 8.13792 0.09908

83 ILYQVPFSV 8.31000 8.74389 -0.43389

84 GILTVILGV 8.34700 7.79924 0.54776

85 NMVPFFPPV 8.39800 8.08772 0.31028

86 ILDQVPFSV 8.48100 7.69013 0.79087

87 YLFPGPVTA 8.49500 8.30049 0.19451

88 YLDQVPFSV 8.63800 8.14747 0.49053

89 ILFQVPFSV 8.69900 8.45386 0.24514

90 ILWQVPFSV 8.77000 8.52076 0.24924

Correlation Coeffecient and Stand Error

CORL: 0.885487 CORL~2: 0.784087

RES: 0.369375 SEE: 0.038936

Statistical indices of Training calculation:

ITERATION (B): 14

No. Sequence Expt. Act Predicted Act Difference

1 VALVGLFVL 5.14800 5.70553 -0.55753

2 GTLVALVGL 5.34200 5.88916 -0.54716

3 LQTTIHDII 5.50100 5.72964 -0.22864

4 SLHVGTQCA 5.84200 6.07070 -0.22870

5 ALPYWNFAT 5.86900 6.67624 -0.80724

6 SLNFMGYVI 5.88100 6.00450 -0.12350

7 NLQSLTNLL 6.00000 6.68855 -0.68855

8 FVTWHRYHL 6.02500 5.75357 0.27143

9 DPKVKQWPL 6.17600 5.74334 0.43266

10 ITSQVPFSV 6.19600 6.52795 -0.33195

11 ALAKAAAAI 6.21100 6.21662 -0.00562

12 GLGQVPLIV 6.30100 6.56274 -0.26174

13 MLDLQPETT 6.33500 6.83585 -0.50085

14 LLSSNLSWL 6.34200 6.34825 -0.00625

15 GLACHQLCA 6.38000 5.97232 0.40768

16 LIGNESFAL 6.41500 7.04246 -0.62746

17 ALAKAAAAV 6.41900 6.43130 -0.01230

18 LLAVGATKV 6.47700 6.48791 -0.01091

19 ALAKAAAAL 6.51100 6.19885 0.31215

20 WILRGTSFV 6.55600 6.90419 -0.34819

21 IISCTCPTV 6.58000 6.65392 -0.07392

22 FLGGTPVCL 6.62300 6.89353 -0.27053

23 ALIHHNTHL 6.62300 6.78900 -0.16600

24 NLSWLSLDV 6.63900 6.04450 0.59450

25 YMIMVKCWM 6.66300 6.78882 -0.12582

26 VLQAGFFLL 6.68200 7.06631 -0.38431

27 GTLGIVCPI 6.71400 6.45088 0.26312

28 VILGVLLLI 6.78500 7.49512 -0.71012

29 VTWHRYHLL 6.79300 6.54431 0.24869

30 PLLPIFFCL 6.79600 7.55841 -0.76241

31 TLGIVCPIC 6.81500 5.99550 0.81950

32 CLTSTVQLV 6.83200 7.10847 -0.27647

33 ILLLCLIFL 6.84500 6.87082 -0.02582

34 FAFRDLCIV 6.88600 6.61786 0.26814

35 FLEPGPVTA 6.89800 7.43475 -0.53675

36 ALAKAAAAA 6.94700 6.71682 0.23018

37 LMAVVLASL 6.95400 7.55759 -0.60359

38 YVITTQHWL 6.98300 6.32898 0.65402

39 LLCLIFLLV 6.99600 7.57460 -0.57860

40 ITAQVPFSV 7.02000 6.61591 0.40409

41 YLEPGPVTL 7.05800 7.16191 -0.10391

42 YTDQVPFSV 7.06600 7.03764 0.02836

43 NLYVSLLLL 7.11400 7.02882 0.08518

44 ILHNGAYSL 7.12700 7.36222 -0.23522

45 SIISAVVGI 7.15900 7.28638 -0.12738

46 VVMGTLVAL 7.17400 7.29527 -0.12127

47 YLEPGPVTI 7.18700 7.17967 0.00733

48 GLSRYVARL 7.24800 7.46382 -0.21582

49 LLAQFTSAI 7.30100 7.44656 -0.14556

50 VLLDYQGML 7.32800 7.61153 -0.28353

51 YLEPGPVTV 7.34200 7.39436 -0.05236

52 ILSPFMPLL 7.34700 7.16351 0.18349

53 YLSPGPVTA 7.38300 7.50289 -0.11989

54 IIDQVPFSV 7.39800 7.65342 -0.25542

55 SVYDFFVWL 7.44400 7.38029 0.06371

56 ITWQVPFSV 7.46300 7.41748 0.04552

57 ITYQVPFSV 7.48000 7.64440 -0.16440

58 GLYSSTVPV 7.48100 7.59656 -0.11556

59 VMGTLVALV 7.55300 7.24883 0.30417

60 LLLCLIFLL 7.58500 7.19892 0.38608

61 SLDDYNHLV 7.58500 7.13800 0.44700

62 VLIQRNPQL 7.64400 6.96349 0.68051

63 SLYADSPSV 7.65800 7.68490 -0.02690

64 ILSQVPFSV 7.69900 7.65599 0.04301

65 IMDQVPFSV 7.71900 8.08306 -0.36406

66 QLFEDNYAL 7.76400 7.49527 0.26873

67 ALMDKSLHV 7.77000 7.51455 0.25545

68 YAIDLPVSV 7.79600 7.54268 0.25332

69 FVWLHYYSV 7.82400 8.15726 -0.33326

70 MLGTHTMEV 7.84500 7.30486 0.54014

71 LLFGYPVYV 7.88600 8.07588 -0.18988

72 ILKEPVHGV 7.92100 7.59538 0.32562

73 YLMPGPVTV 7.93200 7.92554 0.00646

74 WLDQVPFSV 7.93900 7.97709 -0.03809

75 KTWGQYWQV 7.95500 7.65992 0.29508

76 ALMPLYACI 8.00000 7.45412 0.54588

77 YLAPGPVTA 8.03200 7.59085 0.44115

78 YLYPGPVTV 8.05100 8.33382 -0.28282

79 LLMGTLGIV 8.09700 7.68926 0.40774

80 YLWPGPVTV 8.12500 8.10690 0.01810

81 FLLTRILTI 8.14900 7.90545 0.24355

82 GLLGWSPQA 8.23700 8.16583 0.07117

83 ILYQVPFSV 8.31000 8.77244 -0.46244

84 GILTVILGV 8.34700 7.81246 0.53454

85 NMVPFFPPV 8.39800 8.10043 0.29757

86 ILDQVPFSV 8.48100 7.70079 0.78021

87 YLFPGPVTA 8.49500 8.32439 0.17061

88 YLDQVPFSV 8.63800 8.16568 0.47232

89 ILFQVPFSV 8.69900 8.47749 0.22151

90 ILWQVPFSV 8.77000 8.54552 0.22448

Correlation Coeffecient and Stand Error

CORL: 0.885835 CORL~2: 0.784704

RES: 0.369072 SEE: 0.038904

Statistical indices of Training calculation:

ITERATION (A): 15

No. Sequence Expt. Act Predicted Act Difference

1 VALVGLFVL 5.14800 5.73356 -0.58556

2 GTLVALVGL 5.34200 5.91331 -0.57131

3 LQTTIHDII 5.50100 5.76013 -0.25913

4 SLHVGTQCA 5.84200 6.09562 -0.25362

5 ALPYWNFAT 5.86900 6.68562 -0.81662

6 SLNFMGYVI 5.88100 6.02846 -0.14746

7 NLQSLTNLL 6.00000 6.69936 -0.69936

8 FVTWHRYHL 6.02500 5.78174 0.24326

9 DPKVKQWPL 6.17600 5.76710 0.40890

10 ITSQVPFSV 6.19600 6.53778 -0.34178

11 ALAKAAAAI 6.21100 6.23600 -0.02500

12 GLGQVPLIV 6.30100 6.57299 -0.27199

13 MLDLQPETT 6.33500 6.84382 -0.50882

14 LLSSNLSWL 6.34200 6.36608 -0.02408

15 GLACHQLCA 6.38000 6.00159 0.37841

16 LIGNESFAL 6.41500 7.04788 -0.63288

17 ALAKAAAAV 6.41900 6.44699 -0.02799

18 LLAVGATKV 6.47700 6.50245 -0.02545

19 ALAKAAAAL 6.51100 6.21880 0.29220

20 WILRGTSFV 6.55600 6.91078 -0.35478

21 IISCTCPTV 6.58000 6.66690 -0.08690

22 FLGGTPVCL 6.62300 6.89971 -0.27671

23 ALIHHNTHL 6.62300 6.79756 -0.17456

24 NLSWLSLDV 6.63900 6.06822 0.57078

25 YMIMVKCWM 6.66300 6.79697 -0.13397

26 VLQAGFFLL 6.68200 7.07040 -0.38840

27 GTLGIVCPI 6.71400 6.46682 0.24718

28 VILGVLLLI 6.78500 7.49168 -0.70668

29 VTWHRYHLL 6.79300 6.55628 0.23672

30 PLLPIFFCL 6.79600 7.55627 -0.76027

31 TLGIVCPIC 6.81500 6.01765 0.79735

32 CLTSTVQLV 6.83200 7.10906 -0.27706

33 ILLLCLIFL 6.84500 6.87764 -0.03264

34 FAFRDLCIV 6.88600 6.62670 0.25930

35 FLEPGPVTA 6.89800 7.43415 -0.53615

36 ALAKAAAAA 6.94700 6.72751 0.21949

37 LMAVVLASL 6.95400 7.55027 -0.59627

38 YVITTQHWL 6.98300 6.34762 0.63538

39 LLCLIFLLV 6.99600 7.56728 -0.57128

40 ITAQVPFSV 7.02000 6.62351 0.39649

41 YLEPGPVTL 7.05800 7.16624 -0.10824

42 YTDQVPFSV 7.06600 7.03770 0.02830

43 NLYVSLLLL 7.11400 7.03366 0.08034

44 ILHNGAYSL 7.12700 7.36023 -0.23323

45 SIISAVVGI 7.15900 7.28650 -0.12750

46 VVMGTLVAL 7.17400 7.29436 -0.12036

47 YLEPGPVTI 7.18700 7.18344 0.00356

48 GLSRYVARL 7.24800 7.45841 -0.21041

49 LLAQFTSAI 7.30100 7.44178 -0.14078

50 VLLDYQGML 7.32800 7.60538 -0.27738

51 YLEPGPVTV 7.34200 7.39443 -0.05243

52 ILSPFMPLL 7.34700 7.16777 0.17923

53 YLSPGPVTA 7.38300 7.50123 -0.11823

54 IIDQVPFSV 7.39800 7.64458 -0.24658

55 SVYDFFVWL 7.44400 7.37702 0.06698

56 ITWQVPFSV 7.46300 7.41017 0.05283

57 ITYQVPFSV 7.48000 7.63294 -0.15294

58 GLYSSTVPV 7.48100 7.59247 -0.11147

59 VMGTLVALV 7.55300 7.24843 0.30457

60 LLLCLIFLL 7.58500 7.20296 0.38204

61 SLDDYNHLV 7.58500 7.14066 0.44434

62 VLIQRNPQL 7.64400 6.96817 0.67583

63 SLYADSPSV 7.65800 7.67720 -0.01920

64 ILSQVPFSV 7.69900 7.64637 0.05263

65 IMDQVPFSV 7.71900 8.06414 -0.34514

66 QLFEDNYAL 7.76400 7.49143 0.27257

67 ALMDKSLHV 7.77000 7.50831 0.26169

68 YAIDLPVSV 7.79600 7.53408 0.26192

69 FVWLHYYSV 7.82400 8.13971 -0.31571

70 MLGTHTMEV 7.84500 7.30403 0.54097

71 LLFGYPVYV 7.88600 8.05890 -0.17290

72 ILKEPVHGV 7.92100 7.58662 0.33438

73 YLMPGPVTV 7.93200 7.91495 0.01705

74 WLDQVPFSV 7.93900 7.96114 -0.02214

75 KTWGQYWQV 7.95500 7.65045 0.30455

76 ALMPLYACI 8.00000 7.45501 0.54499

77 YLAPGPVTA 8.03200 7.58696 0.44504

78 YLYPGPVTV 8.05100 8.31587 -0.26487

79 LLMGTLGIV 8.09700 7.68065 0.41635

80 YLWPGPVTV 8.12500 8.09310 0.03190

81 FLLTRILTI 8.14900 7.89537 0.25363

82 GLLGWSPQA 8.23700 8.14823 0.08877

83 ILYQVPFSV 8.31000 8.74152 -0.43152

84 GILTVILGV 8.34700 7.80424 0.54276

85 NMVPFFPPV 8.39800 8.08807 0.30993

86 ILDQVPFSV 8.48100 7.69046 0.79054

87 YLFPGPVTA 8.49500 8.30665 0.18835

88 YLDQVPFSV 8.63800 8.14628 0.49172

89 ILFQVPFSV 8.69900 8.45178 0.24722

90 ILWQVPFSV 8.77000 8.51876 0.25124

Correlation Coeffecient and Stand Error

CORL: 0.885856 CORL~2: 0.784741

RES: 0.368816 SEE: 0.038877

Statistical indices of Training calculation:

ITERATION (B): 15

No. Sequence Expt. Act Predicted Act Difference

1 VALVGLFVL 5.14800 5.71093 -0.56293

2 GTLVALVGL 5.34200 5.89514 -0.55314

3 LQTTIHDII 5.50100 5.73991 -0.23891

4 SLHVGTQCA 5.84200 6.08097 -0.23897

5 ALPYWNFAT 5.86900 6.67241 -0.80341

6 SLNFMGYVI 5.88100 5.99940 -0.11840

7 NLQSLTNLL 6.00000 6.69002 -0.69002

8 FVTWHRYHL 6.02500 5.75044 0.27456

9 DPKVKQWPL 6.17600 5.74250 0.43350

10 ITSQVPFSV 6.19600 6.53518 -0.33918

11 ALAKAAAAI 6.21100 6.21945 -0.00845

12 GLGQVPLIV 6.30100 6.56250 -0.26150

13 MLDLQPETT 6.33500 6.83843 -0.50343

14 LLSSNLSWL 6.34200 6.34851 -0.00651

15 GLACHQLCA 6.38000 5.98223 0.39777

16 LIGNESFAL 6.41500 7.04398 -0.62898

17 ALAKAAAAV 6.41900 6.43748 -0.01848

18 LLAVGATKV 6.47700 6.49077 -0.01377

19 ALAKAAAAL 6.51100 6.20167 0.30933

20 WILRGTSFV 6.55600 6.90455 -0.34855

21 IISCTCPTV 6.58000 6.65510 -0.07510

22 FLGGTPVCL 6.62300 6.89110 -0.26810

23 ALIHHNTHL 6.62300 6.78918 -0.16618

24 NLSWLSLDV 6.63900 6.04534 0.59366

25 YMIMVKCWM 6.66300 6.77185 -0.10885

26 VLQAGFFLL 6.68200 7.06297 -0.38097

27 GTLGIVCPI 6.71400 6.45996 0.25404

28 VILGVLLLI 6.78500 7.49266 -0.70766

29 VTWHRYHLL 6.79300 6.54635 0.24665

30 PLLPIFFCL 6.79600 7.55384 -0.75784

31 TLGIVCPIC 6.81500 5.98989 0.82511

32 CLTSTVQLV 6.83200 7.10877 -0.27677

33 ILLLCLIFL 6.84500 6.86096 -0.01596

34 FAFRDLCIV 6.88600 6.62335 0.26265

35 FLEPGPVTA 6.89800 7.44172 -0.54372

36 ALAKAAAAA 6.94700 6.72738 0.21962

37 LMAVVLASL 6.95400 7.55019 -0.59619

38 YVITTQHWL 6.98300 6.33018 0.65282

39 LLCLIFLLV 6.99600 7.56650 -0.57050

40 ITAQVPFSV 7.02000 6.62218 0.39782

41 YLEPGPVTL 7.05800 7.16023 -0.10223

42 YTDQVPFSV 7.06600 7.04222 0.02378

43 NLYVSLLLL 7.11400 7.02268 0.09132

44 ILHNGAYSL 7.12700 7.36059 -0.23359

45 SIISAVVGI 7.15900 7.28853 -0.12953

46 VVMGTLVAL 7.17400 7.29796 -0.12396

47 YLEPGPVTI 7.18700 7.17801 0.00899

48 GLSRYVARL 7.24800 7.46295 -0.21495

49 LLAQFTSAI 7.30100 7.44488 -0.14388

50 VLLDYQGML 7.32800 7.60900 -0.28100

51 YLEPGPVTV 7.34200 7.39604 -0.05404

52 ILSPFMPLL 7.34700 7.16091 0.18609

53 YLSPGPVTA 7.38300 7.50966 -0.12666

54 IIDQVPFSV 7.39800 7.65327 -0.25527

55 SVYDFFVWL 7.44400 7.37783 0.06617

56 ITWQVPFSV 7.46300 7.42043 0.04257

57 ITYQVPFSV 7.48000 7.64648 -0.16648

58 GLYSSTVPV 7.48100 7.60065 -0.11965

59 VMGTLVALV 7.55300 7.24725 0.30575

60 LLLCLIFLL 7.58500 7.19208 0.39292

61 SLDDYNHLV 7.58500 7.14259 0.44241

62 VLIQRNPQL 7.64400 6.96144 0.68256

63 SLYADSPSV 7.65800 7.68792 -0.02992

64 ILSQVPFSV 7.69900 7.65486 0.04414

65 IMDQVPFSV 7.71900 8.07703 -0.35803

66 QLFEDNYAL 7.76400 7.49221 0.27179

67 ALMDKSLHV 7.77000 7.51593 0.25407

68 YAIDLPVSV 7.79600 7.55012 0.24588

69 FVWLHYYSV 7.82400 8.15233 -0.32833

70 MLGTHTMEV 7.84500 7.30630 0.53870

71 LLFGYPVYV 7.88600 8.07018 -0.18418

72 ILKEPVHGV 7.92100 7.59502 0.32598

73 YLMPGPVTV 7.93200 7.92424 0.00776

74 WLDQVPFSV 7.93900 7.97413 -0.03513

75 KTWGQYWQV 7.95500 7.66438 0.29062

76 ALMPLYACI 8.00000 7.45247 0.54753

77 YLAPGPVTA 8.03200 7.59665 0.43535

78 YLYPGPVTV 8.05100 8.33106 -0.28006

79 LLMGTLGIV 8.09700 7.68851 0.40849

80 YLWPGPVTV 8.12500 8.10501 0.01999

81 FLLTRILTI 8.14900 7.90232 0.24668

82 GLLGWSPQA 8.23700 8.17215 0.06485

83 ILYQVPFSV 8.31000 8.76616 -0.45616

84 GILTVILGV 8.34700 7.81574 0.53126

85 NMVPFFPPV 8.39800 8.09907 0.29893

86 ILDQVPFSV 8.48100 7.69960 0.78140

87 YLFPGPVTA 8.49500 8.32694 0.16806

88 YLDQVPFSV 8.63800 8.16190 0.47610

89 ILFQVPFSV 8.69900 8.47215 0.22685

90 ILWQVPFSV 8.77000 8.54011 0.22989

Correlation Coeffecient and Stand Error

CORL: 0.886114 CORL~2: 0.785198

RES: 0.368590 SEE: 0.038853

Statistical indices of Training calculation:

ITERATION (A): 16

No. Sequence Expt. Act Predicted Act Difference

1 VALVGLFVL 5.14800 5.73536 -0.58736

2 GTLVALVGL 5.34200 5.91581 -0.57381

3 LQTTIHDII 5.50100 5.76617 -0.26517

4 SLHVGTQCA 5.84200 6.10254 -0.26054

5 ALPYWNFAT 5.86900 6.68080 -0.81180

6 SLNFMGYVI 5.88100 6.02032 -0.13932

7 NLQSLTNLL 6.00000 6.69924 -0.69924

8 FVTWHRYHL 6.02500 5.77480 0.25020

9 DPKVKQWPL 6.17600 5.76317 0.41283

10 ITSQVPFSV 6.19600 6.54346 -0.34746

11 ALAKAAAAI 6.21100 6.23636 -0.02536

12 GLGQVPLIV 6.30100 6.57165 -0.27065

13 MLDLQPETT 6.33500 6.84542 -0.51042

14 LLSSNLSWL 6.34200 6.36399 -0.02199

15 GLACHQLCA 6.38000 6.00768 0.37232

16 LIGNESFAL 6.41500 7.04870 -0.63370

17 ALAKAAAAV 6.41900 6.45114 -0.03214

18 LLAVGATKV 6.47700 6.50350 -0.02650

19 ALAKAAAAL 6.51100 6.21907 0.29193

20 WILRGTSFV 6.55600 6.91040 -0.35440

21 IISCTCPTV 6.58000 6.66645 -0.08645

22 FLGGTPVCL 6.62300 6.89647 -0.27347

23 ALIHHNTHL 6.62300 6.79658 -0.17358

24 NLSWLSLDV 6.63900 6.06599 0.57301

25 YMIMVKCWM 6.66300 6.77914 -0.11614

26 VLQAGFFLL 6.68200 7.06660 -0.38460

27 GTLGIVCPI 6.71400 6.47334 0.24066

28 VILGVLLLI 6.78500 7.48956 -0.70456

29 VTWHRYHLL 6.79300 6.55639 0.23661

30 PLLPIFFCL 6.79600 7.55212 -0.75612

31 TLGIVCPIC 6.81500 6.00929 0.80571

32 CLTSTVQLV 6.83200 7.10903 -0.27703

33 ILLLCLIFL 6.84500 6.86699 -0.02199

34 FAFRDLCIV 6.88600 6.63110 0.25490

35 FLEPGPVTA 6.89800 7.44121 -0.54321

36 ALAKAAAAA 6.94700 6.73659 0.21041

37 LMAVVLASL 6.95400 7.54379 -0.58979

38 YVITTQHWL 6.98300 6.34631 0.63669

39 LLCLIFLLV 6.99600 7.56024 -0.56424

40 ITAQVPFSV 7.02000 6.62853 0.39147

41 YLEPGPVTL 7.05800 7.16413 -0.10613

42 YTDQVPFSV 7.06600 7.04200 0.02400

43 NLYVSLLLL 7.11400 7.02699 0.08701

44 ILHNGAYSL 7.12700 7.35894 -0.23194

45 SIISAVVGI 7.15900 7.28848 -0.12948

46 VVMGTLVAL 7.17400 7.29680 -0.12280

47 YLEPGPVTI 7.18700 7.18142 0.00558

48 GLSRYVARL 7.24800 7.45849 -0.21049

49 LLAQFTSAI 7.30100 7.44075 -0.13975

50 VLLDYQGML 7.32800 7.60373 -0.27573

51 YLEPGPVTV 7.34200 7.39620 -0.05420

52 ILSPFMPLL 7.34700 7.16463 0.18237

53 YLSPGPVTA 7.38300 7.50826 -0.12526

54 IIDQVPFSV 7.39800 7.64562 -0.24762

55 SVYDFFVWL 7.44400 7.37500 0.06900

56 ITWQVPFSV 7.46300 7.41386 0.04914

57 ITYQVPFSV 7.48000 7.63628 -0.15628

58 GLYSSTVPV 7.48100 7.59697 -0.11597

59 VMGTLVALV 7.55300 7.24697 0.30603

60 LLLCLIFLL 7.58500 7.19578 0.38922

61 SLDDYNHLV 7.58500 7.14483 0.44017

62 VLIQRNPQL 7.64400 6.96556 0.67844

63 SLYADSPSV 7.65800 7.68116 -0.02316

64 ILSQVPFSV 7.69900 7.64659 0.05241

65 IMDQVPFSV 7.71900 8.06057 -0.34157

66 QLFEDNYAL 7.76400 7.48890 0.27510

67 ALMDKSLHV 7.77000 7.51043 0.25957

68 YAIDLPVSV 7.79600 7.54256 0.25344

69 FVWLHYYSV 7.82400 8.13709 -0.31309

70 MLGTHTMEV 7.84500 7.30567 0.53933

71 LLFGYPVYV 7.88600 8.05544 -0.16944

72 ILKEPVHGV 7.92100 7.58736 0.33364

73 YLMPGPVTV 7.93200 7.91515 0.01685

74 WLDQVPFSV 7.93900 7.96037 -0.02137

75 KTWGQYWQV 7.95500 7.65570 0.29930

76 ALMPLYACI 8.00000 7.45324 0.54676

77 YLAPGPVTA 8.03200 7.59332 0.43868

78 YLYPGPVTV 8.05100 8.31563 -0.26463

79 LLMGTLGIV 8.09700 7.68083 0.41617

80 YLWPGPVTV 8.12500 8.09320 0.03180

81 FLLTRILTI 8.14900 7.89375 0.25525

82 GLLGWSPQA 8.23700 8.15676 0.08024

83 ILYQVPFSV 8.31000 8.73941 -0.42941

84 GILTVILGV 8.34700 7.80863 0.53837

85 NMVPFFPPV 8.39800 8.08817 0.30983

86 ILDQVPFSV 8.48100 7.69068 0.79032

87 YLFPGPVTA 8.49500 8.31162 0.18338

88 YLDQVPFSV 8.63800 8.14512 0.49288

89 ILFQVPFSV 8.69900 8.44995 0.24905

90 ILWQVPFSV 8.77000 8.51698 0.25302

Correlation Coeffecient and Stand Error

CORL: 0.886132 CORL~2: 0.785230

RES: 0.368396 SEE: 0.038832

Statistical indices of Training calculation:

ITERATION (B): 16

No. Sequence Expt. Act Predicted Act Difference

1 VALVGLFVL 5.14800 5.71582 -0.56782

2 GTLVALVGL 5.34200 5.90010 -0.55810

3 LQTTIHDII 5.50100 5.74865 -0.24765

4 SLHVGTQCA 5.84200 6.08966 -0.24766

5 ALPYWNFAT 5.86900 6.66907 -0.80007

6 SLNFMGYVI 5.88100 5.99500 -0.11400

7 NLQSLTNLL 6.00000 6.69116 -0.69116

8 FVTWHRYHL 6.02500 5.74752 0.27748

9 DPKVKQWPL 6.17600 5.74185 0.43415

10 ITSQVPFSV 6.19600 6.54122 -0.34522

11 ALAKAAAAI 6.21100 6.22193 -0.01093

12 GLGQVPLIV 6.30100 6.56248 -0.26148

13 MLDLQPETT 6.33500 6.84053 -0.50553

14 LLSSNLSWL 6.34200 6.34874 -0.00674

15 GLACHQLCA 6.38000 5.99074 0.38926

16 LIGNESFAL 6.41500 7.04537 -0.63037

17 ALAKAAAAV 6.41900 6.44279 -0.02379

18 LLAVGATKV 6.47700 6.49322 -0.01622

19 ALAKAAAAL 6.51100 6.20415 0.30685

20 WILRGTSFV 6.55600 6.90489 -0.34889

21 IISCTCPTV 6.58000 6.65611 -0.07611

22 FLGGTPVCL 6.62300 6.88901 -0.26601

23 ALIHHNTHL 6.62300 6.78930 -0.16630

24 NLSWLSLDV 6.63900 6.04592 0.59308

25 YMIMVKCWM 6.66300 6.75722 -0.09422

26 VLQAGFFLL 6.68200 7.06028 -0.37828

27 GTLGIVCPI 6.71400 6.46757 0.24643

28 VILGVLLLI 6.78500 7.49052 -0.70552

29 VTWHRYHLL 6.79300 6.54790 0.24510

30 PLLPIFFCL 6.79600 7.55017 -0.75417

31 TLGIVCPIC 6.81500 5.98513 0.82987

32 CLTSTVQLV 6.83200 7.10872 -0.27672

33 ILLLCLIFL 6.84500 6.85246 -0.00746

34 FAFRDLCIV 6.88600 6.62825 0.25775

35 FLEPGPVTA 6.89800 7.44769 -0.54969

36 ALAKAAAAA 6.94700 6.73633 0.21067

37 LMAVVLASL 6.95400 7.54366 -0.58966

38 YVITTQHWL 6.98300 6.33119 0.65181

39 LLCLIFLLV 6.99600 7.55949 -0.56349

40 ITAQVPFSV 7.02000 6.62738 0.39262

41 YLEPGPVTL 7.05800 7.15892 -0.10092

42 YTDQVPFSV 7.06600 7.04592 0.02008

43 NLYVSLLLL 7.11400 7.01751 0.09649

44 ILHNGAYSL 7.12700 7.35933 -0.23233

45 SIISAVVGI 7.15900 7.29033 -0.13133

46 VVMGTLVAL 7.17400 7.30005 -0.12605

47 YLEPGPVTI 7.18700 7.17670 0.01030

48 GLSRYVARL 7.24800 7.46246 -0.21446

49 LLAQFTSAI 7.30100 7.44340 -0.14240

50 VLLDYQGML 7.32800 7.60692 -0.27892

51 YLEPGPVTV 7.34200 7.39755 -0.05555

52 ILSPFMPLL 7.34700 7.15874 0.18826

53 YLSPGPVTA 7.38300 7.51546 -0.13246

54 IIDQVPFSV 7.39800 7.65314 -0.25514

55 SVYDFFVWL 7.44400 7.37589 0.06811

56 ITWQVPFSV 7.46300 7.42281 0.04019

57 ITYQVPFSV 7.48000 7.64810 -0.16810

58 GLYSSTVPV 7.48100 7.60414 -0.12314

59 VMGTLVALV 7.55300 7.24587 0.30713

60 LLLCLIFLL 7.58500 7.18637 0.39863

61 SLDDYNHLV 7.58500 7.14650 0.43850

62 VLIQRNPQL 7.64400 6.95970 0.68430

63 SLYADSPSV 7.65800 7.69048 -0.03248

64 ILSQVPFSV 7.69900 7.65394 0.04506

65 IMDQVPFSV 7.71900 8.07170 -0.35270

66 QLFEDNYAL 7.76400 7.48968 0.27432

67 ALMDKSLHV 7.77000 7.51704 0.25296

68 YAIDLPVSV 7.79600 7.55654 0.23946

69 FVWLHYYSV 7.82400 8.14811 -0.32411

70 MLGTHTMEV 7.84500 7.30762 0.53738

71 LLFGYPVYV 7.88600 8.06526 -0.17926

72 ILKEPVHGV 7.92100 7.59458 0.32642

73 YLMPGPVTV 7.93200 7.92318 0.00882

74 WLDQVPFSV 7.93900 7.97161 -0.03261

75 KTWGQYWQV 7.95500 7.66800 0.28700

76 ALMPLYACI 8.00000 7.45118 0.54882

77 YLAPGPVTA 8.03200 7.60162 0.43038

78 YLYPGPVTV 8.05100 8.32881 -0.27781

79 LLMGTLGIV 8.09700 7.68764 0.40936

80 YLWPGPVTV 8.12500 8.10352 0.02148

81 FLLTRILTI 8.14900 7.89987 0.24913

82 GLLGWSPQA 8.23700 8.17748 0.05952

83 ILYQVPFSV 8.31000 8.76082 -0.45082

84 GILTVILGV 8.34700 7.81869 0.52831

85 NMVPFFPPV 8.39800 8.09779 0.30021

86 ILDQVPFSV 8.48100 7.69860 0.78240

87 YLFPGPVTA 8.49500 8.32916 0.16584

88 YLDQVPFSV 8.63800 8.15864 0.47936

89 ILFQVPFSV 8.69900 8.46763 0.23137

90 ILWQVPFSV 8.77000 8.53553 0.23447

Correlation Coeffecient and Stand Error

CORL: 0.886326 CORL~2: 0.785573

RES: 0.368225 SEE: 0.038814

Statistical indices of Training calculation:

ITERATION (A): 17

No. Sequence Expt. Act Predicted Act Difference

1 VALVGLFVL 5.14800 5.73720 -0.58920

2 GTLVALVGL 5.34200 5.91804 -0.57604

3 LQTTIHDII 5.50100 5.77145 -0.27045

4 SLHVGTQCA 5.84200 6.10841 -0.26641

5 ALPYWNFAT 5.86900 6.67655 -0.80755

6 SLNFMGYVI 5.88100 6.01335 -0.13235

7 NLQSLTNLL 6.00000 6.69914 -0.69914

8 FVTWHRYHL 6.02500 5.76883 0.25617

9 DPKVKQWPL 6.17600 5.76002 0.41598

10 ITSQVPFSV 6.19600 6.54838 -0.35238

11 ALAKAAAAI 6.21100 6.23674 -0.02574

12 GLGQVPLIV 6.30100 6.57061 -0.26961

13 MLDLQPETT 6.33500 6.84666 -0.51166

14 LLSSNLSWL 6.34200 6.36226 -0.02026

15 GLACHQLCA 6.38000 6.01294 0.36706

16 LIGNESFAL 6.41500 7.04948 -0.63448

17 ALAKAAAAV 6.41900 6.45470 -0.03570

18 LLAVGATKV 6.47700 6.50436 -0.02736

19 ALAKAAAAL 6.51100 6.21939 0.29161

20 WILRGTSFV 6.55600 6.91005 -0.35405

21 IISCTCPTV 6.58000 6.66606 -0.08606

22 FLGGTPVCL 6.62300 6.89375 -0.27075

23 ALIHHNTHL 6.62300 6.79575 -0.17275

24 NLSWLSLDV 6.63900 6.06398 0.57502

25 YMIMVKCWM 6.66300 6.76379 -0.10079

26 VLQAGFFLL 6.68200 7.06351 -0.38151

27 GTLGIVCPI 6.71400 6.47901 0.23499

28 VILGVLLLI 6.78500 7.48776 -0.70276

29 VTWHRYHLL 6.79300 6.55656 0.23644

30 PLLPIFFCL 6.79600 7.54871 -0.75271

31 TLGIVCPIC 6.81500 6.00223 0.81277

32 CLTSTVQLV 6.83200 7.10884 -0.27684

33 ILLLCLIFL 6.84500 6.85780 -0.01280

34 FAFRDLCIV 6.88600 6.63508 0.25092

35 FLEPGPVTA 6.89800 7.44717 -0.54917

36 ALAKAAAAA 6.94700 6.74429 0.20271

37 LMAVVLASL 6.95400 7.53808 -0.58408

38 YVITTQHWL 6.98300 6.34529 0.63771

39 LLCLIFLLV 6.99600 7.55409 -0.55809

40 ITAQVPFSV 7.02000 6.63286 0.38714

41 YLEPGPVTL 7.05800 7.16236 -0.10436

42 YTDQVPFSV 7.06600 7.04565 0.02035

43 NLYVSLLLL 7.11400 7.02135 0.09265

44 ILHNGAYSL 7.12700 7.35791 -0.23091

45 SIISAVVGI 7.15900 7.29020 -0.13120

46 VVMGTLVAL 7.17400 7.29889 -0.12489

47 YLEPGPVTI 7.18700 7.17971 0.00729

48 GLSRYVARL 7.24800 7.45871 -0.21071

49 LLAQFTSAI 7.30100 7.43980 -0.13880

50 VLLDYQGML 7.32800 7.60236 -0.27436

51 YLEPGPVTV 7.34200 7.39767 -0.05567

52 ILSPFMPLL 7.34700 7.16198 0.18502

53 YLSPGPVTA 7.38300 7.51419 -0.13119

54 IIDQVPFSV 7.39800 7.64648 -0.24848

55 SVYDFFVWL 7.44400 7.37349 0.07051

56 ITWQVPFSV 7.46300 7.41702 0.04598

57 ITYQVPFSV 7.48000 7.63914 -0.15914

58 GLYSSTVPV 7.48100 7.60080 -0.11980

59 VMGTLVALV 7.55300 7.24568 0.30732

60 LLLCLIFLL 7.58500 7.18969 0.39531

61 SLDDYNHLV 7.58500 7.14839 0.43661

62 VLIQRNPQL 7.64400 6.96334 0.68066

63 SLYADSPSV 7.65800 7.68449 -0.02649

64 ILSQVPFSV 7.69900 7.64676 0.05224

65 IMDQVPFSV 7.71900 8.05735 -0.33835

66 QLFEDNYAL 7.76400 7.48682 0.27718

67 ALMDKSLHV 7.77000 7.51218 0.25782

68 YAIDLPVSV 7.79600 7.54989 0.24611

69 FVWLHYYSV 7.82400 8.13483 -0.31083

70 MLGTHTMEV 7.84500 7.30709 0.53791

71 LLFGYPVYV 7.88600 8.05239 -0.16639

72 ILKEPVHGV 7.92100 7.58788 0.33312

73 YLMPGPVTV 7.93200 7.91525 0.01675

74 WLDQVPFSV 7.93900 7.95963 -0.02063

75 KTWGQYWQV 7.95500 7.66020 0.29480

76 ALMPLYACI 8.00000 7.45178 0.54822

77 YLAPGPVTA 8.03200 7.59867 0.43333

78 YLYPGPVTV 8.05100 8.31536 -0.26436

79 LLMGTLGIV 8.09700 7.68079 0.41621

80 YLWPGPVTV 8.12500 8.09325 0.03175

81 FLLTRILTI 8.14900 7.89242 0.25658

82 GLLGWSPQA 8.23700 8.16392 0.07308

83 ILYQVPFSV 8.31000 8.73752 -0.42752

84 GILTVILGV 8.34700 7.81243 0.53457

85 NMVPFFPPV 8.39800 8.08815 0.30985

86 ILDQVPFSV 8.48100 7.69084 0.79016

87 YLFPGPVTA 8.49500 8.31576 0.17924

88 YLDQVPFSV 8.63800 8.14403 0.49397

89 ILFQVPFSV 8.69900 8.44832 0.25068

90 ILWQVPFSV 8.77000 8.51540 0.25460

Correlation Coeffecient and Stand Error

CORL: 0.886341 CORL~2: 0.785601

RES: 0.368078 SEE: 0.038799

Statistical indices of Training calculation:

ITERATION (B): 17

No. Sequence Expt. Act Predicted Act Difference

1 VALVGLFVL 5.14800 5.72012 -0.57212

2 GTLVALVGL 5.34200 5.90429 -0.56229

3 LQTTIHDII 5.50100 5.75612 -0.25512

4 SLHVGTQCA 5.84200 6.09713 -0.25513

5 ALPYWNFAT 5.86900 6.66615 -0.79715

6 SLNFMGYVI 5.88100 5.99115 -0.11015

7 NLQSLTNLL 6.00000 6.69209 -0.69209

8 FVTWHRYHL 6.02500 5.74489 0.28011

9 DPKVKQWPL 6.17600 5.74136 0.43464

10 ITSQVPFSV 6.19600 6.54637 -0.35037

11 ALAKAAAAI 6.21100 6.22412 -0.01312

12 GLGQVPLIV 6.30100 6.56256 -0.26156

13 MLDLQPETT 6.33500 6.84230 -0.50730

14 LLSSNLSWL 6.34200 6.34895 -0.00695

15 GLACHQLCA 6.38000 5.99811 0.38189

16 LIGNESFAL 6.41500 7.04659 -0.63159

17 ALAKAAAAV 6.41900 6.44738 -0.02838

18 LLAVGATKV 6.47700 6.49532 -0.01832

19 ALAKAAAAL 6.51100 6.20634 0.30466

20 WILRGTSFV 6.55600 6.90521 -0.34921

21 IISCTCPTV 6.58000 6.65699 -0.07699

22 FLGGTPVCL 6.62300 6.88725 -0.26425

23 ALIHHNTHL 6.62300 6.78939 -0.16639

24 NLSWLSLDV 6.63900 6.04632 0.59268

25 YMIMVKCWM 6.66300 6.74459 -0.08159

26 VLQAGFFLL 6.68200 7.05803 -0.37603

27 GTLGIVCPI 6.71400 6.47402 0.23998

28 VILGVLLLI 6.78500 7.48865 -0.70365

29 VTWHRYHLL 6.79300 6.54917 0.24383

30 PLLPIFFCL 6.79600 7.54710 -0.75110

31 TLGIVCPIC 6.81500 5.98107 0.83393

32 CLTSTVQLV 6.83200 7.10855 -0.27655

33 ILLLCLIFL 6.84500 6.84506 -0.00006

34 FAFRDLCIV 6.88600 6.63257 0.25343

35 FLEPGPVTA 6.89800 7.45282 -0.55482

36 ALAKAAAAA 6.94700 6.74402 0.20298

37 LMAVVLASL 6.95400 7.53793 -0.58393

38 YVITTQHWL 6.98300 6.33207 0.65093

39 LLCLIFLLV 6.99600 7.55339 -0.55739

40 ITAQVPFSV 7.02000 6.63181 0.38819

41 YLEPGPVTL 7.05800 7.15782 -0.09982

42 YTDQVPFSV 7.06600 7.04903 0.01697

43 NLYVSLLLL 7.11400 7.01309 0.10091

44 ILHNGAYSL 7.12700 7.35828 -0.23128

45 SIISAVVGI 7.15900 7.29186 -0.13286

46 VVMGTLVAL 7.17400 7.30178 -0.12778

47 YLEPGPVTI 7.18700 7.17559 0.01141

48 GLSRYVARL 7.24800 7.46217 -0.21417

49 LLAQFTSAI 7.30100 7.44209 -0.14109

50 VLLDYQGML 7.32800 7.60517 -0.27717

51 YLEPGPVTV 7.34200 7.39886 -0.05686

52 ILSPFMPLL 7.34700 7.15686 0.19014

53 YLSPGPVTA 7.38300 7.52047 -0.13747

54 IIDQVPFSV 7.39800 7.65304 -0.25504

55 SVYDFFVWL 7.44400 7.37433 0.06967

56 ITWQVPFSV 7.46300 7.42483 0.03817

57 ITYQVPFSV 7.48000 7.64945 -0.16945

58 GLYSSTVPV 7.48100 7.60713 -0.12613

59 VMGTLVALV 7.55300 7.24470 0.30830

60 LLLCLIFLL 7.58500 7.18149 0.40351

61 SLDDYNHLV 7.58500 7.14984 0.43516

62 VLIQRNPQL 7.64400 6.95822 0.68578

63 SLYADSPSV 7.65800 7.69267 -0.03467

64 ILSQVPFSV 7.69900 7.65316 0.04584

65 IMDQVPFSV 7.71900 8.06706 -0.34806

66 QLFEDNYAL 7.76400 7.48755 0.27645

67 ALMDKSLHV 7.77000 7.51797 0.25203

68 YAIDLPVSV 7.79600 7.56208 0.23392

69 FVWLHYYSV 7.82400 8.14446 -0.32046

70 MLGTHTMEV 7.84500 7.30878 0.53622

71 LLFGYPVYV 7.88600 8.06098 -0.17498

72 ILKEPVHGV 7.92100 7.59415 0.32685

73 YLMPGPVTV 7.93200 7.92228 0.00972

74 WLDQVPFSV 7.93900 7.96943 -0.03043

75 KTWGQYWQV 7.95500 7.67101 0.28399

76 ALMPLYACI 8.00000 7.45006 0.54994

77 YLAPGPVTA 8.03200 7.60591 0.42609

78 YLYPGPVTV 8.05100 8.32691 -0.27591

79 LLMGTLGIV 8.09700 7.68676 0.41024

80 YLWPGPVTV 8.12500 8.10229 0.02271

81 FLLTRILTI 8.14900 7.89782 0.25118

82 GLLGWSPQA 8.23700 8.18203 0.05497

83 ILYQVPFSV 8.31000 8.75624 -0.44624

84 GILTVILGV 8.34700 7.82128 0.52572

85 NMVPFFPPV 8.39800 8.09662 0.30138

86 ILDQVPFSV 8.48100 7.69774 0.78326

87 YLFPGPVTA 8.49500 8.33109 0.16391

88 YLDQVPFSV 8.63800 8.15582 0.48218

89 ILFQVPFSV 8.69900 8.46378 0.23522

90 ILWQVPFSV 8.77000 8.53162 0.23838

Correlation Coeffecient and Stand Error

CORL: 0.886489 CORL~2: 0.785862

RES: 0.367947 SEE: 0.038785

Statistical indices of Training calculation:

ITERATION (A): 18

No. Sequence Expt. Act Predicted Act Difference

1 VALVGLFVL 5.14800 5.73893 -0.59093

2 GTLVALVGL 5.34200 5.92000 -0.57800

3 LQTTIHDII 5.50100 5.77605 -0.27505

4 SLHVGTQCA 5.84200 6.11351 -0.27151

5 ALPYWNFAT 5.86900 6.67282 -0.80382

6 SLNFMGYVI 5.88100 6.00730 -0.12630

7 NLQSLTNLL 6.00000 6.69906 -0.69906

8 FVTWHRYHL 6.02500 5.76370 0.26130

9 DPKVKQWPL 6.17600 5.75745 0.41855

10 ITSQVPFSV 6.19600 6.55265 -0.35665

11 ALAKAAAAI 6.21100 6.23714 -0.02614

12 GLGQVPLIV 6.30100 6.56978 -0.26878

13 MLDLQPETT 6.33500 6.84769 -0.51269

14 LLSSNLSWL 6.34200 6.36081 -0.01881

15 GLACHQLCA 6.38000 6.01755 0.36245

16 LIGNESFAL 6.41500 7.05017 -0.63517

17 ALAKAAAAV 6.41900 6.45782 -0.03882

18 LLAVGATKV 6.47700 6.50509 -0.02809

19 ALAKAAAAL 6.51100 6.21975 0.29125

20 WILRGTSFV 6.55600 6.90975 -0.35375

21 IISCTCPTV 6.58000 6.66575 -0.08575

22 FLGGTPVCL 6.62300 6.89147 -0.26847

23 ALIHHNTHL 6.62300 6.79506 -0.17206

24 NLSWLSLDV 6.63900 6.06219 0.57681

25 YMIMVKCWM 6.66300 6.75054 -0.08754

26 VLQAGFFLL 6.68200 7.06092 -0.37892

27 GTLGIVCPI 6.71400 6.48392 0.23008

28 VILGVLLLI 6.78500 7.48620 -0.70120

29 VTWHRYHLL 6.79300 6.55676 0.23624

30 PLLPIFFCL 6.79600 7.54583 -0.74983

31 TLGIVCPIC 6.81500 5.99622 0.81878

32 CLTSTVQLV 6.83200 7.10860 -0.27660

33 ILLLCLIFL 6.84500 6.84982 -0.00482

34 FAFRDLCIV 6.88600 6.63860 0.24740

35 FLEPGPVTA 6.89800 7.45228 -0.55428

36 ALAKAAAAA 6.94700 6.75095 0.19605

37 LMAVVLASL 6.95400 7.53306 -0.57906

38 YVITTQHWL 6.98300 6.34449 0.63851

39 LLCLIFLLV 6.99600 7.54870 -0.55270

40 ITAQVPFSV 7.02000 6.63662 0.38338

41 YLEPGPVTL 7.05800 7.16084 -0.10284

42 YTDQVPFSV 7.06600 7.04878 0.01722

43 NLYVSLLLL 7.11400 7.01650 0.09750

44 ILHNGAYSL 7.12700 7.35704 -0.23004

45 SIISAVVGI 7.15900 7.29170 -0.13270

46 VVMGTLVAL 7.17400 7.30071 -0.12671

47 YLEPGPVTI 7.18700 7.17823 0.00877

48 GLSRYVARL 7.24800 7.45896 -0.21096

49 LLAQFTSAI 7.30100 7.43894 -0.13794

50 VLLDYQGML 7.32800 7.60118 -0.27318

51 YLEPGPVTV 7.34200 7.39891 -0.05691

52 ILSPFMPLL 7.34700 7.15970 0.18730

53 YLSPGPVTA 7.38300 7.51928 -0.13628

54 IIDQVPFSV 7.39800 7.64722 -0.24922

55 SVYDFFVWL 7.44400 7.37232 0.07168

56 ITWQVPFSV 7.46300 7.41975 0.04325

57 ITYQVPFSV 7.48000 7.64159 -0.16159

58 GLYSSTVPV 7.48100 7.60408 -0.12308

59 VMGTLVALV 7.55300 7.24457 0.30843

60 LLLCLIFLL 7.58500 7.18445 0.40055

61 SLDDYNHLV 7.58500 7.15145 0.43355

62 VLIQRNPQL 7.64400 6.96146 0.68254

63 SLYADSPSV 7.65800 7.68734 -0.02934

64 ILSQVPFSV 7.69900 7.64689 0.05211

65 IMDQVPFSV 7.71900 8.05451 -0.33551

66 QLFEDNYAL 7.76400 7.48506 0.27894

67 ALMDKSLHV 7.77000 7.51365 0.25635

68 YAIDLPVSV 7.79600 7.55623 0.23977

69 FVWLHYYSV 7.82400 8.13286 -0.30886

70 MLGTHTMEV 7.84500 7.30830 0.53670

71 LLFGYPVYV 7.88600 8.04970 -0.16370

72 ILKEPVHGV 7.92100 7.58828 0.33272

73 YLMPGPVTV 7.93200 7.91528 0.01672

74 WLDQVPFSV 7.93900 7.95895 -0.01995

75 KTWGQYWQV 7.95500 7.66405 0.29095

76 ALMPLYACI 8.00000 7.45053 0.54947

77 YLAPGPVTA 8.03200 7.60325 0.42875

78 YLYPGPVTV 8.05100 8.31508 -0.26408

79 LLMGTLGIV 8.09700 7.68066 0.41634

80 YLWPGPVTV 8.12500 8.09325 0.03175

81 FLLTRILTI 8.14900 7.89126 0.25774

82 GLLGWSPQA 8.23700 8.17003 0.06697

83 ILYQVPFSV 8.31000 8.73582 -0.42582

84 GILTVILGV 8.34700 7.81571 0.53129

85 NMVPFFPPV 8.39800 8.08807 0.30993

86 ILDQVPFSV 8.48100 7.69094 0.79006

87 YLFPGPVTA 8.49500 8.31927 0.17573

88 YLDQVPFSV 8.63800 8.14302 0.49498

89 ILFQVPFSV 8.69900 8.44688 0.25212

90 ILWQVPFSV 8.77000 8.51399 0.25601

Correlation Coeffecient and Stand Error

CORL: 0.886502 CORL~2: 0.785887

RES: 0.367833 SEE: 0.038773

Statistical indices of Training calculation:

ITERATION (B): 18

No. Sequence Expt. Act Predicted Act Difference

1 VALVGLFVL 5.14800 5.72387 -0.57587

2 GTLVALVGL 5.34200 5.90787 -0.56587

3 LQTTIHDII 5.50100 5.76255 -0.26155

4 SLHVGTQCA 5.84200 6.10359 -0.26159

5 ALPYWNFAT 5.86900 6.66360 -0.79460

6 SLNFMGYVI 5.88100 5.98773 -0.10673

7 NLQSLTNLL 6.00000 6.69288 -0.69288

8 FVTWHRYHL 6.02500 5.74258 0.28242

9 DPKVKQWPL 6.17600 5.74100 0.43500

10 ITSQVPFSV 6.19600 6.55083 -0.35483

11 ALAKAAAAI 6.21100 6.22604 -0.01504

12 GLGQVPLIV 6.30100 6.56269 -0.26169

13 MLDLQPETT 6.33500 6.84383 -0.50883

14 LLSSNLSWL 6.34200 6.34913 -0.00713

15 GLACHQLCA 6.38000 6.00452 0.37548

16 LIGNESFAL 6.41500 7.04765 -0.63265

17 ALAKAAAAV 6.41900 6.45138 -0.03238

18 LLAVGATKV 6.47700 6.49713 -0.02013

19 ALAKAAAAL 6.51100 6.20829 0.30271

20 WILRGTSFV 6.55600 6.90550 -0.34950

21 IISCTCPTV 6.58000 6.65777 -0.07777

22 FLGGTPVCL 6.62300 6.88576 -0.26276

23 ALIHHNTHL 6.62300 6.78948 -0.16648

24 NLSWLSLDV 6.63900 6.04661 0.59239

25 YMIMVKCWM 6.66300 6.73363 -0.07063

26 VLQAGFFLL 6.68200 7.05614 -0.37414

27 GTLGIVCPI 6.71400 6.47954 0.23446

28 VILGVLLLI 6.78500 7.48701 -0.70201

29 VTWHRYHLL 6.79300 6.55024 0.24276

30 PLLPIFFCL 6.79600 7.54446 -0.74846

31 TLGIVCPIC 6.81500 5.97760 0.83740

32 CLTSTVQLV 6.83200 7.10834 -0.27634

33 ILLLCLIFL 6.84500 6.83859 0.00641

34 FAFRDLCIV 6.88600 6.63636 0.24964

35 FLEPGPVTA 6.89800 7.45726 -0.55926

36 ALAKAAAAA 6.94700 6.75071 0.19629

37 LMAVVLASL 6.95400 7.53293 -0.57893

38 YVITTQHWL 6.98300 6.33286 0.65014

39 LLCLIFLLV 6.99600 7.54805 -0.55205

40 ITAQVPFSV 7.02000 6.63564 0.38436

41 YLEPGPVTL 7.05800 7.15688 -0.09888

42 YTDQVPFSV 7.06600 7.05169 0.01431

43 NLYVSLLLL 7.11400 7.00926 0.10474

44 ILHNGAYSL 7.12700 7.35738 -0.23038

45 SIISAVVGI 7.15900 7.29319 -0.13419

46 VVMGTLVAL 7.17400 7.30326 -0.12926

47 YLEPGPVTI 7.18700 7.17463 0.01237

48 GLSRYVARL 7.24800 7.46200 -0.21400

49 LLAQFTSAI 7.30100 7.44093 -0.13993

50 VLLDYQGML 7.32800 7.60367 -0.27567

51 YLEPGPVTV 7.34200 7.39997 -0.05797

52 ILSPFMPLL 7.34700 7.15521 0.19179

53 YLSPGPVTA 7.38300 7.52482 -0.14182

54 IIDQVPFSV 7.39800 7.65297 -0.25497

55 SVYDFFVWL 7.44400 7.37306 0.07094

56 ITWQVPFSV 7.46300 7.42657 0.03643

57 ITYQVPFSV 7.48000 7.65062 -0.17062

58 GLYSSTVPV 7.48100 7.60970 -0.12870

59 VMGTLVALV 7.55300 7.24371 0.30929

60 LLLCLIFLL 7.58500 7.17725 0.40775

61 SLDDYNHLV 7.58500 7.15273 0.43227

62 VLIQRNPQL 7.64400 6.95695 0.68705

63 SLYADSPSV 7.65800 7.69457 -0.03657

64 ILSQVPFSV 7.69900 7.65250 0.04650

65 IMDQVPFSV 7.71900 8.06303 -0.34403

66 QLFEDNYAL 7.76400 7.48573 0.27827

67 ALMDKSLHV 7.77000 7.51875 0.25125

68 YAIDLPVSV 7.79600 7.56690 0.22910

69 FVWLHYYSV 7.82400 8.14130 -0.31730

70 MLGTHTMEV 7.84500 7.30980 0.53520

71 LLFGYPVYV 7.88600 8.05725 -0.17125

72 ILKEPVHGV 7.92100 7.59378 0.32722

73 YLMPGPVTV 7.93200 7.92150 0.01050

74 WLDQVPFSV 7.93900 7.96754 -0.02854

75 KTWGQYWQV 7.95500 7.67355 0.28145

76 ALMPLYACI 8.00000 7.44907 0.55093

77 YLAPGPVTA 8.03200 7.60963 0.42237

78 YLYPGPVTV 8.05100 8.32528 -0.27428

79 LLMGTLGIV 8.09700 7.68592 0.41108

80 YLWPGPVTV 8.12500 8.10123 0.02377

81 FLLTRILTI 8.14900 7.89605 0.25295

82 GLLGWSPQA 8.23700 8.18595 0.05105

83 ILYQVPFSV 8.31000 8.75229 -0.44229

84 GILTVILGV 8.34700 7.82352 0.52348

85 NMVPFFPPV 8.39800 8.09556 0.30244

86 ILDQVPFSV 8.48100 7.69699 0.78401

87 YLFPGPVTA 8.49500 8.33278 0.16222

88 YLDQVPFSV 8.63800 8.15336 0.48464

89 ILFQVPFSV 8.69900 8.46046 0.23854

90 ILWQVPFSV 8.77000 8.52824 0.24176

Correlation Coeffecient and Stand Error

CORL: 0.886616 CORL~2: 0.786087

RES: 0.367731 SEE: 0.038762

Statistical indices of Training calculation:

ITERATION (A): 19

No. Sequence Expt. Act Predicted Act Difference

1 VALVGLFVL 5.14800 5.74048 -0.59248

2 GTLVALVGL 5.34200 5.92172 -0.57972

3 LQTTIHDII 5.50100 5.78006 -0.27906

4 SLHVGTQCA 5.84200 6.11797 -0.27597

5 ALPYWNFAT 5.86900 6.66956 -0.80056

6 SLNFMGYVI 5.88100 6.00202 -0.12102

7 NLQSLTNLL 6.00000 6.69901 -0.69901

8 FVTWHRYHL 6.02500 5.75926 0.26574

9 DPKVKQWPL 6.17600 5.75531 0.42069

10 ITSQVPFSV 6.19600 6.55639 -0.36039

11 ALAKAAAAI 6.21100 6.23754 -0.02654

12 GLGQVPLIV 6.30100 6.56912 -0.26812

13 MLDLQPETT 6.33500 6.84858 -0.51358

14 LLSSNLSWL 6.34200 6.35959 -0.01759

15 GLACHQLCA 6.38000 6.02162 0.35838

16 LIGNESFAL 6.41500 7.05078 -0.63578

17 ALAKAAAAV 6.41900 6.46056 -0.04156

18 LLAVGATKV 6.47700 6.50573 -0.02873

19 ALAKAAAAL 6.51100 6.22013 0.29087

20 WILRGTSFV 6.55600 6.90952 -0.35352

21 IISCTCPTV 6.58000 6.66552 -0.08552

22 FLGGTPVCL 6.62300 6.88954 -0.26654

23 ALIHHNTHL 6.62300 6.79449 -0.17149

24 NLSWLSLDV 6.63900 6.06061 0.57839

25 YMIMVKCWM 6.66300 6.73903 -0.07603

26 VLQAGFFLL 6.68200 7.05872 -0.37672

27 GTLGIVCPI 6.71400 6.48819 0.22581

28 VILGVLLLI 6.78500 7.48484 -0.69984

29 VTWHRYHLL 6.79300 6.55697 0.23603

30 PLLPIFFCL 6.79600 7.54334 -0.74734

31 TLGIVCPIC 6.81500 5.99107 0.82393

32 CLTSTVQLV 6.83200 7.10837 -0.27637

33 ILLLCLIFL 6.84500 6.84283 0.00217

34 FAFRDLCIV 6.88600 6.64171 0.24429

35 FLEPGPVTA 6.89800 7.45669 -0.55869

36 ALAKAAAAA 6.94700 6.75675 0.19025

37 LMAVVLASL 6.95400 7.52867 -0.57467

38 YVITTQHWL 6.98300 6.34386 0.63914

39 LLCLIFLLV 6.99600 7.54396 -0.54796

40 ITAQVPFSV 7.02000 6.63992 0.38008

41 YLEPGPVTL 7.05800 7.15953 -0.10153

42 YTDQVPFSV 7.06600 7.05149 0.01451

43 NLYVSLLLL 7.11400 7.01230 0.10170

44 ILHNGAYSL 7.12700 7.35628 -0.22928

45 SIISAVVGI 7.15900 7.29302 -0.13402

46 VVMGTLVAL 7.17400 7.30230 -0.12830

47 YLEPGPVTI 7.18700 7.17693 0.01007

48 GLSRYVARL 7.24800 7.45921 -0.21121

49 LLAQFTSAI 7.30100 7.43815 -0.13715

50 VLLDYQGML 7.32800 7.60017 -0.27217

51 YLEPGPVTV 7.34200 7.39996 -0.05796

52 ILSPFMPLL 7.34700 7.15770 0.18930

53 YLSPGPVTA 7.38300 7.52369 -0.14069

54 IIDQVPFSV 7.39800 7.64784 -0.24984

55 SVYDFFVWL 7.44400 7.37137 0.07263

56 ITWQVPFSV 7.46300 7.42211 0.04089

57 ITYQVPFSV 7.48000 7.64370 -0.16370

58 GLYSSTVPV 7.48100 7.60691 -0.12591

59 VMGTLVALV 7.55300 7.24362 0.30938

60 LLLCLIFLL 7.58500 7.17990 0.40510

61 SLDDYNHLV 7.58500 7.15411 0.43089

62 VLIQRNPQL 7.64400 6.95985 0.68415

63 SLYADSPSV 7.65800 7.68981 -0.03181

64 ILSQVPFSV 7.69900 7.64699 0.05201

65 IMDQVPFSV 7.71900 8.05201 -0.33301

66 QLFEDNYAL 7.76400 7.48355 0.28045

67 ALMDKSLHV 7.77000 7.51491 0.25509

68 YAIDLPVSV 7.79600 7.56173 0.23427

69 FVWLHYYSV 7.82400 8.13111 -0.30711

70 MLGTHTMEV 7.84500 7.30936 0.53564

71 LLFGYPVYV 7.88600 8.04731 -0.16131

72 ILKEPVHGV 7.92100 7.58862 0.33238

73 YLMPGPVTV 7.93200 7.91528 0.01672

74 WLDQVPFSV 7.93900 7.95831 -0.01931

75 KTWGQYWQV 7.95500 7.66736 0.28764

76 ALMPLYACI 8.00000 7.44941 0.55059

77 YLAPGPVTA 8.03200 7.60722 0.42478

78 YLYPGPVTV 8.05100 8.31481 -0.26381

79 LLMGTLGIV 8.09700 7.68047 0.41653

80 YLWPGPVTV 8.12500 8.09321 0.03179

81 FLLTRILTI 8.14900 7.89023 0.25877

82 GLLGWSPQA 8.23700 8.17529 0.06171

83 ILYQVPFSV 8.31000 8.73431 -0.42431

84 GILTVILGV 8.34700 7.81855 0.52845

85 NMVPFFPPV 8.39800 8.08795 0.31005

86 ILDQVPFSV 8.48100 7.69099 0.79001

87 YLFPGPVTA 8.49500 8.32229 0.17271

88 YLDQVPFSV 8.63800 8.14210 0.49590

89 ILFQVPFSV 8.69900 8.44559 0.25341

90 ILWQVPFSV 8.77000 8.51271 0.25729

Correlation Coeffecient and Stand Error

CORL: 0.886628 CORL~2: 0.786108

RES: 0.367642 SEE: 0.038753

Statistical indices of Training calculation:

ITERATION (B): 19

No. Sequence Expt. Act Predicted Act Difference

1 VALVGLFVL 5.14800 5.72714 -0.57914

2 GTLVALVGL 5.34200 5.91097 -0.56897

3 LQTTIHDII 5.50100 5.76810 -0.26710

4 SLHVGTQCA 5.84200 6.10923 -0.26723

5 ALPYWNFAT 5.86900 6.66136 -0.79236

6 SLNFMGYVI 5.88100 5.98470 -0.10370

7 NLQSLTNLL 6.00000 6.69355 -0.69355

8 FVTWHRYHL 6.02500 5.74054 0.28446

9 DPKVKQWPL 6.17600 5.74075 0.43525

10 ITSQVPFSV 6.19600 6.55472 -0.35872

11 ALAKAAAAI 6.21100 6.22774 -0.01674

12 GLGQVPLIV 6.30100 6.56284 -0.26184

13 MLDLQPETT 6.33500 6.84516 -0.51016

14 LLSSNLSWL 6.34200 6.34928 -0.00728

15 GLACHQLCA 6.38000 6.01014 0.36986

16 LIGNESFAL 6.41500 7.04857 -0.63357

17 ALAKAAAAV 6.41900 6.45489 -0.03589

18 LLAVGATKV 6.47700 6.49869 -0.02169

19 ALAKAAAAL 6.51100 6.21001 0.30099

20 WILRGTSFV 6.55600 6.90577 -0.34977

21 IISCTCPTV 6.58000 6.65846 -0.07846

22 FLGGTPVCL 6.62300 6.88451 -0.26151

23 ALIHHNTHL 6.62300 6.78956 -0.16656

24 NLSWLSLDV 6.63900 6.04681 0.59219

25 YMIMVKCWM 6.66300 6.72409 -0.06109

26 VLQAGFFLL 6.68200 7.05451 -0.37251

27 GTLGIVCPI 6.71400 6.48430 0.22970

28 VILGVLLLI 6.78500 7.48557 -0.70057

29 VTWHRYHLL 6.79300 6.55119 0.24181

30 PLLPIFFCL 6.79600 7.54216 -0.74616

31 TLGIVCPIC 6.81500 5.97459 0.84041

32 CLTSTVQLV 6.83200 7.10814 -0.27614

33 ILLLCLIFL 6.84500 6.83289 0.01211

34 FAFRDLCIV 6.88600 6.63969 0.24631

35 FLEPGPVTA 6.89800 7.46113 -0.56313

36 ALAKAAAAA 6.94700 6.75656 0.19044

37 LMAVVLASL 6.95400 7.52854 -0.57454

38 YVITTQHWL 6.98300 6.33358 0.64942

39 LLCLIFLLV 6.99600 7.54336 -0.54736

40 ITAQVPFSV 7.02000 6.63899 0.38101

41 YLEPGPVTL 7.05800 7.15605 -0.09805

42 YTDQVPFSV 7.06600 7.05400 0.01200

43 NLYVSLLLL 7.11400 7.00592 0.10808

44 ILHNGAYSL 7.12700 7.35659 -0.22959

45 SIISAVVGI 7.15900 7.29436 -0.13536

46 VVMGTLVAL 7.17400 7.30455 -0.13055

47 YLEPGPVTI 7.18700 7.17377 0.01323

48 GLSRYVARL 7.24800 7.46188 -0.21388

49 LLAQFTSAI 7.30100 7.43990 -0.13890

50 VLLDYQGML 7.32800 7.60237 -0.27437

51 YLEPGPVTV 7.34200 7.40092 -0.05892

52 ILSPFMPLL 7.34700 7.15374 0.19326

53 YLSPGPVTA 7.38300 7.52861 -0.14561

54 IIDQVPFSV 7.39800 7.65292 -0.25492

55 SVYDFFVWL 7.44400 7.37202 0.07198

56 ITWQVPFSV 7.46300 7.42809 0.03491

57 ITYQVPFSV 7.48000 7.65164 -0.17164

58 GLYSSTVPV 7.48100 7.61193 -0.13093

59 VMGTLVALV 7.55300 7.24287 0.31013

60 LLLCLIFLL 7.58500 7.17353 0.41147

61 SLDDYNHLV 7.58500 7.15523 0.42977

62 VLIQRNPQL 7.64400 6.95587 0.68813

63 SLYADSPSV 7.65800 7.69622 -0.03822

64 ILSQVPFSV 7.69900 7.65194 0.04706

65 IMDQVPFSV 7.71900 8.05953 -0.34053

66 QLFEDNYAL 7.76400 7.48416 0.27984

67 ALMDKSLHV 7.77000 7.51942 0.25058

68 YAIDLPVSV 7.79600 7.57111 0.22489

69 FVWLHYYSV 7.82400 8.13855 -0.31455

70 MLGTHTMEV 7.84500 7.31068 0.53432

71 LLFGYPVYV 7.88600 8.05397 -0.16797

72 ILKEPVHGV 7.92100 7.59347 0.32753

73 YLMPGPVTV 7.93200 7.92079 0.01121

74 WLDQVPFSV 7.93900 7.96589 -0.02689

75 KTWGQYWQV 7.95500 7.67573 0.27927

76 ALMPLYACI 8.00000 7.44816 0.55184

77 YLAPGPVTA 8.03200 7.61288 0.41912

78 YLYPGPVTV 8.05100 8.32385 -0.27285

79 LLMGTLGIV 8.09700 7.68513 0.41187

80 YLWPGPVTV 8.12500 8.10030 0.02470

81 FLLTRILTI 8.14900 7.89449 0.25451

82 GLLGWSPQA 8.23700 8.18936 0.04764

83 ILYQVPFSV 8.31000 8.74885 -0.43885

84 GILTVILGV 8.34700 7.82547 0.52153

85 NMVPFFPPV 8.39800 8.09461 0.30339

86 ILDQVPFSV 8.48100 7.69633 0.78467

87 YLFPGPVTA 8.49500 8.33426 0.16074

88 YLDQVPFSV 8.63800 8.15122 0.48678

89 ILFQVPFSV 8.69900 8.45759 0.24141

90 ILWQVPFSV 8.77000 8.52530 0.24470

Correlation Coeffecient and Stand Error

CORL: 0.886715 CORL~2: 0.786264

RES: 0.367563 SEE: 0.038744

Statistical indices of Training calculation:

ITERATION (A): 20

No. Sequence Expt. Act Predicted Act Difference

1 VALVGLFVL 5.14800 5.74187 -0.59387

2 GTLVALVGL 5.34200 5.92324 -0.58124

3 LQTTIHDII 5.50100 5.78357 -0.28257

4 SLHVGTQCA 5.84200 6.12191 -0.27991

5 ALPYWNFAT 5.86900 6.66669 -0.79769

6 SLNFMGYVI 5.88100 5.99738 -0.11638

7 NLQSLTNLL 6.00000 6.69898 -0.69898

8 FVTWHRYHL 6.02500 5.75540 0.26960

9 DPKVKQWPL 6.17600 5.75352 0.42248

10 ITSQVPFSV 6.19600 6.55967 -0.36367

11 ALAKAAAAI 6.21100 6.23793 -0.02693

12 GLGQVPLIV 6.30100 6.56858 -0.26758

13 MLDLQPETT 6.33500 6.84936 -0.51436

14 LLSSNLSWL 6.34200 6.35854 -0.01654

15 GLACHQLCA 6.38000 6.02523 0.35477

16 LIGNESFAL 6.41500 7.05132 -0.63632

17 ALAKAAAAV 6.41900 6.46299 -0.04399

18 LLAVGATKV 6.47700 6.50629 -0.02929

19 ALAKAAAAL 6.51100 6.22052 0.29048

20 WILRGTSFV 6.55600 6.90932 -0.35332

21 IISCTCPTV 6.58000 6.66533 -0.08533

22 FLGGTPVCL 6.62300 6.88790 -0.26490

23 ALIHHNTHL 6.62300 6.79401 -0.17101

24 NLSWLSLDV 6.63900 6.05921 0.57979

25 YMIMVKCWM 6.66300 6.72899 -0.06599

26 VLQAGFFLL 6.68200 7.05682 -0.37482

27 GTLGIVCPI 6.71400 6.49190 0.22210

28 VILGVLLLI 6.78500 7.48365 -0.69865

29 VTWHRYHLL 6.79300 6.55717 0.23583

30 PLLPIFFCL 6.79600 7.54115 -0.74515

31 TLGIVCPIC 6.81500 5.98661 0.82839

32 CLTSTVQLV 6.83200 7.10815 -0.27615

33 ILLLCLIFL 6.84500 6.83668 0.00832

34 FAFRDLCIV 6.88600 6.64444 0.24156

35 FLEPGPVTA 6.89800 7.46055 -0.56255

36 ALAKAAAAA 6.94700 6.76187 0.18513

37 LMAVVLASL 6.95400 7.52480 -0.57080

38 YVITTQHWL 6.98300 6.34336 0.63964

39 LLCLIFLLV 6.99600 7.53977 -0.54377

40 ITAQVPFSV 7.02000 6.64281 0.37719

41 YLEPGPVTL 7.05800 7.15838 -0.10038

42 YTDQVPFSV 7.06600 7.05385 0.01215

43 NLYVSLLLL 7.11400 7.00864 0.10536

44 ILHNGAYSL 7.12700 7.35561 -0.22861

45 SIISAVVGI 7.15900 7.29420 -0.13520

46 VVMGTLVAL 7.17400 7.30370 -0.12970

47 YLEPGPVTI 7.18700 7.17579 0.01121

48 GLSRYVARL 7.24800 7.45945 -0.21145

49 LLAQFTSAI 7.30100 7.43744 -0.13644

50 VLLDYQGML 7.32800 7.59927 -0.27127

51 YLEPGPVTV 7.34200 7.40085 -0.05885

52 ILSPFMPLL 7.34700 7.15595 0.19105

53 YLSPGPVTA 7.38300 7.52754 -0.14454

54 IIDQVPFSV 7.39800 7.64839 -0.25039

55 SVYDFFVWL 7.44400 7.37060 0.07340

56 ITWQVPFSV 7.46300 7.42416 0.03884

57 ITYQVPFSV 7.48000 7.64555 -0.16555

58 GLYSSTVPV 7.48100 7.60938 -0.12838

59 VMGTLVALV 7.55300 7.24281 0.31019

60 LLLCLIFLL 7.58500 7.17590 0.40910

61 SLDDYNHLV 7.58500 7.15643 0.42857

62 VLIQRNPQL 7.64400 6.95846 0.68554

63 SLYADSPSV 7.65800 7.69196 -0.03396

64 ILSQVPFSV 7.69900 7.64706 0.05194

65 IMDQVPFSV 7.71900 8.04980 -0.33080

66 QLFEDNYAL 7.76400 7.48225 0.28175

67 ALMDKSLHV 7.77000 7.51599 0.25401

68 YAIDLPVSV 7.79600 7.56652 0.22948

69 FVWLHYYSV 7.82400 8.12956 -0.30556

70 MLGTHTMEV 7.84500 7.31027 0.53473

71 LLFGYPVYV 7.88600 8.04518 -0.15918

72 ILKEPVHGV 7.92100 7.58891 0.33209

73 YLMPGPVTV 7.93200 7.91524 0.01676

74 WLDQVPFSV 7.93900 7.95772 -0.01872

75 KTWGQYWQV 7.95500 7.67022 0.28478

76 ALMPLYACI 8.00000 7.44841 0.55159

77 YLAPGPVTA 8.03200 7.61068 0.42132

78 YLYPGPVTV 8.05100 8.31454 -0.26354

79 LLMGTLGIV 8.09700 7.68027 0.41673

80 YLWPGPVTV 8.12500 8.09316 0.03184

81 FLLTRILTI 8.14900 7.88930 0.25970

82 GLLGWSPQA 8.23700 8.17985 0.05715

83 ILYQVPFSV 8.31000 8.73294 -0.42294

84 GILTVILGV 8.34700 7.82101 0.52599

85 NMVPFFPPV 8.39800 8.08781 0.31019

86 ILDQVPFSV 8.48100 7.69102 0.78998

87 YLFPGPVTA 8.49500 8.32491 0.17009

88 YLDQVPFSV 8.63800 8.14125 0.49675

89 ILFQVPFSV 8.69900 8.44444 0.25456

90 ILWQVPFSV 8.77000 8.51156 0.25844

Correlation Coeffecient and Stand Error

CORL: 0.886726 CORL~2: 0.786282

RES: 0.367493 SEE: 0.038737

Statistical indices of Training calculation:

ITERATION (B): 20

No. Sequence Expt. Act Predicted Act Difference

1 VALVGLFVL 5.14800 5.72999 -0.58199

2 GTLVALVGL 5.34200 5.91367 -0.57167

3 LQTTIHDII 5.50100 5.77294 -0.27194

4 SLHVGTQCA 5.84200 6.11417 -0.27217

5 ALPYWNFAT 5.86900 6.65939 -0.79039

6 SLNFMGYVI 5.88100 5.98198 -0.10098

7 NLQSLTNLL 6.00000 6.69414 -0.69414

8 FVTWHRYHL 6.02500 5.73875 0.28625

9 DPKVKQWPL 6.17600 5.74057 0.43543

10 ITSQVPFSV 6.19600 6.55814 -0.36214

11 ALAKAAAAI 6.21100 6.22925 -0.01825

12 GLGQVPLIV 6.30100 6.56300 -0.26200

13 MLDLQPETT 6.33500 6.84633 -0.51133

14 LLSSNLSWL 6.34200 6.34940 -0.00740

15 GLACHQLCA 6.38000 6.01507 0.36493

16 LIGNESFAL 6.41500 7.04936 -0.63436

17 ALAKAAAAV 6.41900 6.45797 -0.03897

18 LLAVGATKV 6.47700 6.50005 -0.02305

19 ALAKAAAAL 6.51100 6.21156 0.29944

20 WILRGTSFV 6.55600 6.90602 -0.35002

21 IISCTCPTV 6.58000 6.65908 -0.07908

22 FLGGTPVCL 6.62300 6.88344 -0.26044

23 ALIHHNTHL 6.62300 6.78964 -0.16664

24 NLSWLSLDV 6.63900 6.04695 0.59205

25 YMIMVKCWM 6.66300 6.71573 -0.05273

26 VLQAGFFLL 6.68200 7.05310 -0.37110

27 GTLGIVCPI 6.71400 6.48843 0.22557

28 VILGVLLLI 6.78500 7.48430 -0.69930

29 VTWHRYHLL 6.79300 6.55202 0.24098

30 PLLPIFFCL 6.79600 7.54013 -0.74413

31 TLGIVCPIC 6.81500 5.97199 0.84301

32 CLTSTVQLV 6.83200 7.10795 -0.27595

33 ILLLCLIFL 6.84500 6.82784 0.01716

34 FAFRDLCIV 6.88600 6.64262 0.24338

35 FLEPGPVTA 6.89800 7.46451 -0.56651

36 ALAKAAAAA 6.94700 6.76171 0.18529

37 LMAVVLASL 6.95400 7.52469 -0.57069

38 YVITTQHWL 6.98300 6.33423 0.64877

39 LLCLIFLLV 6.99600 7.53922 -0.54322

40 ITAQVPFSV 7.02000 6.64194 0.37806

41 YLEPGPVTL 7.05800 7.15531 -0.09731

42 YTDQVPFSV 7.06600 7.05603 0.00997

43 NLYVSLLLL 7.11400 7.00299 0.11101

44 ILHNGAYSL 7.12700 7.35589 -0.22889

45 SIISAVVGI 7.15900 7.29539 -0.13639

46 VVMGTLVAL 7.17400 7.30569 -0.13169

47 YLEPGPVTI 7.18700 7.17300 0.01400

48 GLSRYVARL 7.24800 7.46181 -0.21381

49 LLAQFTSAI 7.30100 7.43897 -0.13797

50 VLLDYQGML 7.32800 7.60123 -0.27323

51 YLEPGPVTV 7.34200 7.40173 -0.05973

52 ILSPFMPLL 7.34700 7.15244 0.19456

53 YLSPGPVTA 7.38300 7.53193 -0.14893

54 IIDQVPFSV 7.39800 7.65288 -0.25488

55 SVYDFFVWL 7.44400 7.37116 0.07284

56 ITWQVPFSV 7.46300 7.42943 0.03357

57 ITYQVPFSV 7.48000 7.65255 -0.17255

58 GLYSSTVPV 7.48100 7.61386 -0.13286

59 VMGTLVALV 7.55300 7.24216 0.31084

60 LLLCLIFLL 7.58500 7.17026 0.41474

61 SLDDYNHLV 7.58500 7.15742 0.42758

62 VLIQRNPQL 7.64400 6.95494 0.68906

63 SLYADSPSV 7.65800 7.69767 -0.03967

64 ILSQVPFSV 7.69900 7.65144 0.04756

65 IMDQVPFSV 7.71900 8.05648 -0.33748

66 QLFEDNYAL 7.76400 7.48280 0.28120

67 ALMDKSLHV 7.77000 7.52001 0.24999

68 YAIDLPVSV 7.79600 7.57481 0.22119

69 FVWLHYYSV 7.82400 8.13613 -0.31213

70 MLGTHTMEV 7.84500 7.31145 0.53355

71 LLFGYPVYV 7.88600 8.05109 -0.16509

72 ILKEPVHGV 7.92100 7.59321 0.32779

73 YLMPGPVTV 7.93200 7.92016 0.01184

74 WLDQVPFSV 7.93900 7.96443 -0.02543

75 KTWGQYWQV 7.95500 7.67762 0.27738

76 ALMPLYACI 8.00000 7.44732 0.55268

77 YLAPGPVTA 8.03200 7.61572 0.41628

78 YLYPGPVTV 8.05100 8.32260 -0.27160

79 LLMGTLGIV 8.09700 7.68441 0.41259

80 YLWPGPVTV 8.12500 8.09948 0.02552

81 FLLTRILTI 8.14900 7.89309 0.25591

82 GLLGWSPQA 8.23700 8.19235 0.04465

83 ILYQVPFSV 8.31000 8.74585 -0.43585

84 GILTVILGV 8.34700 7.82717 0.51983

85 NMVPFFPPV 8.39800 8.09375 0.30425

86 ILDQVPFSV 8.48100 7.69575 0.78525

87 YLFPGPVTA 8.49500 8.33557 0.15943

88 YLDQVPFSV 8.63800 8.14933 0.48867

89 ILFQVPFSV 8.69900 8.45508 0.24392

90 ILWQVPFSV 8.77000 8.52273 0.24727

Correlation Coeffecient and Stand Error

CORL: 0.886794 CORL~2: 0.786403

RES: 0.367430 SEE: 0.038731

Statistical indices of Training calculation:

ITERATION (A): 21

No. Sequence Expt. Act Predicted Act Difference

1 VALVGLFVL 5.14800 5.74309 -0.59509

2 GTLVALVGL 5.34200 5.92458 -0.58258

3 LQTTIHDII 5.50100 5.78667 -0.28567

4 SLHVGTQCA 5.84200 6.12540 -0.28340

5 ALPYWNFAT 5.86900 6.66417 -0.79517

6 SLNFMGYVI 5.88100 5.99329 -0.11229

7 NLQSLTNLL 6.00000 6.69896 -0.69896

8 FVTWHRYHL 6.02500 5.75203 0.27297

9 DPKVKQWPL 6.17600 5.75200 0.42400

10 ITSQVPFSV 6.19600 6.56256 -0.36656

11 ALAKAAAAI 6.21100 6.23832 -0.02732

12 GLGQVPLIV 6.30100 6.56813 -0.26713

13 MLDLQPETT 6.33500 6.85006 -0.51506

14 LLSSNLSWL 6.34200 6.35764 -0.01564

15 GLACHQLCA 6.38000 6.02846 0.35154

16 LIGNESFAL 6.41500 7.05179 -0.63679

17 ALAKAAAAV 6.41900 6.46516 -0.04616

18 LLAVGATKV 6.47700 6.50678 -0.02978

19 ALAKAAAAL 6.51100 6.22091 0.29009

20 WILRGTSFV 6.55600 6.90917 -0.35317

21 IISCTCPTV 6.58000 6.66520 -0.08520

22 FLGGTPVCL 6.62300 6.88650 -0.26350

23 ALIHHNTHL 6.62300 6.79360 -0.17060

24 NLSWLSLDV 6.63900 6.05798 0.58102

25 YMIMVKCWM 6.66300 6.72019 -0.05719

26 VLQAGFFLL 6.68200 7.05518 -0.37318

27 GTLGIVCPI 6.71400 6.49515 0.21885

28 VILGVLLLI 6.78500 7.48259 -0.69759

29 VTWHRYHLL 6.79300 6.55737 0.23563

30 PLLPIFFCL 6.79600 7.53923 -0.74323

31 TLGIVCPIC 6.81500 5.98274 0.83226

32 CLTSTVQLV 6.83200 7.10796 -0.27596

33 ILLLCLIFL 6.84500 6.83125 0.01375

34 FAFRDLCIV 6.88600 6.64686 0.23914

35 FLEPGPVTA 6.89800 7.46393 -0.56593

36 ALAKAAAAA 6.94700 6.76639 0.18061

37 LMAVVLASL 6.95400 7.52140 -0.56740

38 YVITTQHWL 6.98300 6.34295 0.64005

39 LLCLIFLLV 6.99600 7.53605 -0.54005

40 ITAQVPFSV 7.02000 6.64536 0.37464

41 YLEPGPVTL 7.05800 7.15737 -0.09937

42 YTDQVPFSV 7.06600 7.05592 0.01008

43 NLYVSLLLL 7.11400 7.00543 0.10857

44 ILHNGAYSL 7.12700 7.35501 -0.22801

45 SIISAVVGI 7.15900 7.29523 -0.13623

46 VVMGTLVAL 7.17400 7.30493 -0.13093

47 YLEPGPVTI 7.18700 7.17478 0.01222

48 GLSRYVARL 7.24800 7.45967 -0.21167

49 LLAQFTSAI 7.30100 7.43680 -0.13580

50 VLLDYQGML 7.32800 7.59848 -0.27048

51 YLEPGPVTV 7.34200 7.40162 -0.05962

52 ILSPFMPLL 7.34700 7.15439 0.19261

53 YLSPGPVTA 7.38300 7.53092 -0.14792

54 IIDQVPFSV 7.39800 7.64885 -0.25085

55 SVYDFFVWL 7.44400 7.36995 0.07405

56 ITWQVPFSV 7.46300 7.42597 0.03703

57 ITYQVPFSV 7.48000 7.64716 -0.16716

58 GLYSSTVPV 7.48100 7.61154 -0.13054

59 VMGTLVALV 7.55300 7.24212 0.31088

60 LLLCLIFLL 7.58500 7.17238 0.41262

61 SLDDYNHLV 7.58500 7.15846 0.42654

62 VLIQRNPQL 7.64400 6.95727 0.68673

63 SLYADSPSV 7.65800 7.69385 -0.03585

64 ILSQVPFSV 7.69900 7.64712 0.05188

65 IMDQVPFSV 7.71900 8.04786 -0.32886

66 QLFEDNYAL 7.76400 7.48111 0.28289

67 ALMDKSLHV 7.77000 7.51694 0.25306

68 YAIDLPVSV 7.79600 7.57072 0.22528

69 FVWLHYYSV 7.82400 8.12818 -0.30418

70 MLGTHTMEV 7.84500 7.31107 0.53393

71 LLFGYPVYV 7.88600 8.04329 -0.15729

72 ILKEPVHGV 7.92100 7.58917 0.33183

73 YLMPGPVTV 7.93200 7.91519 0.01681

74 WLDQVPFSV 7.93900 7.95718 -0.01818

75 KTWGQYWQV 7.95500 7.67269 0.28231

76 ALMPLYACI 8.00000 7.44750 0.55250

77 YLAPGPVTA 8.03200 7.61371 0.41829

78 YLYPGPVTV 8.05100 8.31429 -0.26329

79 LLMGTLGIV 8.09700 7.68005 0.41695

80 YLWPGPVTV 8.12500 8.09309 0.03191

81 FLLTRILTI 8.14900 7.88845 0.26055

82 GLLGWSPQA 8.23700 8.18383 0.05317

83 ILYQVPFSV 8.31000 8.73172 -0.42172

84 GILTVILGV 8.34700 7.82316 0.52384

85 NMVPFFPPV 8.39800 8.08766 0.31034

86 ILDQVPFSV 8.48100 7.69104 0.78996

87 YLFPGPVTA 8.49500 8.32721 0.16779

88 YLDQVPFSV 8.63800 8.14048 0.49752

89 ILFQVPFSV 8.69900 8.44341 0.25559

90 ILWQVPFSV 8.77000 8.51053 0.25947

Correlation Coeffecient and Stand Error

CORL: 0.886803 CORL~2: 0.786420

RES: 0.367375 SEE: 0.038725

Statistical indices of Training calculation:

ITERATION (B): 21

No. Sequence Expt. Act Predicted Act Difference

1 VALVGLFVL 5.14800 5.73248 -0.58448

2 GTLVALVGL 5.34200 5.91603 -0.57403

3 LQTTIHDII 5.50100 5.77718 -0.27618

4 SLHVGTQCA 5.84200 6.11853 -0.27653

5 ALPYWNFAT 5.86900 6.65765 -0.78865

6 SLNFMGYVI 5.88100 5.97956 -0.09856

7 NLQSLTNLL 6.00000 6.69465 -0.69465

8 FVTWHRYHL 6.02500 5.73718 0.28782

9 DPKVKQWPL 6.17600 5.74044 0.43556

10 ITSQVPFSV 6.19600 6.56117 -0.36517

11 ALAKAAAAI 6.21100 6.23059 -0.01959

12 GLGQVPLIV 6.30100 6.56315 -0.26215

13 MLDLQPETT 6.33500 6.84737 -0.51237

14 LLSSNLSWL 6.34200 6.34951 -0.00751

15 GLACHQLCA 6.38000 6.01944 0.36056

16 LIGNESFAL 6.41500 7.05005 -0.63505

17 ALAKAAAAV 6.41900 6.46070 -0.04170

18 LLAVGATKV 6.47700 6.50124 -0.02424

19 ALAKAAAAL 6.51100 6.21293 0.29807

20 WILRGTSFV 6.55600 6.90623 -0.35023

21 IISCTCPTV 6.58000 6.65963 -0.07963

22 FLGGTPVCL 6.62300 6.88253 -0.25953

23 ALIHHNTHL 6.62300 6.78972 -0.16672

24 NLSWLSLDV 6.63900 6.04704 0.59196

25 YMIMVKCWM 6.66300 6.70839 -0.04539

26 VLQAGFFLL 6.68200 7.05187 -0.36987

27 GTLGIVCPI 6.71400 6.49204 0.22196

28 VILGVLLLI 6.78500 7.48317 -0.69817

29 VTWHRYHLL 6.79300 6.55275 0.24025

30 PLLPIFFCL 6.79600 7.53833 -0.74233

31 TLGIVCPIC 6.81500 5.96971 0.84529

32 CLTSTVQLV 6.83200 7.10777 -0.27577

33 ILLLCLIFL 6.84500 6.82337 0.02163

34 FAFRDLCIV 6.88600 6.64521 0.24079

35 FLEPGPVTA 6.89800 7.46748 -0.56948

36 ALAKAAAAA 6.94700 6.76626 0.18074

37 LMAVVLASL 6.95400 7.52130 -0.56730

38 YVITTQHWL 6.98300 6.33483 0.64817

39 LLCLIFLLV 6.99600 7.53555 -0.53955

40 ITAQVPFSV 7.02000 6.64454 0.37546

41 YLEPGPVTL 7.05800 7.15465 -0.09665

42 YTDQVPFSV 7.06600 7.05782 0.00818

43 NLYVSLLLL 7.11400 7.00040 0.11360

44 ILHNGAYSL 7.12700 7.35527 -0.22827

45 SIISAVVGI 7.15900 7.29631 -0.13731

46 VVMGTLVAL 7.17400 7.30670 -0.13270

47 YLEPGPVTI 7.18700 7.17231 0.01469

48 GLSRYVARL 7.24800 7.46176 -0.21376

49 LLAQFTSAI 7.30100 7.43815 -0.13715

50 VLLDYQGML 7.32800 7.60022 -0.27222

51 YLEPGPVTV 7.34200 7.40242 -0.06042

52 ILSPFMPLL 7.34700 7.15127 0.19573

53 YLSPGPVTA 7.38300 7.53485 -0.15185

54 IIDQVPFSV 7.39800 7.65285 -0.25485

55 SVYDFFVWL 7.44400 7.37043 0.07357

56 ITWQVPFSV 7.46300 7.43062 0.03238

57 ITYQVPFSV 7.48000 7.65336 -0.17336

58 GLYSSTVPV 7.48100 7.61556 -0.13456

59 VMGTLVALV 7.55300 7.24156 0.31144

60 LLLCLIFLL 7.58500 7.16736 0.41764

61 SLDDYNHLV 7.58500 7.15934 0.42566

62 VLIQRNPQL 7.64400 6.95414 0.68986

63 SLYADSPSV 7.65800 7.69895 -0.04095

64 ILSQVPFSV 7.69900 7.65101 0.04799

65 IMDQVPFSV 7.71900 8.05380 -0.33480

66 QLFEDNYAL 7.76400 7.48161 0.28239

67 ALMDKSLHV 7.77000 7.52052 0.24948

68 YAIDLPVSV 7.79600 7.57806 0.21794

69 FVWLHYYSV 7.82400 8.13401 -0.31001

70 MLGTHTMEV 7.84500 7.31213 0.53287

71 LLFGYPVYV 7.88600 8.04855 -0.16255

72 ILKEPVHGV 7.92100 7.59299 0.32801

73 YLMPGPVTV 7.93200 7.91959 0.01241

74 WLDQVPFSV 7.93900 7.96315 -0.02415

75 KTWGQYWQV 7.95500 7.67926 0.27574

76 ALMPLYACI 8.00000 7.44655 0.55345

77 YLAPGPVTA 8.03200 7.61823 0.41377

78 YLYPGPVTV 8.05100 8.32148 -0.27048

79 LLMGTLGIV 8.09700 7.68375 0.41325

80 YLWPGPVTV 8.12500 8.09874 0.02626

81 FLLTRILTI 8.14900 7.89184 0.25716

82 GLLGWSPQA 8.23700 8.19497 0.04203

83 ILYQVPFSV 8.31000 8.74321 -0.43321

84 GILTVILGV 8.34700 7.82866 0.51834

85 NMVPFFPPV 8.39800 8.09297 0.30503

86 ILDQVPFSV 8.48100 7.69524 0.78576

87 YLFPGPVTA 8.49500 8.33672 0.15828

88 YLDQVPFSV 8.63800 8.14767 0.49033

89 ILFQVPFSV 8.69900 8.45288 0.24612

90 ILWQVPFSV 8.77000 8.52047 0.24953

Correlation Coeffecient and Stand Error

CORL: 0.886856 CORL~2: 0.786514

RES: 0.367325 SEE: 0.038719

Statistical indices of Training calculation:

ITERATION (A): 22

No. Sequence Expt. Act Predicted Act Difference

1 VALVGLFVL 5.14800 5.74418 -0.59618

2 GTLVALVGL 5.34200 5.92577 -0.58377

3 LQTTIHDII 5.50100 5.78939 -0.28839

4 SLHVGTQCA 5.84200 6.12850 -0.28650

5 ALPYWNFAT 5.86900 6.66194 -0.79294

6 SLNFMGYVI 5.88100 5.98965 -0.10865

7 NLQSLTNLL 6.00000 6.69895 -0.69895

8 FVTWHRYHL 6.02500 5.74908 0.27592

9 DPKVKQWPL 6.17600 5.75070 0.42530

10 ITSQVPFSV 6.19600 6.56512 -0.36912

11 ALAKAAAAI 6.21100 6.23869 -0.02769

12 GLGQVPLIV 6.30100 6.56775 -0.26675

13 MLDLQPETT 6.33500 6.85070 -0.51570

14 LLSSNLSWL 6.34200 6.35685 -0.01485

15 GLACHQLCA 6.38000 6.03134 0.34866

16 LIGNESFAL 6.41500 7.05221 -0.63721

17 ALAKAAAAV 6.41900 6.46710 -0.04810

18 LLAVGATKV 6.47700 6.50723 -0.03023

19 ALAKAAAAL 6.51100 6.22129 0.28971

20 WILRGTSFV 6.55600 6.90905 -0.35305

21 IISCTCPTV 6.58000 6.66509 -0.08509

22 FLGGTPVCL 6.62300 6.88529 -0.26229

23 ALIHHNTHL 6.62300 6.79326 -0.17026

24 NLSWLSLDV 6.63900 6.05688 0.58212

25 YMIMVKCWM 6.66300 6.71244 -0.04944

26 VLQAGFFLL 6.68200 7.05374 -0.37174

27 GTLGIVCPI 6.71400 6.49800 0.21600

28 VILGVLLLI 6.78500 7.48165 -0.69665

29 VTWHRYHLL 6.79300 6.55755 0.23545

30 PLLPIFFCL 6.79600 7.53753 -0.74153

31 TLGIVCPIC 6.81500 5.97935 0.83565

32 CLTSTVQLV 6.83200 7.10778 -0.27578

33 ILLLCLIFL 6.84500 6.82643 0.01857

34 FAFRDLCIV 6.88600 6.64901 0.23699

35 FLEPGPVTA 6.89800 7.46691 -0.56891

36 ALAKAAAAA 6.94700 6.77040 0.17660

37 LMAVVLASL 6.95400 7.51838 -0.56438

38 YVITTQHWL 6.98300 6.34263 0.64037

39 LLCLIFLLV 6.99600 7.53274 -0.53674

40 ITAQVPFSV 7.02000 6.64762 0.37238

41 YLEPGPVTL 7.05800 7.15648 -0.09848

42 YTDQVPFSV 7.06600 7.05774 0.00826

43 NLYVSLLLL 7.11400 7.00260 0.11140

44 ILHNGAYSL 7.12700 7.35448 -0.22748

45 SIISAVVGI 7.15900 7.29616 -0.13716

46 VVMGTLVAL 7.17400 7.30603 -0.13203

47 YLEPGPVTI 7.18700 7.17388 0.01312

48 GLSRYVARL 7.24800 7.45988 -0.21188

49 LLAQFTSAI 7.30100 7.43621 -0.13521

50 VLLDYQGML 7.32800 7.59778 -0.26978

51 YLEPGPVTV 7.34200 7.40229 -0.06029

52 ILSPFMPLL 7.34700 7.15301 0.19399

53 YLSPGPVTA 7.38300 7.53390 -0.15090

54 IIDQVPFSV 7.39800 7.64926 -0.25126

55 SVYDFFVWL 7.44400 7.36940 0.07460

56 ITWQVPFSV 7.46300 7.42755 0.03545

57 ITYQVPFSV 7.48000 7.64857 -0.16857

58 GLYSSTVPV 7.48100 7.61343 -0.13243

59 VMGTLVALV 7.55300 7.24153 0.31147

60 LLLCLIFLL 7.58500 7.16926 0.41574

61 SLDDYNHLV 7.58500 7.16025 0.42475

62 VLIQRNPQL 7.64400 6.95622 0.68778

63 SLYADSPSV 7.65800 7.69551 -0.03751

64 ILSQVPFSV 7.69900 7.64716 0.05184

65 IMDQVPFSV 7.71900 8.04614 -0.32714

66 QLFEDNYAL 7.76400 7.48011 0.28389

67 ALMDKSLHV 7.77000 7.51776 0.25224

68 YAIDLPVSV 7.79600 7.57441 0.22159

69 FVWLHYYSV 7.82400 8.12694 -0.30294

70 MLGTHTMEV 7.84500 7.31177 0.53323

71 LLFGYPVYV 7.88600 8.04159 -0.15559

72 ILKEPVHGV 7.92100 7.58939 0.33161

73 YLMPGPVTV 7.93200 7.91513 0.01687

74 WLDQVPFSV 7.93900 7.95669 -0.01769

75 KTWGQYWQV 7.95500 7.67485 0.28015

76 ALMPLYACI 8.00000 7.44668 0.55332

77 YLAPGPVTA 8.03200 7.61639 0.41561

78 YLYPGPVTV 8.05100 8.31404 -0.26304

79 LLMGTLGIV 8.09700 7.67984 0.41716

80 YLWPGPVTV 8.12500 8.09302 0.03198

81 FLLTRILTI 8.14900 7.88768 0.26132

82 GLLGWSPQA 8.23700 8.18733 0.04967

83 ILYQVPFSV 8.31000 8.73062 -0.42062

84 GILTVILGV 8.34700 7.82504 0.52196

85 NMVPFFPPV 8.39800 8.08751 0.31049

86 ILDQVPFSV 8.48100 7.69103 0.78997

87 YLFPGPVTA 8.49500 8.32922 0.16578

88 YLDQVPFSV 8.63800 8.13978 0.49822

89 ILFQVPFSV 8.69900 8.44249 0.25651

90 ILWQVPFSV 8.77000 8.50959 0.26041

Correlation Coeffecient and Stand Error

CORL: 0.886865 CORL~2: 0.786529

RES: 0.367281 SEE: 0.038715

Statistical indices of Training calculation:

ITERATION (B): 22

No. Sequence Expt. Act Predicted Act Difference

1 VALVGLFVL 5.14800 5.73468 -0.58668

2 GTLVALVGL 5.34200 5.91811 -0.57611

3 LQTTIHDII 5.50100 5.78091 -0.27991

4 SLHVGTQCA 5.84200 6.12238 -0.28038

5 ALPYWNFAT 5.86900 6.65610 -0.78710

6 SLNFMGYVI 5.88100 5.97737 -0.09637

7 NLQSLTNLL 6.00000 6.69510 -0.69510

8 FVTWHRYHL 6.02500 5.73579 0.28921

9 DPKVKQWPL 6.17600 5.74036 0.43564

10 ITSQVPFSV 6.19600 6.56385 -0.36785

11 ALAKAAAAI 6.21100 6.23179 -0.02079

12 GLGQVPLIV 6.30100 6.56330 -0.26230

13 MLDLQPETT 6.33500 6.84829 -0.51329

14 LLSSNLSWL 6.34200 6.34960 -0.00760

15 GLACHQLCA 6.38000 6.02331 0.35669

16 LIGNESFAL 6.41500 7.05066 -0.63566

17 ALAKAAAAV 6.41900 6.46312 -0.04412

18 LLAVGATKV 6.47700 6.50228 -0.02528

19 ALAKAAAAL 6.51100 6.21417 0.29683

20 WILRGTSFV 6.55600 6.90643 -0.35043

21 IISCTCPTV 6.58000 6.66012 -0.08012

22 FLGGTPVCL 6.62300 6.88175 -0.25875

23 ALIHHNTHL 6.62300 6.78980 -0.16680

24 NLSWLSLDV 6.63900 6.04710 0.59190

25 YMIMVKCWM 6.66300 6.70189 -0.03889

26 VLQAGFFLL 6.68200 7.05079 -0.36879

27 GTLGIVCPI 6.71400 6.49521 0.21879

28 VILGVLLLI 6.78500 7.48217 -0.69717

29 VTWHRYHLL 6.79300 6.55341 0.23959

30 PLLPIFFCL 6.79600 7.53673 -0.74073

31 TLGIVCPIC 6.81500 5.96771 0.84729

32 CLTSTVQLV 6.83200 7.10762 -0.27562

33 ILLLCLIFL 6.84500 6.81938 0.02562

34 FAFRDLCIV 6.88600 6.64751 0.23849

35 FLEPGPVTA 6.89800 7.47010 -0.57210

36 ALAKAAAAA 6.94700 6.77031 0.17669

37 LMAVVLASL 6.95400 7.51830 -0.56430

38 YVITTQHWL 6.98300 6.33537 0.64763

39 LLCLIFLLV 6.99600 7.53229 -0.53629

40 ITAQVPFSV 7.02000 6.64686 0.37314

41 YLEPGPVTL 7.05800 7.15406 -0.09606

42 YTDQVPFSV 7.06600 7.05941 0.00659

43 NLYVSLLLL 7.11400 6.99811 0.11589

44 ILHNGAYSL 7.12700 7.35471 -0.22771

45 SIISAVVGI 7.15900 7.29713 -0.13813

46 VVMGTLVAL 7.17400 7.30760 -0.13360

47 YLEPGPVTI 7.18700 7.17169 0.01531

48 GLSRYVARL 7.24800 7.46173 -0.21373

49 LLAQFTSAI 7.30100 7.43742 -0.13642

50 VLLDYQGML 7.32800 7.59934 -0.27134

51 YLEPGPVTV 7.34200 7.40302 -0.06102

52 ILSPFMPLL 7.34700 7.15022 0.19678

53 YLSPGPVTA 7.38300 7.53743 -0.15443

54 IIDQVPFSV 7.39800 7.65283 -0.25483

55 SVYDFFVWL 7.44400 7.36982 0.07418

56 ITWQVPFSV 7.46300 7.43168 0.03132

57 ITYQVPFSV 7.48000 7.65409 -0.17409

58 GLYSSTVPV 7.48100 7.61705 -0.13605

59 VMGTLVALV 7.55300 7.24104 0.31196

60 LLLCLIFLL 7.58500 7.16478 0.42022

61 SLDDYNHLV 7.58500 7.16103 0.42397

62 VLIQRNPQL 7.64400 6.95343 0.69057

63 SLYADSPSV 7.65800 7.70009 -0.04209

64 ILSQVPFSV 7.69900 7.65063 0.04837

65 IMDQVPFSV 7.71900 8.05145 -0.33245

66 QLFEDNYAL 7.76400 7.48057 0.28343

67 ALMDKSLHV 7.77000 7.52096 0.24904

68 YAIDLPVSV 7.79600 7.58094 0.21506

69 FVWLHYYSV 7.82400 8.13213 -0.30813

70 MLGTHTMEV 7.84500 7.31272 0.53228

71 LLFGYPVYV 7.88600 8.04629 -0.16029

72 ILKEPVHGV 7.92100 7.59280 0.32820

73 YLMPGPVTV 7.93200 7.91908 0.01292

74 WLDQVPFSV 7.93900 7.96201 -0.02301

75 KTWGQYWQV 7.95500 7.68070 0.27430

76 ALMPLYACI 8.00000 7.44585 0.55415

77 YLAPGPVTA 8.03200 7.62044 0.41156

78 YLYPGPVTV 8.05100 8.32049 -0.26949

79 LLMGTLGIV 8.09700 7.68315 0.41385

80 YLWPGPVTV 8.12500 8.09808 0.02692

81 FLLTRILTI 8.14900 7.89072 0.25828

82 GLLGWSPQA 8.23700 8.19729 0.03971

83 ILYQVPFSV 8.31000 8.74088 -0.43088

84 GILTVILGV 8.34700 7.82996 0.51704

85 NMVPFFPPV 8.39800 8.09227 0.30573

86 ILDQVPFSV 8.48100 7.69478 0.78622

87 YLFPGPVTA 8.49500 8.33774 0.15726

88 YLDQVPFSV 8.63800 8.14620 0.49180

89 ILFQVPFSV 8.69900 8.45095 0.24805

90 ILWQVPFSV 8.77000 8.51847 0.25153

Correlation Coeffecient and Stand Error

CORL: 0.886906 CORL~2: 0.786603

RES: 0.367241 SEE: 0.038711

Statistical indices of Training calculation:

ITERATION (A): 23

No. Sequence Expt. Act Predicted Act Difference

1 VALVGLFVL 5.14800 5.74515 -0.59715

2 GTLVALVGL 5.34200 5.92683 -0.58483

3 LQTTIHDII 5.50100 5.79181 -0.29081

4 SLHVGTQCA 5.84200 6.13128 -0.28928

5 ALPYWNFAT 5.86900 6.65996 -0.79096

6 SLNFMGYVI 5.88100 5.98642 -0.10542

7 NLQSLTNLL 6.00000 6.69894 -0.69894

8 FVTWHRYHL 6.02500 5.74647 0.27853

9 DPKVKQWPL 6.17600 5.74958 0.42642

10 ITSQVPFSV 6.19600 6.56740 -0.37140

11 ALAKAAAAI 6.21100 6.23904 -0.02804

12 GLGQVPLIV 6.30100 6.56743 -0.26643

13 MLDLQPETT 6.33500 6.85126 -0.51626

14 LLSSNLSWL 6.34200 6.35617 -0.01417

15 GLACHQLCA 6.38000 6.03393 0.34607

16 LIGNESFAL 6.41500 7.05258 -0.63758

17 ALAKAAAAV 6.41900 6.46883 -0.04983

18 LLAVGATKV 6.47700 6.50763 -0.03063

19 ALAKAAAAL 6.51100 6.22165 0.28935

20 WILRGTSFV 6.55600 6.90894 -0.35294

21 IISCTCPTV 6.58000 6.66502 -0.08502

22 FLGGTPVCL 6.62300 6.88425 -0.26125

23 ALIHHNTHL 6.62300 6.79297 -0.16997

24 NLSWLSLDV 6.63900 6.05590 0.58310

25 YMIMVKCWM 6.66300 6.70558 -0.04258

26 VLQAGFFLL 6.68200 7.05248 -0.37048

27 GTLGIVCPI 6.71400 6.50052 0.21348

28 VILGVLLLI 6.78500 7.48081 -0.69581

29 VTWHRYHLL 6.79300 6.55773 0.23527

30 PLLPIFFCL 6.79600 7.53601 -0.74001

31 TLGIVCPIC 6.81500 5.97638 0.83862

32 CLTSTVQLV 6.83200 7.10763 -0.27563

33 ILLLCLIFL 6.84500 6.82214 0.02286

34 FAFRDLCIV 6.88600 6.65091 0.23509

35 FLEPGPVTA 6.89800 7.46955 -0.57155

36 ALAKAAAAA 6.94700 6.77398 0.17302

37 LMAVVLASL 6.95400 7.51571 -0.56171

38 YVITTQHWL 6.98300 6.34237 0.64063

39 LLCLIFLLV 6.99600 7.52979 -0.53379

40 ITAQVPFSV 7.02000 6.64962 0.37038

41 YLEPGPVTL 7.05800 7.15569 -0.09769

42 YTDQVPFSV 7.06600 7.05935 0.00665

43 NLYVSLLLL 7.11400 7.00009 0.11391

44 ILHNGAYSL 7.12700 7.35400 -0.22700

45 SIISAVVGI 7.15900 7.29698 -0.13798

46 VVMGTLVAL 7.17400 7.30701 -0.13301

47 YLEPGPVTI 7.18700 7.17308 0.01392

48 GLSRYVARL 7.24800 7.46006 -0.21206

49 LLAQFTSAI 7.30100 7.43568 -0.13468

50 VLLDYQGML 7.32800 7.59715 -0.26915

51 YLEPGPVTV 7.34200 7.40287 -0.06087

52 ILSPFMPLL 7.34700 7.15178 0.19522

53 YLSPGPVTA 7.38300 7.53654 -0.15354

54 IIDQVPFSV 7.39800 7.64962 -0.25162

55 SVYDFFVWL 7.44400 7.36893 0.07507

56 ITWQVPFSV 7.46300 7.42895 0.03405

57 ITYQVPFSV 7.48000 7.64982 -0.16982

58 GLYSSTVPV 7.48100 7.61511 -0.13411

59 VMGTLVALV 7.55300 7.24102 0.31198

60 LLLCLIFLL 7.58500 7.16649 0.41851

61 SLDDYNHLV 7.58500 7.16183 0.42317

62 VLIQRNPQL 7.64400 6.95531 0.68869

63 SLYADSPSV 7.65800 7.69699 -0.03899

64 ILSQVPFSV 7.69900 7.64720 0.05180

65 IMDQVPFSV 7.71900 8.04462 -0.32562

66 QLFEDNYAL 7.76400 7.47923 0.28477

67 ALMDKSLHV 7.77000 7.51849 0.25151

68 YAIDLPVSV 7.79600 7.57768 0.21832

69 FVWLHYYSV 7.82400 8.12583 -0.30183

70 MLGTHTMEV 7.84500 7.31239 0.53261

71 LLFGYPVYV 7.88600 8.04008 -0.15408

72 ILKEPVHGV 7.92100 7.58959 0.33141

73 YLMPGPVTV 7.93200 7.91507 0.01693

74 WLDQVPFSV 7.93900 7.95624 -0.01724

75 KTWGQYWQV 7.95500 7.67675 0.27825

76 ALMPLYACI 8.00000 7.44594 0.55406

77 YLAPGPVTA 8.03200 7.61877 0.41323

78 YLYPGPVTV 8.05100 8.31382 -0.26282

79 LLMGTLGIV 8.09700 7.67963 0.41737

80 YLWPGPVTV 8.12500 8.09294 0.03206

81 FLLTRILTI 8.14900 7.88698 0.26202

82 GLLGWSPQA 8.23700 8.19042 0.04658

83 ILYQVPFSV 8.31000 8.72962 -0.41962

84 GILTVILGV 8.34700 7.82669 0.52031

85 NMVPFFPPV 8.39800 8.08737 0.31063

86 ILDQVPFSV 8.48100 7.69102 0.78998

87 YLFPGPVTA 8.49500 8.33101 0.16399

88 YLDQVPFSV 8.63800 8.13915 0.49885

89 ILFQVPFSV 8.69900 8.44166 0.25734

90 ILWQVPFSV 8.77000 8.50875 0.26125

Correlation Coeffecient and Stand Error

CORL: 0.886914 CORL~2: 0.786616

RES: 0.367205 SEE: 0.038707

Statistical indices of Training calculation:

ITERATION (B): 23

No. Sequence Expt. Act Predicted Act Difference

1 VALVGLFVL 5.14800 5.73662 -0.58862

2 GTLVALVGL 5.34200 5.91995 -0.57795

3 LQTTIHDII 5.50100 5.78420 -0.28320

4 SLHVGTQCA 5.84200 6.12581 -0.28381

5 ALPYWNFAT 5.86900 6.65472 -0.78572

6 SLNFMGYVI 5.88100 5.97541 -0.09441

7 NLQSLTNLL 6.00000 6.69550 -0.69550

8 FVTWHRYHL 6.02500 5.73455 0.29045

9 DPKVKQWPL 6.17600 5.74030 0.43570

10 ITSQVPFSV 6.19600 6.56623 -0.37023

11 ALAKAAAAI 6.21100 6.23286 -0.02186

12 GLGQVPLIV 6.30100 6.56343 -0.26243

13 MLDLQPETT 6.33500 6.84911 -0.51411

14 LLSSNLSWL 6.34200 6.34968 -0.00768

15 GLACHQLCA 6.38000 6.02675 0.35325

16 LIGNESFAL 6.41500 7.05119 -0.63619

17 ALAKAAAAV 6.41900 6.46528 -0.04628

18 LLAVGATKV 6.47700 6.50320 -0.02620

19 ALAKAAAAL 6.51100 6.21527 0.29573

20 WILRGTSFV 6.55600 6.90661 -0.35061

21 IISCTCPTV 6.58000 6.66057 -0.08057

22 FLGGTPVCL 6.62300 6.88108 -0.25808

23 ALIHHNTHL 6.62300 6.78987 -0.16687

24 NLSWLSLDV 6.63900 6.04713 0.59187

25 YMIMVKCWM 6.66300 6.69614 -0.03314

26 VLQAGFFLL 6.68200 7.04983 -0.36783

27 GTLGIVCPI 6.71400 6.49801 0.21599

28 VILGVLLLI 6.78500 7.48128 -0.69628

29 VTWHRYHLL 6.79300 6.55400 0.23900

30 PLLPIFFCL 6.79600 7.53531 -0.73931

31 TLGIVCPIC 6.81500 5.96595 0.84905

32 CLTSTVQLV 6.83200 7.10748 -0.27548

33 ILLLCLIFL 6.84500 6.81582 0.02918

34 FAFRDLCIV 6.88600 6.64955 0.23645

35 FLEPGPVTA 6.89800 7.47243 -0.57443

36 ALAKAAAAA 6.94700 6.77391 0.17309

37 LMAVVLASL 6.95400 7.51563 -0.56163

38 YVITTQHWL 6.98300 6.33587 0.64713

39 LLCLIFLLV 6.99600 7.52937 -0.53337

40 ITAQVPFSV 7.02000 6.64892 0.37108

41 YLEPGPVTL 7.05800 7.15353 -0.09553

42 YTDQVPFSV 7.06600 7.06082 0.00518

43 NLYVSLLLL 7.11400 6.99608 0.11792

44 ILHNGAYSL 7.12700 7.35421 -0.22721

45 SIISAVVGI 7.15900 7.29786 -0.13886

46 VVMGTLVAL 7.17400 7.30841 -0.13441

47 YLEPGPVTI 7.18700 7.17112 0.01588

48 GLSRYVARL 7.24800 7.46172 -0.21372

49 LLAQFTSAI 7.30100 7.43675 -0.13575

50 VLLDYQGML 7.32800 7.59855 -0.27055

51 YLEPGPVTV 7.34200 7.40353 -0.06153

52 ILSPFMPLL 7.34700 7.14928 0.19772

53 YLSPGPVTA 7.38300 7.53972 -0.15672

54 IIDQVPFSV 7.39800 7.65281 -0.25481

55 SVYDFFVWL 7.44400 7.36930 0.07470

56 ITWQVPFSV 7.46300 7.43263 0.03037

57 ITYQVPFSV 7.48000 7.65475 -0.17475

58 GLYSSTVPV 7.48100 7.61836 -0.13736

59 VMGTLVALV 7.55300 7.24059 0.31241

60 LLLCLIFLL 7.58500 7.16248 0.42252

61 SLDDYNHLV 7.58500 7.16253 0.42247

62 VLIQRNPQL 7.64400 6.95281 0.69119

63 SLYADSPSV 7.65800 7.70110 -0.04310

64 ILSQVPFSV 7.69900 7.65030 0.04870

65 IMDQVPFSV 7.71900 8.04937 -0.33037

66 QLFEDNYAL 7.76400 7.47964 0.28436

67 ALMDKSLHV 7.77000 7.52136 0.24864

68 YAIDLPVSV 7.79600 7.58350 0.21250

69 FVWLHYYSV 7.82400 8.13047 -0.30647

70 MLGTHTMEV 7.84500 7.31325 0.53175

71 LLFGYPVYV 7.88600 8.04428 -0.15828

72 ILKEPVHGV 7.92100 7.59264 0.32836

73 YLMPGPVTV 7.93200 7.91862 0.01338

74 WLDQVPFSV 7.93900 7.96100 -0.02200

75 KTWGQYWQV 7.95500 7.68196 0.27304

76 ALMPLYACI 8.00000 7.44520 0.55480

77 YLAPGPVTA 8.03200 7.62241 0.40959

78 YLYPGPVTV 8.05100 8.31961 -0.26861

79 LLMGTLGIV 8.09700 7.68260 0.41440

80 YLWPGPVTV 8.12500 8.09749 0.02751

81 FLLTRILTI 8.14900 7.88971 0.25929

82 GLLGWSPQA 8.23700 8.19934 0.03766

83 ILYQVPFSV 8.31000 8.73882 -0.42882

84 GILTVILGV 8.34700 7.83111 0.51589

85 NMVPFFPPV 8.39800 8.09164 0.30636

86 ILDQVPFSV 8.48100 7.69437 0.78663

87 YLFPGPVTA 8.49500 8.33866 0.15634

88 YLDQVPFSV 8.63800 8.14489 0.49311

89 ILFQVPFSV 8.69900 8.44923 0.24977

90 ILWQVPFSV 8.77000 8.51670 0.25330

Correlation Coeffecient and Stand Error

CORL: 0.886947 CORL~2: 0.786675

RES: 0.367173 SEE: 0.038703

Statistical indices of Training calculation:

ITERATION (A): 24

No. Sequence Expt. Act Predicted Act Difference

1 VALVGLFVL 5.14800 5.74602 -0.59802

2 GTLVALVGL 5.34200 5.92777 -0.58577

3 LQTTIHDII 5.50100 5.79397 -0.29297

4 SLHVGTQCA 5.84200 6.13377 -0.29177

5 ALPYWNFAT 5.86900 6.65820 -0.78920

6 SLNFMGYVI 5.88100 5.98354 -0.10254

7 NLQSLTNLL 6.00000 6.69895 -0.69895

8 FVTWHRYHL 6.02500 5.74417 0.28083

9 DPKVKQWPL 6.17600 5.74861 0.42739

10 ITSQVPFSV 6.19600 6.56943 -0.37343

11 ALAKAAAAI 6.21100 6.23936 -0.02836

12 GLGQVPLIV 6.30100 6.56715 -0.26615

13 MLDLQPETT 6.33500 6.85178 -0.51678

14 LLSSNLSWL 6.34200 6.35556 -0.01356

15 GLACHQLCA 6.38000 6.03626 0.34374

16 LIGNESFAL 6.41500 7.05290 -0.63790

17 ALAKAAAAV 6.41900 6.47039 -0.05139

18 LLAVGATKV 6.47700 6.50798 -0.03098

19 ALAKAAAAL 6.51100 6.22199 0.28901

20 WILRGTSFV 6.55600 6.90886 -0.35286

21 IISCTCPTV 6.58000 6.66496 -0.08496

22 FLGGTPVCL 6.62300 6.88333 -0.26033

23 ALIHHNTHL 6.62300 6.79272 -0.16972

24 NLSWLSLDV 6.63900 6.05502 0.58398

25 YMIMVKCWM 6.66300 6.69949 -0.03649

26 VLQAGFFLL 6.68200 7.05136 -0.36936

27 GTLGIVCPI 6.71400 6.50275 0.21125

28 VILGVLLLI 6.78500 7.48005 -0.69505

29 VTWHRYHLL 6.79300 6.55789 0.23511

30 PLLPIFFCL 6.79600 7.53466 -0.73866

31 TLGIVCPIC 6.81500 5.97375 0.84125

32 CLTSTVQLV 6.83200 7.10749 -0.27549

33 ILLLCLIFL 6.84500 6.81831 0.02669

34 FAFRDLCIV 6.88600 6.65261 0.23339

35 FLEPGPVTA 6.89800 7.47190 -0.57390

36 ALAKAAAAA 6.94700 6.77718 0.16982

37 LMAVVLASL 6.95400 7.51333 -0.55933

38 YVITTQHWL 6.98300 6.34216 0.64084

39 LLCLIFLLV 6.99600 7.52715 -0.53115

40 ITAQVPFSV 7.02000 6.65141 0.36859

41 YLEPGPVTL 7.05800 7.15498 -0.09698

42 YTDQVPFSV 7.06600 7.06078 0.00522

43 NLYVSLLLL 7.11400 6.99786 0.11614

44 ILHNGAYSL 7.12700 7.35357 -0.22657

45 SIISAVVGI 7.15900 7.29772 -0.13872

46 VVMGTLVAL 7.17400 7.30789 -0.13389

47 YLEPGPVTI 7.18700 7.17236 0.01464

48 GLSRYVARL 7.24800 7.46023 -0.21223

49 LLAQFTSAI 7.30100 7.43520 -0.13420

50 VLLDYQGML 7.32800 7.59659 -0.26859

51 YLEPGPVTV 7.34200 7.40338 -0.06138

52 ILSPFMPLL 7.34700 7.15067 0.19633

53 YLSPGPVTA 7.38300 7.53889 -0.15589

54 IIDQVPFSV 7.39800 7.64994 -0.25194

55 SVYDFFVWL 7.44400 7.36853 0.07547

56 ITWQVPFSV 7.46300 7.43019 0.03281

57 ITYQVPFSV 7.48000 7.65093 -0.17093

58 GLYSSTVPV 7.48100 7.61659 -0.13559

59 VMGTLVALV 7.55300 7.24058 0.31242

60 LLLCLIFLL 7.58500 7.16402 0.42098

61 SLDDYNHLV 7.58500 7.16324 0.42176

62 VLIQRNPQL 7.64400 6.95451 0.68949

63 SLYADSPSV 7.65800 7.69830 -0.04030

64 ILSQVPFSV 7.69900 7.64722 0.05178

65 IMDQVPFSV 7.71900 8.04326 -0.32426

66 QLFEDNYAL 7.76400 7.47845 0.28555

67 ALMDKSLHV 7.77000 7.51913 0.25087

68 YAIDLPVSV 7.79600 7.58057 0.21543

69 FVWLHYYSV 7.82400 8.12483 -0.30083

70 MLGTHTMEV 7.84500 7.31294 0.53206

71 LLFGYPVYV 7.88600 8.03871 -0.15271

72 ILKEPVHGV 7.92100 7.58977 0.33123

73 YLMPGPVTV 7.93200 7.91500 0.01700

74 WLDQVPFSV 7.93900 7.95582 -0.01682

75 KTWGQYWQV 7.95500 7.67841 0.27659

76 ALMPLYACI 8.00000 7.44526 0.55474

77 YLAPGPVTA 8.03200 7.62088 0.41112

78 YLYPGPVTV 8.05100 8.31361 -0.26261

79 LLMGTLGIV 8.09700 7.67943 0.41757

80 YLWPGPVTV 8.12500 8.09286 0.03214

81 FLLTRILTI 8.14900 7.88633 0.26267

82 GLLGWSPQA 8.23700 8.19316 0.04384

83 ILYQVPFSV 8.31000 8.72873 -0.41873

84 GILTVILGV 8.34700 7.82816 0.51884

85 NMVPFFPPV 8.39800 8.08723 0.31077

86 ILDQVPFSV 8.48100 7.69100 0.79000

87 YLFPGPVTA 8.49500 8.33259 0.16241

88 YLDQVPFSV 8.63800 8.13857 0.49943

89 ILFQVPFSV 8.69900 8.44091 0.25809

90 ILWQVPFSV 8.77000 8.50798 0.26202

Correlation Coeffecient and Stand Error

CORL: 0.886954 CORL~2: 0.786687

RES: 0.367145 SEE: 0.038700

Statistical indices of Training calculation:

ITERATION (B): 24

No. Sequence Expt. Act Predicted Act Difference

1 VALVGLFVL 5.14800 5.73834 -0.59034

2 GTLVALVGL 5.34200 5.92159 -0.57959

3 LQTTIHDII 5.50100 5.78712 -0.28612

4 SLHVGTQCA 5.84200 6.12887 -0.28687

5 ALPYWNFAT 5.86900 6.65348 -0.78448

6 SLNFMGYVI 5.88100 5.97365 -0.09265

7 NLQSLTNLL 6.00000 6.69586 -0.69586

8 FVTWHRYHL 6.02500 5.73345 0.29155

9 DPKVKQWPL 6.17600 5.74027 0.43573

10 ITSQVPFSV 6.19600 6.56836 -0.37236

11 ALAKAAAAI 6.21100 6.23382 -0.02282

12 GLGQVPLIV 6.30100 6.56356 -0.26256

13 MLDLQPETT 6.33500 6.84985 -0.51485

14 LLSSNLSWL 6.34200 6.34974 -0.00774

15 GLACHQLCA 6.38000 6.02983 0.35017

16 LIGNESFAL 6.41500 7.05166 -0.63666

17 ALAKAAAAV 6.41900 6.46720 -0.04820

18 LLAVGATKV 6.47700 6.50401 -0.02701

19 ALAKAAAAL 6.51100 6.21627 0.29473

20 WILRGTSFV 6.55600 6.90677 -0.35077

21 IISCTCPTV 6.58000 6.66097 -0.08097

22 FLGGTPVCL 6.62300 6.88049 -0.25749

23 ALIHHNTHL 6.62300 6.78994 -0.16694

24 NLSWLSLDV 6.63900 6.04715 0.59185

25 YMIMVKCWM 6.66300 6.69102 -0.02802

26 VLQAGFFLL 6.68200 7.04899 -0.36699

27 GTLGIVCPI 6.71400 6.50048 0.21352

28 VILGVLLLI 6.78500 7.48048 -0.69548

29 VTWHRYHLL 6.79300 6.55453 0.23847

30 PLLPIFFCL 6.79600 7.53403 -0.73803

31 TLGIVCPIC 6.81500 5.96438 0.85062

32 CLTSTVQLV 6.83200 7.10735 -0.27535

33 ILLLCLIFL 6.84500 6.81262 0.03238

34 FAFRDLCIV 6.88600 6.65138 0.23462

35 FLEPGPVTA 6.89800 7.47449 -0.57649

36 ALAKAAAAA 6.94700 6.77712 0.16988

37 LMAVVLASL 6.95400 7.51325 -0.55925

38 YVITTQHWL 6.98300 6.33632 0.64668

39 LLCLIFLLV 6.99600 7.52677 -0.53077

40 ITAQVPFSV 7.02000 6.65076 0.36924

41 YLEPGPVTL 7.05800 7.15306 -0.09506

42 YTDQVPFSV 7.06600 7.06208 0.00392

43 NLYVSLLLL 7.11400 6.99426 0.11974

44 ILHNGAYSL 7.12700 7.35376 -0.22676

45 SIISAVVGI 7.15900 7.29851 -0.13951

46 VVMGTLVAL 7.17400 7.30914 -0.13514

47 YLEPGPVTI 7.18700 7.17061 0.01639

48 GLSRYVARL 7.24800 7.46171 -0.21371

49 LLAQFTSAI 7.30100 7.43616 -0.13516

50 VLLDYQGML 7.32800 7.59784 -0.26984

51 YLEPGPVTV 7.34200 7.40399 -0.06199

52 ILSPFMPLL 7.34700 7.14843 0.19857

53 YLSPGPVTA 7.38300 7.54176 -0.15876

54 IIDQVPFSV 7.39800 7.65280 -0.25480

55 SVYDFFVWL 7.44400 7.36886 0.07514

56 ITWQVPFSV 7.46300 7.43348 0.02952

57 ITYQVPFSV 7.48000 7.65534 -0.17534

58 GLYSSTVPV 7.48100 7.61953 -0.13853

59 VMGTLVALV 7.55300 7.24020 0.31280

60 LLLCLIFLL 7.58500 7.16042 0.42458

61 SLDDYNHLV 7.58500 7.16387 0.42113

62 VLIQRNPQL 7.64400 6.95227 0.69173

63 SLYADSPSV 7.65800 7.70200 -0.04400

64 ILSQVPFSV 7.69900 7.65000 0.04900

65 IMDQVPFSV 7.71900 8.04752 -0.32852

66 QLFEDNYAL 7.76400 7.47882 0.28518

67 ALMDKSLHV 7.77000 7.52171 0.24829

68 YAIDLPVSV 7.79600 7.58578 0.21022

69 FVWLHYYSV 7.82400 8.12898 -0.30498

70 MLGTHTMEV 7.84500 7.31371 0.53129

71 LLFGYPVYV 7.88600 8.04248 -0.15648

72 ILKEPVHGV 7.92100 7.59251 0.32849

73 YLMPGPVTV 7.93200 7.91820 0.01380

74 WLDQVPFSV 7.93900 7.96010 -0.02110

75 KTWGQYWQV 7.95500 7.68308 0.27192

76 ALMPLYACI 8.00000 7.44461 0.55539

77 YLAPGPVTA 8.03200 7.62416 0.40784

78 YLYPGPVTV 8.05100 8.31881 -0.26781

79 LLMGTLGIV 8.09700 7.68210 0.41490

80 YLWPGPVTV 8.12500 8.09696 0.02804

81 FLLTRILTI 8.14900 7.88879 0.26021

82 GLLGWSPQA 8.23700 8.20117 0.03583

83 ILYQVPFSV 8.31000 8.73698 -0.42698

84 GILTVILGV 8.34700 7.83212 0.51488

85 NMVPFFPPV 8.39800 8.09108 0.30692

86 ILDQVPFSV 8.48100 7.69400 0.78700

87 YLFPGPVTA 8.49500 8.33947 0.15553

88 YLDQVPFSV 8.63800 8.14372 0.49428

89 ILFQVPFSV 8.69900 8.44771 0.25129

90 ILWQVPFSV 8.77000 8.51512 0.25488

Correlation Coeffecient and Stand Error

CORL: 0.886980 CORL~2: 0.786733

RES: 0.367119 SEE: 0.038698

Statistical indices of Training calculation:

ITERATION (A): 25

No. Sequence Expt. Act Predicted Act Difference

1 VALVGLFVL 5.14800 5.74679 -0.59879

2 GTLVALVGL 5.34200 5.92862 -0.58662

3 LQTTIHDII 5.50100 5.79588 -0.29488

4 SLHVGTQCA 5.84200 6.13600 -0.29400

5 ALPYWNFAT 5.86900 6.65663 -0.78763

6 SLNFMGYVI 5.88100 5.98096 -0.09996

7 NLQSLTNLL 6.00000 6.69896 -0.69896

8 FVTWHRYHL 6.02500 5.74212 0.28288

9 DPKVKQWPL 6.17600 5.74777 0.42823

10 ITSQVPFSV 6.19600 6.57124 -0.37524

11 ALAKAAAAI 6.21100 6.23967 -0.02867

12 GLGQVPLIV 6.30100 6.56692 -0.26592

13 MLDLQPETT 6.33500 6.85224 -0.51724

14 LLSSNLSWL 6.34200 6.35503 -0.01303

15 GLACHQLCA 6.38000 6.03836 0.34164

16 LIGNESFAL 6.41500 7.05319 -0.63819

17 ALAKAAAAV 6.41900 6.47179 -0.05279

18 LLAVGATKV 6.47700 6.50831 -0.03131

19 ALAKAAAAL 6.51100 6.22231 0.28869

20 WILRGTSFV 6.55600 6.90879 -0.35279

21 IISCTCPTV 6.58000 6.66492 -0.08492

22 FLGGTPVCL 6.62300 6.88253 -0.25953

23 ALIHHNTHL 6.62300 6.79250 -0.16950

24 NLSWLSLDV 6.63900 6.05424 0.58476

25 YMIMVKCWM 6.66300 6.69407 -0.03107

26 VLQAGFFLL 6.68200 7.05037 -0.36837

27 GTLGIVCPI 6.71400 6.50473 0.20927

28 VILGVLLLI 6.78500 7.47938 -0.69438

29 VTWHRYHLL 6.79300 6.55804 0.23496

30 PLLPIFFCL 6.79600 7.53344 -0.73744

31 TLGIVCPIC 6.81500 5.97143 0.84357

32 CLTSTVQLV 6.83200 7.10736 -0.27536

33 ILLLCLIFL 6.84500 6.81488 0.03012

34 FAFRDLCIV 6.88600 6.65414 0.23186

35 FLEPGPVTA 6.89800 7.47400 -0.57600

36 ALAKAAAAA 6.94700 6.78005 0.16695

37 LMAVVLASL 6.95400 7.51120 -0.55720

38 YVITTQHWL 6.98300 6.34198 0.64102

39 LLCLIFLLV 6.99600 7.52477 -0.52877

40 ITAQVPFSV 7.02000 6.65302 0.36698

41 YLEPGPVTL 7.05800 7.15435 -0.09635

42 YTDQVPFSV 7.06600 7.06205 0.00395

43 NLYVSLLLL 7.11400 6.99587 0.11813

44 ILHNGAYSL 7.12700 7.35319 -0.22619

45 SIISAVVGI 7.15900 7.29838 -0.13938

46 VVMGTLVAL 7.17400 7.30867 -0.13467

47 YLEPGPVTI 7.18700 7.17171 0.01529

48 GLSRYVARL 7.24800 7.46038 -0.21238

49 LLAQFTSAI 7.30100 7.43476 -0.13376

50 VLLDYQGML 7.32800 7.59608 -0.26808

51 YLEPGPVTV 7.34200 7.40383 -0.06183

52 ILSPFMPLL 7.34700 7.14968 0.19732

53 YLSPGPVTA 7.38300 7.54099 -0.15799

54 IIDQVPFSV 7.39800 7.65022 -0.25222

55 SVYDFFVWL 7.44400 7.36818 0.07582

56 ITWQVPFSV 7.46300 7.43130 0.03170

57 ITYQVPFSV 7.48000 7.65192 -0.17192

58 GLYSSTVPV 7.48100 7.61792 -0.13692

59 VMGTLVALV 7.55300 7.24020 0.31280

60 LLLCLIFLL 7.58500 7.16182 0.42318

61 SLDDYNHLV 7.58500 7.16449 0.42051

62 VLIQRNPQL 7.64400 6.95380 0.69020

63 SLYADSPSV 7.65800 7.69946 -0.04146

64 ILSQVPFSV 7.69900 7.64724 0.05176

65 IMDQVPFSV 7.71900 8.04205 -0.32305

66 QLFEDNYAL 7.76400 7.47776 0.28624

67 ALMDKSLHV 7.77000 7.51970 0.25030

68 YAIDLPVSV 7.79600 7.58315 0.21285

69 FVWLHYYSV 7.82400 8.12393 -0.29993

70 MLGTHTMEV 7.84500 7.31343 0.53157

71 LLFGYPVYV 7.88600 8.03749 -0.15149

72 ILKEPVHGV 7.92100 7.58993 0.33107

73 YLMPGPVTV 7.93200 7.91493 0.01707

74 WLDQVPFSV 7.93900 7.95545 -0.01645

75 KTWGQYWQV 7.95500 7.67989 0.27511

76 ALMPLYACI 8.00000 7.44465 0.55535

77 YLAPGPVTA 8.03200 7.62277 0.40923

78 YLYPGPVTV 8.05100 8.31341 -0.26241

79 LLMGTLGIV 8.09700 7.67924 0.41776

80 YLWPGPVTV 8.12500 8.09278 0.03222

81 FLLTRILTI 8.14900 7.88575 0.26325

82 GLLGWSPQA 8.23700 8.19559 0.04141

83 ILYQVPFSV 8.31000 8.72792 -0.41792

84 GILTVILGV 8.34700 7.82945 0.51755

85 NMVPFFPPV 8.39800 8.08709 0.31091

86 ILDQVPFSV 8.48100 7.69097 0.79003

87 YLFPGPVTA 8.49500 8.33400 0.16100

88 YLDQVPFSV 8.63800 8.13805 0.49995

89 ILFQVPFSV 8.69900 8.44024 0.25876

90 ILWQVPFSV 8.77000 8.50729 0.26271

Correlation Coeffecient and Stand Error

CORL: 0.886986 CORL~2: 0.786744

RES: 0.367096 SEE: 0.038695

Statistical indices of Training calculation:

ITERATION (B): 25

No. Sequence Expt. Act Predicted Act Difference

1 VALVGLFVL 5.14800 5.73987 -0.59187

2 GTLVALVGL 5.34200 5.92304 -0.58104

3 LQTTIHDII 5.50100 5.78972 -0.28872

4 SLHVGTQCA 5.84200 6.13160 -0.28960

5 ALPYWNFAT 5.86900 6.65238 -0.78338

6 SLNFMGYVI 5.88100 5.97205 -0.09105

7 NLQSLTNLL 6.00000 6.69618 -0.69618

8 FVTWHRYHL 6.02500 5.73247 0.29253

9 DPKVKQWPL 6.17600 5.74025 0.43575

10 ITSQVPFSV 6.19600 6.57027 -0.37427

11 ALAKAAAAI 6.21100 6.23469 -0.02369

12 GLGQVPLIV 6.30100 6.56369 -0.26269

13 MLDLQPETT 6.33500 6.85051 -0.51551

14 LLSSNLSWL 6.34200 6.34979 -0.00779

15 GLACHQLCA 6.38000 6.03258 0.34742

16 LIGNESFAL 6.41500 7.05208 -0.63708

17 ALAKAAAAV 6.41900 6.46892 -0.04992

18 LLAVGATKV 6.47700 6.50473 -0.02773

19 ALAKAAAAL 6.51100 6.21717 0.29383

20 WILRGTSFV 6.55600 6.90692 -0.35092

21 IISCTCPTV 6.58000 6.66133 -0.08133

22 FLGGTPVCL 6.62300 6.87998 -0.25698

23 ALIHHNTHL 6.62300 6.79000 -0.16700

24 NLSWLSLDV 6.63900 6.04715 0.59185

25 YMIMVKCWM 6.66300 6.68645 -0.02345

26 VLQAGFFLL 6.68200 7.04824 -0.36624

27 GTLGIVCPI 6.71400 6.50268 0.21132

28 VILGVLLLI 6.78500 7.47977 -0.69477

29 VTWHRYHLL 6.79300 6.55501 0.23799

30 PLLPIFFCL 6.79600 7.53289 -0.73689

31 TLGIVCPIC 6.81500 5.96300 0.85200

32 CLTSTVQLV 6.83200 7.10724 -0.27524

33 ILLLCLIFL 6.84500 6.80976 0.03524

34 FAFRDLCIV 6.88600 6.65301 0.23299

35 FLEPGPVTA 6.89800 7.47634 -0.57834

36 ALAKAAAAA 6.94700 6.78000 0.16700

37 LMAVVLASL 6.95400 7.51113 -0.55713

38 YVITTQHWL 6.98300 6.33673 0.64627

39 LLCLIFLLV 6.99600 7.52443 -0.52843

40 ITAQVPFSV 7.02000 6.65242 0.36758

41 YLEPGPVTL 7.05800 7.15263 -0.09463

42 YTDQVPFSV 7.06600 7.06321 0.00279

43 NLYVSLLLL 7.11400 6.99264 0.12136

44 ILHNGAYSL 7.12700 7.35336 -0.22636

45 SIISAVVGI 7.15900 7.29909 -0.14009

46 VVMGTLVAL 7.17400 7.30979 -0.13579

47 YLEPGPVTI 7.18700 7.17015 0.01685

48 GLSRYVARL 7.24800 7.46171 -0.21371

49 LLAQFTSAI 7.30100 7.43562 -0.13462

50 VLLDYQGML 7.32800 7.59721 -0.26921

51 YLEPGPVTV 7.34200 7.40438 -0.06238

52 ILSPFMPLL 7.34700 7.14767 0.19933

53 YLSPGPVTA 7.38300 7.54358 -0.16058

54 IIDQVPFSV 7.39800 7.65279 -0.25479

55 SVYDFFVWL 7.44400 7.36847 0.07553

56 ITWQVPFSV 7.46300 7.43424 0.02876

57 ITYQVPFSV 7.48000 7.65587 -0.17587

58 GLYSSTVPV 7.48100 7.62056 -0.13956

59 VMGTLVALV 7.55300 7.23986 0.31314

60 LLLCLIFLL 7.58500 7.15858 0.42642

61 SLDDYNHLV 7.58500 7.16506 0.41994

62 VLIQRNPQL 7.64400 6.95178 0.69222

63 SLYADSPSV 7.65800 7.70280 -0.04480

64 ILSQVPFSV 7.69900 7.64974 0.04926

65 IMDQVPFSV 7.71900 8.04589 -0.32689

66 QLFEDNYAL 7.76400 7.47810 0.28590

67 ALMDKSLHV 7.77000 7.52203 0.24797

68 YAIDLPVSV 7.79600 7.58782 0.20818

69 FVWLHYYSV 7.82400 8.12766 -0.30366

70 MLGTHTMEV 7.84500 7.31412 0.53088

71 LLFGYPVYV 7.88600 8.04088 -0.15488

72 ILKEPVHGV 7.92100 7.59240 0.32860

73 YLMPGPVTV 7.93200 7.91782 0.01418

74 WLDQVPFSV 7.93900 7.95929 -0.02029

75 KTWGQYWQV 7.95500 7.68408 0.27092

76 ALMPLYACI 8.00000 7.44407 0.55593

77 YLAPGPVTA 8.03200 7.62573 0.40627

78 YLYPGPVTV 8.05100 8.31810 -0.26710

79 LLMGTLGIV 8.09700 7.68164 0.41536

80 YLWPGPVTV 8.12500 8.09647 0.02853

81 FLLTRILTI 8.14900 7.88796 0.26104

82 GLLGWSPQA 8.23700 8.20280 0.03420

83 ILYQVPFSV 8.31000 8.73534 -0.42534

84 GILTVILGV 8.34700 7.83303 0.51397

85 NMVPFFPPV 8.39800 8.09057 0.30743

86 ILDQVPFSV 8.48100 7.69367 0.78733

87 YLFPGPVTA 8.49500 8.34020 0.15480

88 YLDQVPFSV 8.63800 8.14268 0.49532

89 ILFQVPFSV 8.69900 8.44636 0.25264

90 ILWQVPFSV 8.77000 8.51371 0.25629

Correlation Coeffecient and Stand Error

CORL: 0.887006 CORL~2: 0.786780

RES: 0.367075 SEE: 0.038693

Statistical indices of Training calculation:

ITERATION (A): 26

No. Sequence Expt. Act Predicted Act Difference

1 VALVGLFVL 5.14800 5.74749 -0.59949

2 GTLVALVGL 5.34200 5.92938 -0.58738

3 LQTTIHDII 5.50100 5.79760 -0.29660

4 SLHVGTQCA 5.84200 6.13801 -0.29601

5 ALPYWNFAT 5.86900 6.65523 -0.78623

6 SLNFMGYVI 5.88100 5.97865 -0.09765

7 NLQSLTNLL 6.00000 6.69897 -0.69897

8 FVTWHRYHL 6.02500 5.74030 0.28470

9 DPKVKQWPL 6.17600 5.74703 0.42897

10 ITSQVPFSV 6.19600 6.57287 -0.37687

11 ALAKAAAAI 6.21100 6.23996 -0.02896

12 GLGQVPLIV 6.30100 6.56671 -0.26571

13 MLDLQPETT 6.33500 6.85266 -0.51766

14 LLSSNLSWL 6.34200 6.35456 -0.01256

15 GLACHQLCA 6.38000 6.04025 0.33975

16 LIGNESFAL 6.41500 7.05345 -0.63845

17 ALAKAAAAV 6.41900 6.47305 -0.05405

18 LLAVGATKV 6.47700 6.50859 -0.03159

19 ALAKAAAAL 6.51100 6.22261 0.28839

20 WILRGTSFV 6.55600 6.90874 -0.35274

21 IISCTCPTV 6.58000 6.66488 -0.08488

22 FLGGTPVCL 6.62300 6.88183 -0.25883

23 ALIHHNTHL 6.62300 6.79231 -0.16931

24 NLSWLSLDV 6.63900 6.05354 0.58546

25 YMIMVKCWM 6.66300 6.68923 -0.02623

26 VLQAGFFLL 6.68200 7.04949 -0.36749

27 GTLGIVCPI 6.71400 6.50649 0.20751

28 VILGVLLLI 6.78500 7.47878 -0.69378

29 VTWHRYHLL 6.79300 6.55817 0.23483

30 PLLPIFFCL 6.79600 7.53235 -0.73635

31 TLGIVCPIC 6.81500 5.96936 0.84564

32 CLTSTVQLV 6.83200 7.10725 -0.27525

33 ILLLCLIFL 6.84500 6.81180 0.03320

34 FAFRDLCIV 6.88600 6.65550 0.23050

35 FLEPGPVTA 6.89800 7.47587 -0.57787

36 ALAKAAAAA 6.94700 6.78263 0.16437

37 LMAVVLASL 6.95400 7.50929 -0.55529

38 YVITTQHWL 6.98300 6.34184 0.64116

39 LLCLIFLLV 6.99600 7.52264 -0.52664

40 ITAQVPFSV 7.02000 6.65445 0.36555

41 YLEPGPVTL 7.05800 7.15379 -0.09579

42 YTDQVPFSV 7.06600 7.06319 0.00281

43 NLYVSLLLL 7.11400 6.99409 0.11991

44 ILHNGAYSL 7.12700 7.35284 -0.22584

45 SIISAVVGI 7.15900 7.29898 -0.13998

46 VVMGTLVAL 7.17400 7.30937 -0.13537

47 YLEPGPVTI 7.18700 7.17113 0.01587

48 GLSRYVARL 7.24800 7.46051 -0.21251

49 LLAQFTSAI 7.30100 7.43437 -0.13337

50 VLLDYQGML 7.32800 7.59563 -0.26763

51 YLEPGPVTV 7.34200 7.40423 -0.06223

52 ILSPFMPLL 7.34700 7.14879 0.19821

53 YLSPGPVTA 7.38300 7.54287 -0.15987

54 IIDQVPFSV 7.39800 7.65047 -0.25247

55 SVYDFFVWL 7.44400 7.36788 0.07612

56 ITWQVPFSV 7.46300 7.43228 0.03072

57 ITYQVPFSV 7.48000 7.65280 -0.17280

58 GLYSSTVPV 7.48100 7.61909 -0.13809

59 VMGTLVALV 7.55300 7.23986 0.31314

60 LLLCLIFLL 7.58500 7.15984 0.42516

61 SLDDYNHLV 7.58500 7.16561 0.41939

62 VLIQRNPQL 7.64400 6.95317 0.69083

63 SLYADSPSV 7.65800 7.70051 -0.04251

64 ILSQVPFSV 7.69900 7.64725 0.05175

65 IMDQVPFSV 7.71900 8.04097 -0.32197

66 QLFEDNYAL 7.76400 7.47714 0.28686

67 ALMDKSLHV 7.77000 7.52021 0.24979

68 YAIDLPVSV 7.79600 7.58545 0.21055

69 FVWLHYYSV 7.82400 8.12312 -0.29912

70 MLGTHTMEV 7.84500 7.31386 0.53114

71 LLFGYPVYV 7.88600 8.03638 -0.15038

72 ILKEPVHGV 7.92100 7.59008 0.33092

73 YLMPGPVTV 7.93200 7.91487 0.01713

74 WLDQVPFSV 7.93900 7.95510 -0.01610

75 KTWGQYWQV 7.95500 7.68120 0.27380

76 ALMPLYACI 8.00000 7.44409 0.55591

77 YLAPGPVTA 8.03200 7.62445 0.40755

78 YLYPGPVTV 8.05100 8.31322 -0.26222

79 LLMGTLGIV 8.09700 7.67906 0.41794

80 YLWPGPVTV 8.12500 8.09271 0.03229

81 FLLTRILTI 8.14900 7.88522 0.26378

82 GLLGWSPQA 8.23700 8.19776 0.03924

83 ILYQVPFSV 8.31000 8.72718 -0.41718

84 GILTVILGV 8.34700 7.83060 0.51640

85 NMVPFFPPV 8.39800 8.08696 0.31104

86 ILDQVPFSV 8.48100 7.69095 0.79005

87 YLFPGPVTA 8.49500 8.33525 0.15975

88 YLDQVPFSV 8.63800 8.13757 0.50043

89 ILFQVPFSV 8.69900 8.43963 0.25937

90 ILWQVPFSV 8.77000 8.50666 0.26334

Correlation Coeffecient and Stand Error

CORL: 0.887012 CORL~2: 0.786790

RES: 0.367056 SEE: 0.038691

Statistical indices of Training calculation:

ITERATION (B): 26

No. Sequence Expt. Act Predicted Act Difference

1 VALVGLFVL 5.14800 5.74124 -0.59324

2 GTLVALVGL 5.34200 5.92434 -0.58234

3 LQTTIHDII 5.50100 5.79204 -0.29104

4 SLHVGTQCA 5.84200 6.13405 -0.29205

5 ALPYWNFAT 5.86900 6.65139 -0.78239

6 SLNFMGYVI 5.88100 5.97061 -0.08961

7 NLQSLTNLL 6.00000 6.69646 -0.69646

8 FVTWHRYHL 6.02500 5.73159 0.29341

9 DPKVKQWPL 6.17600 5.74025 0.43575

10 ITSQVPFSV 6.19600 6.57198 -0.37598

11 ALAKAAAAI 6.21100 6.23547 -0.02447

12 GLGQVPLIV 6.30100 6.56380 -0.26280

13 MLDLQPETT 6.33500 6.85110 -0.51610

14 LLSSNLSWL 6.34200 6.34984 -0.00784

15 GLACHQLCA 6.38000 6.03505 0.34495

16 LIGNESFAL 6.41500 7.05244 -0.63744

17 ALAKAAAAV 6.41900 6.47047 -0.05147

18 LLAVGATKV 6.47700 6.50538 -0.02838

19 ALAKAAAAL 6.51100 6.21798 0.29302

20 WILRGTSFV 6.55600 6.90705 -0.35105

21 IISCTCPTV 6.58000 6.66165 -0.08165

22 FLGGTPVCL 6.62300 6.87953 -0.25653

23 ALIHHNTHL 6.62300 6.79006 -0.16706

24 NLSWLSLDV 6.63900 6.04715 0.59185

25 YMIMVKCWM 6.66300 6.68236 -0.01936

26 VLQAGFFLL 6.68200 7.04757 -0.36557

27 GTLGIVCPI 6.71400 6.50464 0.20936

28 VILGVLLLI 6.78500 7.47912 -0.69412

29 VTWHRYHLL 6.79300 6.55544 0.23756

30 PLLPIFFCL 6.79600 7.53186 -0.73586

31 TLGIVCPIC 6.81500 5.96176 0.85324

32 CLTSTVQLV 6.83200 7.10714 -0.27514

33 ILLLCLIFL 6.84500 6.80718 0.03782

34 FAFRDLCIV 6.88600 6.65447 0.23153

35 FLEPGPVTA 6.89800 7.47799 -0.57999

36 ALAKAAAAA 6.94700 6.78259 0.16441

37 LMAVVLASL 6.95400 7.50923 -0.55523

38 YVITTQHWL 6.98300 6.33710 0.64590

39 LLCLIFLLV 6.99600 7.52233 -0.52633

40 ITAQVPFSV 7.02000 6.65390 0.36610

41 YLEPGPVTL 7.05800 7.15224 -0.09424

42 YTDQVPFSV 7.06600 7.06422 0.00178

43 NLYVSLLLL 7.11400 6.99118 0.12282

44 ILHNGAYSL 7.12700 7.35299 -0.22599

45 SIISAVVGI 7.15900 7.29962 -0.14062

46 VVMGTLVAL 7.17400 7.31038 -0.13638

47 YLEPGPVTI 7.18700 7.16972 0.01728

48 GLSRYVARL 7.24800 7.46171 -0.21371

49 LLAQFTSAI 7.30100 7.43514 -0.13414

50 VLLDYQGML 7.32800 7.59665 -0.26865

51 YLEPGPVTV 7.34200 7.40473 -0.06273

52 ILSPFMPLL 7.34700 7.14698 0.20002

53 YLSPGPVTA 7.38300 7.54521 -0.16221

54 IIDQVPFSV 7.39800 7.65278 -0.25478

55 SVYDFFVWL 7.44400 7.36814 0.07586

56 ITWQVPFSV 7.46300 7.43493 0.02807

57 ITYQVPFSV 7.48000 7.65635 -0.17635

58 GLYSSTVPV 7.48100 7.62149 -0.14049

59 VMGTLVALV 7.55300 7.23956 0.31344

60 LLLCLIFLL 7.58500 7.15692 0.42808

61 SLDDYNHLV 7.58500 7.16612 0.41888

62 VLIQRNPQL 7.64400 6.95135 0.69265

63 SLYADSPSV 7.65800 7.70352 -0.04552

64 ILSQVPFSV 7.69900 7.64950 0.04950

65 IMDQVPFSV 7.71900 8.04442 -0.32542

66 QLFEDNYAL 7.76400 7.47745 0.28655

67 ALMDKSLHV 7.77000 7.52231 0.24769

68 YAIDLPVSV 7.79600 7.58965 0.20635

69 FVWLHYYSV 7.82400 8.12647 -0.30247

70 MLGTHTMEV 7.84500 7.31449 0.53051

71 LLFGYPVYV 7.88600 8.03943 -0.15343

72 ILKEPVHGV 7.92100 7.59230 0.32870

73 YLMPGPVTV 7.93200 7.91747 0.01453

74 WLDQVPFSV 7.93900 7.95856 -0.01956

75 KTWGQYWQV 7.95500 7.68497 0.27003

76 ALMPLYACI 8.00000 7.44357 0.55643

77 YLAPGPVTA 8.03200 7.62713 0.40487

78 YLYPGPVTV 8.05100 8.31747 -0.26647

79 LLMGTLGIV 8.09700 7.68123 0.41577

80 YLWPGPVTV 8.12500 8.09604 0.02896

81 FLLTRILTI 8.14900 7.88722 0.26178

82 GLLGWSPQA 8.23700 8.20426 0.03274

83 ILYQVPFSV 8.31000 8.73388 -0.42388

84 GILTVILGV 8.34700 7.83383 0.51317

85 NMVPFFPPV 8.39800 8.09010 0.30790

86 ILDQVPFSV 8.48100 7.69338 0.78762

87 YLFPGPVTA 8.49500 8.34085 0.15415

88 YLDQVPFSV 8.63800 8.14174 0.49626

89 ILFQVPFSV 8.69900 8.44514 0.25386

90 ILWQVPFSV 8.77000 8.51245 0.25755

Correlation Coeffecient and Stand Error

CORL: 0.887028 CORL~2: 0.786818

RES: 0.367039 SEE: 0.038689

Statistical indices of Training calculation:

ITERATION (A): 27

No. Sequence Expt. Act Predicted Act Difference

1 VALVGLFVL 5.14800 5.74811 -0.60011

2 GTLVALVGL 5.34200 5.93006 -0.58806

3 LQTTIHDII 5.50100 5.79914 -0.29814

4 SLHVGTQCA 5.84200 6.13981 -0.29781

5 ALPYWNFAT 5.86900 6.65396 -0.78496

6 SLNFMGYVI 5.88100 5.97657 -0.09557

7 NLQSLTNLL 6.00000 6.69898 -0.69898

8 FVTWHRYHL 6.02500 5.73867 0.28633

9 DPKVKQWPL 6.17600 5.74638 0.42962

10 ITSQVPFSV 6.19600 6.57433 -0.37833

11 ALAKAAAAI 6.21100 6.24022 -0.02922

12 GLGQVPLIV 6.30100 6.56653 -0.26553

13 MLDLQPETT 6.33500 6.85305 -0.51805

14 LLSSNLSWL 6.34200 6.35414 -0.01214

15 GLACHQLCA 6.38000 6.04196 0.33804

16 LIGNESFAL 6.41500 7.05368 -0.63868

17 ALAKAAAAV 6.41900 6.47419 -0.05519

18 LLAVGATKV 6.47700 6.50885 -0.03185

19 ALAKAAAAL 6.51100 6.22289 0.28811

20 WILRGTSFV 6.55600 6.90869 -0.35269

21 IISCTCPTV 6.58000 6.66486 -0.08486

22 FLGGTPVCL 6.62300 6.88121 -0.25821

23 ALIHHNTHL 6.62300 6.79215 -0.16915

24 NLSWLSLDV 6.63900 6.05291 0.58609

25 YMIMVKCWM 6.66300 6.68489 -0.02189

26 VLQAGFFLL 6.68200 7.04870 -0.36670

27 GTLGIVCPI 6.71400 6.50807 0.20593

28 VILGVLLLI 6.78500 7.47823 -0.69323

29 VTWHRYHLL 6.79300 6.55830 0.23470

30 PLLPIFFCL 6.79600 7.53138 -0.73538

31 TLGIVCPIC 6.81500 5.96751 0.84749

32 CLTSTVQLV 6.83200 7.10714 -0.27514

33 ILLLCLIFL 6.84500 6.80903 0.03597

34 FAFRDLCIV 6.88600 6.65672 0.22928

35 FLEPGPVTA 6.89800 7.47755 -0.57955

36 ALAKAAAAA 6.94700 6.78495 0.16205

37 LMAVVLASL 6.95400 7.50758 -0.55358

38 YVITTQHWL 6.98300 6.34172 0.64128

39 LLCLIFLLV 6.99600 7.52072 -0.52472

40 ITAQVPFSV 7.02000 6.65574 0.36426

41 YLEPGPVTL 7.05800 7.15328 -0.09528

42 YTDQVPFSV 7.06600 7.06421 0.00179

43 NLYVSLLLL 7.11400 6.99250 0.12150

44 ILHNGAYSL 7.12700 7.35252 -0.22552

45 SIISAVVGI 7.15900 7.29951 -0.14051

46 VVMGTLVAL 7.17400 7.31000 -0.13600

47 YLEPGPVTI 7.18700 7.17061 0.01639

48 GLSRYVARL 7.24800 7.46064 -0.21264

49 LLAQFTSAI 7.30100 7.43400 -0.13300

50 VLLDYQGML 7.32800 7.59522 -0.26722

51 YLEPGPVTV 7.34200 7.40458 -0.06258

52 ILSPFMPLL 7.34700 7.14799 0.19901

53 YLSPGPVTA 7.38300 7.54455 -0.16155

54 IIDQVPFSV 7.39800 7.65069 -0.25269

55 SVYDFFVWL 7.44400 7.36762 0.07638

56 ITWQVPFSV 7.46300 7.43317 0.02983

57 ITYQVPFSV 7.48000 7.65359 -0.17359

58 GLYSSTVPV 7.48100 7.62015 -0.13915

59 VMGTLVALV 7.55300 7.23956 0.31344

60 LLLCLIFLL 7.58500 7.15806 0.42694

61 SLDDYNHLV 7.58500 7.16661 0.41839

62 VLIQRNPQL 7.64400 6.95261 0.69139

63 SLYADSPSV 7.65800 7.70144 -0.04344

64 ILSQVPFSV 7.69900 7.64726 0.05174

65 IMDQVPFSV 7.71900 8.04000 -0.32100

66 QLFEDNYAL 7.76400 7.47658 0.28742

67 ALMDKSLHV 7.77000 7.52067 0.24933

68 YAIDLPVSV 7.79600 7.58750 0.20850

69 FVWLHYYSV 7.82400 8.12239 -0.29839

70 MLGTHTMEV 7.84500 7.31425 0.53075

71 LLFGYPVYV 7.88600 8.03538 -0.14938

72 ILKEPVHGV 7.92100 7.59021 0.33079

73 YLMPGPVTV 7.93200 7.91480 0.01720

74 WLDQVPFSV 7.93900 7.95479 -0.01579

75 KTWGQYWQV 7.95500 7.68236 0.27264

76 ALMPLYACI 8.00000 7.44359 0.55641

77 YLAPGPVTA 8.03200 7.62597 0.40603

78 YLYPGPVTV 8.05100 8.31306 -0.26206

79 LLMGTLGIV 8.09700 7.67889 0.41811

80 YLWPGPVTV 8.12500 8.09263 0.03237

81 FLLTRILTI 8.14900 7.88473 0.26427

82 GLLGWSPQA 8.23700 8.19970 0.03730

83 ILYQVPFSV 8.31000 8.72652 -0.41652

84 GILTVILGV 8.34700 7.83163 0.51537

85 NMVPFFPPV 8.39800 8.08684 0.31116

86 ILDQVPFSV 8.48100 7.69092 0.79008

87 YLFPGPVTA 8.49500 8.33638 0.15862

88 YLDQVPFSV 8.63800 8.13714 0.50086

89 ILFQVPFSV 8.69900 8.43908 0.25992

90 ILWQVPFSV 8.77000 8.50610 0.26390

Correlation Coeffecient and Stand Error

CORL: 0.887033 CORL~2: 0.786827

RES: 0.367024 SEE: 0.038688

Statistical indices of Training calculation:

ITERATION (B): 27

No. Sequence Expt. Act Predicted Act Difference

1 VALVGLFVL 5.14800 5.74246 -0.59446

2 GTLVALVGL 5.34200 5.92550 -0.58350

3 LQTTIHDII 5.50100 5.79411 -0.29311

4 SLHVGTQCA 5.84200 6.13624 -0.29424

5 ALPYWNFAT 5.86900 6.65049 -0.78149

6 SLNFMGYVI 5.88100 5.96931 -0.08831

7 NLQSLTNLL 6.00000 6.69672 -0.69672

8 FVTWHRYHL 6.02500 5.73080 0.29420

9 DPKVKQWPL 6.17600 5.74025 0.43575

10 ITSQVPFSV 6.19600 6.57351 -0.37751

11 ALAKAAAAI 6.21100 6.23617 -0.02517

12 GLGQVPLIV 6.30100 6.56390 -0.26290

13 MLDLQPETT 6.33500 6.85164 -0.51664

14 LLSSNLSWL 6.34200 6.34988 -0.00788

15 GLACHQLCA 6.38000 6.03727 0.34273

16 LIGNESFAL 6.41500 7.05277 -0.63777

17 ALAKAAAAV 6.41900 6.47186 -0.05286

18 LLAVGATKV 6.47700 6.50595 -0.02895

19 ALAKAAAAL 6.51100 6.21871 0.29229

20 WILRGTSFV 6.55600 6.90717 -0.35117

21 IISCTCPTV 6.58000 6.66195 -0.08195

22 FLGGTPVCL 6.62300 6.87914 -0.25614

23 ALIHHNTHL 6.62300 6.79012 -0.16712

24 NLSWLSLDV 6.63900 6.04714 0.59186

25 YMIMVKCWM 6.66300 6.67869 -0.01569

26 VLQAGFFLL 6.68200 7.04697 -0.36497

27 GTLGIVCPI 6.71400 6.50639 0.20761

28 VILGVLLLI 6.78500 7.47855 -0.69355

29 VTWHRYHLL 6.79300 6.55582 0.23718

30 PLLPIFFCL 6.79600 7.53093 -0.73493

31 TLGIVCPIC 6.81500 5.96065 0.85435

32 CLTSTVQLV 6.83200 7.10704 -0.27504

33 ILLLCLIFL 6.84500 6.80486 0.04014

34 FAFRDLCIV 6.88600 6.65579 0.23021

35 FLEPGPVTA 6.89800 7.47947 -0.58147

36 ALAKAAAAA 6.94700 6.78492 0.16208

37 LMAVVLASL 6.95400 7.50753 -0.55353

38 YVITTQHWL 6.98300 6.33744 0.64556

39 LLCLIFLLV 6.99600 7.52043 -0.52443

40 ITAQVPFSV 7.02000 6.65523 0.36477

41 YLEPGPVTL 7.05800 7.15189 -0.09389

42 YTDQVPFSV 7.06600 7.06513 0.00087

43 NLYVSLLLL 7.11400 6.98987 0.12413

44 ILHNGAYSL 7.12700 7.35266 -0.22566

45 SIISAVVGI 7.15900 7.30010 -0.14110

46 VVMGTLVAL 7.17400 7.31091 -0.13691

47 YLEPGPVTI 7.18700 7.16934 0.01766

48 GLSRYVARL 7.24800 7.46172 -0.21372

49 LLAQFTSAI 7.30100 7.43470 -0.13370

50 VLLDYQGML 7.32800 7.59614 -0.26814

51 YLEPGPVTV 7.34200 7.40504 -0.06304

52 ILSPFMPLL 7.34700 7.14636 0.20064

53 YLSPGPVTA 7.38300 7.54667 -0.16367

54 IIDQVPFSV 7.39800 7.65278 -0.25478

55 SVYDFFVWL 7.44400 7.36785 0.07615

56 ITWQVPFSV 7.46300 7.43555 0.02745

57 ITYQVPFSV 7.48000 7.65679 -0.17679

58 GLYSSTVPV 7.48100 7.62232 -0.14132

59 VMGTLVALV 7.55300 7.23929 0.31371

60 LLLCLIFLL 7.58500 7.15543 0.42957

61 SLDDYNHLV 7.58500 7.16707 0.41793

62 VLIQRNPQL 7.64400 6.95097 0.69303

63 SLYADSPSV 7.65800 7.70417 -0.04617

64 ILSQVPFSV 7.69900 7.64929 0.04971

65 IMDQVPFSV 7.71900 8.04312 -0.32412

66 QLFEDNYAL 7.76400 7.47687 0.28713

67 ALMDKSLHV 7.77000 7.52256 0.24744

68 YAIDLPVSV 7.79600 7.59129 0.20471

69 FVWLHYYSV 7.82400 8.12541 -0.30141

70 MLGTHTMEV 7.84500 7.31482 0.53018

71 LLFGYPVYV 7.88600 8.03814 -0.15214

72 ILKEPVHGV 7.92100 7.59221 0.32879

73 YLMPGPVTV 7.93200 7.91716 0.01484

74 WLDQVPFSV 7.93900 7.95791 -0.01891

75 KTWGQYWQV 7.95500 7.68576 0.26924

76 ALMPLYACI 8.00000 7.44312 0.55688

77 YLAPGPVTA 8.03200 7.62839 0.40361

78 YLYPGPVTV 8.05100 8.31689 -0.26589

79 LLMGTLGIV 8.09700 7.68085 0.41615

80 YLWPGPVTV 8.12500 8.09565 0.02935

81 FLLTRILTI 8.14900 7.88654 0.26246

82 GLLGWSPQA 8.23700 8.20557 0.03143

83 ILYQVPFSV 8.31000 8.73256 -0.42256

84 GILTVILGV 8.34700 7.83455 0.51245

85 NMVPFFPPV 8.39800 8.08968 0.30832

86 ILDQVPFSV 8.48100 7.69311 0.78789

87 YLFPGPVTA 8.49500 8.34144 0.15356

88 YLDQVPFSV 8.63800 8.14090 0.49710

89 ILFQVPFSV 8.69900 8.44406 0.25494

90 ILWQVPFSV 8.77000 8.51132 0.25868

Correlation Coeffecient and Stand Error

CORL: 0.887045 CORL~2: 0.786850

RES: 0.367010 SEE: 0.038686

Statistical indices of Training calculation:

ITERATION (A): 28

No. Sequence Expt. Act Predicted Act Difference

1 VALVGLFVL 5.14800 5.74867 -0.60067

2 GTLVALVGL 5.34200 5.93067 -0.58867

3 LQTTIHDII 5.50100 5.80053 -0.29953

4 SLHVGTQCA 5.84200 6.14145 -0.29945

5 ALPYWNFAT 5.86900 6.65283 -0.78383

6 SLNFMGYVI 5.88100 5.97470 -0.09370

7 NLQSLTNLL 6.00000 6.69899 -0.69899

8 FVTWHRYHL 6.02500 5.73722 0.28778

9 DPKVKQWPL 6.17600 5.74581 0.43019

10 ITSQVPFSV 6.19600 6.57564 -0.37964

11 ALAKAAAAI 6.21100 6.24047 -0.02947

12 GLGQVPLIV 6.30100 6.56638 -0.26538

13 MLDLQPETT 6.33500 6.85339 -0.51839

14 LLSSNLSWL 6.34200 6.35377 -0.01177

15 GLACHQLCA 6.38000 6.04351 0.33649

16 LIGNESFAL 6.41500 7.05388 -0.63888

17 ALAKAAAAV 6.41900 6.47522 -0.05622

18 LLAVGATKV 6.47700 6.50909 -0.03209

19 ALAKAAAAL 6.51100 6.22315 0.28785

20 WILRGTSFV 6.55600 6.90865 -0.35265

21 IISCTCPTV 6.58000 6.66485 -0.08485

22 FLGGTPVCL 6.62300 6.88066 -0.25766

23 ALIHHNTHL 6.62300 6.79200 -0.16900

24 NLSWLSLDV 6.63900 6.05234 0.58666

25 YMIMVKCWM 6.66300 6.68100 -0.01800

26 VLQAGFFLL 6.68200 7.04800 -0.36600

27 GTLGIVCPI 6.71400 6.50949 0.20451

28 VILGVLLLI 6.78500 7.47774 -0.69274

29 VTWHRYHLL 6.79300 6.55842 0.23458

30 PLLPIFFCL 6.79600 7.53050 -0.73450

31 TLGIVCPIC 6.81500 5.96587 0.84913

32 CLTSTVQLV 6.83200 7.10705 -0.27505

33 ILLLCLIFL 6.84500 6.80654 0.03846

34 FAFRDLCIV 6.88600 6.65783 0.22817

35 FLEPGPVTA 6.89800 7.47906 -0.58106

36 ALAKAAAAA 6.94700 6.78704 0.15996

37 LMAVVLASL 6.95400 7.50605 -0.55205

38 YVITTQHWL 6.98300 6.34162 0.64138

39 LLCLIFLLV 6.99600 7.51899 -0.52299

40 ITAQVPFSV 7.02000 6.65690 0.36310

41 YLEPGPVTL 7.05800 7.15283 -0.09483

42 YTDQVPFSV 7.06600 7.06513 0.00087

43 NLYVSLLLL 7.11400 6.99107 0.12293

44 ILHNGAYSL 7.12700 7.35224 -0.22524

45 SIISAVVGI 7.15900 7.29999 -0.14099

46 VVMGTLVAL 7.17400 7.31057 -0.13657

47 YLEPGPVTI 7.18700 7.17014 0.01686

48 GLSRYVARL 7.24800 7.46075 -0.21275

49 LLAQFTSAI 7.30100 7.43368 -0.13268

50 VLLDYQGML 7.32800 7.59485 -0.26685

51 YLEPGPVTV 7.34200 7.40489 -0.06289

52 ILSPFMPLL 7.34700 7.14727 0.19973

53 YLSPGPVTA 7.38300 7.54606 -0.16306

54 IIDQVPFSV 7.39800 7.65089 -0.25289

55 SVYDFFVWL 7.44400 7.36739 0.07661

56 ITWQVPFSV 7.46300 7.43396 0.02904

57 ITYQVPFSV 7.48000 7.65430 -0.17430

58 GLYSSTVPV 7.48100 7.62109 -0.14009

59 VMGTLVALV 7.55300 7.23930 0.31370

60 LLLCLIFLL 7.58500 7.15647 0.42853

61 SLDDYNHLV 7.58500 7.16751 0.41749

62 VLIQRNPQL 7.64400 6.95211 0.69189

63 SLYADSPSV 7.65800 7.70228 -0.04428

64 ILSQVPFSV 7.69900 7.64726 0.05174

65 IMDQVPFSV 7.71900 8.03912 -0.32012

66 QLFEDNYAL 7.76400 7.47609 0.28791

67 ALMDKSLHV 7.77000 7.52107 0.24893

68 YAIDLPVSV 7.79600 7.58935 0.20665

69 FVWLHYYSV 7.82400 8.12172 -0.29772

70 MLGTHTMEV 7.84500 7.31460 0.53040

71 LLFGYPVYV 7.88600 8.03448 -0.14848

72 ILKEPVHGV 7.92100 7.59032 0.33068

73 YLMPGPVTV 7.93200 7.91474 0.01726

74 WLDQVPFSV 7.93900 7.95450 -0.01550

75 KTWGQYWQV 7.95500 7.68340 0.27160

76 ALMPLYACI 8.00000 7.44313 0.55687

77 YLAPGPVTA 8.03200 7.62733 0.40467

78 YLYPGPVTV 8.05100 8.31290 -0.26190

79 LLMGTLGIV 8.09700 7.67874 0.41826

80 YLWPGPVTV 8.12500 8.09256 0.03244

81 FLLTRILTI 8.14900 7.88428 0.26472

82 GLLGWSPQA 8.23700 8.20144 0.03556

83 ILYQVPFSV 8.31000 8.72592 -0.41592

84 GILTVILGV 8.34700 7.83255 0.51445

85 NMVPFFPPV 8.39800 8.08673 0.31127

86 ILDQVPFSV 8.48100 7.69089 0.79011

87 YLFPGPVTA 8.49500 8.33739 0.15761

88 YLDQVPFSV 8.63800 8.13675 0.50125

89 ILFQVPFSV 8.69900 8.43859 0.26041

90 ILWQVPFSV 8.77000 8.50558 0.26442

Correlation Coeffecient and Stand Error

CORL: 0.887050 CORL~2: 0.786858

RES: 0.366998 SEE: 0.038685

Statistical indices of Training calculation:

ITERATION (B): 28

No. Sequence Expt. Act Predicted Act Difference

1 VALVGLFVL 5.14800 5.74356 -0.59556

2 GTLVALVGL 5.34200 5.92654 -0.58454

3 LQTTIHDII 5.50100 5.79597 -0.29497

4 SLHVGTQCA 5.84200 6.13822 -0.29622

5 ALPYWNFAT 5.86900 6.64969 -0.78069

6 SLNFMGYVI 5.88100 5.96813 -0.08713

7 NLQSLTNLL 6.00000 6.69695 -0.69695

8 FVTWHRYHL 6.02500 5.73009 0.29491

9 DPKVKQWPL 6.17600 5.74026 0.43574

10 ITSQVPFSV 6.19600 6.57490 -0.37890

11 ALAKAAAAI 6.21100 6.23680 -0.02580

12 GLGQVPLIV 6.30100 6.56400 -0.26300

13 MLDLQPETT 6.33500 6.85212 -0.51712

14 LLSSNLSWL 6.34200 6.34992 -0.00792

15 GLACHQLCA 6.38000 6.03928 0.34072

16 LIGNESFAL 6.41500 7.05307 -0.63807

17 ALAKAAAAV 6.41900 6.47312 -0.05412

18 LLAVGATKV 6.47700 6.50647 -0.02947

19 ALAKAAAAL 6.51100 6.21938 0.29162

20 WILRGTSFV 6.55600 6.90728 -0.35128

21 IISCTCPTV 6.58000 6.66221 -0.08221

22 FLGGTPVCL 6.62300 6.87879 -0.25579

23 ALIHHNTHL 6.62300 6.79017 -0.16717

24 NLSWLSLDV 6.63900 6.04712 0.59188

25 YMIMVKCWM 6.66300 6.67539 -0.01239

26 VLQAGFFLL 6.68200 7.04643 -0.36443

27 GTLGIVCPI 6.71400 6.50797 0.20603

28 VILGVLLLI 6.78500 7.47802 -0.69302

29 VTWHRYHLL 6.79300 6.55617 0.23683

30 PLLPIFFCL 6.79600 7.53010 -0.73410

31 TLGIVCPIC 6.81500 5.95966 0.85534

32 CLTSTVQLV 6.83200 7.10696 -0.27496

33 ILLLCLIFL 6.84500 6.80276 0.04224

34 FAFRDLCIV 6.88600 6.65698 0.22902

35 FLEPGPVTA 6.89800 7.48080 -0.58280

36 ALAKAAAAA 6.94700 6.78701 0.15999

37 LMAVVLASL 6.95400 7.50600 -0.55200

38 YVITTQHWL 6.98300 6.33775 0.64525

39 LLCLIFLLV 6.99600 7.51873 -0.52273

40 ITAQVPFSV 7.02000 6.65643 0.36357

41 YLEPGPVTL 7.05800 7.15157 -0.09357

42 YTDQVPFSV 7.06600 7.06595 0.00005

43 NLYVSLLLL 7.11400 6.98869 0.12531

44 ILHNGAYSL 7.12700 7.35236 -0.22536

45 SIISAVVGI 7.15900 7.30052 -0.14152

46 VVMGTLVAL 7.17400 7.31139 -0.13739

47 YLEPGPVTI 7.18700 7.16900 0.01800

48 GLSRYVARL 7.24800 7.46172 -0.21372

49 LLAQFTSAI 7.30100 7.43430 -0.13330

50 VLLDYQGML 7.32800 7.59569 -0.26769

51 YLEPGPVTV 7.34200 7.40531 -0.06331

52 ILSPFMPLL 7.34700 7.14579 0.20121

53 YLSPGPVTA 7.38300 7.54799 -0.16499

54 IIDQVPFSV 7.39800 7.65277 -0.25477

55 SVYDFFVWL 7.44400 7.36759 0.07641

56 ITWQVPFSV 7.46300 7.43610 0.02690

57 ITYQVPFSV 7.48000 7.65719 -0.17719

58 GLYSSTVPV 7.48100 7.62306 -0.14206

59 VMGTLVALV 7.55300 7.23906 0.31394

60 LLLCLIFLL 7.58500 7.15409 0.43091

61 SLDDYNHLV 7.58500 7.16793 0.41707

62 VLIQRNPQL 7.64400 6.95063 0.69337

63 SLYADSPSV 7.65800 7.70475 -0.04675

64 ILSQVPFSV 7.69900 7.64910 0.04990

65 IMDQVPFSV 7.71900 8.04194 -0.32294

66 QLFEDNYAL 7.76400 7.47635 0.28765

67 ALMDKSLHV 7.77000 7.52279 0.24721

68 YAIDLPVSV 7.79600 7.59277 0.20323

69 FVWLHYYSV 7.82400 8.12445 -0.30045

70 MLGTHTMEV 7.84500 7.31512 0.52988

71 LLFGYPVYV 7.88600 8.03697 -0.15097

72 ILKEPVHGV 7.92100 7.59213 0.32887

73 YLMPGPVTV 7.93200 7.91688 0.01512

74 WLDQVPFSV 7.93900 7.95732 -0.01832

75 KTWGQYWQV 7.95500 7.68647 0.26853

76 ALMPLYACI 8.00000 7.44271 0.55729

77 YLAPGPVTA 8.03200 7.62952 0.40248

78 YLYPGPVTV 8.05100 8.31637 -0.26537

79 LLMGTLGIV 8.09700 7.68051 0.41649

80 YLWPGPVTV 8.12500 8.09529 0.02971

81 FLLTRILTI 8.14900 7.88592 0.26308

82 GLLGWSPQA 8.23700 8.20675 0.03025

83 ILYQVPFSV 8.31000 8.73138 -0.42138

84 GILTVILGV 8.34700 7.83519 0.51181

85 NMVPFFPPV 8.39800 8.08930 0.30870

86 ILDQVPFSV 8.48100 7.69287 0.78813

87 YLFPGPVTA 8.49500 8.34197 0.15303

88 YLDQVPFSV 8.63800 8.14015 0.49785

89 ILFQVPFSV 8.69900 8.44308 0.25592

90 ILWQVPFSV 8.77000 8.51030 0.25970

Correlation Coeffecient and Stand Error

CORL: 0.887060 CORL~2: 0.786875

RES: 0.366987 SEE: 0.038684

Statistical indices of Training calculation:

ITERATION (A): 29

No. Sequence Expt. Act Predicted Act Difference

1 VALVGLFVL 5.14800 5.74918 -0.60118

2 GTLVALVGL 5.34200 5.93122 -0.58922

3 LQTTIHDII 5.50100 5.80177 -0.30077

4 SLHVGTQCA 5.84200 6.14292 -0.30092

5 ALPYWNFAT 5.86900 6.65181 -0.78281

6 SLNFMGYVI 5.88100 5.97301 -0.09201

7 NLQSLTNLL 6.00000 6.69900 -0.69900

8 FVTWHRYHL 6.02500 5.73591 0.28909

9 DPKVKQWPL 6.17600 5.74530 0.43070

10 ITSQVPFSV 6.19600 6.57683 -0.38083

11 ALAKAAAAI 6.21100 6.24069 -0.02969

12 GLGQVPLIV 6.30100 6.56624 -0.26524

13 MLDLQPETT 6.33500 6.85371 -0.51871

14 LLSSNLSWL 6.34200 6.35343 -0.01143

15 GLACHQLCA 6.38000 6.04491 0.33509

16 LIGNESFAL 6.41500 7.05407 -0.63907

17 ALAKAAAAV 6.41900 6.47615 -0.05715

18 LLAVGATKV 6.47700 6.50930 -0.03230

19 ALAKAAAAL 6.51100 6.22340 0.28760

20 WILRGTSFV 6.55600 6.90861 -0.35261

21 IISCTCPTV 6.58000 6.66484 -0.08484

22 FLGGTPVCL 6.62300 6.88018 -0.25718

23 ALIHHNTHL 6.62300 6.79187 -0.16887

24 NLSWLSLDV 6.63900 6.05183 0.58717

25 YMIMVKCWM 6.66300 6.67750 -0.01450

26 VLQAGFFLL 6.68200 7.04736 -0.36536

27 GTLGIVCPI 6.71400 6.51076 0.20324

28 VILGVLLLI 6.78500 7.47729 -0.69229

29 VTWHRYHLL 6.79300 6.55853 0.23447

30 PLLPIFFCL 6.79600 7.52970 -0.73370

31 TLGIVCPIC 6.81500 5.96439 0.85061

32 CLTSTVQLV 6.83200 7.10697 -0.27497

33 ILLLCLIFL 6.84500 6.80429 0.04071

34 FAFRDLCIV 6.88600 6.65882 0.22718

35 FLEPGPVTA 6.89800 7.48041 -0.58241

36 ALAKAAAAA 6.94700 6.78892 0.15808

37 LMAVVLASL 6.95400 7.50466 -0.55066

38 YVITTQHWL 6.98300 6.34154 0.64146

39 LLCLIFLLV 6.99600 7.51743 -0.52143

40 ITAQVPFSV 7.02000 6.65795 0.36205

41 YLEPGPVTL 7.05800 7.15242 -0.09442

42 YTDQVPFSV 7.06600 7.06595 0.00005

43 NLYVSLLLL 7.11400 6.98978 0.12422

44 ILHNGAYSL 7.12700 7.35198 -0.22498

45 SIISAVVGI 7.15900 7.30043 -0.14143

46 VVMGTLVAL 7.17400 7.31108 -0.13708

47 YLEPGPVTI 7.18700 7.16971 0.01729

48 GLSRYVARL 7.24800 7.46086 -0.21286

49 LLAQFTSAI 7.30100 7.43338 -0.13238

50 VLLDYQGML 7.32800 7.59452 -0.26652

51 YLEPGPVTV 7.34200 7.40517 -0.06317

52 ILSPFMPLL 7.34700 7.14662 0.20038

53 YLSPGPVTA 7.38300 7.54743 -0.16443

54 IIDQVPFSV 7.39800 7.65106 -0.25306

55 SVYDFFVWL 7.44400 7.36718 0.07682

56 ITWQVPFSV 7.46300 7.43467 0.02833

57 ITYQVPFSV 7.48000 7.65494 -0.17494

58 GLYSSTVPV 7.48100 7.62194 -0.14094

59 VMGTLVALV 7.55300 7.23907 0.31393

60 LLLCLIFLL 7.58500 7.15503 0.42997

61 SLDDYNHLV 7.58500 7.16832 0.41668

62 VLIQRNPQL 7.64400 6.95166 0.69234

63 SLYADSPSV 7.65800 7.70304 -0.04504

64 ILSQVPFSV 7.69900 7.64727 0.05173

65 IMDQVPFSV 7.71900 8.03834 -0.31934

66 QLFEDNYAL 7.76400 7.47564 0.28836

67 ALMDKSLHV 7.77000 7.52144 0.24856

68 YAIDLPVSV 7.79600 7.59101 0.20499

69 FVWLHYYSV 7.82400 8.12112 -0.29712

70 MLGTHTMEV 7.84500 7.31491 0.53009

71 LLFGYPVYV 7.88600 8.03367 -0.14767

72 ILKEPVHGV 7.92100 7.59043 0.33057

73 YLMPGPVTV 7.93200 7.91468 0.01732

74 WLDQVPFSV 7.93900 7.95424 -0.01524

75 KTWGQYWQV 7.95500 7.68433 0.27067

76 ALMPLYACI 8.00000 7.44271 0.55729

77 YLAPGPVTA 8.03200 7.62855 0.40345

78 YLYPGPVTV 8.05100 8.31276 -0.26176

79 LLMGTLGIV 8.09700 7.67859 0.41841

80 YLWPGPVTV 8.12500 8.09249 0.03251

81 FLLTRILTI 8.14900 7.88388 0.26512

82 GLLGWSPQA 8.23700 8.20300 0.03400

83 ILYQVPFSV 8.31000 8.72537 -0.41537

84 GILTVILGV 8.34700 7.83337 0.51363

85 NMVPFFPPV 8.39800 8.08663 0.31137

86 ILDQVPFSV 8.48100 7.69086 0.79014

87 YLFPGPVTA 8.49500 8.33830 0.15670

88 YLDQVPFSV 8.63800 8.13639 0.50161

89 ILFQVPFSV 8.69900 8.43814 0.26086

90 ILWQVPFSV 8.77000 8.50511 0.26489

Correlation Coeffecient and Stand Error

CORL: 0.887064 CORL~2: 0.786883

RES: 0.366976 SEE: 0.038683

Statistical indices of Training calculation:

ITERATION (B): 29

No. Sequence Expt. Act Predicted Act Difference

1 VALVGLFVL 5.14800 5.74454 -0.59654

2 GTLVALVGL 5.34200 5.92748 -0.58548

3 LQTTIHDII 5.50100 5.79765 -0.29665

4 SLHVGTQCA 5.84200 6.14001 -0.29801

5 ALPYWNFAT 5.86900 6.64896 -0.77996

6 SLNFMGYVI 5.88100 5.96706 -0.08606

7 NLQSLTNLL 6.00000 6.69715 -0.69715

8 FVTWHRYHL 6.02500 5.72945 0.29555

9 DPKVKQWPL 6.17600 5.74027 0.43573

10 ITSQVPFSV 6.19600 6.57615 -0.38015

11 ALAKAAAAI 6.21100 6.23738 -0.02638

12 GLGQVPLIV 6.30100 6.56408 -0.26308

13 MLDLQPETT 6.33500 6.85256 -0.51756

14 LLSSNLSWL 6.34200 6.34995 -0.00795

15 GLACHQLCA 6.38000 6.04108 0.33892

16 LIGNESFAL 6.41500 7.05333 -0.63833

17 ALAKAAAAV 6.41900 6.47425 -0.05525

18 LLAVGATKV 6.47700 6.50693 -0.02993

19 ALAKAAAAL 6.51100 6.21998 0.29102

20 WILRGTSFV 6.55600 6.90737 -0.35137

21 IISCTCPTV 6.58000 6.66245 -0.08245

22 FLGGTPVCL 6.62300 6.87848 -0.25548

23 ALIHHNTHL 6.62300 6.79022 -0.16722

24 NLSWLSLDV 6.63900 6.04710 0.59190

25 YMIMVKCWM 6.66300 6.67242 -0.00942

26 VLQAGFFLL 6.68200 7.04595 -0.36395

27 GTLGIVCPI 6.71400 6.50938 0.20462

28 VILGVLLLI 6.78500 7.47755 -0.69255

29 VTWHRYHLL 6.79300 6.55649 0.23651

30 PLLPIFFCL 6.79600 7.52934 -0.73334

31 TLGIVCPIC 6.81500 5.95876 0.85624

32 CLTSTVQLV 6.83200 7.10689 -0.27489

33 ILLLCLIFL 6.84500 6.80086 0.04414

34 FAFRDLCIV 6.88600 6.65805 0.22795

35 FLEPGPVTA 6.89800 7.48199 -0.58399

36 ALAKAAAAA 6.94700 6.78891 0.15809

37 LMAVVLASL 6.95400 7.50462 -0.55062

38 YVITTQHWL 6.98300 6.33804 0.64496

39 LLCLIFLLV 6.99600 7.51719 -0.52119

40 ITAQVPFSV 7.02000 6.65752 0.36248

41 YLEPGPVTL 7.05800 7.15128 -0.09328

42 YTDQVPFSV 7.06600 7.06669 -0.00069

43 NLYVSLLLL 7.11400 6.98763 0.12637

44 ILHNGAYSL 7.12700 7.35209 -0.22509

45 SIISAVVGI 7.15900 7.30091 -0.14191

46 VVMGTLVAL 7.17400 7.31183 -0.13783

47 YLEPGPVTI 7.18700 7.16868 0.01832

48 GLSRYVARL 7.24800 7.46173 -0.21373

49 LLAQFTSAI 7.30100 7.43394 -0.13294

50 VLLDYQGML 7.32800 7.59528 -0.26728

51 YLEPGPVTV 7.34200 7.40555 -0.06355

52 ILSPFMPLL 7.34700 7.14528 0.20172

53 YLSPGPVTA 7.38300 7.54917 -0.16617

54 IIDQVPFSV 7.39800 7.65277 -0.25477

55 SVYDFFVWL 7.44400 7.36737 0.07663

56 ITWQVPFSV 7.46300 7.43661 0.02639

57 ITYQVPFSV 7.48000 7.65754 -0.17754

58 GLYSSTVPV 7.48100 7.62373 -0.14273

59 VMGTLVALV 7.55300 7.23885 0.31415

60 LLLCLIFLL 7.58500 7.15287 0.43213

61 SLDDYNHLV 7.58500 7.16870 0.41630

62 VLIQRNPQL 7.64400 6.95032 0.69368

63 SLYADSPSV 7.65800 7.70527 -0.04727

64 ILSQVPFSV 7.69900 7.64893 0.05007

65 IMDQVPFSV 7.71900 8.04089 -0.32189

66 QLFEDNYAL 7.76400 7.47588 0.28812

67 ALMDKSLHV 7.77000 7.52299 0.24701

68 YAIDLPVSV 7.79600 7.59410 0.20190

69 FVWLHYYSV 7.82400 8.12359 -0.29959

70 MLGTHTMEV 7.84500 7.31538 0.52962

71 LLFGYPVYV 7.88600 8.03592 -0.14992

72 ILKEPVHGV 7.92100 7.59207 0.32893

73 YLMPGPVTV 7.93200 7.91662 0.01538

74 WLDQVPFSV 7.93900 7.95679 -0.01779

75 KTWGQYWQV 7.95500 7.68710 0.26790

76 ALMPLYACI 8.00000 7.44233 0.55767

77 YLAPGPVTA 8.03200 7.63054 0.40146

78 YLYPGPVTV 8.05100 8.31591 -0.26491

79 LLMGTLGIV 8.09700 7.68020 0.41680

80 YLWPGPVTV 8.12500 8.09497 0.03003

81 FLLTRILTI 8.14900 7.88536 0.26364

82 GLLGWSPQA 8.23700 8.20781 0.02919

83 ILYQVPFSV 8.31000 8.73032 -0.42032

84 GILTVILGV 8.34700 7.83577 0.51123

85 NMVPFFPPV 8.39800 8.08896 0.30904

86 ILDQVPFSV 8.48100 7.69265 0.78835

87 YLFPGPVTA 8.49500 8.34245 0.15255

88 YLDQVPFSV 8.63800 8.13947 0.49853

89 ILFQVPFSV 8.69900 8.44221 0.25679

90 ILWQVPFSV 8.77000 8.50939 0.26061

Correlation Coeffecient and Stand Error

CORL: 0.887072 CORL~2: 0.786897

RES: 0.366967 SEE: 0.038682

Statistical indices of Training calculation:

ITERATION (A): 30

No. Sequence Expt. Act Predicted Act Difference

1 VALVGLFVL 5.14800 5.74964 -0.60164

2 GTLVALVGL 5.34200 5.93172 -0.58972

3 LQTTIHDII 5.50100 5.80290 -0.30190

4 SLHVGTQCA 5.84200 6.14426 -0.30226

5 ALPYWNFAT 5.86900 6.65088 -0.78188

6 SLNFMGYVI 5.88100 5.97149 -0.09049

7 NLQSLTNLL 6.00000 6.69902 -0.69902

8 FVTWHRYHL 6.02500 5.73473 0.29027

9 DPKVKQWPL 6.17600 5.74485 0.43115

10 ITSQVPFSV 6.19600 6.57790 -0.38190

11 ALAKAAAAI 6.21100 6.24090 -0.02990

12 GLGQVPLIV 6.30100 6.56612 -0.26512

13 MLDLQPETT 6.33500 6.85400 -0.51900

14 LLSSNLSWL 6.34200 6.35313 -0.01113

15 GLACHQLCA 6.38000 6.04619 0.33381

16 LIGNESFAL 6.41500 7.05424 -0.63924

17 ALAKAAAAV 6.41900 6.47699 -0.05799

18 LLAVGATKV 6.47700 6.50950 -0.03250

19 ALAKAAAAL 6.51100 6.22362 0.28738

20 WILRGTSFV 6.55600 6.90858 -0.35258

21 IISCTCPTV 6.58000 6.66483 -0.08483

22 FLGGTPVCL 6.62300 6.87974 -0.25674

23 ALIHHNTHL 6.62300 6.79176 -0.16876

24 NLSWLSLDV 6.63900 6.05137 0.58763

25 YMIMVKCWM 6.66300 6.67434 -0.01134

26 VLQAGFFLL 6.68200 7.04680 -0.36480

27 GTLGIVCPI 6.71400 6.51190 0.20210

28 VILGVLLLI 6.78500 7.47689 -0.69189

29 VTWHRYHLL 6.79300 6.55862 0.23438

30 PLLPIFFCL 6.79600 7.52898 -0.73298

31 TLGIVCPIC 6.81500 5.96306 0.85194

32 CLTSTVQLV 6.83200 7.10689 -0.27489

33 ILLLCLIFL 6.84500 6.80225 0.04275

34 FAFRDLCIV 6.88600 6.65972 0.22628

35 FLEPGPVTA 6.89800 7.48164 -0.58364

36 ALAKAAAAA 6.94700 6.79063 0.15637

37 LMAVVLASL 6.95400 7.50341 -0.54941

38 YVITTQHWL 6.98300 6.34147 0.64153

39 LLCLIFLLV 6.99600 7.51601 -0.52001

40 ITAQVPFSV 7.02000 6.65890 0.36110

41 YLEPGPVTL 7.05800 7.15205 -0.09405

42 YTDQVPFSV 7.06600 7.06669 -0.00069

43 NLYVSLLLL 7.11400 6.98862 0.12538

44 ILHNGAYSL 7.12700 7.35175 -0.22475

45 SIISAVVGI 7.15900 7.30082 -0.14182

46 VVMGTLVAL 7.17400 7.31155 -0.13755

47 YLEPGPVTI 7.18700 7.16933 0.01767

48 GLSRYVARL 7.24800 7.46095 -0.21295

49 LLAQFTSAI 7.30100 7.43310 -0.13210

50 VLLDYQGML 7.32800 7.59422 -0.26622

51 YLEPGPVTV 7.34200 7.40542 -0.06342

52 ILSPFMPLL 7.34700 7.14603 0.20097

53 YLSPGPVTA 7.38300 7.54865 -0.16565

54 IIDQVPFSV 7.39800 7.65122 -0.25322

55 SVYDFFVWL 7.44400 7.36701 0.07699

56 ITWQVPFSV 7.46300 7.43531 0.02769

57 ITYQVPFSV 7.48000 7.65551 -0.17551

58 GLYSSTVPV 7.48100 7.62270 -0.14170

59 VMGTLVALV 7.55300 7.23887 0.31413

60 LLLCLIFLL 7.58500 7.15373 0.43127

61 SLDDYNHLV 7.58500 7.16906 0.41594

62 VLIQRNPQL 7.64400 6.95126 0.69274

63 SLYADSPSV 7.65800 7.70372 -0.04572

64 ILSQVPFSV 7.69900 7.64727 0.05173

65 IMDQVPFSV 7.71900 8.03763 -0.31863

66 QLFEDNYAL 7.76400 7.47524 0.28876

67 ALMDKSLHV 7.77000 7.52177 0.24823

68 YAIDLPVSV 7.79600 7.59251 0.20349

69 FVWLHYYSV 7.82400 8.12058 -0.29658

70 MLGTHTMEV 7.84500 7.31520 0.52980

71 LLFGYPVYV 7.88600 8.03293 -0.14693

72 ILKEPVHGV 7.92100 7.59052 0.33048

73 YLMPGPVTV 7.93200 7.91463 0.01737

74 WLDQVPFSV 7.93900 7.95400 -0.01500

75 KTWGQYWQV 7.95500 7.68517 0.26983

76 ALMPLYACI 8.00000 7.44233 0.55767

77 YLAPGPVTA 8.03200 7.62965 0.40235

78 YLYPGPVTV 8.05100 8.31262 -0.26162

79 LLMGTLGIV 8.09700 7.67845 0.41855

80 YLWPGPVTV 8.12500 8.09243 0.03257

81 FLLTRILTI 8.14900 7.88351 0.26549

82 GLLGWSPQA 8.23700 8.20440 0.03260

83 ILYQVPFSV 8.31000 8.72488 -0.41488

84 GILTVILGV 8.34700 7.83411 0.51289

85 NMVPFFPPV 8.39800 8.08653 0.31147

86 ILDQVPFSV 8.48100 7.69083 0.79017

87 YLFPGPVTA 8.49500 8.33911 0.15589

88 YLDQVPFSV 8.63800 8.13606 0.50194

89 ILFQVPFSV 8.69900 8.43773 0.26127

90 ILWQVPFSV 8.77000 8.50468 0.26532

Correlation Coeffecient and Stand Error

CORL: 0.887076 CORL~2: 0.786903

RES: 0.366959 SEE: 0.038681

Statistical indices of Training calculation:

ITERATION (B): 30

No. Sequence Expt. Act Predicted Act Difference

1 VALVGLFVL 5.14800 5.74543 -0.59743

2 GTLVALVGL 5.34200 5.92832 -0.58632

3 LQTTIHDII 5.50100 5.79915 -0.29815

4 SLHVGTQCA 5.84200 6.14162 -0.29962

5 ALPYWNFAT 5.86900 6.64830 -0.77930

6 SLNFMGYVI 5.88100 5.96609 -0.08509

7 NLQSLTNLL 6.00000 6.69734 -0.69734

8 FVTWHRYHL 6.02500 5.72888 0.29612

9 DPKVKQWPL 6.17600 5.74029 0.43571

10 ITSQVPFSV 6.19600 6.57728 -0.38128

11 ALAKAAAAI 6.21100 6.23790 -0.02690

12 GLGQVPLIV 6.30100 6.56417 -0.26317

13 MLDLQPETT 6.33500 6.85295 -0.51795

14 LLSSNLSWL 6.34200 6.34998 -0.00798

15 GLACHQLCA 6.38000 6.04272 0.33728

16 LIGNESFAL 6.41500 7.05357 -0.63857

17 ALAKAAAAV 6.41900 6.47527 -0.05627

18 LLAVGATKV 6.47700 6.50734 -0.03034

19 ALAKAAAAL 6.51100 6.22052 0.29048

20 WILRGTSFV 6.55600 6.90746 -0.35146

21 IISCTCPTV 6.58000 6.66267 -0.08267

22 FLGGTPVCL 6.62300 6.87820 -0.25520

23 ALIHHNTHL 6.62300 6.79026 -0.16726

24 NLSWLSLDV 6.63900 6.04708 0.59192

25 YMIMVKCWM 6.66300 6.66974 -0.00674

26 VLQAGFFLL 6.68200 7.04551 -0.36351

27 GTLGIVCPI 6.71400 6.51065 0.20335

28 VILGVLLLI 6.78500 7.47713 -0.69213

29 VTWHRYHLL 6.79300 6.55677 0.23623

30 PLLPIFFCL 6.79600 7.52866 -0.73266

31 TLGIVCPIC 6.81500 5.95796 0.85704

32 CLTSTVQLV 6.83200 7.10682 -0.27482

33 ILLLCLIFL 6.84500 6.79915 0.04585

34 FAFRDLCIV 6.88600 6.65902 0.22698

35 FLEPGPVTA 6.89800 7.48307 -0.58507

36 ALAKAAAAA 6.94700 6.79062 0.15638

37 LMAVVLASL 6.95400 7.50337 -0.54937

38 YVITTQHWL 6.98300 6.33829 0.64471

39 LLCLIFLLV 6.99600 7.51579 -0.51979

40 ITAQVPFSV 7.02000 6.65850 0.36150

41 YLEPGPVTL 7.05800 7.15102 -0.09302

42 YTDQVPFSV 7.06600 7.06736 -0.00136

43 NLYVSLLLL 7.11400 6.98667 0.12733

44 ILHNGAYSL 7.12700 7.35185 -0.22485

45 SIISAVVGI 7.15900 7.30126 -0.14226

46 VVMGTLVAL 7.17400 7.31222 -0.13822

47 YLEPGPVTI 7.18700 7.16839 0.01861

48 GLSRYVARL 7.24800 7.46175 -0.21375

49 LLAQFTSAI 7.30100 7.43362 -0.13262

50 VLLDYQGML 7.32800 7.59490 -0.26690

51 YLEPGPVTV 7.34200 7.40577 -0.06377

52 ILSPFMPLL 7.34700 7.14481 0.20219

53 YLSPGPVTA 7.38300 7.55024 -0.16724

54 IIDQVPFSV 7.39800 7.65277 -0.25477

55 SVYDFFVWL 7.44400 7.36717 0.07683

56 ITWQVPFSV 7.46300 7.43707 0.02593

57 ITYQVPFSV 7.48000 7.65787 -0.17787

58 GLYSSTVPV 7.48100 7.62433 -0.14333

59 VMGTLVALV 7.55300 7.23867 0.31433

60 LLLCLIFLL 7.58500 7.15178 0.43322

61 SLDDYNHLV 7.58500 7.16940 0.41560

62 VLIQRNPQL 7.64400 6.95004 0.69396

63 SLYADSPSV 7.65800 7.70575 -0.04775

64 ILSQVPFSV 7.69900 7.64877 0.05023

65 IMDQVPFSV 7.71900 8.03994 -0.32094

66 QLFEDNYAL 7.76400 7.47546 0.28854

67 ALMDKSLHV 7.77000 7.52317 0.24683

68 YAIDLPVSV 7.79600 7.59530 0.20070

69 FVWLHYYSV 7.82400 8.12281 -0.29881

70 MLGTHTMEV 7.84500 7.31562 0.52938

71 LLFGYPVYV 7.88600 8.03497 -0.14897

72 ILKEPVHGV 7.92100 7.59201 0.32899

73 YLMPGPVTV 7.93200 7.91638 0.01562

74 WLDQVPFSV 7.93900 7.95631 -0.01731

75 KTWGQYWQV 7.95500 7.68768 0.26732

76 ALMPLYACI 8.00000 7.44199 0.55801

77 YLAPGPVTA 8.03200 7.63146 0.40054

78 YLYPGPVTV 8.05100 8.31548 -0.26448

79 LLMGTLGIV 8.09700 7.67991 0.41709

80 YLWPGPVTV 8.12500 8.09468 0.03032

81 FLLTRILTI 8.14900 7.88486 0.26414

82 GLLGWSPQA 8.23700 8.20877 0.02823

83 ILYQVPFSV 8.31000 8.72936 -0.41936

84 GILTVILGV 8.34700 7.83628 0.51072

85 NMVPFFPPV 8.39800 8.08865 0.30935

86 ILDQVPFSV 8.48100 7.69246 0.78854

87 YLFPGPVTA 8.49500 8.34288 0.15212

88 YLDQVPFSV 8.63800 8.13885 0.49915

89 ILFQVPFSV 8.69900 8.44142 0.25758

90 ILWQVPFSV 8.77000 8.50856 0.26144

Correlation Coeffecient and Stand Error

CORL: 0.887082 CORL~2: 0.786914

RES: 0.366951 SEE: 0.038680

Statistical indices of Training calculation:

ITERATION (A): 31

No. Sequence Expt. Act Predicted Act Difference

1 VALVGLFVL 5.14800 5.75005 -0.60205

2 GTLVALVGL 5.34200 5.93217 -0.59017

3 LQTTIHDII 5.50100 5.80391 -0.30291

4 SLHVGTQCA 5.84200 6.14547 -0.30347

5 ALPYWNFAT 5.86900 6.65005 -0.78105

6 SLNFMGYVI 5.88100 5.97011 -0.08911

7 NLQSLTNLL 6.00000 6.69903 -0.69903

8 FVTWHRYHL 6.02500 5.73367 0.29133

9 DPKVKQWPL 6.17600 5.74445 0.43155

10 ITSQVPFSV 6.19600 6.57887 -0.38287

11 ALAKAAAAI 6.21100 6.24109 -0.03009

12 GLGQVPLIV 6.30100 6.56602 -0.26502

13 MLDLQPETT 6.33500 6.85426 -0.51926

14 LLSSNLSWL 6.34200 6.35286 -0.01086

15 GLACHQLCA 6.38000 6.04734 0.33266

16 LIGNESFAL 6.41500 7.05439 -0.63939

17 ALAKAAAAV 6.41900 6.47776 -0.05876

18 LLAVGATKV 6.47700 6.50967 -0.03267

19 ALAKAAAAL 6.51100 6.22383 0.28717

20 WILRGTSFV 6.55600 6.90856 -0.35256

21 IISCTCPTV 6.58000 6.66483 -0.08483

22 FLGGTPVCL 6.62300 6.87935 -0.25635

23 ALIHHNTHL 6.62300 6.79166 -0.16866

24 NLSWLSLDV 6.63900 6.05095 0.58805

25 YMIMVKCWM 6.66300 6.67149 -0.00849

26 VLQAGFFLL 6.68200 7.04629 -0.36429

27 GTLGIVCPI 6.71400 6.51293 0.20107

28 VILGVLLLI 6.78500 7.47653 -0.69153

29 VTWHRYHLL 6.79300 6.55872 0.23428

30 PLLPIFFCL 6.79600 7.52833 -0.73233

31 TLGIVCPIC 6.81500 5.96186 0.85314

32 CLTSTVQLV 6.83200 7.10682 -0.27482

33 ILLLCLIFL 6.84500 6.80041 0.04459

34 FAFRDLCIV 6.88600 6.66054 0.22546

35 FLEPGPVTA 6.89800 7.48274 -0.58474

36 ALAKAAAAA 6.94700 6.79218 0.15482

37 LMAVVLASL 6.95400 7.50228 -0.54828

38 YVITTQHWL 6.98300 6.34141 0.64159

39 LLCLIFLLV 6.99600 7.51473 -0.51873

40 ITAQVPFSV 7.02000 6.65975 0.36025

41 YLEPGPVTL 7.05800 7.15171 -0.09371

42 YTDQVPFSV 7.06600 7.06736 -0.00136

43 NLYVSLLLL 7.11400 6.98757 0.12643

44 ILHNGAYSL 7.12700 7.35153 -0.22453

45 SIISAVVGI 7.15900 7.30118 -0.14218

46 VVMGTLVAL 7.17400 7.31197 -0.13797

47 YLEPGPVTI 7.18700 7.16898 0.01802

48 GLSRYVARL 7.24800 7.46104 -0.21304

49 LLAQFTSAI 7.30100 7.43286 -0.13186

50 VLLDYQGML 7.32800 7.59395 -0.26595

51 YLEPGPVTV 7.34200 7.40564 -0.06364

52 ILSPFMPLL 7.34700 7.14549 0.20151

53 YLSPGPVTA 7.38300 7.54976 -0.16676

54 IIDQVPFSV 7.39800 7.65136 -0.25336

55 SVYDFFVWL 7.44400 7.36685 0.07715

56 ITWQVPFSV 7.46300 7.43589 0.02711

57 ITYQVPFSV 7.48000 7.65603 -0.17603

58 GLYSSTVPV 7.48100 7.62339 -0.14239

59 VMGTLVALV 7.55300 7.23868 0.31432

60 LLLCLIFLL 7.58500 7.15255 0.43245

61 SLDDYNHLV 7.58500 7.16972 0.41528

62 VLIQRNPQL 7.64400 6.95090 0.69310

63 SLYADSPSV 7.65800 7.70433 -0.04633

64 ILSQVPFSV 7.69900 7.64727 0.05173

65 IMDQVPFSV 7.71900 8.03699 -0.31799

66 QLFEDNYAL 7.76400 7.47488 0.28912

67 ALMDKSLHV 7.77000 7.52206 0.24794

68 YAIDLPVSV 7.79600 7.59386 0.20214

69 FVWLHYYSV 7.82400 8.12008 -0.29608

70 MLGTHTMEV 7.84500 7.31545 0.52955

71 LLFGYPVYV 7.88600 8.03226 -0.14626

72 ILKEPVHGV 7.92100 7.59061 0.33039

73 YLMPGPVTV 7.93200 7.91457 0.01743

74 WLDQVPFSV 7.93900 7.95378 -0.01478

75 KTWGQYWQV 7.95500 7.68592 0.26908

76 ALMPLYACI 8.00000 7.44198 0.55802

77 YLAPGPVTA 8.03200 7.63065 0.40135

78 YLYPGPVTV 8.05100 8.31250 -0.26150

79 LLMGTLGIV 8.09700 7.67833 0.41867

80 YLWPGPVTV 8.12500 8.09237 0.03263

81 FLLTRILTI 8.14900 7.88317 0.26583

82 GLLGWSPQA 8.23700 8.20567 0.03133

83 ILYQVPFSV 8.31000 8.72443 -0.41443

84 GILTVILGV 8.34700 7.83478 0.51222

85 NMVPFFPPV 8.39800 8.08644 0.31156

86 ILDQVPFSV 8.48100 7.69081 0.79019

87 YLFPGPVTA 8.49500 8.33985 0.15515

88 YLDQVPFSV 8.63800 8.13576 0.50224

89 ILFQVPFSV 8.69900 8.43735 0.26165

90 ILWQVPFSV 8.77000 8.50429 0.26571

Correlation Coeffecient and Stand Error

CORL: 0.887085 CORL~2: 0.786920

RES: 0.366944 SEE: 0.038679

Statistical indices of Training calculation:

ITERATION (B): 31

No. Sequence Expt. Act Predicted Act Difference

1 VALVGLFVL 5.14800 5.74623 -0.59823

2 GTLVALVGL 5.34200 5.92909 -0.58709

3 LQTTIHDII 5.50100 5.80051 -0.29951

4 SLHVGTQCA 5.84200 6.14307 -0.30107

5 ALPYWNFAT 5.86900 6.64770 -0.77870

6 SLNFMGYVI 5.88100 5.96520 -0.08420

7 NLQSLTNLL 6.00000 6.69751 -0.69751

8 FVTWHRYHL 6.02500 5.72836 0.29664

9 DPKVKQWPL 6.17600 5.74031 0.43569

10 ITSQVPFSV 6.19600 6.57830 -0.38230

11 ALAKAAAAI 6.21100 6.23837 -0.02737

12 GLGQVPLIV 6.30100 6.56424 -0.26324

13 MLDLQPETT 6.33500 6.85331 -0.51831

14 LLSSNLSWL 6.34200 6.35000 -0.00800

15 GLACHQLCA 6.38000 6.04420 0.33580

16 LIGNESFAL 6.41500 7.05378 -0.63878

17 ALAKAAAAV 6.41900 6.47620 -0.05720

18 LLAVGATKV 6.47700 6.50772 -0.03072

19 ALAKAAAAL 6.51100 6.22102 0.28998

20 WILRGTSFV 6.55600 6.90754 -0.35154

21 IISCTCPTV 6.58000 6.66287 -0.08287

22 FLGGTPVCL 6.62300 6.87796 -0.25496

23 ALIHHNTHL 6.62300 6.79030 -0.16730

24 NLSWLSLDV 6.63900 6.04705 0.59195

25 YMIMVKCWM 6.66300 6.66732 -0.00432

26 VLQAGFFLL 6.68200 7.04512 -0.36312

27 GTLGIVCPI 6.71400 6.51179 0.20221

28 VILGVLLLI 6.78500 7.47674 -0.69174

29 VTWHRYHLL 6.79300 6.55703 0.23597

30 PLLPIFFCL 6.79600 7.52804 -0.73204

31 TLGIVCPIC 6.81500 5.95723 0.85777

32 CLTSTVQLV 6.83200 7.10675 -0.27475

33 ILLLCLIFL 6.84500 6.79759 0.04741

34 FAFRDLCIV 6.88600 6.65989 0.22611

35 FLEPGPVTA 6.89800 7.48405 -0.58605

36 ALAKAAAAA 6.94700 6.79217 0.15483

37 LMAVVLASL 6.95400 7.50224 -0.54824

38 YVITTQHWL 6.98300 6.33853 0.64447

39 LLCLIFLLV 6.99600 7.51453 -0.51853

40 ITAQVPFSV 7.02000 6.65939 0.36061

41 YLEPGPVTL 7.05800 7.15078 -0.09278

42 YTDQVPFSV 7.06600 7.06796 -0.00196

43 NLYVSLLLL 7.11400 6.98581 0.12819

44 ILHNGAYSL 7.12700 7.35163 -0.22463

45 SIISAVVGI 7.15900 7.30158 -0.14258

46 VVMGTLVAL 7.17400 7.31258 -0.13858

47 YLEPGPVTI 7.18700 7.16813 0.01887

48 GLSRYVARL 7.24800 7.46176 -0.21376

49 LLAQFTSAI 7.30100 7.43332 -0.13232

50 VLLDYQGML 7.32800 7.59457 -0.26657

51 YLEPGPVTV 7.34200 7.40596 -0.06396

52 ILSPFMPLL 7.34700 7.14439 0.20261

53 YLSPGPVTA 7.38300 7.55120 -0.16820

54 IIDQVPFSV 7.39800 7.65277 -0.25477

55 SVYDFFVWL 7.44400 7.36699 0.07701

56 ITWQVPFSV 7.46300 7.43748 0.02552

57 ITYQVPFSV 7.48000 7.65816 -0.17816

58 GLYSSTVPV 7.48100 7.62487 -0.14387

59 VMGTLVALV 7.55300 7.23851 0.31449

60 LLLCLIFLL 7.58500 7.15078 0.43422

61 SLDDYNHLV 7.58500 7.17003 0.41497

62 VLIQRNPQL 7.64400 6.94980 0.69420

63 SLYADSPSV 7.65800 7.70618 -0.04818

64 ILSQVPFSV 7.69900 7.64863 0.05037

65 IMDQVPFSV 7.71900 8.03909 -0.32009

66 QLFEDNYAL 7.76400 7.47508 0.28892

67 ALMDKSLHV 7.77000 7.52334 0.24666

68 YAIDLPVSV 7.79600 7.59639 0.19961

69 FVWLHYYSV 7.82400 8.12211 -0.29811

70 MLGTHTMEV 7.84500 7.31584 0.52916

71 LLFGYPVYV 7.88600 8.03411 -0.14811

72 ILKEPVHGV 7.92100 7.59195 0.32905

73 YLMPGPVTV 7.93200 7.91617 0.01583

74 WLDQVPFSV 7.93900 7.95588 -0.01688

75 KTWGQYWQV 7.95500 7.68819 0.26681

76 ALMPLYACI 8.00000 7.44168 0.55832

77 YLAPGPVTA 8.03200 7.63229 0.39971

78 YLYPGPVTV 8.05100 8.31510 -0.26410

79 LLMGTLGIV 8.09700 7.67965 0.41735

80 YLWPGPVTV 8.12500 8.09441 0.03059

81 FLLTRILTI 8.14900 7.88440 0.26460

82 GLLGWSPQA 8.23700 8.20963 0.02737

83 ILYQVPFSV 8.31000 8.72850 -0.41850

84 GILTVILGV 8.34700 7.83675 0.51025

85 NMVPFFPPV 8.39800 8.08836 0.30964

86 ILDQVPFSV 8.48100 7.69228 0.78872

87 YLFPGPVTA 8.49500 8.34327 0.15173

88 YLDQVPFSV 8.63800 8.13829 0.49971

89 ILFQVPFSV 8.69900 8.44070 0.25830

90 ILWQVPFSV 8.77000 8.50781 0.26219

Correlation Coeffecient and Stand Error

CORL: 0.887090 CORL~2: 0.786929

RES: 0.366938 SEE: 0.038679

Statistical indices of Training calculation:

ITERATION (A): 32

No. Sequence Expt. Act Predicted Act Difference

1 VALVGLFVL 5.14800 5.75043 -0.60243

2 GTLVALVGL 5.34200 5.93258 -0.59058

3 LQTTIHDII 5.50100 5.80483 -0.30383

4 SLHVGTQCA 5.84200 6.14657 -0.30457

5 ALPYWNFAT 5.86900 6.64930 -0.78030

6 SLNFMGYVI 5.88100 5.96886 -0.08786

7 NLQSLTNLL 6.00000 6.69904 -0.69904

8 FVTWHRYHL 6.02500 5.73272 0.29228

9 DPKVKQWPL 6.17600 5.74409 0.43191

10 ITSQVPFSV 6.19600 6.57974 -0.38374

11 ALAKAAAAI 6.21100 6.24127 -0.03027

12 GLGQVPLIV 6.30100 6.56592 -0.26492

13 MLDLQPETT 6.33500 6.85449 -0.51949

14 LLSSNLSWL 6.34200 6.35262 -0.01062

15 GLACHQLCA 6.38000 6.04839 0.33161

16 LIGNESFAL 6.41500 7.05452 -0.63952

17 ALAKAAAAV 6.41900 6.47845 -0.05945

18 LLAVGATKV 6.47700 6.50983 -0.03283

19 ALAKAAAAL 6.51100 6.22402 0.28698

20 WILRGTSFV 6.55600 6.90854 -0.35254

21 IISCTCPTV 6.58000 6.66483 -0.08483

22 FLGGTPVCL 6.62300 6.87901 -0.25601

23 ALIHHNTHL 6.62300 6.79157 -0.16857

24 NLSWLSLDV 6.63900 6.05057 0.58843

25 YMIMVKCWM 6.66300 6.66892 -0.00592

26 VLQAGFFLL 6.68200 7.04582 -0.36382

27 GTLGIVCPI 6.71400 6.51386 0.20014

28 VILGVLLLI 6.78500 7.47620 -0.69120

29 VTWHRYHLL 6.79300 6.55880 0.23420

30 PLLPIFFCL 6.79600 7.52775 -0.73175

31 TLGIVCPIC 6.81500 5.96079 0.85421

32 CLTSTVQLV 6.83200 7.10676 -0.27476

33 ILLLCLIFL 6.84500 6.79874 0.04626

34 FAFRDLCIV 6.88600 6.66127 0.22473

35 FLEPGPVTA 6.89800 7.48374 -0.58574

36 ALAKAAAAA 6.94700 6.79358 0.15342

37 LMAVVLASL 6.95400 7.50126 -0.54726

38 YVITTQHWL 6.98300 6.34136 0.64164

39 LLCLIFLLV 6.99600 7.51356 -0.51756

40 ITAQVPFSV 7.02000 6.66053 0.35947

41 YLEPGPVTL 7.05800 7.15141 -0.09341

42 YTDQVPFSV 7.06600 7.06797 -0.00197

43 NLYVSLLLL 7.11400 6.98662 0.12738

44 ILHNGAYSL 7.12700 7.35134 -0.22434

45 SIISAVVGI 7.15900 7.30150 -0.14250

46 VVMGTLVAL 7.17400 7.31235 -0.13835

47 YLEPGPVTI 7.18700 7.16866 0.01834

48 GLSRYVARL 7.24800 7.46112 -0.21312

49 LLAQFTSAI 7.30100 7.43263 -0.13163

50 VLLDYQGML 7.32800 7.59370 -0.26570

51 YLEPGPVTV 7.34200 7.40584 -0.06384

52 ILSPFMPLL 7.34700 7.14501 0.20199

53 YLSPGPVTA 7.38300 7.55077 -0.16777

54 IIDQVPFSV 7.39800 7.65149 -0.25349

55 SVYDFFVWL 7.44400 7.36671 0.07729

56 ITWQVPFSV 7.46300 7.43642 0.02658

57 ITYQVPFSV 7.48000 7.65649 -0.17649

58 GLYSSTVPV 7.48100 7.62402 -0.14302

59 VMGTLVALV 7.55300 7.23852 0.31448

60 LLLCLIFLL 7.58500 7.15149 0.43351

61 SLDDYNHLV 7.58500 7.17031 0.41469

62 VLIQRNPQL 7.64400 6.95057 0.69343

63 SLYADSPSV 7.65800 7.70489 -0.04689

64 ILSQVPFSV 7.69900 7.64727 0.05173

65 IMDQVPFSV 7.71900 8.03641 -0.31741

66 QLFEDNYAL 7.76400 7.47456 0.28944

67 ALMDKSLHV 7.77000 7.52233 0.24767

68 YAIDLPVSV 7.79600 7.59508 0.20092

69 FVWLHYYSV 7.82400 8.11963 -0.29563

70 MLGTHTMEV 7.84500 7.31568 0.52932

71 LLFGYPVYV 7.88600 8.03165 -0.14565

72 ILKEPVHGV 7.92100 7.59069 0.33031

73 YLMPGPVTV 7.93200 7.91452 0.01748

74 WLDQVPFSV 7.93900 7.95358 -0.01458

75 KTWGQYWQV 7.95500 7.68659 0.26841

76 ALMPLYACI 8.00000 7.44166 0.55834

77 YLAPGPVTA 8.03200 7.63155 0.40045

78 YLYPGPVTV 8.05100 8.31239 -0.26139

79 LLMGTLGIV 8.09700 7.67821 0.41879

80 YLWPGPVTV 8.12500 8.09231 0.03269

81 FLLTRILTI 8.14900 7.88286 0.26614

82 GLLGWSPQA 8.23700 8.20682 0.03018

83 ILYQVPFSV 8.31000 8.72402 -0.41402

84 GILTVILGV 8.34700 7.83538 0.51162

85 NMVPFFPPV 8.39800 8.08636 0.31164

86 ILDQVPFSV 8.48100 7.69078 0.79022

87 YLFPGPVTA 8.49500 8.34052 0.15448

88 YLDQVPFSV 8.63800 8.13549 0.50251

89 ILFQVPFSV 8.69900 8.43702 0.26198

90 ILWQVPFSV 8.77000 8.50394 0.26606

Correlation Coeffecient and Stand Error

CORL: 0.887093 CORL~2: 0.786934

RES: 0.366932 SEE: 0.038678

Statistical indices of Training calculation:

ITERATION (B): 32

No. Sequence Expt. Act Predicted Act Difference

1 VALVGLFVL 5.14800 5.74695 -0.59895

2 GTLVALVGL 5.34200 5.92977 -0.58777

3 LQTTIHDII 5.50100 5.80174 -0.30074

4 SLHVGTQCA 5.84200 6.14439 -0.30239

5 ALPYWNFAT 5.86900 6.64716 -0.77816

6 SLNFMGYVI 5.88100 5.96440 -0.08340

7 NLQSLTNLL 6.00000 6.69766 -0.69766

8 FVTWHRYHL 6.02500 5.72788 0.29712

9 DPKVKQWPL 6.17600 5.74032 0.43568

10 ITSQVPFSV 6.19600 6.57923 -0.38323

11 ALAKAAAAI 6.21100 6.23880 -0.02780

12 GLGQVPLIV 6.30100 6.56431 -0.26331

13 MLDLQPETT 6.33500 6.85363 -0.51863

14 LLSSNLSWL 6.34200 6.35002 -0.00802

15 GLACHQLCA 6.38000 6.04554 0.33446

16 LIGNESFAL 6.41500 7.05397 -0.63897

17 ALAKAAAAV 6.41900 6.47703 -0.05803

18 LLAVGATKV 6.47700 6.50806 -0.03106

19 ALAKAAAAL 6.51100 6.22147 0.28953

20 WILRGTSFV 6.55600 6.90761 -0.35161

21 IISCTCPTV 6.58000 6.66305 -0.08305

22 FLGGTPVCL 6.62300 6.87774 -0.25474

23 ALIHHNTHL 6.62300 6.79034 -0.16734

24 NLSWLSLDV 6.63900 6.04703 0.59197

25 YMIMVKCWM 6.66300 6.66512 -0.00212

26 VLQAGFFLL 6.68200 7.04477 -0.36277

27 GTLGIVCPI 6.71400 6.51282 0.20118

28 VILGVLLLI 6.78500 7.47639 -0.69139

29 VTWHRYHLL 6.79300 6.55727 0.23573

30 PLLPIFFCL 6.79600 7.52748 -0.73148

31 TLGIVCPIC 6.81500 5.95658 0.85842

32 CLTSTVQLV 6.83200 7.10670 -0.27470

33 ILLLCLIFL 6.84500 6.79618 0.04882

34 FAFRDLCIV 6.88600 6.66069 0.22531

35 FLEPGPVTA 6.89800 7.48493 -0.58693

36 ALAKAAAAA 6.94700 6.79357 0.15343

37 LMAVVLASL 6.95400 7.50122 -0.54722

38 YVITTQHWL 6.98300 6.33874 0.64426

39 LLCLIFLLV 6.99600 7.51338 -0.51738

40 ITAQVPFSV 7.02000 6.66020 0.35980

41 YLEPGPVTL 7.05800 7.15057 -0.09257

42 YTDQVPFSV 7.06600 7.06851 -0.00251

43 NLYVSLLLL 7.11400 6.98502 0.12898

44 ILHNGAYSL 7.12700 7.35143 -0.22443

45 SIISAVVGI 7.15900 7.30186 -0.14286

46 VVMGTLVAL 7.17400 7.31290 -0.13890

47 YLEPGPVTI 7.18700 7.16790 0.01910

48 GLSRYVARL 7.24800 7.46177 -0.21377

49 LLAQFTSAI 7.30100 7.43305 -0.13205

50 VLLDYQGML 7.32800 7.59426 -0.26626

51 YLEPGPVTV 7.34200 7.40614 -0.06414

52 ILSPFMPLL 7.34700 7.14401 0.20299

53 YLSPGPVTA 7.38300 7.55208 -0.16908

54 IIDQVPFSV 7.39800 7.65277 -0.25477

55 SVYDFFVWL 7.44400 7.36684 0.07716

56 ITWQVPFSV 7.46300 7.43785 0.02515

57 ITYQVPFSV 7.48000 7.65843 -0.17843

58 GLYSSTVPV 7.48100 7.62536 -0.14436

59 VMGTLVALV 7.55300 7.23836 0.31464

60 LLLCLIFLL 7.58500 7.14988 0.43512

61 SLDDYNHLV 7.58500 7.17059 0.41441

62 VLIQRNPQL 7.64400 6.94957 0.69443

63 SLYADSPSV 7.65800 7.70657 -0.04857

64 ILSQVPFSV 7.69900 7.64851 0.05049

65 IMDQVPFSV 7.71900 8.03831 -0.31931

66 QLFEDNYAL 7.76400 7.47474 0.28926

67 ALMDKSLHV 7.77000 7.52349 0.24651

68 YAIDLPVSV 7.79600 7.59738 0.19862

69 FVWLHYYSV 7.82400 8.12147 -0.29747

70 MLGTHTMEV 7.84500 7.31603 0.52897

71 LLFGYPVYV 7.88600 8.03333 -0.14733

72 ILKEPVHGV 7.92100 7.59191 0.32909

73 YLMPGPVTV 7.93200 7.91598 0.01602

74 WLDQVPFSV 7.93900 7.95549 -0.01649

75 KTWGQYWQV 7.95500 7.68866 0.26634

76 ALMPLYACI 8.00000 7.44139 0.55861

77 YLAPGPVTA 8.03200 7.63305 0.39895

78 YLYPGPVTV 8.05100 8.31475 -0.26375

79 LLMGTLGIV 8.09700 7.67942 0.41758

80 YLWPGPVTV 8.12500 8.09417 0.03083

81 FLLTRILTI 8.14900 7.88398 0.26502

82 GLLGWSPQA 8.23700 8.21041 0.02659

83 ILYQVPFSV 8.31000 8.72771 -0.41771

84 GILTVILGV 8.34700 7.83717 0.50983

85 NMVPFFPPV 8.39800 8.08811 0.30989

86 ILDQVPFSV 8.48100 7.69212 0.78888

87 YLFPGPVTA 8.49500 8.34363 0.15137

88 YLDQVPFSV 8.63800 8.13779 0.50021

89 ILFQVPFSV 8.69900 8.44006 0.25894

90 ILWQVPFSV 8.77000 8.50713 0.26287

Correlation Coeffecient and Stand Error

CORL: 0.887097 CORL~2: 0.786941

RES: 0.366927 SEE: 0.038677

Statistical indices of Training calculation:

ITERATION (A): 33

No. Sequence Expt. Act Predicted Act Difference

1 VALVGLFVL 5.14800 5.75077 -0.60277

2 GTLVALVGL 5.34200 5.93295 -0.59095

3 LQTTIHDII 5.50100 5.80567 -0.30467

4 SLHVGTQCA 5.84200 6.14756 -0.30556

5 ALPYWNFAT 5.86900 6.64861 -0.77961

6 SLNFMGYVI 5.88100 5.96772 -0.08672

7 NLQSLTNLL 6.00000 6.69906 -0.69906

8 FVTWHRYHL 6.02500 5.73185 0.29315

9 DPKVKQWPL 6.17600 5.74377 0.43223

10 ITSQVPFSV 6.19600 6.58054 -0.38454

11 ALAKAAAAI 6.21100 6.24143 -0.03043

12 GLGQVPLIV 6.30100 6.56584 -0.26484

13 MLDLQPETT 6.33500 6.85471 -0.51971

14 LLSSNLSWL 6.34200 6.35240 -0.01040

15 GLACHQLCA 6.38000 6.04935 0.33065

16 LIGNESFAL 6.41500 7.05465 -0.63965

17 ALAKAAAAV 6.41900 6.47908 -0.06008

18 LLAVGATKV 6.47700 6.50997 -0.03297

19 ALAKAAAAL 6.51100 6.22420 0.28680

20 WILRGTSFV 6.55600 6.90852 -0.35252

21 IISCTCPTV 6.58000 6.66483 -0.08483

22 FLGGTPVCL 6.62300 6.87869 -0.25569

23 ALIHHNTHL 6.62300 6.79150 -0.16850

24 NLSWLSLDV 6.63900 6.05023 0.58877

25 YMIMVKCWM 6.66300 6.66659 -0.00359

26 VLQAGFFLL 6.68200 7.04541 -0.36341

27 GTLGIVCPI 6.71400 6.51470 0.19930

28 VILGVLLLI 6.78500 7.47589 -0.69089

29 VTWHRYHLL 6.79300 6.55887 0.23413

30 PLLPIFFCL 6.79600 7.52721 -0.73121

31 TLGIVCPIC 6.81500 5.95981 0.85519

32 CLTSTVQLV 6.83200 7.10670 -0.27470

33 ILLLCLIFL 6.84500 6.79723 0.04777

34 FAFRDLCIV 6.88600 6.66194 0.22406

35 FLEPGPVTA 6.89800 7.48465 -0.58665

36 ALAKAAAAA 6.94700 6.79485 0.15215

37 LMAVVLASL 6.95400 7.50033 -0.54633

38 YVITTQHWL 6.98300 6.34132 0.64168

39 LLCLIFLLV 6.99600 7.51251 -0.51651

40 ITAQVPFSV 7.02000 6.66123 0.35877

41 YLEPGPVTL 7.05800 7.15114 -0.09314

42 YTDQVPFSV 7.06600 7.06852 -0.00252

43 NLYVSLLLL 7.11400 6.98577 0.12823

44 ILHNGAYSL 7.12700 7.35117 -0.22417

45 SIISAVVGI 7.15900 7.30179 -0.14279

46 VVMGTLVAL 7.17400 7.31269 -0.13869

47 YLEPGPVTI 7.18700 7.16838 0.01862

48 GLSRYVARL 7.24800 7.46119 -0.21319

49 LLAQFTSAI 7.30100 7.43242 -0.13142

50 VLLDYQGML 7.32800 7.59347 -0.26547

51 YLEPGPVTV 7.34200 7.40603 -0.06403

52 ILSPFMPLL 7.34700 7.14457 0.20243

53 YLSPGPVTA 7.38300 7.55167 -0.16867

54 IIDQVPFSV 7.39800 7.65161 -0.25361

55 SVYDFFVWL 7.44400 7.36658 0.07742

56 ITWQVPFSV 7.46300 7.43689 0.02611

57 ITYQVPFSV 7.48000 7.65692 -0.17692

58 GLYSSTVPV 7.48100 7.62458 -0.14358

59 VMGTLVALV 7.55300 7.23837 0.31463

60 LLLCLIFLL 7.58500 7.15053 0.43447

61 SLDDYNHLV 7.58500 7.17085 0.41415

62 VLIQRNPQL 7.64400 6.95028 0.69372

63 SLYADSPSV 7.65800 7.70539 -0.04739

64 ILSQVPFSV 7.69900 7.64727 0.05173

65 IMDQVPFSV 7.71900 8.03588 -0.31688

66 QLFEDNYAL 7.76400 7.47426 0.28974

67 ALMDKSLHV 7.77000 7.52257 0.24743

68 YAIDLPVSV 7.79600 7.59618 0.19982

69 FVWLHYYSV 7.82400 8.11922 -0.29522

70 MLGTHTMEV 7.84500 7.31589 0.52911

71 LLFGYPVYV 7.88600 8.03109 -0.14509

72 ILKEPVHGV 7.92100 7.59075 0.33025

73 YLMPGPVTV 7.93200 7.91448 0.01752

74 WLDQVPFSV 7.93900 7.95340 -0.01440

75 KTWGQYWQV 7.95500 7.68720 0.26780

76 ALMPLYACI 8.00000 7.44138 0.55862

77 YLAPGPVTA 8.03200 7.63237 0.39963

78 YLYPGPVTV 8.05100 8.31229 -0.26129

79 LLMGTLGIV 8.09700 7.67811 0.41889

80 YLWPGPVTV 8.12500 8.09226 0.03274

81 FLLTRILTI 8.14900 7.88258 0.26642

82 GLLGWSPQA 8.23700 8.20785 0.02915

83 ILYQVPFSV 8.31000 8.72364 -0.41364

84 GILTVILGV 8.34700 7.83592 0.51108

85 NMVPFFPPV 8.39800 8.08628 0.31172

86 ILDQVPFSV 8.48100 7.69075 0.79025

87 YLFPGPVTA 8.49500 8.34112 0.15388

88 YLDQVPFSV 8.63800 8.13525 0.50275

89 ILFQVPFSV 8.69900 8.43671 0.26229

90 ILWQVPFSV 8.77000 8.50362 0.26638

Correlation Coeffecient and Stand Error

CORL: 0.887100 CORL~2: 0.786946

RES: 0.366922 SEE: 0.038677

Statistical indices of Training calculation:

ITERATION (B): 33

No. Sequence Expt. Act Predicted Act Difference

1 VALVGLFVL 5.14800 5.74761 -0.59961

2 GTLVALVGL 5.34200 5.93040 -0.58840

3 LQTTIHDII 5.50100 5.80286 -0.30186

4 SLHVGTQCA 5.84200 6.14559 -0.30359

5 ALPYWNFAT 5.86900 6.64667 -0.77767

6 SLNFMGYVI 5.88100 5.96367 -0.08267

7 NLQSLTNLL 6.00000 6.69780 -0.69780

8 FVTWHRYHL 6.02500 5.72746 0.29754

9 DPKVKQWPL 6.17600 5.74034 0.43566

10 ITSQVPFSV 6.19600 6.58006 -0.38406

11 ALAKAAAAI 6.21100 6.23918 -0.02818

12 GLGQVPLIV 6.30100 6.56437 -0.26337

13 MLDLQPETT 6.33500 6.85393 -0.51893

14 LLSSNLSWL 6.34200 6.35004 -0.00804

15 GLACHQLCA 6.38000 6.04675 0.33325

16 LIGNESFAL 6.41500 7.05415 -0.63915

17 ALAKAAAAV 6.41900 6.47780 -0.05880

18 LLAVGATKV 6.47700 6.50836 -0.03136

19 ALAKAAAAL 6.51100 6.22187 0.28913

20 WILRGTSFV 6.55600 6.90768 -0.35168

21 IISCTCPTV 6.58000 6.66321 -0.08321

22 FLGGTPVCL 6.62300 6.87754 -0.25454

23 ALIHHNTHL 6.62300 6.79037 -0.16737

24 NLSWLSLDV 6.63900 6.04701 0.59199

25 YMIMVKCWM 6.66300 6.66314 -0.00014

26 VLQAGFFLL 6.68200 7.04445 -0.36245

27 GTLGIVCPI 6.71400 6.51376 0.20024

28 VILGVLLLI 6.78500 7.47607 -0.69107

29 VTWHRYHLL 6.79300 6.55748 0.23552

30 PLLPIFFCL 6.79600 7.52697 -0.73097

31 TLGIVCPIC 6.81500 5.95599 0.85901

32 CLTSTVQLV 6.83200 7.10664 -0.27464

33 ILLLCLIFL 6.84500 6.79490 0.05010

34 FAFRDLCIV 6.88600 6.66141 0.22459

35 FLEPGPVTA 6.89800 7.48573 -0.58773

36 ALAKAAAAA 6.94700 6.79484 0.15216

37 LMAVVLASL 6.95400 7.50030 -0.54630

38 YVITTQHWL 6.98300 6.33894 0.64406

39 LLCLIFLLV 6.99600 7.51234 -0.51634

40 ITAQVPFSV 7.02000 6.66093 0.35907

41 YLEPGPVTL 7.05800 7.15037 -0.09237

42 YTDQVPFSV 7.06600 7.06901 -0.00301

43 NLYVSLLLL 7.11400 6.98431 0.12969

44 ILHNGAYSL 7.12700 7.35125 -0.22425

45 SIISAVVGI 7.15900 7.30212 -0.14312

46 VVMGTLVAL 7.17400 7.31319 -0.13919

47 YLEPGPVTI 7.18700 7.16768 0.01932

48 GLSRYVARL 7.24800 7.46178 -0.21378

49 LLAQFTSAI 7.30100 7.43280 -0.13180

50 VLLDYQGML 7.32800 7.59399 -0.26599

51 YLEPGPVTV 7.34200 7.40629 -0.06429

52 ILSPFMPLL 7.34700 7.14366 0.20334

53 YLSPGPVTA 7.38300 7.55287 -0.16987

54 IIDQVPFSV 7.39800 7.65277 -0.25477

55 SVYDFFVWL 7.44400 7.36670 0.07730

56 ITWQVPFSV 7.46300 7.43820 0.02480

57 ITYQVPFSV 7.48000 7.65868 -0.17868

58 GLYSSTVPV 7.48100 7.62580 -0.14480

59 VMGTLVALV 7.55300 7.23823 0.31477

60 LLLCLIFLL 7.58500 7.14906 0.43594

61 SLDDYNHLV 7.58500 7.17111 0.41389

62 VLIQRNPQL 7.64400 6.94937 0.69463

63 SLYADSPSV 7.65800 7.70692 -0.04892

64 ILSQVPFSV 7.69900 7.64839 0.05061

65 IMDQVPFSV 7.71900 8.03762 -0.31862

66 QLFEDNYAL 7.76400 7.47443 0.28957

67 ALMDKSLHV 7.77000 7.52363 0.24637

68 YAIDLPVSV 7.79600 7.59827 0.19773

69 FVWLHYYSV 7.82400 8.12089 -0.29689

70 MLGTHTMEV 7.84500 7.31621 0.52879

71 LLFGYPVYV 7.88600 8.03263 -0.14663

72 ILKEPVHGV 7.92100 7.59187 0.32913

73 YLMPGPVTV 7.93200 7.91580 0.01620

74 WLDQVPFSV 7.93900 7.95513 -0.01613

75 KTWGQYWQV 7.95500 7.68908 0.26592

76 ALMPLYACI 8.00000 7.44113 0.55887

77 YLAPGPVTA 8.03200 7.63373 0.39827

78 YLYPGPVTV 8.05100 8.31443 -0.26343

79 LLMGTLGIV 8.09700 7.67920 0.41780

80 YLWPGPVTV 8.12500 8.09395 0.03105

81 FLLTRILTI 8.14900 7.88360 0.26540

82 GLLGWSPQA 8.23700 8.21112 0.02588

83 ILYQVPFSV 8.31000 8.72700 -0.41700

84 GILTVILGV 8.34700 7.83755 0.50945

85 NMVPFFPPV 8.39800 8.08787 0.31013

86 ILDQVPFSV 8.48100 7.69197 0.78903

87 YLFPGPVTA 8.49500 8.34395 0.15105

88 YLDQVPFSV 8.63800 8.13733 0.50067

89 ILFQVPFSV 8.69900 8.43947 0.25953

90 ILWQVPFSV 8.77000 8.50652 0.26348

Correlation Coeffecient and Stand Error

CORL: 0.887102 CORL~2: 0.786951

RES: 0.366918 SEE: 0.038677

Statistical indices of Training calculation:

ITERATION (A): 34

No. Sequence Expt. Act Predicted Act Difference

1 VALVGLFVL 5.14800 5.75108 -0.60308

2 GTLVALVGL 5.34200 5.93329 -0.59129

3 LQTTIHDII 5.50100 5.80642 -0.30542

4 SLHVGTQCA 5.84200 6.14847 -0.30647

5 ALPYWNFAT 5.86900 6.64799 -0.77899

6 SLNFMGYVI 5.88100 5.96670 -0.08570

7 NLQSLTNLL 6.00000 6.69907 -0.69907

8 FVTWHRYHL 6.02500 5.73107 0.29393

9 DPKVKQWPL 6.17600 5.74348 0.43252

10 ITSQVPFSV 6.19600 6.58126 -0.38526

11 ALAKAAAAI 6.21100 6.24159 -0.03059

12 GLGQVPLIV 6.30100 6.56576 -0.26476

13 MLDLQPETT 6.33500 6.85491 -0.51991

14 LLSSNLSWL 6.34200 6.35221 -0.01021

15 GLACHQLCA 6.38000 6.05022 0.32978

16 LIGNESFAL 6.41500 7.05476 -0.63976

17 ALAKAAAAV 6.41900 6.47966 -0.06066

18 LLAVGATKV 6.47700 6.51011 -0.03311

19 ALAKAAAAL 6.51100 6.22436 0.28664

20 WILRGTSFV 6.55600 6.90850 -0.35250

21 IISCTCPTV 6.58000 6.66483 -0.08483

22 FLGGTPVCL 6.62300 6.87841 -0.25541

23 ALIHHNTHL 6.62300 6.79143 -0.16843

24 NLSWLSLDV 6.63900 6.04991 0.58909

25 YMIMVKCWM 6.66300 6.66448 -0.00148

26 VLQAGFFLL 6.68200 7.04503 -0.36303

27 GTLGIVCPI 6.71400 6.51547 0.19853

28 VILGVLLLI 6.78500 7.47562 -0.69062

29 VTWHRYHLL 6.79300 6.55894 0.23406

30 PLLPIFFCL 6.79600 7.52673 -0.73073

31 TLGIVCPIC 6.81500 5.95893 0.85607

32 CLTSTVQLV 6.83200 7.10665 -0.27465

33 ILLLCLIFL 6.84500 6.79585 0.04915

34 FAFRDLCIV 6.88600 6.66255 0.22345

35 FLEPGPVTA 6.89800 7.48547 -0.58747

36 ALAKAAAAA 6.94700 6.79600 0.15100

37 LMAVVLASL 6.95400 7.49949 -0.54549

38 YVITTQHWL 6.98300 6.34128 0.64172

39 LLCLIFLLV 6.99600 7.51155 -0.51555

40 ITAQVPFSV 7.02000 6.66187 0.35813

41 YLEPGPVTL 7.05800 7.15089 -0.09289

42 YTDQVPFSV 7.06600 7.06902 -0.00302

43 NLYVSLLLL 7.11400 6.98499 0.12901

44 ILHNGAYSL 7.12700 7.35101 -0.22401

45 SIISAVVGI 7.15900 7.30206 -0.14306

46 VVMGTLVAL 7.17400 7.31300 -0.13900

47 YLEPGPVTI 7.18700 7.16812 0.01888

48 GLSRYVARL 7.24800 7.46125 -0.21325

49 LLAQFTSAI 7.30100 7.43224 -0.13124

50 VLLDYQGML 7.32800 7.59327 -0.26527

51 YLEPGPVTV 7.34200 7.40619 -0.06419

52 ILSPFMPLL 7.34700 7.14417 0.20283

53 YLSPGPVTA 7.38300 7.55250 -0.16950

54 IIDQVPFSV 7.39800 7.65171 -0.25371

55 SVYDFFVWL 7.44400 7.36647 0.07753

56 ITWQVPFSV 7.46300 7.43732 0.02568

57 ITYQVPFSV 7.48000 7.65730 -0.17730

58 GLYSSTVPV 7.48100 7.62509 -0.14409

59 VMGTLVALV 7.55300 7.23824 0.31476

60 LLLCLIFLL 7.58500 7.14965 0.43535

61 SLDDYNHLV 7.58500 7.17134 0.41366

62 VLIQRNPQL 7.64400 6.95001 0.69399

63 SLYADSPSV 7.65800 7.70585 -0.04785

64 ILSQVPFSV 7.69900 7.64726 0.05174

65 IMDQVPFSV 7.71900 8.03541 -0.31641

66 QLFEDNYAL 7.76400 7.47400 0.29000

67 ALMDKSLHV 7.77000 7.52279 0.24721

68 YAIDLPVSV 7.79600 7.59718 0.19882

69 FVWLHYYSV 7.82400 8.11885 -0.29485

70 MLGTHTMEV 7.84500 7.31608 0.52892

71 LLFGYPVYV 7.88600 8.03059 -0.14459

72 ILKEPVHGV 7.92100 7.59082 0.33018

73 YLMPGPVTV 7.93200 7.91444 0.01756

74 WLDQVPFSV 7.93900 7.95323 -0.01423

75 KTWGQYWQV 7.95500 7.68776 0.26724

76 ALMPLYACI 8.00000 7.44111 0.55889

77 YLAPGPVTA 8.03200 7.63311 0.39889

78 YLYPGPVTV 8.05100 8.31219 -0.26119

79 LLMGTLGIV 8.09700 7.67801 0.41899

80 YLWPGPVTV 8.12500 8.09221 0.03279

81 FLLTRILTI 8.14900 7.88232 0.26668

82 GLLGWSPQA 8.23700 8.20879 0.02821

83 ILYQVPFSV 8.31000 8.72330 -0.41330

84 GILTVILGV 8.34700 7.83642 0.51058

85 NMVPFFPPV 8.39800 8.08621 0.31179

86 ILDQVPFSV 8.48100 7.69073 0.79027

87 YLFPGPVTA 8.49500 8.34166 0.15334

88 YLDQVPFSV 8.63800 8.13502 0.50298

89 ILFQVPFSV 8.69900 8.43643 0.26257

90 ILWQVPFSV 8.77000 8.50332 0.26668

Correlation Coeffecient and Stand Error

CORL: 0.887105 CORL~2: 0.786955

RES: 0.366914 SEE: 0.038676

Statistical indices of Training calculation:

ITERATION (B): 34

No. Sequence Expt. Act Predicted Act Difference

1 VALVGLFVL 5.14800 5.74820 -0.60020

2 GTLVALVGL 5.34200 5.93096 -0.58896

3 LQTTIHDII 5.50100 5.80386 -0.30286

4 SLHVGTQCA 5.84200 6.14667 -0.30467

5 ALPYWNFAT 5.86900 6.64623 -0.77723

6 SLNFMGYVI 5.88100 5.96301 -0.08201

7 NLQSLTNLL 6.00000 6.69792 -0.69792

8 FVTWHRYHL 6.02500 5.72707 0.29793

9 DPKVKQWPL 6.17600 5.74036 0.43564

10 ITSQVPFSV 6.19600 6.58083 -0.38483

11 ALAKAAAAI 6.21100 6.23954 -0.02854

12 GLGQVPLIV 6.30100 6.56443 -0.26343

13 MLDLQPETT 6.33500 6.85420 -0.51920

14 LLSSNLSWL 6.34200 6.35005 -0.00805

15 GLACHQLCA 6.38000 6.04786 0.33214

16 LIGNESFAL 6.41500 7.05430 -0.63930

17 ALAKAAAAV 6.41900 6.47849 -0.05949

18 LLAVGATKV 6.47700 6.50864 -0.03164

19 ALAKAAAAL 6.51100 6.22225 0.28875

20 WILRGTSFV 6.55600 6.90774 -0.35174

21 IISCTCPTV 6.58000 6.66336 -0.08336

22 FLGGTPVCL 6.62300 6.87737 -0.25437

23 ALIHHNTHL 6.62300 6.79040 -0.16740

24 NLSWLSLDV 6.63900 6.04698 0.59202

25 YMIMVKCWM 6.66300 6.66134 0.00166

26 VLQAGFFLL 6.68200 7.04416 -0.36216

27 GTLGIVCPI 6.71400 6.51460 0.19940

28 VILGVLLLI 6.78500 7.47578 -0.69078

29 VTWHRYHLL 6.79300 6.55767 0.23533

30 PLLPIFFCL 6.79600 7.52651 -0.73051

31 TLGIVCPIC 6.81500 5.95545 0.85955

32 CLTSTVQLV 6.83200 7.10660 -0.27460

33 ILLLCLIFL 6.84500 6.79373 0.05127

34 FAFRDLCIV 6.88600 6.66206 0.22394

35 FLEPGPVTA 6.89800 7.48645 -0.58845

36 ALAKAAAAA 6.94700 6.79600 0.15100

37 LMAVVLASL 6.95400 7.49946 -0.54546

38 YVITTQHWL 6.98300 6.33912 0.64388

39 LLCLIFLLV 6.99600 7.51139 -0.51539

40 ITAQVPFSV 7.02000 6.66159 0.35841

41 YLEPGPVTL 7.05800 7.15019 -0.09219

42 YTDQVPFSV 7.06600 7.06946 -0.00346

43 NLYVSLLLL 7.11400 6.98366 0.13034

44 ILHNGAYSL 7.12700 7.35108 -0.22408

45 SIISAVVGI 7.15900 7.30236 -0.14336

46 VVMGTLVAL 7.17400 7.31346 -0.13946

47 YLEPGPVTI 7.18700 7.16748 0.01952

48 GLSRYVARL 7.24800 7.46179 -0.21379

49 LLAQFTSAI 7.30100 7.43258 -0.13158

50 VLLDYQGML 7.32800 7.59374 -0.26574

51 YLEPGPVTV 7.34200 7.40643 -0.06443

52 ILSPFMPLL 7.34700 7.14334 0.20366

53 YLSPGPVTA 7.38300 7.55359 -0.17059

54 IIDQVPFSV 7.39800 7.65276 -0.25476

55 SVYDFFVWL 7.44400 7.36658 0.07742

56 ITWQVPFSV 7.46300 7.43851 0.02449

57 ITYQVPFSV 7.48000 7.65890 -0.17890

58 GLYSSTVPV 7.48100 7.62620 -0.14520

59 VMGTLVALV 7.55300 7.23811 0.31489

60 LLLCLIFLL 7.58500 7.14832 0.43668

61 SLDDYNHLV 7.58500 7.17158 0.41342

62 VLIQRNPQL 7.64400 6.94919 0.69481

63 SLYADSPSV 7.65800 7.70724 -0.04924

64 ILSQVPFSV 7.69900 7.64829 0.05071

65 IMDQVPFSV 7.71900 8.03698 -0.31798

66 QLFEDNYAL 7.76400 7.47415 0.28985

67 ALMDKSLHV 7.77000 7.52375 0.24625

68 YAIDLPVSV 7.79600 7.59908 0.19692

69 FVWLHYYSV 7.82400 8.12037 -0.29637

70 MLGTHTMEV 7.84500 7.31637 0.52863

71 LLFGYPVYV 7.88600 8.03198 -0.14598

72 ILKEPVHGV 7.92100 7.59183 0.32917

73 YLMPGPVTV 7.93200 7.91564 0.01636

74 WLDQVPFSV 7.93900 7.95481 -0.01581

75 KTWGQYWQV 7.95500 7.68946 0.26554

76 ALMPLYACI 8.00000 7.44089 0.55911

77 YLAPGPVTA 8.03200 7.63435 0.39765

78 YLYPGPVTV 8.05100 8.31415 -0.26315

79 LLMGTLGIV 8.09700 7.67900 0.41800

80 YLWPGPVTV 8.12500 8.09375 0.03125

81 FLLTRILTI 8.14900 7.88325 0.26575

82 GLLGWSPQA 8.23700 8.21177 0.02523

83 ILYQVPFSV 8.31000 8.72636 -0.41636

84 GILTVILGV 8.34700 7.83790 0.50910

85 NMVPFFPPV 8.39800 8.08766 0.31034

86 ILDQVPFSV 8.48100 7.69184 0.78916

87 YLFPGPVTA 8.49500 8.34424 0.15076

88 YLDQVPFSV 8.63800 8.13692 0.50108

89 ILFQVPFSV 8.69900 8.43895 0.26005

90 ILWQVPFSV 8.77000 8.50597 0.26403

Correlation Coeffecient and Stand Error

CORL: 0.887107 CORL~2: 0.786959

RES: 0.366910 SEE: 0.038676

Statistical indices of Training calculation:

ITERATION (A): 35

No. Sequence Expt. Act Predicted Act Difference

1 VALVGLFVL 5.14800 5.75136 -0.60336

2 GTLVALVGL 5.34200 5.93359 -0.59159

3 LQTTIHDII 5.50100 5.80711 -0.30611

4 SLHVGTQCA 5.84200 6.14929 -0.30729

5 ALPYWNFAT 5.86900 6.64743 -0.77843

6 SLNFMGYVI 5.88100 5.96576 -0.08476

7 NLQSLTNLL 6.00000 6.69908 -0.69908

8 FVTWHRYHL 6.02500 5.73036 0.29464

9 DPKVKQWPL 6.17600 5.74322 0.43278

10 ITSQVPFSV 6.19600 6.58192 -0.38592

11 ALAKAAAAI 6.21100 6.24172 -0.03072

12 GLGQVPLIV 6.30100 6.56570 -0.26470

13 MLDLQPETT 6.33500 6.85509 -0.52009

14 LLSSNLSWL 6.34200 6.35203 -0.01003

15 GLACHQLCA 6.38000 6.05101 0.32899

16 LIGNESFAL 6.41500 7.05486 -0.63986

17 ALAKAAAAV 6.41900 6.48018 -0.06118

18 LLAVGATKV 6.47700 6.51023 -0.03323

19 ALAKAAAAL 6.51100 6.22451 0.28649

20 WILRGTSFV 6.55600 6.90849 -0.35249

21 IISCTCPTV 6.58000 6.66484 -0.08484

22 FLGGTPVCL 6.62300 6.87816 -0.25516

23 ALIHHNTHL 6.62300 6.79136 -0.16836

24 NLSWLSLDV 6.63900 6.04963 0.58937

25 YMIMVKCWM 6.66300 6.66256 0.00044

26 VLQAGFFLL 6.68200 7.04469 -0.36269

27 GTLGIVCPI 6.71400 6.51616 0.19784

28 VILGVLLLI 6.78500 7.47537 -0.69037

29 VTWHRYHLL 6.79300 6.55901 0.23399

30 PLLPIFFCL 6.79600 7.52629 -0.73029

31 TLGIVCPIC 6.81500 5.95814 0.85686

32 CLTSTVQLV 6.83200 7.10660 -0.27460

33 ILLLCLIFL 6.84500 6.79460 0.05040

34 FAFRDLCIV 6.88600 6.66310 0.22290

35 FLEPGPVTA 6.89800 7.48621 -0.58821

36 ALAKAAAAA 6.94700 6.79705 0.14995

37 LMAVVLASL 6.95400 7.49872 -0.54472

38 YVITTQHWL 6.98300 6.34125 0.64175

39 LLCLIFLLV 6.99600 7.51067 -0.51467

40 ITAQVPFSV 7.02000 6.66245 0.35755

41 YLEPGPVTL 7.05800 7.15066 -0.09266

42 YTDQVPFSV 7.06600 7.06947 -0.00347

43 NLYVSLLLL 7.11400 6.98428 0.12972

44 ILHNGAYSL 7.12700 7.35086 -0.22386

45 SIISAVVGI 7.15900 7.30230 -0.14330

46 VVMGTLVAL 7.17400 7.31329 -0.13929

47 YLEPGPVTI 7.18700 7.16788 0.01912

48 GLSRYVARL 7.24800 7.46131 -0.21331

49 LLAQFTSAI 7.30100 7.43206 -0.13106

50 VLLDYQGML 7.32800 7.59308 -0.26508

51 YLEPGPVTV 7.34200 7.40634 -0.06434

52 ILSPFMPLL 7.34700 7.14380 0.20320

53 YLSPGPVTA 7.38300 7.55325 -0.17025

54 IIDQVPFSV 7.39800 7.65181 -0.25381

55 SVYDFFVWL 7.44400 7.36637 0.07763

56 ITWQVPFSV 7.46300 7.43771 0.02529

57 ITYQVPFSV 7.48000 7.65765 -0.17765

58 GLYSSTVPV 7.48100 7.62555 -0.14455

59 VMGTLVALV 7.55300 7.23812 0.31488

60 LLLCLIFLL 7.58500 7.14886 0.43614

61 SLDDYNHLV 7.58500 7.17179 0.41321

62 VLIQRNPQL 7.64400 6.94977 0.69423

63 SLYADSPSV 7.65800 7.70626 -0.04826

64 ILSQVPFSV 7.69900 7.64726 0.05174

65 IMDQVPFSV 7.71900 8.03497 -0.31597

66 QLFEDNYAL 7.76400 7.47376 0.29024

67 ALMDKSLHV 7.77000 7.52299 0.24701

68 YAIDLPVSV 7.79600 7.59809 0.19791

69 FVWLHYYSV 7.82400 8.11851 -0.29451

70 MLGTHTMEV 7.84500 7.31625 0.52875

71 LLFGYPVYV 7.88600 8.03013 -0.14413

72 ILKEPVHGV 7.92100 7.59087 0.33013

73 YLMPGPVTV 7.93200 7.91440 0.01760

74 WLDQVPFSV 7.93900 7.95308 -0.01408

75 KTWGQYWQV 7.95500 7.68825 0.26675

76 ALMPLYACI 8.00000 7.44087 0.55913

77 YLAPGPVTA 8.03200 7.63378 0.39822

78 YLYPGPVTV 8.05100 8.31210 -0.26110

79 LLMGTLGIV 8.09700 7.67792 0.41908

80 YLWPGPVTV 8.12500 8.09217 0.03283

81 FLLTRILTI 8.14900 7.88209 0.26691

82 GLLGWSPQA 8.23700 8.20964 0.02736

83 ILYQVPFSV 8.31000 8.72299 -0.41299

84 GILTVILGV 8.34700 7.83686 0.51014

85 NMVPFFPPV 8.39800 8.08614 0.31186

86 ILDQVPFSV 8.48100 7.69071 0.79029

87 YLFPGPVTA 8.49500 8.34216 0.15284

88 YLDQVPFSV 8.63800 8.13481 0.50319

89 ILFQVPFSV 8.69900 8.43617 0.26283

90 ILWQVPFSV 8.77000 8.50305 0.26695

Correlation Coeffecient and Stand Error

CORL: 0.887109 CORL~2: 0.786963

RES: 0.366907 SEE: 0.038675

Statistical indices of Training calculation:

ITERATION (B): 35

No. Sequence Expt. Act Predicted Act Difference

1 VALVGLFVL 5.14800 5.74874 -0.60074

2 GTLVALVGL 5.34200 5.93147 -0.58947

3 LQTTIHDII 5.50100 5.80478 -0.30378

4 SLHVGTQCA 5.84200 6.14766 -0.30566

5 ALPYWNFAT 5.86900 6.64582 -0.77682

6 SLNFMGYVI 5.88100 5.96240 -0.08140

7 NLQSLTNLL 6.00000 6.69804 -0.69804

8 FVTWHRYHL 6.02500 5.72672 0.29828

9 DPKVKQWPL 6.17600 5.74038 0.43562

10 ITSQVPFSV 6.19600 6.58152 -0.38552

11 ALAKAAAAI 6.21100 6.23986 -0.02886

12 GLGQVPLIV 6.30100 6.56448 -0.26348

13 MLDLQPETT 6.33500 6.85444 -0.51944

14 LLSSNLSWL 6.34200 6.35007 -0.00807

15 GLACHQLCA 6.38000 6.04886 0.33114

16 LIGNESFAL 6.41500 7.05445 -0.63945

17 ALAKAAAAV 6.41900 6.47911 -0.06011

18 LLAVGATKV 6.47700 6.50889 -0.03189

19 ALAKAAAAL 6.51100 6.22259 0.28841

20 WILRGTSFV 6.55600 6.90779 -0.35179

21 IISCTCPTV 6.58000 6.66349 -0.08349

22 FLGGTPVCL 6.62300 6.87721 -0.25421

23 ALIHHNTHL 6.62300 6.79043 -0.16743

24 NLSWLSLDV 6.63900 6.04696 0.59204

25 YMIMVKCWM 6.66300 6.65970 0.00330

26 VLQAGFFLL 6.68200 7.04389 -0.36189

27 GTLGIVCPI 6.71400 6.51537 0.19863

28 VILGVLLLI 6.78500 7.47552 -0.69052

29 VTWHRYHLL 6.79300 6.55785 0.23515

30 PLLPIFFCL 6.79600 7.52609 -0.73009

31 TLGIVCPIC 6.81500 5.95497 0.86003

32 CLTSTVQLV 6.83200 7.10655 -0.27455

33 ILLLCLIFL 6.84500 6.79267 0.05233

34 FAFRDLCIV 6.88600 6.66266 0.22334

35 FLEPGPVTA 6.89800 7.48711 -0.58911

36 ALAKAAAAA 6.94700 6.79705 0.14995

37 LMAVVLASL 6.95400 7.49870 -0.54470

38 YVITTQHWL 6.98300 6.33928 0.64372

39 LLCLIFLLV 6.99600 7.51053 -0.51453

40 ITAQVPFSV 7.02000 6.66219 0.35781

41 YLEPGPVTL 7.05800 7.15003 -0.09203

42 YTDQVPFSV 7.06600 7.06987 -0.00387

43 NLYVSLLLL 7.11400 6.98307 0.13093

44 ILHNGAYSL 7.12700 7.35093 -0.22393

45 SIISAVVGI 7.15900 7.30258 -0.14358

46 VVMGTLVAL 7.17400 7.31370 -0.13970

47 YLEPGPVTI 7.18700 7.16730 0.01970

48 GLSRYVARL 7.24800 7.46180 -0.21380

49 LLAQFTSAI 7.30100 7.43238 -0.13138

50 VLLDYQGML 7.32800 7.59351 -0.26551

51 YLEPGPVTV 7.34200 7.40656 -0.06456

52 ILSPFMPLL 7.34700 7.14305 0.20395

53 YLSPGPVTA 7.38300 7.55424 -0.17124

54 IIDQVPFSV 7.39800 7.65276 -0.25476

55 SVYDFFVWL 7.44400 7.36647 0.07753

56 ITWQVPFSV 7.46300 7.43879 0.02421

57 ITYQVPFSV 7.48000 7.65910 -0.17910

58 GLYSSTVPV 7.48100 7.62656 -0.14556

59 VMGTLVALV 7.55300 7.23800 0.31500

60 LLLCLIFLL 7.58500 7.14765 0.43735

61 SLDDYNHLV 7.58500 7.17200 0.41300

62 VLIQRNPQL 7.64400 6.94902 0.69498

63 SLYADSPSV 7.65800 7.70753 -0.04953

64 ILSQVPFSV 7.69900 7.64819 0.05081

65 IMDQVPFSV 7.71900 8.03641 -0.31741

66 QLFEDNYAL 7.76400 7.47389 0.29011

67 ALMDKSLHV 7.77000 7.52386 0.24614

68 YAIDLPVSV 7.79600 7.59981 0.19619

69 FVWLHYYSV 7.82400 8.11989 -0.29589

70 MLGTHTMEV 7.84500 7.31651 0.52849

71 LLFGYPVYV 7.88600 8.03140 -0.14540

72 ILKEPVHGV 7.92100 7.59179 0.32921

73 YLMPGPVTV 7.93200 7.91549 0.01651

74 WLDQVPFSV 7.93900 7.95451 -0.01551

75 KTWGQYWQV 7.95500 7.68980 0.26520

76 ALMPLYACI 8.00000 7.44067 0.55933

77 YLAPGPVTA 8.03200 7.63491 0.39709

78 YLYPGPVTV 8.05100 8.31389 -0.26289

79 LLMGTLGIV 8.09700 7.67882 0.41818

80 YLWPGPVTV 8.12500 8.09357 0.03143

81 FLLTRILTI 8.14900 7.88293 0.26607

82 GLLGWSPQA 8.23700 8.21235 0.02465

83 ILYQVPFSV 8.31000 8.72578 -0.41578

84 GILTVILGV 8.34700 7.83821 0.50879

85 NMVPFFPPV 8.39800 8.08746 0.31054

86 ILDQVPFSV 8.48100 7.69172 0.78928

87 YLFPGPVTA 8.49500 8.34451 0.15049

88 YLDQVPFSV 8.63800 8.13654 0.50146

89 ILFQVPFSV 8.69900 8.43847 0.26053

90 ILWQVPFSV 8.77000 8.50546 0.26454

Correlation Coeffecient and Stand Error

CORL: 0.887111 CORL~2: 0.786966

RES: 0.366904 SEE: 0.038675

Statistical indices of Training calculation:

ITERATION (A): 36

No. Sequence Expt. Act Predicted Act Difference

1 VALVGLFVL 5.14800 5.75162 -0.60362

2 GTLVALVGL 5.34200 5.93387 -0.59187

3 LQTTIHDII 5.50100 5.80773 -0.30673

4 SLHVGTQCA 5.84200 6.15004 -0.30804

5 ALPYWNFAT 5.86900 6.64692 -0.77792

6 SLNFMGYVI 5.88100 5.96491 -0.08391

7 NLQSLTNLL 6.00000 6.69909 -0.69909

8 FVTWHRYHL 6.02500 5.72972 0.29528

9 DPKVKQWPL 6.17600 5.74299 0.43301

10 ITSQVPFSV 6.19600 6.58251 -0.38651

11 ALAKAAAAI 6.21100 6.24185 -0.03085

12 GLGQVPLIV 6.30100 6.56564 -0.26464

13 MLDLQPETT 6.33500 6.85525 -0.52025

14 LLSSNLSWL 6.34200 6.35187 -0.00987

15 GLACHQLCA 6.38000 6.05173 0.32827

16 LIGNESFAL 6.41500 7.05495 -0.63995

17 ALAKAAAAV 6.41900 6.48066 -0.06166

18 LLAVGATKV 6.47700 6.51033 -0.03333

19 ALAKAAAAL 6.51100 6.22465 0.28635

20 WILRGTSFV 6.55600 6.90848 -0.35248

21 IISCTCPTV 6.58000 6.66484 -0.08484

22 FLGGTPVCL 6.62300 6.87793 -0.25493

23 ALIHHNTHL 6.62300 6.79131 -0.16831

24 NLSWLSLDV 6.63900 6.04937 0.58963

25 YMIMVKCWM 6.66300 6.66082 0.00218

26 VLQAGFFLL 6.68200 7.04438 -0.36238

27 GTLGIVCPI 6.71400 6.51678 0.19722

28 VILGVLLLI 6.78500 7.47515 -0.69015

29 VTWHRYHLL 6.79300 6.55907 0.23393

30 PLLPIFFCL 6.79600 7.52589 -0.72989

31 TLGIVCPIC 6.81500 5.95741 0.85759

32 CLTSTVQLV 6.83200 7.10656 -0.27456

33 ILLLCLIFL 6.84500 6.79347 0.05153

34 FAFRDLCIV 6.88600 6.66361 0.22239

35 FLEPGPVTA 6.89800 7.48689 -0.58889

36 ALAKAAAAA 6.94700 6.79801 0.14899

37 LMAVVLASL 6.95400 7.49803 -0.54403

38 YVITTQHWL 6.98300 6.34123 0.64177

39 LLCLIFLLV 6.99600 7.50988 -0.51388

40 ITAQVPFSV 7.02000 6.66298 0.35702

41 YLEPGPVTL 7.05800 7.15046 -0.09246

42 YTDQVPFSV 7.06600 7.06988 -0.00388

43 NLYVSLLLL 7.11400 6.98364 0.13036

44 ILHNGAYSL 7.12700 7.35073 -0.22373

45 SIISAVVGI 7.15900 7.30252 -0.14352

46 VVMGTLVAL 7.17400 7.31355 -0.13955

47 YLEPGPVTI 7.18700 7.16766 0.01934

48 GLSRYVARL 7.24800 7.46137 -0.21337

49 LLAQFTSAI 7.30100 7.43191 -0.13091

50 VLLDYQGML 7.32800 7.59291 -0.26491

51 YLEPGPVTV 7.34200 7.40647 -0.06447

52 ILSPFMPLL 7.34700 7.14347 0.20353

53 YLSPGPVTA 7.38300 7.55393 -0.17093

54 IIDQVPFSV 7.39800 7.65189 -0.25389

55 SVYDFFVWL 7.44400 7.36628 0.07772

56 ITWQVPFSV 7.46300 7.43806 0.02494

57 ITYQVPFSV 7.48000 7.65796 -0.17796

58 GLYSSTVPV 7.48100 7.62597 -0.14497

59 VMGTLVALV 7.55300 7.23802 0.31498

60 LLLCLIFLL 7.58500 7.14814 0.43686

61 SLDDYNHLV 7.58500 7.17219 0.41281

62 VLIQRNPQL 7.64400 6.94955 0.69445

63 SLYADSPSV 7.65800 7.70664 -0.04864

64 ILSQVPFSV 7.69900 7.64726 0.05174

65 IMDQVPFSV 7.71900 8.03458 -0.31558

66 QLFEDNYAL 7.76400 7.47354 0.29046

67 ALMDKSLHV 7.77000 7.52317 0.24683

68 YAIDLPVSV 7.79600 7.59891 0.19709

69 FVWLHYYSV 7.82400 8.11820 -0.29420

70 MLGTHTMEV 7.84500 7.31640 0.52860

71 LLFGYPVYV 7.88600 8.02971 -0.14371

72 ILKEPVHGV 7.92100 7.59093 0.33007

73 YLMPGPVTV 7.93200 7.91436 0.01764

74 WLDQVPFSV 7.93900 7.95294 -0.01394

75 KTWGQYWQV 7.95500 7.68871 0.26629

76 ALMPLYACI 8.00000 7.44065 0.55935

77 YLAPGPVTA 8.03200 7.63439 0.39761

78 YLYPGPVTV 8.05100 8.31202 -0.26102

79 LLMGTLGIV 8.09700 7.67783 0.41917

80 YLWPGPVTV 8.12500 8.09212 0.03288

81 FLLTRILTI 8.14900 7.88188 0.26712

82 GLLGWSPQA 8.23700 8.21041 0.02659

83 ILYQVPFSV 8.31000 8.72271 -0.41271

84 GILTVILGV 8.34700 7.83726 0.50974

85 NMVPFFPPV 8.39800 8.08608 0.31192

86 ILDQVPFSV 8.48100 7.69069 0.79031

87 YLFPGPVTA 8.49500 8.34261 0.15239

88 YLDQVPFSV 8.63800 8.13463 0.50337

89 ILFQVPFSV 8.69900 8.43594 0.26306

90 ILWQVPFSV 8.77000 8.50281 0.26719

Correlation Coeffecient and Stand Error

CORL: 0.887113 CORL~2: 0.786970

RES: 0.366902 SEE: 0.038675

Statistical indices of Training calculation:

ITERATION (B): 36

No. Sequence Expt. Act Predicted Act Difference

1 VALVGLFVL 5.14800 5.74922 -0.60122

2 GTLVALVGL 5.34200 5.93194 -0.58994

3 LQTTIHDII 5.50100 5.80561 -0.30461

4 SLHVGTQCA 5.84200 6.14855 -0.30655

5 ALPYWNFAT 5.86900 6.64545 -0.77645

6 SLNFMGYVI 5.88100 5.96185 -0.08085

7 NLQSLTNLL 6.00000 6.69814 -0.69814

8 FVTWHRYHL 6.02500 5.72640 0.29860

9 DPKVKQWPL 6.17600 5.74040 0.43560

10 ITSQVPFSV 6.19600 6.58215 -0.38615

11 ALAKAAAAI 6.21100 6.24015 -0.02915

12 GLGQVPLIV 6.30100 6.56453 -0.26353

13 MLDLQPETT 6.33500 6.85466 -0.51966

14 LLSSNLSWL 6.34200 6.35008 -0.00808

15 GLACHQLCA 6.38000 6.04978 0.33022

16 LIGNESFAL 6.41500 7.05458 -0.63958

17 ALAKAAAAV 6.41900 6.47968 -0.06068

18 LLAVGATKV 6.47700 6.50912 -0.03212

19 ALAKAAAAL 6.51100 6.22289 0.28811

20 WILRGTSFV 6.55600 6.90784 -0.35184

21 IISCTCPTV 6.58000 6.66362 -0.08362

22 FLGGTPVCL 6.62300 6.87706 -0.25406

23 ALIHHNTHL 6.62300 6.79046 -0.16746

24 NLSWLSLDV 6.63900 6.04694 0.59206

25 YMIMVKCWM 6.66300 6.65822 0.00478

26 VLQAGFFLL 6.68200 7.04365 -0.36165

27 GTLGIVCPI 6.71400 6.51607 0.19793

28 VILGVLLLI 6.78500 7.47528 -0.69028

29 VTWHRYHLL 6.79300 6.55801 0.23499

30 PLLPIFFCL 6.79600 7.52571 -0.72971

31 TLGIVCPIC 6.81500 5.95452 0.86048

32 CLTSTVQLV 6.83200 7.10651 -0.27451

33 ILLLCLIFL 6.84500 6.79171 0.05329

34 FAFRDLCIV 6.88600 6.66320 0.22280

35 FLEPGPVTA 6.89800 7.48771 -0.58971

36 ALAKAAAAA 6.94700 6.79800 0.14900

37 LMAVVLASL 6.95400 7.49801 -0.54401

38 YVITTQHWL 6.98300 6.33943 0.64357

39 LLCLIFLLV 6.99600 7.50975 -0.51375

40 ITAQVPFSV 7.02000 6.66274 0.35726

41 YLEPGPVTL 7.05800 7.14988 -0.09188

42 YTDQVPFSV 7.06600 7.07024 -0.00424

43 NLYVSLLLL 7.11400 6.98254 0.13146

44 ILHNGAYSL 7.12700 7.35079 -0.22379

45 SIISAVVGI 7.15900 7.30277 -0.14377

46 VVMGTLVAL 7.17400 7.31392 -0.13992

47 YLEPGPVTI 7.18700 7.16714 0.01986

48 GLSRYVARL 7.24800 7.46181 -0.21381

49 LLAQFTSAI 7.30100 7.43219 -0.13119

50 VLLDYQGML 7.32800 7.59330 -0.26530

51 YLEPGPVTV 7.34200 7.40667 -0.06467

52 ILSPFMPLL 7.34700 7.14279 0.20421

53 YLSPGPVTA 7.38300 7.55483 -0.17183

54 IIDQVPFSV 7.39800 7.65276 -0.25476

55 SVYDFFVWL 7.44400 7.36637 0.07763

56 ITWQVPFSV 7.46300 7.43904 0.02396

57 ITYQVPFSV 7.48000 7.65929 -0.17929

58 GLYSSTVPV 7.48100 7.62689 -0.14589

59 VMGTLVALV 7.55300 7.23791 0.31509

60 LLLCLIFLL 7.58500 7.14704 0.43796

61 SLDDYNHLV 7.58500 7.17238 0.41262

62 VLIQRNPQL 7.64400 6.94887 0.69513

63 SLYADSPSV 7.65800 7.70779 -0.04979

64 ILSQVPFSV 7.69900 7.64811 0.05089

65 IMDQVPFSV 7.71900 8.03589 -0.31689

66 QLFEDNYAL 7.76400 7.47366 0.29034

67 ALMDKSLHV 7.77000 7.52396 0.24604

68 YAIDLPVSV 7.79600 7.60048 0.19552

69 FVWLHYYSV 7.82400 8.11946 -0.29546

70 MLGTHTMEV 7.84500 7.31664 0.52836

71 LLFGYPVYV 7.88600 8.03087 -0.14487

72 ILKEPVHGV 7.92100 7.59176 0.32924

73 YLMPGPVTV 7.93200 7.91536 0.01664

74 WLDQVPFSV 7.93900 7.95425 -0.01525

75 KTWGQYWQV 7.95500 7.69012 0.26488

76 ALMPLYACI 8.00000 7.44047 0.55953

77 YLAPGPVTA 8.03200 7.63542 0.39658

78 YLYPGPVTV 8.05100 8.31365 -0.26265

79 LLMGTLGIV 8.09700 7.67866 0.41834

80 YLWPGPVTV 8.12500 8.09340 0.03160

81 FLLTRILTI 8.14900 7.88264 0.26636

82 GLLGWSPQA 8.23700 8.21288 0.02412

83 ILYQVPFSV 8.31000 8.72525 -0.41525

84 GILTVILGV 8.34700 7.83849 0.50851

85 NMVPFFPPV 8.39800 8.08729 0.31071

86 ILDQVPFSV 8.48100 7.69160 0.78940

87 YLFPGPVTA 8.49500 8.34475 0.15025

88 YLDQVPFSV 8.63800 8.13620 0.50180

89 ILFQVPFSV 8.69900 8.43803 0.26097

90 ILWQVPFSV 8.77000 8.50500 0.26500

Correlation Coeffecient and Stand Error

CORL: 0.887114 CORL~2: 0.786972

RES: 0.366899 SEE: 0.038675

Statistical indices of Training calculation:

ITERATION (A): 37

No. Sequence Expt. Act Predicted Act Difference

1 VALVGLFVL 5.14800 5.75185 -0.60385

2 GTLVALVGL 5.34200 5.93412 -0.59212

3 LQTTIHDII 5.50100 5.80830 -0.30730

4 SLHVGTQCA 5.84200 6.15073 -0.30873

5 ALPYWNFAT 5.86900 6.64646 -0.77746

6 SLNFMGYVI 5.88100 5.96414 -0.08314

7 NLQSLTNLL 6.00000 6.69910 -0.69910

8 FVTWHRYHL 6.02500 5.72913 0.29587

9 DPKVKQWPL 6.17600 5.74278 0.43322

10 ITSQVPFSV 6.19600 6.58305 -0.38705

11 ALAKAAAAI 6.21100 6.24197 -0.03097

12 GLGQVPLIV 6.30100 6.56558 -0.26458

13 MLDLQPETT 6.33500 6.85540 -0.52040

14 LLSSNLSWL 6.34200 6.35172 -0.00972

15 GLACHQLCA 6.38000 6.05239 0.32761

16 LIGNESFAL 6.41500 7.05503 -0.64003

17 ALAKAAAAV 6.41900 6.48109 -0.06209

18 LLAVGATKV 6.47700 6.51043 -0.03343

19 ALAKAAAAL 6.51100 6.22477 0.28623

20 WILRGTSFV 6.55600 6.90847 -0.35247

21 IISCTCPTV 6.58000 6.66484 -0.08484

22 FLGGTPVCL 6.62300 6.87773 -0.25473

23 ALIHHNTHL 6.62300 6.79125 -0.16825

24 NLSWLSLDV 6.63900 6.04914 0.58986

25 YMIMVKCWM 6.66300 6.65924 0.00376

26 VLQAGFFLL 6.68200 7.04410 -0.36210

27 GTLGIVCPI 6.71400 6.51735 0.19665

28 VILGVLLLI 6.78500 7.47494 -0.68994

29 VTWHRYHLL 6.79300 6.55912 0.23388

30 PLLPIFFCL 6.79600 7.52552 -0.72952

31 TLGIVCPIC 6.81500 5.95676 0.85824

32 CLTSTVQLV 6.83200 7.10652 -0.27452

33 ILLLCLIFL 6.84500 6.79243 0.05257

34 FAFRDLCIV 6.88600 6.66406 0.22194

35 FLEPGPVTA 6.89800 7.48751 -0.58951

36 ALAKAAAAA 6.94700 6.79888 0.14812

37 LMAVVLASL 6.95400 7.49740 -0.54340

38 YVITTQHWL 6.98300 6.34120 0.64180

39 LLCLIFLLV 6.99600 7.50915 -0.51315

40 ITAQVPFSV 7.02000 6.66346 0.35654

41 YLEPGPVTL 7.05800 7.15027 -0.09227

42 YTDQVPFSV 7.06600 7.07025 -0.00425

43 NLYVSLLLL 7.11400 6.98306 0.13094

44 ILHNGAYSL 7.12700 7.35061 -0.22361

45 SIISAVVGI 7.15900 7.30272 -0.14372

46 VVMGTLVAL 7.17400 7.31378 -0.13978

47 YLEPGPVTI 7.18700 7.16747 0.01953

48 GLSRYVARL 7.24800 7.46142 -0.21342

49 LLAQFTSAI 7.30100 7.43176 -0.13076

50 VLLDYQGML 7.32800 7.59276 -0.26476

51 YLEPGPVTV 7.34200 7.40659 -0.06459

52 ILSPFMPLL 7.34700 7.14317 0.20383

53 YLSPGPVTA 7.38300 7.55454 -0.17154

54 IIDQVPFSV 7.39800 7.65197 -0.25397

55 SVYDFFVWL 7.44400 7.36620 0.07780

56 ITWQVPFSV 7.46300 7.43838 0.02462

57 ITYQVPFSV 7.48000 7.65825 -0.17825

58 GLYSSTVPV 7.48100 7.62635 -0.14535

59 VMGTLVALV 7.55300 7.23792 0.31508

60 LLLCLIFLL 7.58500 7.14748 0.43752

61 SLDDYNHLV 7.58500 7.17256 0.41244

62 VLIQRNPQL 7.64400 6.94936 0.69464

63 SLYADSPSV 7.65800 7.70698 -0.04898

64 ILSQVPFSV 7.69900 7.64726 0.05174

65 IMDQVPFSV 7.71900 8.03422 -0.31522

66 QLFEDNYAL 7.76400 7.47334 0.29066

67 ALMDKSLHV 7.77000 7.52333 0.24667

68 YAIDLPVSV 7.79600 7.59966 0.19634

69 FVWLHYYSV 7.82400 8.11792 -0.29392

70 MLGTHTMEV 7.84500 7.31654 0.52846

71 LLFGYPVYV 7.88600 8.02933 -0.14333

72 ILKEPVHGV 7.92100 7.59097 0.33003

73 YLMPGPVTV 7.93200 7.91433 0.01767

74 WLDQVPFSV 7.93900 7.95281 -0.01381

75 KTWGQYWQV 7.95500 7.68912 0.26588

76 ALMPLYACI 8.00000 7.44045 0.55955

77 YLAPGPVTA 8.03200 7.63495 0.39705

78 YLYPGPVTV 8.05100 8.31195 -0.26095

79 LLMGTLGIV 8.09700 7.67775 0.41925

80 YLWPGPVTV 8.12500 8.09209 0.03291

81 FLLTRILTI 8.14900 7.88168 0.26732

82 GLLGWSPQA 8.23700 8.21111 0.02589

83 ILYQVPFSV 8.31000 8.72245 -0.41245

84 GILTVILGV 8.34700 7.83763 0.50937

85 NMVPFFPPV 8.39800 8.08603 0.31197

86 ILDQVPFSV 8.48100 7.69067 0.79033

87 YLFPGPVTA 8.49500 8.34302 0.15198

88 YLDQVPFSV 8.63800 8.13445 0.50355

89 ILFQVPFSV 8.69900 8.43573 0.26327

90 ILWQVPFSV 8.77000 8.50259 0.26741

Correlation Coeffecient and Stand Error

CORL: 0.887116 CORL~2: 0.786975

RES: 0.366897 SEE: 0.038674

Statistical indices of Training calculation:

ITERATION (B): 37

No. Sequence Expt. Act Predicted Act Difference

1 VALVGLFVL 5.14800 5.74967 -0.60167

2 GTLVALVGL 5.34200 5.93236 -0.59036

3 LQTTIHDII 5.50100 5.80636 -0.30536

4 SLHVGTQCA 5.84200 6.14937 -0.30737

5 ALPYWNFAT 5.86900 6.64512 -0.77612

6 SLNFMGYVI 5.88100 5.96135 -0.08035

7 NLQSLTNLL 6.00000 6.69823 -0.69823

8 FVTWHRYHL 6.02500 5.72610 0.29890

9 DPKVKQWPL 6.17600 5.74042 0.43558

10 ITSQVPFSV 6.19600 6.58272 -0.38672

11 ALAKAAAAI 6.21100 6.24042 -0.02942

12 GLGQVPLIV 6.30100 6.56457 -0.26357

13 MLDLQPETT 6.33500 6.85487 -0.51987

14 LLSSNLSWL 6.34200 6.35009 -0.00809

15 GLACHQLCA 6.38000 6.05061 0.32939

16 LIGNESFAL 6.41500 7.05469 -0.63969

17 ALAKAAAAV 6.41900 6.48020 -0.06120

18 LLAVGATKV 6.47700 6.50932 -0.03232

19 ALAKAAAAL 6.51100 6.22318 0.28782

20 WILRGTSFV 6.55600 6.90789 -0.35189

21 IISCTCPTV 6.58000 6.66373 -0.08373

22 FLGGTPVCL 6.62300 6.87694 -0.25394

23 ALIHHNTHL 6.62300 6.79048 -0.16748

24 NLSWLSLDV 6.63900 6.04692 0.59208

25 YMIMVKCWM 6.66300 6.65687 0.00613

26 VLQAGFFLL 6.68200 7.04344 -0.36144

27 GTLGIVCPI 6.71400 6.51670 0.19730

28 VILGVLLLI 6.78500 7.47506 -0.69006

29 VTWHRYHLL 6.79300 6.55816 0.23484

30 PLLPIFFCL 6.79600 7.52536 -0.72936

31 TLGIVCPIC 6.81500 5.95412 0.86088

32 CLTSTVQLV 6.83200 7.10648 -0.27448

33 ILLLCLIFL 6.84500 6.79083 0.05417

34 FAFRDLCIV 6.88600 6.66369 0.22231

35 FLEPGPVTA 6.89800 7.48825 -0.59025

36 ALAKAAAAA 6.94700 6.79887 0.14813

37 LMAVVLASL 6.95400 7.49738 -0.54338

38 YVITTQHWL 6.98300 6.33957 0.64343

39 LLCLIFLLV 6.99600 7.50904 -0.51304

40 ITAQVPFSV 7.02000 6.66324 0.35676

41 YLEPGPVTL 7.05800 7.14975 -0.09175

42 YTDQVPFSV 7.06600 7.07058 -0.00458

43 NLYVSLLLL 7.11400 6.98205 0.13195

44 ILHNGAYSL 7.12700 7.35066 -0.22366

45 SIISAVVGI 7.15900 7.30295 -0.14395

46 VVMGTLVAL 7.17400 7.31412 -0.14012

47 YLEPGPVTI 7.18700 7.16699 0.02001

48 GLSRYVARL 7.24800 7.46182 -0.21382

49 LLAQFTSAI 7.30100 7.43202 -0.13102

50 VLLDYQGML 7.32800 7.59311 -0.26511

51 YLEPGPVTV 7.34200 7.40678 -0.06478

52 ILSPFMPLL 7.34700 7.14255 0.20445

53 YLSPGPVTA 7.38300 7.55537 -0.17237

54 IIDQVPFSV 7.39800 7.65276 -0.25476

55 SVYDFFVWL 7.44400 7.36628 0.07772

56 ITWQVPFSV 7.46300 7.43928 0.02372

57 ITYQVPFSV 7.48000 7.65946 -0.17946

58 GLYSSTVPV 7.48100 7.62719 -0.14619

59 VMGTLVALV 7.55300 7.23782 0.31518

60 LLLCLIFLL 7.58500 7.14648 0.43852

61 SLDDYNHLV 7.58500 7.17273 0.41227

62 VLIQRNPQL 7.64400 6.94873 0.69527

63 SLYADSPSV 7.65800 7.70803 -0.05003

64 ILSQVPFSV 7.69900 7.64803 0.05097

65 IMDQVPFSV 7.71900 8.03541 -0.31641

66 QLFEDNYAL 7.76400 7.47345 0.29055

67 ALMDKSLHV 7.77000 7.52406 0.24594

68 YAIDLPVSV 7.79600 7.60109 0.19491

69 FVWLHYYSV 7.82400 8.11907 -0.29507

70 MLGTHTMEV 7.84500 7.31676 0.52824

71 LLFGYPVYV 7.88600 8.03039 -0.14439

72 ILKEPVHGV 7.92100 7.59174 0.32926

73 YLMPGPVTV 7.93200 7.91524 0.01676

74 WLDQVPFSV 7.93900 7.95400 -0.01500

75 KTWGQYWQV 7.95500 7.69040 0.26460

76 ALMPLYACI 8.00000 7.44028 0.55972

77 YLAPGPVTA 8.03200 7.63589 0.39611

78 YLYPGPVTV 8.05100 8.31343 -0.26243

79 LLMGTLGIV 8.09700 7.67851 0.41849

80 YLWPGPVTV 8.12500 8.09325 0.03175

81 FLLTRILTI 8.14900 7.88238 0.26662

82 GLLGWSPQA 8.23700 8.21336 0.02364

83 ILYQVPFSV 8.31000 8.72476 -0.41476

84 GILTVILGV 8.34700 7.83875 0.50825

85 NMVPFFPPV 8.39800 8.08712 0.31088

86 ILDQVPFSV 8.48100 7.69150 0.78950

87 YLFPGPVTA 8.49500 8.34497 0.15003

88 YLDQVPFSV 8.63800 8.13589 0.50211

89 ILFQVPFSV 8.69900 8.43763 0.26137

90 ILWQVPFSV 8.77000 8.50459 0.26541

Correlation Coeffecient and Stand Error

CORL: 0.887117 CORL~2: 0.786976

RES: 0.366895 SEE: 0.038674

Statistical indices of Training calculation:

ITERATION (A): 38

No. Sequence Expt. Act Predicted Act Difference

1 VALVGLFVL 5.14800 5.75206 -0.60406

2 GTLVALVGL 5.34200 5.93435 -0.59235

3 LQTTIHDII 5.50100 5.80881 -0.30781

4 SLHVGTQCA 5.84200 6.15135 -0.30935

5 ALPYWNFAT 5.86900 6.64603 -0.77703

6 SLNFMGYVI 5.88100 5.96344 -0.08244

7 NLQSLTNLL 6.00000 6.69911 -0.69911

8 FVTWHRYHL 6.02500 5.72860 0.29640

9 DPKVKQWPL 6.17600 5.74259 0.43341

10 ITSQVPFSV 6.19600 6.58355 -0.38755

11 ALAKAAAAI 6.21100 6.24207 -0.03107

12 GLGQVPLIV 6.30100 6.56554 -0.26454

13 MLDLQPETT 6.33500 6.85554 -0.52054

14 LLSSNLSWL 6.34200 6.35159 -0.00959

15 GLACHQLCA 6.38000 6.05299 0.32701

16 LIGNESFAL 6.41500 7.05511 -0.64011

17 ALAKAAAAV 6.41900 6.48149 -0.06249

18 LLAVGATKV 6.47700 6.51052 -0.03352

19 ALAKAAAAL 6.51100 6.22489 0.28611

20 WILRGTSFV 6.55600 6.90846 -0.35246

21 IISCTCPTV 6.58000 6.66485 -0.08485

22 FLGGTPVCL 6.62300 6.87754 -0.25454

23 ALIHHNTHL 6.62300 6.79121 -0.16821

24 NLSWLSLDV 6.63900 6.04892 0.59008

25 YMIMVKCWM 6.66300 6.65780 0.00520

26 VLQAGFFLL 6.68200 7.04384 -0.36184

27 GTLGIVCPI 6.71400 6.51787 0.19613

28 VILGVLLLI 6.78500 7.47475 -0.68975

29 VTWHRYHLL 6.79300 6.55917 0.23383

30 PLLPIFFCL 6.79600 7.52519 -0.72919

31 TLGIVCPIC 6.81500 5.95616 0.85884

32 CLTSTVQLV 6.83200 7.10648 -0.27448

33 ILLLCLIFL 6.84500 6.79149 0.05351

34 FAFRDLCIV 6.88600 6.66448 0.22152

35 FLEPGPVTA 6.89800 7.48807 -0.59007

36 ALAKAAAAA 6.94700 6.79967 0.14733

37 LMAVVLASL 6.95400 7.49682 -0.54282

38 YVITTQHWL 6.98300 6.34118 0.64182

39 LLCLIFLLV 6.99600 7.50849 -0.51249

40 ITAQVPFSV 7.02000 6.66389 0.35611

41 YLEPGPVTL 7.05800 7.15010 -0.09210

42 YTDQVPFSV 7.06600 7.07059 -0.00459

43 NLYVSLLLL 7.11400 6.98253 0.13147

44 ILHNGAYSL 7.12700 7.35050 -0.22350

45 SIISAVVGI 7.15900 7.30290 -0.14390

46 VVMGTLVAL 7.17400 7.31399 -0.13999

47 YLEPGPVTI 7.18700 7.16729 0.01971

48 GLSRYVARL 7.24800 7.46146 -0.21346

49 LLAQFTSAI 7.30100 7.43163 -0.13063

50 VLLDYQGML 7.32800 7.59262 -0.26462

51 YLEPGPVTV 7.34200 7.40670 -0.06470

52 ILSPFMPLL 7.34700 7.14290 0.20410

53 YLSPGPVTA 7.38300 7.55511 -0.17211

54 IIDQVPFSV 7.39800 7.65204 -0.25404

55 SVYDFFVWL 7.44400 7.36612 0.07788

56 ITWQVPFSV 7.46300 7.43868 0.02432

57 ITYQVPFSV 7.48000 7.65851 -0.17851

58 GLYSSTVPV 7.48100 7.62670 -0.14570

59 VMGTLVALV 7.55300 7.23783 0.31517

60 LLLCLIFLL 7.58500 7.14689 0.43811

61 SLDDYNHLV 7.58500 7.17289 0.41211

62 VLIQRNPQL 7.64400 6.94918 0.69482

63 SLYADSPSV 7.65800 7.70729 -0.04929

64 ILSQVPFSV 7.69900 7.64725 0.05175

65 IMDQVPFSV 7.71900 8.03390 -0.31490

66 QLFEDNYAL 7.76400 7.47316 0.29084

67 ALMDKSLHV 7.77000 7.52348 0.24652

68 YAIDLPVSV 7.79600 7.60034 0.19566

69 FVWLHYYSV 7.82400 8.11766 -0.29366

70 MLGTHTMEV 7.84500 7.31667 0.52833

71 LLFGYPVYV 7.88600 8.02899 -0.14299

72 ILKEPVHGV 7.92100 7.59102 0.32998

73 YLMPGPVTV 7.93200 7.91429 0.01771

74 WLDQVPFSV 7.93900 7.95270 -0.01370

75 KTWGQYWQV 7.95500 7.68949 0.26551

76 ALMPLYACI 8.00000 7.44027 0.55973

77 YLAPGPVTA 8.03200 7.63545 0.39655

78 YLYPGPVTV 8.05100 8.31188 -0.26088

79 LLMGTLGIV 8.09700 7.67768 0.41932

80 YLWPGPVTV 8.12500 8.09205 0.03295

81 FLLTRILTI 8.14900 7.88150 0.26750

82 GLLGWSPQA 8.23700 8.21175 0.02525

83 ILYQVPFSV 8.31000 8.72222 -0.41222

84 GILTVILGV 8.34700 7.83796 0.50904

85 NMVPFFPPV 8.39800 8.08597 0.31203

86 ILDQVPFSV 8.48100 7.69065 0.79035

87 YLFPGPVTA 8.49500 8.34339 0.15161

88 YLDQVPFSV 8.63800 8.13430 0.50370

89 ILFQVPFSV 8.69900 8.43554 0.26346

90 ILWQVPFSV 8.77000 8.50238 0.26762

Correlation Coeffecient and Stand Error

CORL: 0.887119 CORL~2: 0.786980

RES: 0.366893 SEE: 0.038674

Statistical indices of Training calculation:

ITERATION (B): 38

No. Sequence Expt. Act Predicted Act Difference

1 VALVGLFVL 5.14800 5.75007 -0.60207

2 GTLVALVGL 5.34200 5.93275 -0.59075

3 LQTTIHDII 5.50100 5.80704 -0.30604

4 SLHVGTQCA 5.84200 6.15011 -0.30811

5 ALPYWNFAT 5.86900 6.64481 -0.77581

6 SLNFMGYVI 5.88100 5.96089 -0.07989

7 NLQSLTNLL 6.00000 6.69832 -0.69832

8 FVTWHRYHL 6.02500 5.72584 0.29916

9 DPKVKQWPL 6.17600 5.74043 0.43557

10 ITSQVPFSV 6.19600 6.58324 -0.38724

11 ALAKAAAAI 6.21100 6.24066 -0.02966

12 GLGQVPLIV 6.30100 6.56461 -0.26361

13 MLDLQPETT 6.33500 6.85505 -0.52005

14 LLSSNLSWL 6.34200 6.35010 -0.00810

15 GLACHQLCA 6.38000 6.05137 0.32863

16 LIGNESFAL 6.41500 7.05480 -0.63980

17 ALAKAAAAV 6.41900 6.48068 -0.06168

18 LLAVGATKV 6.47700 6.50951 -0.03251

19 ALAKAAAAL 6.51100 6.22343 0.28757

20 WILRGTSFV 6.55600 6.90793 -0.35193

21 IISCTCPTV 6.58000 6.66383 -0.08383

22 FLGGTPVCL 6.62300 6.87682 -0.25382

23 ALIHHNTHL 6.62300 6.79050 -0.16750

24 NLSWLSLDV 6.63900 6.04690 0.59210

25 YMIMVKCWM 6.66300 6.65564 0.00736

26 VLQAGFFLL 6.68200 7.04324 -0.36124

27 GTLGIVCPI 6.71400 6.51727 0.19673

28 VILGVLLLI 6.78500 7.47487 -0.68987

29 VTWHRYHLL 6.79300 6.55829 0.23471

30 PLLPIFFCL 6.79600 7.52504 -0.72904

31 TLGIVCPIC 6.81500 5.95376 0.86124

32 CLTSTVQLV 6.83200 7.10644 -0.27444

33 ILLLCLIFL 6.84500 6.79003 0.05497

34 FAFRDLCIV 6.88600 6.66414 0.22186

35 FLEPGPVTA 6.89800 7.48875 -0.59075

36 ALAKAAAAA 6.94700 6.79967 0.14733

37 LMAVVLASL 6.95400 7.49680 -0.54280

38 YVITTQHWL 6.98300 6.33969 0.64331

39 LLCLIFLLV 6.99600 7.50839 -0.51239

40 ITAQVPFSV 7.02000 6.66370 0.35630

41 YLEPGPVTL 7.05800 7.14962 -0.09162

42 YTDQVPFSV 7.06600 7.07089 -0.00489

43 NLYVSLLLL 7.11400 6.98161 0.13239

44 ILHNGAYSL 7.12700 7.35055 -0.22355

45 SIISAVVGI 7.15900 7.30311 -0.14411

46 VVMGTLVAL 7.17400 7.31431 -0.14031

47 YLEPGPVTI 7.18700 7.16685 0.02015

48 GLSRYVARL 7.24800 7.46183 -0.21383

49 LLAQFTSAI 7.30100 7.43187 -0.13087

50 VLLDYQGML 7.32800 7.59294 -0.26494

51 YLEPGPVTV 7.34200 7.40687 -0.06487

52 ILSPFMPLL 7.34700 7.14233 0.20467

53 YLSPGPVTA 7.38300 7.55586 -0.17286

54 IIDQVPFSV 7.39800 7.65276 -0.25476

55 SVYDFFVWL 7.44400 7.36620 0.07780

56 ITWQVPFSV 7.46300 7.43949 0.02351

57 ITYQVPFSV 7.48000 7.65961 -0.17961

58 GLYSSTVPV 7.48100 7.62747 -0.14647

59 VMGTLVALV 7.55300 7.23774 0.31526

60 LLLCLIFLL 7.58500 7.14597 0.43903

61 SLDDYNHLV 7.58500 7.17305 0.41195

62 VLIQRNPQL 7.64400 6.94861 0.69539

63 SLYADSPSV 7.65800 7.70825 -0.05025

64 ILSQVPFSV 7.69900 7.64796 0.05104

65 IMDQVPFSV 7.71900 8.03498 -0.31598

66 QLFEDNYAL 7.76400 7.47326 0.29074

67 ALMDKSLHV 7.77000 7.52414 0.24586

68 YAIDLPVSV 7.79600 7.60165 0.19435

69 FVWLHYYSV 7.82400 8.11871 -0.29471

70 MLGTHTMEV 7.84500 7.31687 0.52813

71 LLFGYPVYV 7.88600 8.02995 -0.14395

72 ILKEPVHGV 7.92100 7.59171 0.32929

73 YLMPGPVTV 7.93200 7.91513 0.01687

74 WLDQVPFSV 7.93900 7.95378 -0.01478

75 KTWGQYWQV 7.95500 7.69066 0.26434

76 ALMPLYACI 8.00000 7.44012 0.55988

77 YLAPGPVTA 8.03200 7.63631 0.39569

78 YLYPGPVTV 8.05100 8.31324 -0.26224

79 LLMGTLGIV 8.09700 7.67837 0.41863

80 YLWPGPVTV 8.12500 8.09312 0.03188

81 FLLTRILTI 8.14900 7.88214 0.26686

82 GLLGWSPQA 8.23700 8.21380 0.02320

83 ILYQVPFSV 8.31000 8.72433 -0.41433

84 GILTVILGV 8.34700 7.83898 0.50802

85 NMVPFFPPV 8.39800 8.08698 0.31102

86 ILDQVPFSV 8.48100 7.69141 0.78959

87 YLFPGPVTA 8.49500 8.34517 0.14983

88 YLDQVPFSV 8.63800 8.13561 0.50239

89 ILFQVPFSV 8.69900 8.43727 0.26173

90 ILWQVPFSV 8.77000 8.50421 0.26579

Correlation Coeffecient and Stand Error

CORL: 0.887119 CORL~2: 0.786981

RES: 0.366891 SEE: 0.038674

Statistical indices of Training calculation:

ITERATION (A): 39

No. Sequence Expt. Act Predicted Act Difference

1 VALVGLFVL 5.14800 5.75226 -0.60426

2 GTLVALVGL 5.34200 5.93456 -0.59256

3 LQTTIHDII 5.50100 5.80928 -0.30828

4 SLHVGTQCA 5.84200 6.15192 -0.30992

5 ALPYWNFAT 5.86900 6.64565 -0.77665

6 SLNFMGYVI 5.88100 5.96279 -0.08179

7 NLQSLTNLL 6.00000 6.69912 -0.69912

8 FVTWHRYHL 6.02500 5.72812 0.29688

9 DPKVKQWPL 6.17600 5.74241 0.43359

10 ITSQVPFSV 6.19600 6.58400 -0.38800

11 ALAKAAAAI 6.21100 6.24217 -0.03117

12 GLGQVPLIV 6.30100 6.56549 -0.26449

13 MLDLQPETT 6.33500 6.85567 -0.52067

14 LLSSNLSWL 6.34200 6.35147 -0.00947

15 GLACHQLCA 6.38000 6.05354 0.32646

16 LIGNESFAL 6.41500 7.05518 -0.64018

17 ALAKAAAAV 6.41900 6.48185 -0.06285

18 LLAVGATKV 6.47700 6.51061 -0.03361

19 ALAKAAAAL 6.51100 6.22500 0.28600

20 WILRGTSFV 6.55600 6.90845 -0.35245

21 IISCTCPTV 6.58000 6.66485 -0.08485

22 FLGGTPVCL 6.62300 6.87737 -0.25437

23 ALIHHNTHL 6.62300 6.79117 -0.16817

24 NLSWLSLDV 6.63900 6.04873 0.59027

25 YMIMVKCWM 6.66300 6.65649 0.00651

26 VLQAGFFLL 6.68200 7.04361 -0.36161

27 GTLGIVCPI 6.71400 6.51834 0.19566

28 VILGVLLLI 6.78500 7.47458 -0.68958

29 VTWHRYHLL 6.79300 6.55922 0.23378

30 PLLPIFFCL 6.79600 7.52489 -0.72889

31 TLGIVCPIC 6.81500 5.95562 0.85938

32 CLTSTVQLV 6.83200 7.10645 -0.27445

33 ILLLCLIFL 6.84500 6.79063 0.05437

34 FAFRDLCIV 6.88600 6.66486 0.22114

35 FLEPGPVTA 6.89800 7.48857 -0.59057

36 ALAKAAAAA 6.94700 6.80039 0.14661

37 LMAVVLASL 6.95400 7.49630 -0.54230

38 YVITTQHWL 6.98300 6.34117 0.64183

39 LLCLIFLLV 6.99600 7.50789 -0.51189

40 ITAQVPFSV 7.02000 6.66429 0.35571

41 YLEPGPVTL 7.05800 7.14995 -0.09195

42 YTDQVPFSV 7.06600 7.07090 -0.00490

43 NLYVSLLLL 7.11400 6.98204 0.13196

44 ILHNGAYSL 7.12700 7.35040 -0.22340

45 SIISAVVGI 7.15900 7.30307 -0.14407

46 VVMGTLVAL 7.17400 7.31419 -0.14019

47 YLEPGPVTI 7.18700 7.16712 0.01988

48 GLSRYVARL 7.24800 7.46151 -0.21351

49 LLAQFTSAI 7.30100 7.43151 -0.13051

50 VLLDYQGML 7.32800 7.59249 -0.26449

51 YLEPGPVTV 7.34200 7.40680 -0.06480

52 ILSPFMPLL 7.34700 7.14265 0.20435

53 YLSPGPVTA 7.38300 7.55562 -0.17262

54 IIDQVPFSV 7.39800 7.65210 -0.25410

55 SVYDFFVWL 7.44400 7.36606 0.07794

56 ITWQVPFSV 7.46300 7.43894 0.02406

57 ITYQVPFSV 7.48000 7.65875 -0.17875

58 GLYSSTVPV 7.48100 7.62701 -0.14601

59 VMGTLVALV 7.55300 7.23775 0.31525

60 LLLCLIFLL 7.58500 7.14634 0.43866

61 SLDDYNHLV 7.58500 7.17320 0.41180

62 VLIQRNPQL 7.64400 6.94902 0.69498

63 SLYADSPSV 7.65800 7.70757 -0.04957

64 ILSQVPFSV 7.69900 7.64725 0.05175

65 IMDQVPFSV 7.71900 8.03360 -0.31460

66 QLFEDNYAL 7.76400 7.47299 0.29101

67 ALMDKSLHV 7.77000 7.52361 0.24639

68 YAIDLPVSV 7.79600 7.60096 0.19504

69 FVWLHYYSV 7.82400 8.11743 -0.29343

70 MLGTHTMEV 7.84500 7.31679 0.52821

71 LLFGYPVYV 7.88600 8.02867 -0.14267

72 ILKEPVHGV 7.92100 7.59105 0.32995

73 YLMPGPVTV 7.93200 7.91426 0.01774

74 WLDQVPFSV 7.93900 7.95259 -0.01359

75 KTWGQYWQV 7.95500 7.68983 0.26517

76 ALMPLYACI 8.00000 7.44010 0.55990

77 YLAPGPVTA 8.03200 7.63591 0.39609

78 YLYPGPVTV 8.05100 8.31182 -0.26082

79 LLMGTLGIV 8.09700 7.67762 0.41938

80 YLWPGPVTV 8.12500 8.09202 0.03298

81 FLLTRILTI 8.14900 7.88133 0.26767

82 GLLGWSPQA 8.23700 8.21233 0.02467

83 ILYQVPFSV 8.31000 8.72200 -0.41200

84 GILTVILGV 8.34700 7.83826 0.50874

85 NMVPFFPPV 8.39800 8.08593 0.31207

86 ILDQVPFSV 8.48100 7.69063 0.79037

87 YLFPGPVTA 8.49500 8.34373 0.15127

88 YLDQVPFSV 8.63800 8.13415 0.50385

89 ILFQVPFSV 8.69900 8.43536 0.26364

90 ILWQVPFSV 8.77000 8.50220 0.26780

Correlation Coeffecient and Stand Error

CORL: 0.887121 CORL~2: 0.786983

RES: 0.366890 SEE: 0.038674

Statistical indices of Training calculation:

ITERATION (B): 39

No. Sequence Expt. Act Predicted Act Difference

1 VALVGLFVL 5.14800 5.75044 -0.60244

2 GTLVALVGL 5.34200 5.93310 -0.59110

3 LQTTIHDII 5.50100 5.80767 -0.30667

4 SLHVGTQCA 5.84200 6.15079 -0.30879

5 ALPYWNFAT 5.86900 6.64453 -0.77553

6 SLNFMGYVI 5.88100 5.96047 -0.07947

7 NLQSLTNLL 6.00000 6.69840 -0.69840

8 FVTWHRYHL 6.02500 5.72560 0.29940

9 DPKVKQWPL 6.17600 5.74045 0.43555

10 ITSQVPFSV 6.19600 6.58372 -0.38772

11 ALAKAAAAI 6.21100 6.24088 -0.02988

12 GLGQVPLIV 6.30100 6.56465 -0.26365

13 MLDLQPETT 6.33500 6.85522 -0.52022

14 LLSSNLSWL 6.34200 6.35011 -0.00811

15 GLACHQLCA 6.38000 6.05206 0.32794

16 LIGNESFAL 6.41500 7.05489 -0.63989

17 ALAKAAAAV 6.41900 6.48111 -0.06211

18 LLAVGATKV 6.47700 6.50968 -0.03268

19 ALAKAAAAL 6.51100 6.22367 0.28733

20 WILRGTSFV 6.55600 6.90797 -0.35197

21 IISCTCPTV 6.58000 6.66393 -0.08393

22 FLGGTPVCL 6.62300 6.87671 -0.25371

23 ALIHHNTHL 6.62300 6.79052 -0.16752

24 NLSWLSLDV 6.63900 6.04688 0.59212

25 YMIMVKCWM 6.66300 6.65452 0.00848

26 VLQAGFFLL 6.68200 7.04306 -0.36106

27 GTLGIVCPI 6.71400 6.51780 0.19620

28 VILGVLLLI 6.78500 7.47469 -0.68969

29 VTWHRYHLL 6.79300 6.55841 0.23459

30 PLLPIFFCL 6.79600 7.52475 -0.72875

31 TLGIVCPIC 6.81500 5.95343 0.86157

32 CLTSTVQLV 6.83200 7.10641 -0.27441

33 ILLLCLIFL 6.84500 6.78929 0.05571

34 FAFRDLCIV 6.88600 6.66455 0.22145

35 FLEPGPVTA 6.89800 7.48920 -0.59120

36 ALAKAAAAA 6.94700 6.80039 0.14661

37 LMAVVLASL 6.95400 7.49628 -0.54228

38 YVITTQHWL 6.98300 6.33980 0.64320

39 LLCLIFLLV 6.99600 7.50779 -0.51179

40 ITAQVPFSV 7.02000 6.66411 0.35589

41 YLEPGPVTL 7.05800 7.14951 -0.09151

42 YTDQVPFSV 7.06600 7.07117 -0.00517

43 NLYVSLLLL 7.11400 6.98121 0.13279

44 ILHNGAYSL 7.12700 7.35045 -0.22345

45 SIISAVVGI 7.15900 7.30326 -0.14426

46 VVMGTLVAL 7.17400 7.31447 -0.14047

47 YLEPGPVTI 7.18700 7.16673 0.02027

48 GLSRYVARL 7.24800 7.46184 -0.21384

49 LLAQFTSAI 7.30100 7.43173 -0.13073

50 VLLDYQGML 7.32800 7.59279 -0.26479

51 YLEPGPVTV 7.34200 7.40695 -0.06495

52 ILSPFMPLL 7.34700 7.14213 0.20487

53 YLSPGPVTA 7.38300 7.55631 -0.17331

54 IIDQVPFSV 7.39800 7.65277 -0.25477

55 SVYDFFVWL 7.44400 7.36612 0.07788

56 ITWQVPFSV 7.46300 7.43968 0.02332

57 ITYQVPFSV 7.48000 7.65975 -0.17975

58 GLYSSTVPV 7.48100 7.62772 -0.14672

59 VMGTLVALV 7.55300 7.23767 0.31533

60 LLLCLIFLL 7.58500 7.14550 0.43950

61 SLDDYNHLV 7.58500 7.17334 0.41166

62 VLIQRNPQL 7.64400 6.94850 0.69550

63 SLYADSPSV 7.65800 7.70845 -0.05045

64 ILSQVPFSV 7.69900 7.64790 0.05110

65 IMDQVPFSV 7.71900 8.03459 -0.31559

66 QLFEDNYAL 7.76400 7.47309 0.29091

67 ALMDKSLHV 7.77000 7.52422 0.24578

68 YAIDLPVSV 7.79600 7.60215 0.19385

69 FVWLHYYSV 7.82400 8.11838 -0.29438

70 MLGTHTMEV 7.84500 7.31697 0.52803

71 LLFGYPVYV 7.88600 8.02955 -0.14355

72 ILKEPVHGV 7.92100 7.59169 0.32931

73 YLMPGPVTV 7.93200 7.91503 0.01697

74 WLDQVPFSV 7.93900 7.95358 -0.01458

75 KTWGQYWQV 7.95500 7.69090 0.26410

76 ALMPLYACI 8.00000 7.43996 0.56004

77 YLAPGPVTA 8.03200 7.63670 0.39530

78 YLYPGPVTV 8.05100 8.31306 -0.26206

79 LLMGTLGIV 8.09700 7.67824 0.41876

80 YLWPGPVTV 8.12500 8.09299 0.03201

81 FLLTRILTI 8.14900 7.88192 0.26708

82 GLLGWSPQA 8.23700 8.21420 0.02280

83 ILYQVPFSV 8.31000 8.72393 -0.41393

84 GILTVILGV 8.34700 7.83920 0.50780

85 NMVPFFPPV 8.39800 8.08684 0.31116

86 ILDQVPFSV 8.48100 7.69133 0.78967

87 YLFPGPVTA 8.49500 8.34535 0.14965

88 YLDQVPFSV 8.63800 8.13535 0.50265

89 ILFQVPFSV 8.69900 8.43694 0.26206

90 ILWQVPFSV 8.77000 8.50386 0.26614

Correlation Coeffecient and Stand Error

CORL: 0.887121 CORL~2: 0.786984

RES: 0.366888 SEE: 0.038673

Statistical indices of Training calculation:

ITERATION (A): 40

No. Sequence Expt. Act Predicted Act Difference

1 VALVGLFVL 5.14800 5.75243 -0.60443

2 GTLVALVGL 5.34200 5.93476 -0.59276

3 LQTTIHDII 5.50100 5.80971 -0.30871

4 SLHVGTQCA 5.84200 6.15243 -0.31043

5 ALPYWNFAT 5.86900 6.64529 -0.77629

6 SLNFMGYVI 5.88100 5.96221 -0.08121

7 NLQSLTNLL 6.00000 6.69912 -0.69912

8 FVTWHRYHL 6.02500 5.72768 0.29732

9 DPKVKQWPL 6.17600 5.74226 0.43374

10 ITSQVPFSV 6.19600 6.58441 -0.38841

11 ALAKAAAAI 6.21100 6.24226 -0.03126

12 GLGQVPLIV 6.30100 6.56545 -0.26445

13 MLDLQPETT 6.33500 6.85578 -0.52078

14 LLSSNLSWL 6.34200 6.35136 -0.00936

15 GLACHQLCA 6.38000 6.05404 0.32596

16 LIGNESFAL 6.41500 7.05524 -0.64024

17 ALAKAAAAV 6.41900 6.48218 -0.06318

18 LLAVGATKV 6.47700 6.51068 -0.03368

19 ALAKAAAAL 6.51100 6.22509 0.28591

20 WILRGTSFV 6.55600 6.90844 -0.35244

21 IISCTCPTV 6.58000 6.66486 -0.08486

22 FLGGTPVCL 6.62300 6.87722 -0.25422

23 ALIHHNTHL 6.62300 6.79113 -0.16813

24 NLSWLSLDV 6.63900 6.04855 0.59045

25 YMIMVKCWM 6.66300 6.65530 0.00770

26 VLQAGFFLL 6.68200 7.04340 -0.36140

27 GTLGIVCPI 6.71400 6.51877 0.19523

28 VILGVLLLI 6.78500 7.47443 -0.68943

29 VTWHRYHLL 6.79300 6.55926 0.23374

30 PLLPIFFCL 6.79600 7.52461 -0.72861

31 TLGIVCPIC 6.81500 5.95513 0.85987

32 CLTSTVQLV 6.83200 7.10641 -0.27441

33 ILLLCLIFL 6.84500 6.78985 0.05515

34 FAFRDLCIV 6.88600 6.66521 0.22079

35 FLEPGPVTA 6.89800 7.48904 -0.59104

36 ALAKAAAAA 6.94700 6.80105 0.14595

37 LMAVVLASL 6.95400 7.49582 -0.54182

38 YVITTQHWL 6.98300 6.34115 0.64185

39 LLCLIFLLV 6.99600 7.50734 -0.51134

40 ITAQVPFSV 7.02000 6.66466 0.35534

41 YLEPGPVTL 7.05800 7.14981 -0.09181

42 YTDQVPFSV 7.06600 7.07118 -0.00518

43 NLYVSLLLL 7.11400 6.98160 0.13240

44 ILHNGAYSL 7.12700 7.35031 -0.22331

45 SIISAVVGI 7.15900 7.30322 -0.14422

46 VVMGTLVAL 7.17400 7.31437 -0.14037

47 YLEPGPVTI 7.18700 7.16698 0.02002

48 GLSRYVARL 7.24800 7.46154 -0.21354

49 LLAQFTSAI 7.30100 7.43140 -0.13040

50 VLLDYQGML 7.32800 7.59238 -0.26438

51 YLEPGPVTV 7.34200 7.40689 -0.06489

52 ILSPFMPLL 7.34700 7.14242 0.20458

53 YLSPGPVTA 7.38300 7.55608 -0.17308

54 IIDQVPFSV 7.39800 7.65216 -0.25416

55 SVYDFFVWL 7.44400 7.36600 0.07800

56 ITWQVPFSV 7.46300 7.43918 0.02382

57 ITYQVPFSV 7.48000 7.65896 -0.17896

58 GLYSSTVPV 7.48100 7.62730 -0.14630

59 VMGTLVALV 7.55300 7.23768 0.31532

60 LLLCLIFLL 7.58500 7.14585 0.43915

61 SLDDYNHLV 7.58500 7.17347 0.41153

62 VLIQRNPQL 7.64400 6.94887 0.69513

63 SLYADSPSV 7.65800 7.70783 -0.04983

64 ILSQVPFSV 7.69900 7.64725 0.05175

65 IMDQVPFSV 7.71900 8.03333 -0.31433

66 QLFEDNYAL 7.76400 7.47284 0.29116

67 ALMDKSLHV 7.77000 7.52373 0.24627

68 YAIDLPVSV 7.79600 7.60153 0.19447

69 FVWLHYYSV 7.82400 8.11721 -0.29321

70 MLGTHTMEV 7.84500 7.31689 0.52811

71 LLFGYPVYV 7.88600 8.02838 -0.14238

72 ILKEPVHGV 7.92100 7.59109 0.32991

73 YLMPGPVTV 7.93200 7.91424 0.01776

74 WLDQVPFSV 7.93900 7.95249 -0.01349

75 KTWGQYWQV 7.95500 7.69013 0.26487

76 ALMPLYACI 8.00000 7.43995 0.56005

77 YLAPGPVTA 8.03200 7.63633 0.39567

78 YLYPGPVTV 8.05100 8.31176 -0.26076

79 LLMGTLGIV 8.09700 7.67756 0.41944

80 YLWPGPVTV 8.12500 8.09199 0.03301

81 FLLTRILTI 8.14900 7.88118 0.26782

82 GLLGWSPQA 8.23700 8.21285 0.02415

83 ILYQVPFSV 8.31000 8.72180 -0.41180

84 GILTVILGV 8.34700 7.83854 0.50846

85 NMVPFFPPV 8.39800 8.08588 0.31212

86 ILDQVPFSV 8.48100 7.69062 0.79038

87 YLFPGPVTA 8.49500 8.34403 0.15097

88 YLDQVPFSV 8.63800 8.13402 0.50398

89 ILFQVPFSV 8.69900 8.43520 0.26380

90 ILWQVPFSV 8.77000 8.50202 0.26798

Correlation Coeffecient and Stand Error

CORL: 0.887123 CORL~2: 0.786986

RES: 0.366887 SEE: 0.038673

Statistical indices of Training calculation:

ITERATION (B): 40

No. Sequence Expt. Act Predicted Act Difference

1 VALVGLFVL 5.14800 5.75077 -0.60277

2 GTLVALVGL 5.34200 5.93341 -0.59141

3 LQTTIHDII 5.50100 5.80824 -0.30724

4 SLHVGTQCA 5.84200 6.15140 -0.30940

5 ALPYWNFAT 5.86900 6.64427 -0.77527

6 SLNFMGYVI 5.88100 5.96009 -0.07909

7 NLQSLTNLL 6.00000 6.69847 -0.69847

8 FVTWHRYHL 6.02500 5.72538 0.29962

9 DPKVKQWPL 6.17600 5.74046 0.43554

10 ITSQVPFSV 6.19600 6.58415 -0.38815

11 ALAKAAAAI 6.21100 6.24108 -0.03008

12 GLGQVPLIV 6.30100 6.56468 -0.26368

13 MLDLQPETT 6.33500 6.85537 -0.52037

14 LLSSNLSWL 6.34200 6.35012 -0.00812

15 GLACHQLCA 6.38000 6.05269 0.32731

16 LIGNESFAL 6.41500 7.05498 -0.63998

17 ALAKAAAAV 6.41900 6.48150 -0.06250

18 LLAVGATKV 6.47700 6.50984 -0.03284

19 ALAKAAAAL 6.51100 6.22388 0.28712

20 WILRGTSFV 6.55600 6.90800 -0.35200

21 IISCTCPTV 6.58000 6.66401 -0.08401

22 FLGGTPVCL 6.62300 6.87662 -0.25362

23 ALIHHNTHL 6.62300 6.79054 -0.16754

24 NLSWLSLDV 6.63900 6.04686 0.59214

25 YMIMVKCWM 6.66300 6.65349 0.00951

26 VLQAGFFLL 6.68200 7.04290 -0.36090

27 GTLGIVCPI 6.71400 6.51827 0.19573

28 VILGVLLLI 6.78500 7.47452 -0.68952

29 VTWHRYHLL 6.79300 6.55852 0.23448

30 PLLPIFFCL 6.79600 7.52449 -0.72849

31 TLGIVCPIC 6.81500 5.95313 0.86187

32 CLTSTVQLV 6.83200 7.10638 -0.27438

33 ILLLCLIFL 6.84500 6.78863 0.05637

34 FAFRDLCIV 6.88600 6.66492 0.22108

35 FLEPGPVTA 6.89800 7.48961 -0.59161

36 ALAKAAAAA 6.94700 6.80105 0.14595

37 LMAVVLASL 6.95400 7.49581 -0.54181

38 YVITTQHWL 6.98300 6.33991 0.64309

39 LLCLIFLLV 6.99600 7.50725 -0.51125

40 ITAQVPFSV 7.02000 6.66449 0.35551

41 YLEPGPVTL 7.05800 7.14941 -0.09141

42 YTDQVPFSV 7.06600 7.07143 -0.00543

43 NLYVSLLLL 7.11400 6.98084 0.13316

44 ILHNGAYSL 7.12700 7.35035 -0.22335

45 SIISAVVGI 7.15900 7.30339 -0.14439

46 VVMGTLVAL 7.17400 7.31463 -0.14063

47 YLEPGPVTI 7.18700 7.16661 0.02039

48 GLSRYVARL 7.24800 7.46185 -0.21385

49 LLAQFTSAI 7.30100 7.43160 -0.13060

50 VLLDYQGML 7.32800 7.59264 -0.26464

51 YLEPGPVTV 7.34200 7.40703 -0.06503

52 ILSPFMPLL 7.34700 7.14195 0.20505

53 YLSPGPVTA 7.38300 7.55671 -0.17371

54 IIDQVPFSV 7.39800 7.65277 -0.25477

55 SVYDFFVWL 7.44400 7.36606 0.07794

56 ITWQVPFSV 7.46300 7.43986 0.02314

57 ITYQVPFSV 7.48000 7.65988 -0.17988

58 GLYSSTVPV 7.48100 7.62794 -0.14694

59 VMGTLVALV 7.55300 7.23761 0.31539

60 LLLCLIFLL 7.58500 7.14508 0.43992

61 SLDDYNHLV 7.58500 7.17361 0.41139

62 VLIQRNPQL 7.64400 6.94839 0.69561

63 SLYADSPSV 7.65800 7.70863 -0.05063

64 ILSQVPFSV 7.69900 7.64784 0.05116

65 IMDQVPFSV 7.71900 8.03424 -0.31524

66 QLFEDNYAL 7.76400 7.47293 0.29107

67 ALMDKSLHV 7.77000 7.52429 0.24571

68 YAIDLPVSV 7.79600 7.60261 0.19339

69 FVWLHYYSV 7.82400 8.11808 -0.29408

70 MLGTHTMEV 7.84500 7.31706 0.52794

71 LLFGYPVYV 7.88600 8.02918 -0.14318

72 ILKEPVHGV 7.92100 7.59167 0.32933

73 YLMPGPVTV 7.93200 7.91493 0.01707

74 WLDQVPFSV 7.93900 7.95340 -0.01440

75 KTWGQYWQV 7.95500 7.69111 0.26389

76 ALMPLYACI 8.00000 7.43982 0.56018

77 YLAPGPVTA 8.03200 7.63705 0.39495

78 YLYPGPVTV 8.05100 8.31289 -0.26189

79 LLMGTLGIV 8.09700 7.67813 0.41887

80 YLWPGPVTV 8.12500 8.09287 0.03213

81 FLLTRILTI 8.14900 7.88172 0.26728

82 GLLGWSPQA 8.23700 8.21457 0.02243

83 ILYQVPFSV 8.31000 8.72356 -0.41356

84 GILTVILGV 8.34700 7.83939 0.50761

85 NMVPFFPPV 8.39800 8.08672 0.31128

86 ILDQVPFSV 8.48100 7.69125 0.78975

87 YLFPGPVTA 8.49500 8.34552 0.14948

88 YLDQVPFSV 8.63800 8.13511 0.50289

89 ILFQVPFSV 8.69900 8.43664 0.26236

90 ILWQVPFSV 8.77000 8.50354 0.26646

Correlation Coeffecient and Stand Error

CORL: 0.887123 CORL~2: 0.786987

RES: 0.366886 SEE: 0.038673

Statistical indices of Training calculation:

ITERATION (A): 41

No. Sequence Expt. Act Predicted Act Difference

1 VALVGLFVL 5.14800 5.75259 -0.60459

2 GTLVALVGL 5.34200 5.93493 -0.59293

3 LQTTIHDII 5.50100 5.81010 -0.30910

4 SLHVGTQCA 5.84200 6.15291 -0.31091

5 ALPYWNFAT 5.86900 6.64497 -0.77597

6 SLNFMGYVI 5.88100 5.96168 -0.08068

7 NLQSLTNLL 6.00000 6.69913 -0.69913

8 FVTWHRYHL 6.02500 5.72728 0.29772

9 DPKVKQWPL 6.17600 5.74212 0.43388

10 ITSQVPFSV 6.19600 6.58478 -0.38878

11 ALAKAAAAI 6.21100 6.24234 -0.03134

12 GLGQVPLIV 6.30100 6.56542 -0.26442

13 MLDLQPETT 6.33500 6.85588 -0.52088

14 LLSSNLSWL 6.34200 6.35126 -0.00926

15 GLACHQLCA 6.38000 6.05450 0.32550

16 LIGNESFAL 6.41500 7.05530 -0.64030

17 ALAKAAAAV 6.41900 6.48248 -0.06348

18 LLAVGATKV 6.47700 6.51075 -0.03375

19 ALAKAAAAL 6.51100 6.22518 0.28582

20 WILRGTSFV 6.55600 6.90843 -0.35243

21 IISCTCPTV 6.58000 6.66486 -0.08486

22 FLGGTPVCL 6.62300 6.87708 -0.25408

23 ALIHHNTHL 6.62300 6.79110 -0.16810

24 NLSWLSLDV 6.63900 6.04839 0.59061

25 YMIMVKCWM 6.66300 6.65421 0.00879

26 VLQAGFFLL 6.68200 7.04321 -0.36121

27 GTLGIVCPI 6.71400 6.51916 0.19484

28 VILGVLLLI 6.78500 7.47428 -0.68928

29 VTWHRYHLL 6.79300 6.55929 0.23371

30 PLLPIFFCL 6.79600 7.52436 -0.72836

31 TLGIVCPIC 6.81500 5.95468 0.86032

32 CLTSTVQLV 6.83200 7.10639 -0.27439

33 ILLLCLIFL 6.84500 6.78913 0.05587

34 FAFRDLCIV 6.88600 6.66552 0.22048

35 FLEPGPVTA 6.89800 7.48946 -0.59146

36 ALAKAAAAA 6.94700 6.80165 0.14535

37 LMAVVLASL 6.95400 7.49539 -0.54139

38 YVITTQHWL 6.98300 6.34114 0.64186

39 LLCLIFLLV 6.99600 7.50684 -0.51084

40 ITAQVPFSV 7.02000 6.66499 0.35501

41 YLEPGPVTL 7.05800 7.14968 -0.09168

42 YTDQVPFSV 7.06600 7.07144 -0.00544

43 NLYVSLLLL 7.11400 6.98120 0.13280

44 ILHNGAYSL 7.12700 7.35023 -0.22323

45 SIISAVVGI 7.15900 7.30336 -0.14436

46 VVMGTLVAL 7.17400 7.31453 -0.14053

47 YLEPGPVTI 7.18700 7.16684 0.02016

48 GLSRYVARL 7.24800 7.46158 -0.21358

49 LLAQFTSAI 7.30100 7.43130 -0.13030

50 VLLDYQGML 7.32800 7.59227 -0.26427

51 YLEPGPVTV 7.34200 7.40697 -0.06497

52 ILSPFMPLL 7.34700 7.14221 0.20479

53 YLSPGPVTA 7.38300 7.55651 -0.17351

54 IIDQVPFSV 7.39800 7.65221 -0.25421

55 SVYDFFVWL 7.44400 7.36594 0.07806

56 ITWQVPFSV 7.46300 7.43941 0.02359

57 ITYQVPFSV 7.48000 7.65916 -0.17916

58 GLYSSTVPV 7.48100 7.62756 -0.14656

59 VMGTLVALV 7.55300 7.23762 0.31538

60 LLLCLIFLL 7.58500 7.14539 0.43961

61 SLDDYNHLV 7.58500 7.17373 0.41127

62 VLIQRNPQL 7.64400 6.94873 0.69527

63 SLYADSPSV 7.65800 7.70806 -0.05006

64 ILSQVPFSV 7.69900 7.64725 0.05175

65 IMDQVPFSV 7.71900 8.03308 -0.31408

66 QLFEDNYAL 7.76400 7.47271 0.29129

67 ALMDKSLHV 7.77000 7.52385 0.24615

68 YAIDLPVSV 7.79600 7.60204 0.19396

69 FVWLHYYSV 7.82400 8.11702 -0.29302

70 MLGTHTMEV 7.84500 7.31699 0.52801

71 LLFGYPVYV 7.88600 8.02812 -0.14212

72 ILKEPVHGV 7.92100 7.59112 0.32988

73 YLMPGPVTV 7.93200 7.91421 0.01779

74 WLDQVPFSV 7.93900 7.95240 -0.01340

75 KTWGQYWQV 7.95500 7.69041 0.26459

76 ALMPLYACI 8.00000 7.43981 0.56019

77 YLAPGPVTA 8.03200 7.63671 0.39529

78 YLYPGPVTV 8.05100 8.31171 -0.26071

79 LLMGTLGIV 8.09700 7.67750 0.41950

80 YLWPGPVTV 8.12500 8.09196 0.03304

81 FLLTRILTI 8.14900 7.88105 0.26795

82 GLLGWSPQA 8.23700 8.21333 0.02367

83 ILYQVPFSV 8.31000 8.72162 -0.41162

84 GILTVILGV 8.34700 7.83879 0.50821

85 NMVPFFPPV 8.39800 8.08584 0.31216

86 ILDQVPFSV 8.48100 7.69060 0.79040

87 YLFPGPVTA 8.49500 8.34431 0.15069

88 YLDQVPFSV 8.63800 8.13390 0.50410

89 ILFQVPFSV 8.69900 8.43505 0.26395

90 ILWQVPFSV 8.77000 8.50187 0.26813

Correlation Coeffecient and Stand Error

CORL: 0.887124 CORL~2: 0.786989

RES: 0.366885 SEE: 0.038673

Statistical indices of Training calculation:

ITERATION (B): 41

No. Sequence Expt. Act Predicted Act Difference

1 VALVGLFVL 5.14800 5.75108 -0.60308

2 GTLVALVGL 5.34200 5.93371 -0.59171

3 LQTTIHDII 5.50100 5.80875 -0.30775

4 SLHVGTQCA 5.84200 6.15197 -0.30997

5 ALPYWNFAT 5.86900 6.64404 -0.77504

6 SLNFMGYVI 5.88100 5.95974 -0.07874

7 NLQSLTNLL 6.00000 6.69853 -0.69853

8 FVTWHRYHL 6.02500 5.72518 0.29982

9 DPKVKQWPL 6.17600 5.74048 0.43552

10 ITSQVPFSV 6.19600 6.58455 -0.38855

11 ALAKAAAAI 6.21100 6.24127 -0.03027

12 GLGQVPLIV 6.30100 6.56472 -0.26372

13 MLDLQPETT 6.33500 6.85551 -0.52051

14 LLSSNLSWL 6.34200 6.35013 -0.00813

15 GLACHQLCA 6.38000 6.05326 0.32674

16 LIGNESFAL 6.41500 7.05506 -0.64006

17 ALAKAAAAV 6.41900 6.48186 -0.06286

18 LLAVGATKV 6.47700 6.50998 -0.03298

19 ALAKAAAAL 6.51100 6.22408 0.28692

20 WILRGTSFV 6.55600 6.90803 -0.35203

21 IISCTCPTV 6.58000 6.66409 -0.08409

22 FLGGTPVCL 6.62300 6.87653 -0.25353

23 ALIHHNTHL 6.62300 6.79056 -0.16756

24 NLSWLSLDV 6.63900 6.04685 0.59215

25 YMIMVKCWM 6.66300 6.65256 0.01044

26 VLQAGFFLL 6.68200 7.04275 -0.36075

27 GTLGIVCPI 6.71400 6.51871 0.19529

28 VILGVLLLI 6.78500 7.47437 -0.68937

29 VTWHRYHLL 6.79300 6.55862 0.23438

30 PLLPIFFCL 6.79600 7.52425 -0.72825

31 TLGIVCPIC 6.81500 5.95285 0.86215

32 CLTSTVQLV 6.83200 7.10636 -0.27436

33 ILLLCLIFL 6.84500 6.78802 0.05698

34 FAFRDLCIV 6.88600 6.66526 0.22074

35 FLEPGPVTA 6.89800 7.48998 -0.59198

36 ALAKAAAAA 6.94700 6.80165 0.14535

37 LMAVVLASL 6.95400 7.49537 -0.54137

38 YVITTQHWL 6.98300 6.34000 0.64300

39 LLCLIFLLV 6.99600 7.50676 -0.51076

40 ITAQVPFSV 7.02000 6.66483 0.35517

41 YLEPGPVTL 7.05800 7.14932 -0.09132

42 YTDQVPFSV 7.06600 7.07166 -0.00566

43 NLYVSLLLL 7.11400 6.98051 0.13349

44 ILHNGAYSL 7.12700 7.35026 -0.22326

45 SIISAVVGI 7.15900 7.30352 -0.14452

46 VVMGTLVAL 7.17400 7.31477 -0.14077

47 YLEPGPVTI 7.18700 7.16651 0.02049

48 GLSRYVARL 7.24800 7.46186 -0.21386

49 LLAQFTSAI 7.30100 7.43148 -0.13048

50 VLLDYQGML 7.32800 7.59251 -0.26451

51 YLEPGPVTV 7.34200 7.40710 -0.06510

52 ILSPFMPLL 7.34700 7.14178 0.20522

53 YLSPGPVTA 7.38300 7.55708 -0.17408

54 IIDQVPFSV 7.39800 7.65277 -0.25477

55 SVYDFFVWL 7.44400 7.36600 0.07800

56 ITWQVPFSV 7.46300 7.44002 0.02298

57 ITYQVPFSV 7.48000 7.66000 -0.18000

58 GLYSSTVPV 7.48100 7.62815 -0.14715

59 VMGTLVALV 7.55300 7.23755 0.31545

60 LLLCLIFLL 7.58500 7.14470 0.44030

61 SLDDYNHLV 7.58500 7.17385 0.41115

62 VLIQRNPQL 7.64400 6.94830 0.69570

63 SLYADSPSV 7.65800 7.70880 -0.05080

64 ILSQVPFSV 7.69900 7.64778 0.05122

65 IMDQVPFSV 7.71900 8.03391 -0.31491

66 QLFEDNYAL 7.76400 7.47279 0.29121

67 ALMDKSLHV 7.77000 7.52435 0.24565

68 YAIDLPVSV 7.79600 7.60303 0.19297

69 FVWLHYYSV 7.82400 8.11781 -0.29381

70 MLGTHTMEV 7.84500 7.31714 0.52786

71 LLFGYPVYV 7.88600 8.02885 -0.14285

72 ILKEPVHGV 7.92100 7.59165 0.32935

73 YLMPGPVTV 7.93200 7.91485 0.01715

74 WLDQVPFSV 7.93900 7.95323 -0.01423

75 KTWGQYWQV 7.95500 7.69130 0.26370

76 ALMPLYACI 8.00000 7.43970 0.56030

77 YLAPGPVTA 8.03200 7.63737 0.39463

78 YLYPGPVTV 8.05100 8.31274 -0.26174

79 LLMGTLGIV 8.09700 7.67802 0.41898

80 YLWPGPVTV 8.12500 8.09277 0.03223

81 FLLTRILTI 8.14900 7.88153 0.26747

82 GLLGWSPQA 8.23700 8.21490 0.02210

83 ILYQVPFSV 8.31000 8.72323 -0.41323

84 GILTVILGV 8.34700 7.83957 0.50743

85 NMVPFFPPV 8.39800 8.08661 0.31139

86 ILDQVPFSV 8.48100 7.69118 0.78982

87 YLFPGPVTA 8.49500 8.34567 0.14933

88 YLDQVPFSV 8.63800 8.13490 0.50310

89 ILFQVPFSV 8.69900 8.43637 0.26263

90 ILWQVPFSV 8.77000 8.50326 0.26674

Correlation Coeffecient and Stand Error

CORL: 0.887124 CORL~2: 0.786989

RES: 0.366884 SEE: 0.038673

Statistical indices of Training calculation:

ITERATION (A): 42

No. Sequence Expt. Act Predicted Act Difference

1 VALVGLFVL 5.14800 5.75274 -0.60474

2 GTLVALVGL 5.34200 5.93509 -0.59309

3 LQTTIHDII 5.50100 5.81046 -0.30946

4 SLHVGTQCA 5.84200 6.15334 -0.31134

5 ALPYWNFAT 5.86900 6.64468 -0.77568

6 SLNFMGYVI 5.88100 5.96119 -0.08019

7 NLQSLTNLL 6.00000 6.69914 -0.69914

8 FVTWHRYHL 6.02500 5.72692 0.29808

9 DPKVKQWPL 6.17600 5.74199 0.43401

10 ITSQVPFSV 6.19600 6.58512 -0.38912

11 ALAKAAAAI 6.21100 6.24242 -0.03142

12 GLGQVPLIV 6.30100 6.56539 -0.26439

13 MLDLQPETT 6.33500 6.85598 -0.52098

14 LLSSNLSWL 6.34200 6.35117 -0.00917

15 GLACHQLCA 6.38000 6.05492 0.32508

16 LIGNESFAL 6.41500 7.05535 -0.64035

17 ALAKAAAAV 6.41900 6.48275 -0.06375

18 LLAVGATKV 6.47700 6.51081 -0.03381

19 ALAKAAAAL 6.51100 6.22527 0.28573

20 WILRGTSFV 6.55600 6.90843 -0.35243

21 IISCTCPTV 6.58000 6.66487 -0.08487

22 FLGGTPVCL 6.62300 6.87695 -0.25395

23 ALIHHNTHL 6.62300 6.79106 -0.16806

24 NLSWLSLDV 6.63900 6.04824 0.59076

25 YMIMVKCWM 6.66300 6.65322 0.00978

26 VLQAGFFLL 6.68200 7.04303 -0.36103

27 GTLGIVCPI 6.71400 6.51952 0.19448

28 VILGVLLLI 6.78500 7.47415 -0.68915

29 VTWHRYHLL 6.79300 6.55933 0.23367

30 PLLPIFFCL 6.79600 7.52413 -0.72813

31 TLGIVCPIC 6.81500 5.95427 0.86073

32 CLTSTVQLV 6.83200 7.10636 -0.27436

33 ILLLCLIFL 6.84500 6.78848 0.05652

34 FAFRDLCIV 6.88600 6.66581 0.22019

35 FLEPGPVTA 6.89800 7.48985 -0.59185

36 ALAKAAAAA 6.94700 6.80220 0.14480

37 LMAVVLASL 6.95400 7.49499 -0.54099

38 YVITTQHWL 6.98300 6.34113 0.64187

39 LLCLIFLLV 6.99600 7.50639 -0.51039

40 ITAQVPFSV 7.02000 6.66529 0.35471

41 YLEPGPVTL 7.05800 7.14956 -0.09156

42 YTDQVPFSV 7.06600 7.07167 -0.00567

43 NLYVSLLLL 7.11400 6.98084 0.13316

44 ILHNGAYSL 7.12700 7.35015 -0.22315

45 SIISAVVGI 7.15900 7.30348 -0.14448

46 VVMGTLVAL 7.17400 7.31468 -0.14068

47 YLEPGPVTI 7.18700 7.16672 0.02028

48 GLSRYVARL 7.24800 7.46161 -0.21361

49 LLAQFTSAI 7.30100 7.43121 -0.13021

50 VLLDYQGML 7.32800 7.59217 -0.26417

51 YLEPGPVTV 7.34200 7.40705 -0.06505

52 ILSPFMPLL 7.34700 7.14202 0.20498

53 YLSPGPVTA 7.38300 7.55690 -0.17390

54 IIDQVPFSV 7.39800 7.65226 -0.25426

55 SVYDFFVWL 7.44400 7.36590 0.07810

56 ITWQVPFSV 7.46300 7.43961 0.02339

57 ITYQVPFSV 7.48000 7.65934 -0.17934

58 GLYSSTVPV 7.48100 7.62780 -0.14680

59 VMGTLVALV 7.55300 7.23756 0.31544

60 LLLCLIFLL 7.58500 7.14498 0.44002

61 SLDDYNHLV 7.58500 7.17396 0.41104

62 VLIQRNPQL 7.64400 6.94861 0.69539

63 SLYADSPSV 7.65800 7.70828 -0.05028

64 ILSQVPFSV 7.69900 7.64724 0.05176

65 IMDQVPFSV 7.71900 8.03286 -0.31386

66 QLFEDNYAL 7.76400 7.47258 0.29142

67 ALMDKSLHV 7.77000 7.52395 0.24605

68 YAIDLPVSV 7.79600 7.60251 0.19349

69 FVWLHYYSV 7.82400 8.11684 -0.29284

70 MLGTHTMEV 7.84500 7.31708 0.52792

71 LLFGYPVYV 7.88600 8.02788 -0.14188

72 ILKEPVHGV 7.92100 7.59115 0.32985

73 YLMPGPVTV 7.93200 7.91419 0.01781

74 WLDQVPFSV 7.93900 7.95232 -0.01332

75 KTWGQYWQV 7.95500 7.69067 0.26433

76 ALMPLYACI 8.00000 7.43968 0.56032

77 YLAPGPVTA 8.03200 7.63706 0.39494

78 YLYPGPVTV 8.05100 8.31166 -0.26066

79 LLMGTLGIV 8.09700 7.67745 0.41955

80 YLWPGPVTV 8.12500 8.09193 0.03307

81 FLLTRILTI 8.14900 7.88092 0.26808

82 GLLGWSPQA 8.23700 8.21377 0.02323

83 ILYQVPFSV 8.31000 8.72146 -0.41146

84 GILTVILGV 8.34700 7.83901 0.50799

85 NMVPFFPPV 8.39800 8.08581 0.31219

86 ILDQVPFSV 8.48100 7.69059 0.79041

87 YLFPGPVTA 8.49500 8.34457 0.15043

88 YLDQVPFSV 8.63800 8.13379 0.50421

89 ILFQVPFSV 8.69900 8.43492 0.26408

90 ILWQVPFSV 8.77000 8.50173 0.26827

Correlation Coeffecient and Stand Error

CORL: 0.887125 CORL~2: 0.786991

RES: 0.366883 SEE: 0.038673

Statistical indices of Training calculation:

ITERATION (B): 42

No. Sequence Expt. Act Predicted Act Difference

1 VALVGLFVL 5.14800 5.75135 -0.60335

2 GTLVALVGL 5.34200 5.93397 -0.59197

3 LQTTIHDII 5.50100 5.80923 -0.30823

4 SLHVGTQCA 5.84200 6.15248 -0.31048

5 ALPYWNFAT 5.86900 6.64383 -0.77483

6 SLNFMGYVI 5.88100 5.95942 -0.07842

7 NLQSLTNLL 6.00000 6.69859 -0.69859

8 FVTWHRYHL 6.02500 5.72499 0.30001

9 DPKVKQWPL 6.17600 5.74049 0.43551

10 ITSQVPFSV 6.19600 6.58491 -0.38891

11 ALAKAAAAI 6.21100 6.24144 -0.03044

12 GLGQVPLIV 6.30100 6.56474 -0.26374

13 MLDLQPETT 6.33500 6.85564 -0.52064

14 LLSSNLSWL 6.34200 6.35013 -0.00813

15 GLACHQLCA 6.38000 6.05379 0.32621

16 LIGNESFAL 6.41500 7.05514 -0.64014

17 ALAKAAAAV 6.41900 6.48219 -0.06319

18 LLAVGATKV 6.47700 6.51011 -0.03311

19 ALAKAAAAL 6.51100 6.22426 0.28674

20 WILRGTSFV 6.55600 6.90806 -0.35206

21 IISCTCPTV 6.58000 6.66416 -0.08416

22 FLGGTPVCL 6.62300 6.87645 -0.25345

23 ALIHHNTHL 6.62300 6.79057 -0.16757

24 NLSWLSLDV 6.63900 6.04683 0.59217

25 YMIMVKCWM 6.66300 6.65171 0.01129

26 VLQAGFFLL 6.68200 7.04261 -0.36061

27 GTLGIVCPI 6.71400 6.51910 0.19490

28 VILGVLLLI 6.78500 7.47423 -0.68923

29 VTWHRYHLL 6.79300 6.55872 0.23428

30 PLLPIFFCL 6.79600 7.52403 -0.72803

31 TLGIVCPIC 6.81500 5.95260 0.86240

32 CLTSTVQLV 6.83200 7.10634 -0.27434

33 ILLLCLIFL 6.84500 6.78746 0.05754

34 FAFRDLCIV 6.88600 6.66557 0.22043

35 FLEPGPVTA 6.89800 7.49032 -0.59232

36 ALAKAAAAA 6.94700 6.80220 0.14480

37 LMAVVLASL 6.95400 7.49498 -0.54098

38 YVITTQHWL 6.98300 6.34009 0.64291

39 LLCLIFLLV 6.99600 7.50631 -0.51031

40 ITAQVPFSV 7.02000 6.66515 0.35485

41 YLEPGPVTL 7.05800 7.14923 -0.09123

42 YTDQVPFSV 7.06600 7.07188 -0.00588

43 NLYVSLLLL 7.11400 6.98020 0.13380

44 ILHNGAYSL 7.12700 7.35018 -0.22318

45 SIISAVVGI 7.15900 7.30363 -0.14463

46 VVMGTLVAL 7.17400 7.31489 -0.14089

47 YLEPGPVTI 7.18700 7.16641 0.02059

48 GLSRYVARL 7.24800 7.46187 -0.21387

49 LLAQFTSAI 7.30100 7.43138 -0.13038

50 VLLDYQGML 7.32800 7.59240 -0.26440

51 YLEPGPVTV 7.34200 7.40717 -0.06517

52 ILSPFMPLL 7.34700 7.14163 0.20537

53 YLSPGPVTA 7.38300 7.55742 -0.17442

54 IIDQVPFSV 7.39800 7.65277 -0.25477

55 SVYDFFVWL 7.44400 7.36595 0.07805

56 ITWQVPFSV 7.46300 7.44017 0.02283

57 ITYQVPFSV 7.48000 7.66010 -0.18010

58 GLYSSTVPV 7.48100 7.62834 -0.14734

59 VMGTLVALV 7.55300 7.23750 0.31550

60 LLLCLIFLL 7.58500 7.14434 0.44066

61 SLDDYNHLV 7.58500 7.17407 0.41093

62 VLIQRNPQL 7.64400 6.94821 0.69579

63 SLYADSPSV 7.65800 7.70895 -0.05095

64 ILSQVPFSV 7.69900 7.64773 0.05127

65 IMDQVPFSV 7.71900 8.03362 -0.31462

66 QLFEDNYAL 7.76400 7.47265 0.29135

67 ALMDKSLHV 7.77000 7.52441 0.24559

68 YAIDLPVSV 7.79600 7.60342 0.19258

69 FVWLHYYSV 7.82400 8.11756 -0.29356

70 MLGTHTMEV 7.84500 7.31722 0.52778

71 LLFGYPVYV 7.88600 8.02855 -0.14255

72 ILKEPVHGV 7.92100 7.59163 0.32937

73 YLMPGPVTV 7.93200 7.91477 0.01723

74 WLDQVPFSV 7.93900 7.95308 -0.01408

75 KTWGQYWQV 7.95500 7.69148 0.26352

76 ALMPLYACI 8.00000 7.43958 0.56042

77 YLAPGPVTA 8.03200 7.63766 0.39434

78 YLYPGPVTV 8.05100 8.31261 -0.26161

79 LLMGTLGIV 8.09700 7.67793 0.41907

80 YLWPGPVTV 8.12500 8.09267 0.03233

81 FLLTRILTI 8.14900 7.88137 0.26763

82 GLLGWSPQA 8.23700 8.21520 0.02180

83 ILYQVPFSV 8.31000 8.72293 -0.41293

84 GILTVILGV 8.34700 7.83973 0.50727

85 NMVPFFPPV 8.39800 8.08650 0.31150

86 ILDQVPFSV 8.48100 7.69112 0.78988

87 YLFPGPVTA 8.49500 8.34581 0.14919

88 YLDQVPFSV 8.63800 8.13470 0.50330

89 ILFQVPFSV 8.69900 8.43612 0.26288

90 ILWQVPFSV 8.77000 8.50299 0.26701

Correlation Coeffecient and Stand Error

CORL: 0.887125 CORL~2: 0.786991

RES: 0.366882 SEE: 0.038673

Statistical indices of Training calculation:

ITERATION (A): 43

No. Sequence Expt. Act Predicted Act Difference

1 VALVGLFVL 5.14800 5.75288 -0.60488

2 GTLVALVGL 5.34200 5.93524 -0.59324

3 LQTTIHDII 5.50100 5.81078 -0.30978

4 SLHVGTQCA 5.84200 6.15373 -0.31173

5 ALPYWNFAT 5.86900 6.64441 -0.77541

6 SLNFMGYVI 5.88100 5.96075 -0.07975

7 NLQSLTNLL 6.00000 6.69915 -0.69915

8 FVTWHRYHL 6.02500 5.72658 0.29842

9 DPKVKQWPL 6.17600 5.74187 0.43413

10 ITSQVPFSV 6.19600 6.58544 -0.38944

11 ALAKAAAAI 6.21100 6.24249 -0.03149

12 GLGQVPLIV 6.30100 6.56536 -0.26436

13 MLDLQPETT 6.33500 6.85607 -0.52107

14 LLSSNLSWL 6.34200 6.35108 -0.00908

15 GLACHQLCA 6.38000 6.05530 0.32470

16 LIGNESFAL 6.41500 7.05540 -0.64040

17 ALAKAAAAV 6.41900 6.48300 -0.06400

18 LLAVGATKV 6.47700 6.51087 -0.03387

19 ALAKAAAAL 6.51100 6.22534 0.28566

20 WILRGTSFV 6.55600 6.90842 -0.35242

21 IISCTCPTV 6.58000 6.66487 -0.08487

22 FLGGTPVCL 6.62300 6.87684 -0.25384

23 ALIHHNTHL 6.62300 6.79104 -0.16804

24 NLSWLSLDV 6.63900 6.04810 0.59090

25 YMIMVKCWM 6.66300 6.65232 0.01068

26 VLQAGFFLL 6.68200 7.04287 -0.36087

27 GTLGIVCPI 6.71400 6.51984 0.19416

28 VILGVLLLI 6.78500 7.47403 -0.68903

29 VTWHRYHLL 6.79300 6.55936 0.23364

30 PLLPIFFCL 6.79600 7.52392 -0.72792

31 TLGIVCPIC 6.81500 5.95390 0.86110

32 CLTSTVQLV 6.83200 7.10634 -0.27434

33 ILLLCLIFL 6.84500 6.78789 0.05711

34 FAFRDLCIV 6.88600 6.66607 0.21993

35 FLEPGPVTA 6.89800 7.49020 -0.59220

36 ALAKAAAAA 6.94700 6.80270 0.14430

37 LMAVVLASL 6.95400 7.49463 -0.54063

38 YVITTQHWL 6.98300 6.34112 0.64188

39 LLCLIFLLV 6.99600 7.50597 -0.50997

40 ITAQVPFSV 7.02000 6.66557 0.35443

41 YLEPGPVTL 7.05800 7.14946 -0.09146

42 YTDQVPFSV 7.06600 7.07189 -0.00589

43 NLYVSLLLL 7.11400 6.98050 0.13350

44 ILHNGAYSL 7.12700 7.35008 -0.22308

45 SIISAVVGI 7.15900 7.30360 -0.14460

46 VVMGTLVAL 7.17400 7.31481 -0.14081

47 YLEPGPVTI 7.18700 7.16660 0.02040

48 GLSRYVARL 7.24800 7.46164 -0.21364

49 LLAQFTSAI 7.30100 7.43113 -0.13013

50 VLLDYQGML 7.32800 7.59208 -0.26408

51 YLEPGPVTV 7.34200 7.40711 -0.06511

52 ILSPFMPLL 7.34700 7.14185 0.20515

53 YLSPGPVTA 7.38300 7.55725 -0.17425

54 IIDQVPFSV 7.39800 7.65231 -0.25431

55 SVYDFFVWL 7.44400 7.36585 0.07815

56 ITWQVPFSV 7.46300 7.43979 0.02321

57 ITYQVPFSV 7.48000 7.65950 -0.17950

58 GLYSSTVPV 7.48100 7.62801 -0.14701

59 VMGTLVALV 7.55300 7.23751 0.31549

60 LLLCLIFLL 7.58500 7.14460 0.44040

61 SLDDYNHLV 7.58500 7.17417 0.41083

62 VLIQRNPQL 7.64400 6.94850 0.69550

63 SLYADSPSV 7.65800 7.70847 -0.05047

64 ILSQVPFSV 7.69900 7.64724 0.05176

65 IMDQVPFSV 7.71900 8.03266 -0.31366

66 QLFEDNYAL 7.76400 7.47247 0.29153

67 ALMDKSLHV 7.77000 7.52404 0.24596

68 YAIDLPVSV 7.79600 7.60294 0.19306

69 FVWLHYYSV 7.82400 8.11668 -0.29268

70 MLGTHTMEV 7.84500 7.31716 0.52784

71 LLFGYPVYV 7.88600 8.02766 -0.14166

72 ILKEPVHGV 7.92100 7.59118 0.32982

73 YLMPGPVTV 7.93200 7.91417 0.01783

74 WLDQVPFSV 7.93900 7.95225 -0.01325

75 KTWGQYWQV 7.95500 7.69090 0.26410

76 ALMPLYACI 8.00000 7.43957 0.56043

77 YLAPGPVTA 8.03200 7.63738 0.39462

78 YLYPGPVTV 8.05100 8.31162 -0.26062

79 LLMGTLGIV 8.09700 7.67740 0.41960

80 YLWPGPVTV 8.12500 8.09191 0.03309

81 FLLTRILTI 8.14900 7.88081 0.26819

82 GLLGWSPQA 8.23700 8.21417 0.02283

83 ILYQVPFSV 8.31000 8.72131 -0.41131

84 GILTVILGV 8.34700 7.83922 0.50778

85 NMVPFFPPV 8.39800 8.08577 0.31223

86 ILDQVPFSV 8.48100 7.69058 0.79042

87 YLFPGPVTA 8.49500 8.34480 0.15020

88 YLDQVPFSV 8.63800 8.13369 0.50431

89 ILFQVPFSV 8.69900 8.43479 0.26421

90 ILWQVPFSV 8.77000 8.50160 0.26840

Correlation Coeffecient and Stand Error

CORL: 0.887126 CORL~2: 0.786993

RES: 0.366881 SEE: 0.038673

Statistical indices of Training calculation:

ITERATION (B): 43

No. Sequence Expt. Act Predicted Act Difference

1 VALVGLFVL 5.14800 5.75161 -0.60361

2 GTLVALVGL 5.34200 5.93421 -0.59221

3 LQTTIHDII 5.50100 5.80966 -0.30866

4 SLHVGTQCA 5.84200 6.15295 -0.31095

5 ALPYWNFAT 5.86900 6.64364 -0.77464

6 SLNFMGYVI 5.88100 5.95912 -0.07812

7 NLQSLTNLL 6.00000 6.69864 -0.69864

8 FVTWHRYHL 6.02500 5.72483 0.30017

9 DPKVKQWPL 6.17600 5.74050 0.43550

10 ITSQVPFSV 6.19600 6.58524 -0.38924

11 ALAKAAAAI 6.21100 6.24159 -0.03059

12 GLGQVPLIV 6.30100 6.56477 -0.26377

13 MLDLQPETT 6.33500 6.85576 -0.52076

14 LLSSNLSWL 6.34200 6.35014 -0.00814

15 GLACHQLCA 6.38000 6.05427 0.32573

16 LIGNESFAL 6.41500 7.05520 -0.64020

17 ALAKAAAAV 6.41900 6.48249 -0.06349

18 LLAVGATKV 6.47700 6.51023 -0.03323

19 ALAKAAAAL 6.51100 6.22442 0.28658

20 WILRGTSFV 6.55600 6.90809 -0.35209

21 IISCTCPTV 6.58000 6.66423 -0.08423

22 FLGGTPVCL 6.62300 6.87638 -0.25338

23 ALIHHNTHL 6.62300 6.79059 -0.16759

24 NLSWLSLDV 6.63900 6.04682 0.59218

25 YMIMVKCWM 6.66300 6.65094 0.01206

26 VLQAGFFLL 6.68200 7.04249 -0.36049

27 GTLGIVCPI 6.71400 6.51946 0.19454

28 VILGVLLLI 6.78500 7.47411 -0.68911

29 VTWHRYHLL 6.79300 6.55880 0.23420

30 PLLPIFFCL 6.79600 7.52383 -0.72783

31 TLGIVCPIC 6.81500 5.95237 0.86263

32 CLTSTVQLV 6.83200 7.10631 -0.27431

33 ILLLCLIFL 6.84500 6.78695 0.05805

34 FAFRDLCIV 6.88600 6.66585 0.22015

35 FLEPGPVTA 6.89800 7.49064 -0.59264

36 ALAKAAAAA 6.94700 6.80270 0.14430

37 LMAVVLASL 6.95400 7.49461 -0.54061

38 YVITTQHWL 6.98300 6.34017 0.64283

39 LLCLIFLLV 6.99600 7.50590 -0.50990

40 ITAQVPFSV 7.02000 6.66544 0.35456

41 YLEPGPVTL 7.05800 7.14915 -0.09115

42 YTDQVPFSV 7.06600 7.07207 -0.00607

43 NLYVSLLLL 7.11400 6.97992 0.13408

44 ILHNGAYSL 7.12700 7.35011 -0.22311

45 SIISAVVGI 7.15900 7.30373 -0.14473

46 VVMGTLVAL 7.17400 7.31501 -0.14101

47 YLEPGPVTI 7.18700 7.16633 0.02067

48 GLSRYVARL 7.24800 7.46188 -0.21388

49 LLAQFTSAI 7.30100 7.43128 -0.13028

50 VLLDYQGML 7.32800 7.59229 -0.26429

51 YLEPGPVTV 7.34200 7.40722 -0.06522

52 ILSPFMPLL 7.34700 7.14149 0.20551

53 YLSPGPVTA 7.38300 7.55773 -0.17473

54 IIDQVPFSV 7.39800 7.65277 -0.25477

55 SVYDFFVWL 7.44400 7.36590 0.07810

56 ITWQVPFSV 7.46300 7.44031 0.02269

57 ITYQVPFSV 7.48000 7.66020 -0.18020

58 GLYSSTVPV 7.48100 7.62851 -0.14751

59 VMGTLVALV 7.55300 7.23745 0.31555

60 LLLCLIFLL 7.58500 7.14402 0.44098

61 SLDDYNHLV 7.58500 7.17427 0.41073

62 VLIQRNPQL 7.64400 6.94814 0.69586

63 SLYADSPSV 7.65800 7.70909 -0.05109

64 ILSQVPFSV 7.69900 7.64769 0.05131

65 IMDQVPFSV 7.71900 8.03335 -0.31435

66 QLFEDNYAL 7.76400 7.47253 0.29147

67 ALMDKSLHV 7.77000 7.52446 0.24554

68 YAIDLPVSV 7.79600 7.60377 0.19223

69 FVWLHYYSV 7.82400 8.11734 -0.29334

70 MLGTHTMEV 7.84500 7.31729 0.52771

71 LLFGYPVYV 7.88600 8.02827 -0.14227

72 ILKEPVHGV 7.92100 7.59162 0.32938

73 YLMPGPVTV 7.93200 7.91470 0.01730

74 WLDQVPFSV 7.93900 7.95294 -0.01394

75 KTWGQYWQV 7.95500 7.69164 0.26336

76 ALMPLYACI 8.00000 7.43947 0.56053

77 YLAPGPVTA 8.03200 7.63793 0.39407

78 YLYPGPVTV 8.05100 8.31248 -0.26148

79 LLMGTLGIV 8.09700 7.67784 0.41916

80 YLWPGPVTV 8.12500 8.09259 0.03241

81 FLLTRILTI 8.14900 7.88121 0.26779

82 GLLGWSPQA 8.23700 8.21548 0.02152

83 ILYQVPFSV 8.31000 8.72265 -0.41265

84 GILTVILGV 8.34700 7.83987 0.50713

85 NMVPFFPPV 8.39800 8.08641 0.31159

86 ILDQVPFSV 8.48100 7.69106 0.78994

87 YLFPGPVTA 8.49500 8.34594 0.14906

88 YLDQVPFSV 8.63800 8.13452 0.50348

89 ILFQVPFSV 8.69900 8.43590 0.26310

90 ILWQVPFSV 8.77000 8.50275 0.26725

Correlation Coeffecient and Stand Error

CORL: 0.887126 CORL~2: 0.786993

RES: 0.366881 SEE: 0.038673

Statistical indices of Training calculation:

ITERATION (A): 44

No. Sequence Expt. Act Predicted Act Difference

1 VALVGLFVL 5.14800 5.75300 -0.60500

2 GTLVALVGL 5.34200 5.93537 -0.59337

3 LQTTIHDII 5.50100 5.81108 -0.31008

4 SLHVGTQCA 5.84200 6.15409 -0.31209

5 ALPYWNFAT 5.86900 6.64417 -0.77517

6 SLNFMGYVI 5.88100 5.96034 -0.07934

7 NLQSLTNLL 6.00000 6.69915 -0.69915

8 FVTWHRYHL 6.02500 5.72628 0.29872

9 DPKVKQWPL 6.17600 5.74177 0.43423

10 ITSQVPFSV 6.19600 6.58572 -0.38972

11 ALAKAAAAI 6.21100 6.24255 -0.03155

12 GLGQVPLIV 6.30100 6.56533 -0.26433

13 MLDLQPETT 6.33500 6.85615 -0.52115

14 LLSSNLSWL 6.34200 6.35101 -0.00901

15 GLACHQLCA 6.38000 6.05565 0.32435

16 LIGNESFAL 6.41500 7.05544 -0.64044

17 ALAKAAAAV 6.41900 6.48323 -0.06423

18 LLAVGATKV 6.47700 6.51092 -0.03392

19 ALAKAAAAL 6.51100 6.22541 0.28559

20 WILRGTSFV 6.55600 6.90842 -0.35242

21 IISCTCPTV 6.58000 6.66488 -0.08488

22 FLGGTPVCL 6.62300 6.87674 -0.25374

23 ALIHHNTHL 6.62300 6.79101 -0.16801

24 NLSWLSLDV 6.63900 6.04798 0.59102

25 YMIMVKCWM 6.66300 6.65149 0.01151

26 VLQAGFFLL 6.68200 7.04272 -0.36072

27 GTLGIVCPI 6.71400 6.52014 0.19386

28 VILGVLLLI 6.78500 7.47393 -0.68893

29 VTWHRYHLL 6.79300 6.55939 0.23361

30 PLLPIFFCL 6.79600 7.52373 -0.72773

31 TLGIVCPIC 6.81500 5.95356 0.86144

32 CLTSTVQLV 6.83200 7.10632 -0.27432

33 ILLLCLIFL 6.84500 6.78734 0.05766

34 FAFRDLCIV 6.88600 6.66631 0.21969

35 FLEPGPVTA 6.89800 7.49052 -0.59252

36 ALAKAAAAA 6.94700 6.80316 0.14384

37 LMAVVLASL 6.95400 7.49430 -0.54030

38 YVITTQHWL 6.98300 6.34111 0.64189

39 LLCLIFLLV 6.99600 7.50559 -0.50959

40 ITAQVPFSV 7.02000 6.66582 0.35418

41 YLEPGPVTL 7.05800 7.14936 -0.09136

42 YTDQVPFSV 7.06600 7.07208 -0.00608

43 NLYVSLLLL 7.11400 6.98020 0.13380

44 ILHNGAYSL 7.12700 7.35002 -0.22302

45 SIISAVVGI 7.15900 7.30370 -0.14470

46 VVMGTLVAL 7.17400 7.31494 -0.14094

47 YLEPGPVTI 7.18700 7.16650 0.02050

48 GLSRYVARL 7.24800 7.46167 -0.21367

49 LLAQFTSAI 7.30100 7.43105 -0.13005

50 VLLDYQGML 7.32800 7.59200 -0.26400

51 YLEPGPVTV 7.34200 7.40718 -0.06518

52 ILSPFMPLL 7.34700 7.14169 0.20531

53 YLSPGPVTA 7.38300 7.55757 -0.17457

54 IIDQVPFSV 7.39800 7.65235 -0.25435

55 SVYDFFVWL 7.44400 7.36581 0.07819

56 ITWQVPFSV 7.46300 7.43996 0.02304

57 ITYQVPFSV 7.48000 7.65965 -0.17965

58 GLYSSTVPV 7.48100 7.62821 -0.14721

59 VMGTLVALV 7.55300 7.23746 0.31554

60 LLLCLIFLL 7.58500 7.14426 0.44074

61 SLDDYNHLV 7.58500 7.17436 0.41064

62 VLIQRNPQL 7.64400 6.94840 0.69560

63 SLYADSPSV 7.65800 7.70865 -0.05065

64 ILSQVPFSV 7.69900 7.64724 0.05176

65 IMDQVPFSV 7.71900 8.03247 -0.31347

66 QLFEDNYAL 7.76400 7.47236 0.29164

67 ALMDKSLHV 7.77000 7.52413 0.24587

68 YAIDLPVSV 7.79600 7.60333 0.19267

69 FVWLHYYSV 7.82400 8.11653 -0.29253

70 MLGTHTMEV 7.84500 7.31723 0.52777

71 LLFGYPVYV 7.88600 8.02746 -0.14146

72 ILKEPVHGV 7.92100 7.59120 0.32980

73 YLMPGPVTV 7.93200 7.91415 0.01785

74 WLDQVPFSV 7.93900 7.95218 -0.01318

75 KTWGQYWQV 7.95500 7.69111 0.26389

76 ALMPLYACI 8.00000 7.43946 0.56054

77 YLAPGPVTA 8.03200 7.63767 0.39433

78 YLYPGPVTV 8.05100 8.31158 -0.26058

79 LLMGTLGIV 8.09700 7.67736 0.41964

80 YLWPGPVTV 8.12500 8.09188 0.03312

81 FLLTRILTI 8.14900 7.88070 0.26830

82 GLLGWSPQA 8.23700 8.21454 0.02246

83 ILYQVPFSV 8.31000 8.72117 -0.41117

84 GILTVILGV 8.34700 7.83941 0.50759
[truncated: 556,909 more chars]
